# Supplementary material for: Cooperating with machines
Source: Nat Commun. 2018 Jan 16;9:233. doi: 10.1038/s41467-017-02597-8 (PMC5770455; doi:10.1038/s41467-017-02597-8)
Supplement: Supplementary file 1 — Supplementary Information [file 41467_2017_2597_MOESM1_ESM.pdf]

Supplementary Note 1

Overview of the Supplementary Notes

This paper reports on years of effort to develop and analyze an AI algorithm that learns to effectively interact with a human partner or another machine in two-player repeated interactions in which the players have conflicting, but not fully opposed, interests. We assume that the algorithm is not given prior information about the tendencies (or identity) of its partner. Our specific goal is to find an algorithm that establish and maintain mutually cooperative relationships with people in arbitrary repeated games at levels that rival human cooperation, and has superior performance when associating with other machines. In so doing, we seek to understand the algorithmic mechanisms that facilitate this capability.

We hasten to acknowledge that the goal of such an algorithm is not cooperation in and of itself. An autonomous machine (which we assume in this work) should cooperate only if doing so maximizes its utilities, which are given to it by its stakeholder (typically its owner). However, when players repeatedly interact with each other over long periods of time in games of conflicting (i.e., mixed) interests, cooperation is very often the best policy despite the differences between the players. Thus, to have the ability to maximize payoffs in repeated games, an effective algorithm must be able to derive and establish cooperative relationships when interacting with other intelligent entities, including humans.

The algorithm we eventually produced to achieve these feats is called S# (pronounced 'S sharp'). S# interweaves S++ [1, 2] with new algorithmic mechanisms designed to give it the ability to engage in cheap talk (the sending and receiving of costless and non-binding communication signals) with its partner. The algorithm combines and builds upon decades of work by researchers in computer science, economics, and the diverse array of behavioral and social sciences by packaging diverse and complex findings and algorithmic structures from these fields in a way that hides much of the complexity of the problem. S++ is a simple expert algorithm that uses a novel version of aspiration learning to select among a finite set of experts, each of which itself uses known mathematics to produce (or learn) a strategy over the entire space of the game. This hierarchical learning structure allows the algorithm to produce rich and complex strategies within only a small number of interactions. Importantly, the hierarchical nature of the algorithm also allows it to express and interpret speech acts at a level conducive to human interaction. Our results show that these capabilities to engage in cheap talk are a critical linchpin for a machine to elicit cooperative behavior from a human partner.

Our journey in understanding, deriving, and analyzing S# is explained in detail in this supplementary information, the contents of which follow the storyline of the main article. In Supplementary Notes 2–4, we provide technical algorithmic details and analysis, including the problem description, descriptions of S#, and analysis of the performance of these algorithms when paired with other computer algorithms. Next, Supplementary Notes 5–7 describe user studies in which we paired S++, S#, and several existing machine-learning algorithms with people.

We first provide a summary of each section (or note) of the supplementary information. The Supplementary Notes themselves then follow.

Problem Formulation and Overview of Methods

In Supplementary Note 2, we give a formal description of the problem we address in this paper, and enumerate assumptions made throughout the paper. We also discuss key technical challenges that must be addressed to develop successful algorithms for interacting with people and other (unknown) machines in arbitrary repeated games. We also overview the methods that we use to evaluate the ability of algorithms to establish

effective long-term relationships with people and machines.

Evaluating Existing Algorithms for Repeated Games

Our first goal is to (1) find an algorithm that has robust performance characteristics when interacting with other algorithms in arbitrary two-player repeated games and (2) identify the algorithmic mechanisms that make it successful. Many algorithms for playing repeated games have been produced over the last several decades across many disciplines. In Supplementary Note 3, we compare the performance of 25 representative algorithms (chosen to represent various theories and methods) for playing repeated games from these disciplines. We compare these algorithms with respect to six different performance metrics across the full set of 2x2 repeated normal-form games in which the players' payoffs follow a strict preference ordering. The results of this comparison show that S++ [1, 2] is consistently among the top-performing algorithms regardless of the duration of the game, the associate, or the payoff structure of the game. Thus, we also identify the algorithmic mechanisms that cause this algorithm to be successful.

S++'s high performance in interacting with other machines (established by the results described in Supplementary Note 3) makes it a candidate algorithm for also reaching our second goal: an algorithm that learns to cooperate with people in arbitrary repeated games. We focus on this second goal in the remainder of the paper.

Descriptions of S++ and S#

In this Supplementary Note 4, we present S#. We first review S++ [1, 2], which is used to generate S#'s behavioral strategies. We then describe how S# uses S++ to generate and respond to cheap talk in order to better establish cooperative relationships with people.

User Study: Human vs. Machine 1

Supplementary Note 5 contains a description of a 58-participant user study in which we paired S++, model-based reinforcement learning (MBRL-1), and humans with each other in four repeated normal-form games (Prisoner's Dilemma, Chicken, Chaos, and Shapley's Game), each lasting approximately 50 rounds. The primary purpose of this user study was to determine the ability of S++ to consistently elicit cooperative behavior from people in repeated interactions. The results of the study showed that S++ was more effective in self play than both people and MBRL-1. Additionally it was as effective at establishing cooperative relationships with people as people were. However, both S++ and people typically failed to cooperate with human partners in these games. This failure of S++ to consistently establish cooperative relationships with people caused us to conduct additional user studies to try to determine what algorithmic characteristics would allow a machine to more effectively establish cooperative relationships with people.

User Study: Human vs. Machine 2

Given the failure of S++ to consistently establish cooperative relationships with people, we extended the algorithm with the ability to generate and voice cheap talk. We dub the resulting algorithm S#–, an early (but incomplete) version of S#. We then conducted a second (96-participant) study, carried out in two parts. This study is described in Supplementary Note 6. In Part 1 of the study, we essentially repeated user study 1, except that, in this user study, we paired S++, CFR (the core technology behind world-class computer-poker algorithms [3]), and people together in two different repeated stochastic games rather than repeated

normal-form games. The results of this study were essentially identical to the results of the first user study. In Part 2 of this user study, we allowed players to communicate with each other. Consistent with prior work [4, 5], we observed that the ability to talk to each other throughout the game vastly improved mutual cooperation among people. S#– was also able to raise its level of cooperation when paired with people, though cooperation emerged at a much slower rate than in human-human relationships.

User Study: Human vs. Machine 3

We attributed the fact that S#– learned to cooperate with people much slower than people learned to cooperate with each other to the fact that S#– did not respond to the cheap talk expressed by people. Thus, we developed S#, which not only generates and voices cheap talk, but also reacts to the cheap talk produced by its partner to help it select experts more effectively. We then conducted a final (66-participant) user study in which we paired S# and humans in three different repeated normal-form games (Prisoner's Dilemma, Chicken, and Alternator), selected to represent different families of games. This study is described in Supplementary Note 7. The results of this study showed several important outcomes. First, S# paired with S# produced, by far, the highest levels of mutual cooperation under conditions in which players could and could not cooperate. Second, people did not consistently cooperate with each other in the absence of communication (mutual cooperation was approximately 30%). However, the frequency of mutual cooperation between people doubled when people could communicate (mutual cooperation was approximately 60%). Human-S# pairings followed identical trends. In both conditions, S# was able to learn to cooperate with people as well as people cooperated with each other. These, and other results, illustrate that S# is able to successfully generate and respond to signals from a human partner so as to better elicit cooperative behaviors from people.

Supplementary Note 2

Problem Formulation and Overview of Methods

This paper deals with decision-making in scenarios in which an autonomous machine repeatedly interacts with a person or another machine over an extended period of time (of unknown duration). Life consists of many long-term interactions between two entities, including relationships between family members, neighbors, co-workers, business partners, and nations, as well as interactions between people and their laptops, handheld devices, cars, and other embedded systems. These scenarios vary widely in many dimensions, though common attributes emerge in each problem, perhaps the most salient of which is that there are often mixed (i.e., conflicting) interests between the players. While individuals can benefit from cooperating with each other, each player has incentive to not cooperate. However, because the interactions are repeated (and often for long periods of time), non-cooperative behavior is often reciprocated, which can lead to dysfunctional relationships if the individuals cannot eventually find common ground.

We note that while the emergence of cooperation is often studied in an evolutionary context, the challenge we address in this paper is very different. In this work, we are concerned with developing an algorithm that, over the course of an interaction, develops profitable relationships with partners with whom it has had no prior interactions in arbitrary repeated games. On the other hand, the evolutionary context deals with population dynamics, and hence concerns an algorithm's performance with an entire society over generations. While the two problems have some similarities, they also have substantial differences.

In this section, we give a formal problem description and enumerate key technical challenges that must be addressed to develop successful algorithms for interacting with people and other (unknown) machines in arbitrary repeated games. We also overview the methods that we use to evaluate the ability of algorithms to establish effective long-term relationships with people and machines in these games.

Formal Problem Description

In this subsection, we define the scope of interactions we consider in this paper, which are repeated stochastic games. We then describe how we measure the performance of algorithms in these games. Finally, we state a set of assumptions made throughout the paper.

Repeated Stochastic Games In this paper, we assume that interactions between players can be modeled as repeated stochastic games (RSGs). While such games do not capture every nuance of all interactions, the set of scenarios that can be modeled by repeated stochastic games is quite rich. For example, RSGs are general enough to include repeated normal-form games, repeated extensive-form games, and repeated (multi-stage) stochastic games.

We consider two-player RSGs played by players i and $-i$. An RSG consists of a set of *stage games* S. In each stage $s \in S$, both players choose an action from a finite set. Let $A(s) = A_i(s) \times A_{-i}(s)$ be the set of *joint actions* available in stage s, where $A_i(s)$ and $A_{-i}(s)$ are the action sets of players i and $-i$, respectively. In each stage, each player simultaneously selects an action from its set of actions. Once joint action $\mathbf{a} = (a_i, a_{-i})$ is played in stage s, each player receives a finite reward, denoted $r_i(s, \mathbf{a})$ and $r_{-i}(s, \mathbf{a})$, respectively. The world also transitions to some new stage s' with probability defined by $P_M(s, \mathbf{a}, s')$.

Each *round* of an RSG begins in the start stage $\hat{s} \in S$ and terminates when some goal stage $s_g \in G \subseteq S$ is reached. A new round then begins in stage $\hat{s}$. The game repeats for an unknown number of rounds.

Player i's strategy, denoted π_i, defines how it will act in each world *state*. In general-sum RSGs, it is often useful (and necessary) to define state not only in terms of stages, but also in terms of the history of the players' actions. Let H denote the set of possible joint-action histories. Then, the set of states is given by $\Sigma = S \times H$. Let $\pi_i(\sigma)$ denote the policy of player i in state $\sigma = (s, h) \in \Sigma$. That is, $\pi_i(\sigma)$ is a probability distribution over the action set $A_i(s)$. When $\pi_i(\sigma)$ places all probability on a single action $a_i \in A(s)$, the policy is said to be a *pure strategy*. Otherwise, the policy is called a *mixed strategy*.

For foundational purposes, we focus primarily on two-player, two-action normal-form games, or RSGs that have a single stage (i.e., $|S| = 1$). This allows us to fully enumerate the problem domain under the assumption that payoff functions follow strict ordinal preference orderings. However, our broader objective is to develop algorithms for RSGs in general. Thus, to demonstrate the potential of algorithms to generalize to other domains, we also conducted experiments with other game forms.

Metrics of Success The success of a player in an RSG is measured by the payoffs it receives. An ideal algorithm would maximize the sum of the payoffs it receives over all rounds of the interaction given the "algorithm" used by its partner. However, it is not possible to guarantee optimal (or even near optimal) behavior against arbitrary associates, even in simple games [6, 7, 1]. Thus, due to the difficulty in achieving, defining, and measuring optimal behavior in RSGs in general, much work on algorithmic development has focused on developing algorithms that meet certain criteria, such as convergence to Nash equilibria [8, 9, 10, 11], convergence to Pareto optimal solutions [12, 13], minimizing regret (i.e., *no regret*, also known as *universal consistency*) [14, 15, 16, 8], and being secure [8, 12][1].

Despite the allure that such properties might provide, achieving any one (or multiple) of these properties does not necessarily guarantee high payoffs in repeated general-sum games. For example, while minimizing regret seems like a good idea, regret minimization is not guaranteed to correlate with higher payoffs in repeated games – a player with lower regret often obtains lower payoffs than a player with higher regret [17, 18, 1]. As another example, many repeated general-sum games have an infinite number of Nash equilibria [19], each of which can have different values to the players. Thus, guaranteeing convergence to Nash equilibria (alone) [11, 20] does not guarantee high performance in repeated games.

Therefore, in this paper, we focus on two metrics of success:

1. *Empirical Performance*: Ultimately, the success of a player in an RSG is measured by the sum of the payoffs the player receives over the duration of the game. A successful algorithm should have high empirical performance across a broad range of games when paired with many different kinds of associates. We are interested in the best-case, worst-case, and average-case payoffs received by algorithms. We also use evolutionary dynamics and tournaments to help assess the robustness of an algorithm's empirical performance when paired with many kinds of associates.

2. *Proportion of Mutual Cooperation*: Since the level of mutual cooperation (i.e., how often both players cooperate with each other) often highly correlates with a player's empirical performance, the ability to establish cooperative relationships is a key attribute of successful algorithms. Thus, we sometimes track the level of mutual cooperation achieved by algorithms. However, we do not consider mutual cooperation as a substitute for high empirical performance, but rather as a supporting factor.

   The term cooperation has specific meaning in well-known games such as the Prisoner's Dilemma. However, in other games, the term is much more nebulous. Furthermore, mutual cooperation can be achieved in degrees; it is not necessarily an all or nothing event. However, for simplicity in this work,

[1] We omit formal definitions of these terms. Definitions of these terms are provided in standard texts on game theory.

we define mutual cooperation as the *Nash bargaining solution* of the game [21], or the solution that maximizes the product of the advantages to the players. For each of the games considered in this work, we explicitly state this solution to avoid confusion.

Assumptions Though we focus primarily on 2x2 games in much (but not all) of our analysis, the algorithms developed and analyzed in this work are designed for arbitrary two-player repeated stochastic games. This problem domain is quite general, though we do make several common assumptions:

- As is common with the vast majority of repeated game experiments in behavior economics and biology [22, 23, 24, 25, 26], we assume that the full game description is given to both players prior to the beginning of the game. Both players are told (1) the set of stages S, the set of joint actions $A(s)$ for all $s \in S$, the transition function P_M, and the reward functions $r_i(s, \mathbf{a})$, and $r_{-i}(s, \mathbf{a})$ for all $(s, \mathbf{a})$.
- Likewise, throughout the course of the game, we assume that the current stage s and the payoffs received by both players at the end of each stage are fully known to both players.
- We assume that the game is repeated for an unknown number of rounds, long enough that the players have sufficient time to develop and profit from the relationships they build. In our user studies, games are repeated for approximately 50 rounds, which is consistent with past work reporting experiments in which people play repeated games [22, 23]. Players were not informed about the length of the games.
- Unless stated otherwise, the players were not told the identity of their associate, who could be a computer following some algorithm or another person. Participants were simply told that they were interacting with another player. This helps remove biases in strategies caused by predispositions toward certain player types, thus allowing for a properly controlled experiment.
- For foundational purposes, we focus primarily on two-player, two-action normal-form games, or RSGs that have a single stage (i.e., $|S| = 1$). This allows us to fully enumerate the problem domain under the assumption that payoff functions follow strict ordinal preference orderings. However, our broader objective is to develop algorithms for RSGs in general. Thus, to demonstrate the potential of algorithms to generalize to other domains, we also conducted experiments with other game forms.

These assumptions were made to properly scope the work, though they can be relaxed in many instances.

In this paper, we deal with repeated general-sum games, which include *zero-sum games* (one player's gain is the other player's loss), *fully cooperative games* (both player's receive the same payoff in all cases), and *games of conflicting interest* (i.e., mixed-interest games – the players receive different payoffs for at least some outcomes, but their payoffs are not fully in competition with each other). Given our focus on cooperation in this paper, our analysis primarily concerns scenarios that have conflicting interests.

Technical Challenges

To develop an algorithm that is general enough to perform effectively in arbitrary RSGs played against unknown associates (and particularly with people), we must address many technical challenges. We now outline and briefly discuss some of these technical challenges.

- *Wide variety of games.* An algorithm must not be domain-specific. It must have superior performance in a wide variety of repeated games, including zero-sum games, fully cooperative games, and games

of conflicting interests. An algorithm should not be tailored to a particular game (such as the Prisoner's Dilemma), as the ability to elicit cooperative behavior from associates in one game does not necessarily entail that the same algorithm can induce cooperative behavior in another game. Furthermore, algorithms that perform effectively in zero-sum games often perform very poorly in games of conflicting interests (and vice versa). As an example, CFR, which plays a simplified version of Poker as well as people [3], cannot learn to cooperate in RSG's of conflicting interests [2] (see also results in Supplementary Note 6).

- *Multiple (even infinite) Nash equilibria.* RSGs often have multiple Nash equilibria. In fact, the folk theorem for repeated games tells us that, if the game is continued with high enough probability after each round, then many games have an infinite number of Nash equilibria of the repeated game [19]. Furthermore, it is often not possible for players to agree on which of these Nash equilibria is *best*, even if we assume "rational" players. Thus, due to these characteristics, attempts to "solve" RSGs via rationality assumptions and equilibrium computation alone are wholly insufficient. To maximize its payoffs, an algorithm must be able to learn (in real time) from its experiences, so that it can adapt to the unknown behaviors of people and other (unknown) associates. This knowledge has given raise to a large number of machine-learning algorithms for RSGs (see Supplementary Note 3).

- *Adaptive, unknown partners.* Standard machine-learning algorithms rely on stationary environments. However, when one's partner also learns, the environment is non-stationary with respect to the state-space of any practical machine-learning algorithm. Thus, straightforward applications of standard machine-learning algorithms are not guaranteed to produce effective behavior in RSGs, and often do not in practice. For example, such algorithms often learn myopic behaviors that preclude cooperation. In fact, the non-stationary nature of these environments is such that efforts to play a best response to the current estimates of the associate's strategy often produce non-cooperative behavior.

  The algorithm should be capable of establishing effective relationships with many different kinds of partners (including both itself, other algorithms, and people) whose behavioral dynamics may or may not change over time (i.e., one's partner might be learning too!). There has been a tendency to focus on analyzing the performance of algorithms against static (non-adaptive) partners. While this focus lends itself to elegant theoretical results, such results say little about whether algorithms will be able to effectively interact with people and many other machines (who's behavior is not static).

- *Large strategy spaces.* Even simple RSGs have rather large strategy spaces. As mentioned previously, an algorithm's strategy must define a policy in each state $\sigma \in S \times H$. Even if $|A_i(s)| = 2$ for all $s \in S$ and we only consider pure strategies, the size of a player's strategy space is on the order of $2^{|S||H|}$. As such, many machine-learning algorithms require thousands of rounds of experience to converge, even in extremely simple scenarios [11, 13]. Furthermore, large strategy spaces make it difficult for algorithms to reason at a high enough level to represent and reason about cooperation, reciprocity, and other important strategic considerations.

- *Limited experience (data).* Unfortunately, in order to effectively interact with people, an algorithm must be able to derive effective behaviors in scenarios it has never seen before, within only a few rounds of experience. If a machine fails to produce realistic behaviors within short timescales, people are unlikely to interact with it in the future, thus eliminating the possibility of cooperation.

- *Human attributes.* The previous technical challenges are sufficient in and of themselves. However, human-machine interactions rely on more than just algorithmic achievements. To form effective rela-

tionships with people, machines must also address human attributes, including issues related to trust, friendship, and human "ways of thinking." Addressing such challenges is not easy. For example, human cooperation appears to not be derived from sheer computational power, but rather relies on intuition [27], cultural norms [28], and pre-evolved dispositions toward cooperation [29], common-sense mechanisms that are difficult to encode in machines. On the other hand, online-learning algorithms rely on random exploration [30] to investigate the benefits of various courses of action. However, random exploration is likely to breed distrust in a human partner, thus leading to dysfunctional, non-cooperative, relationships.

These technical challenges make developing algorithms for RSGs interesting and extremely challenging. Failures to meet these challenges often cause AI algorithms to defect rather than to cooperate when cooperation and self-interest appear to be in conflict.

Evaluation Methods

Our goal is to find an algorithm that establishes effective long-term relationships with people in arbitrary games, as well as having effective and robust performance when associating with other machines. Since it is impossible to guarantee by way of analytical proof that an algorithm will act optimally in all scenarios [6, 7, 1], we are left with the impractical task of demonstrating robust and effective performance empirically. Such a task is particularly daunting in the case of interactions with people, as we can only practically design and carry out user studies to assess the ability of algorithms to interact with people in only a relatively small number of specific scenarios.

Given these practical limitations, we adopt a two-step approach to evaluating the ability of algorithms to establish effective long-term relationships with both people and machines. First, we conduct a large empirical study in which we evaluate the ability of algorithms to interact with each other in a large (and, in some sense, exhaustive) set of two-player, two-action normal-form games. Second, we leverage game-classification schemes to carefully select the representative games in which we evaluate the ability of algorithms to establish cooperative relationships with people. Selecting games in this manner gives us reasonable confidence that results could generalize to many other scenarios.

A more detailed overview of this methodology is provided in what follows. Additional details are provided in Supplementary Notes 3, 5, 6, and 7.

Step 1: A (Comprehensive) Empirical Evaluation in 2x2 Games: Algorithm vs. Algorithm Because of their ability to clearly abstract fundamental components of interactions, two-player, two-action normal-form games (i.e., 2x2 games) have been commonly used to study relationships. Such games have been categorized and studied extensively in the literature [33, 34, 35, 31, 32]. When considering only strict preferences orderings over the four game outcomes, there are 144 unique 2x2 games[2]. Supplementary Figure 1 enumerates and classifies the games according to the Robinson-Goforth topology of games [31]. The games are grouped according to their equilibrium characteristics with respect to the *one-shot game* (i.e., relationships consisting of only a single round of interaction).

The set of 144 games in the periodic table shown in Supplementary Figure 1 is comprehensive in that it includes all 2x2 game structures in which both players have strict ordinal preference orderings over the four game outcomes. While the actual payoff values assigned to the ordinal preferences do not affect Nash

[2]There are just 78 unique game structures [33] when both players have strict ordinal preference orderings. However, since a player must deal with both sides of each game (i.e., as both the *row* and *column* player) there are 144 unique scenarios that a player can face.

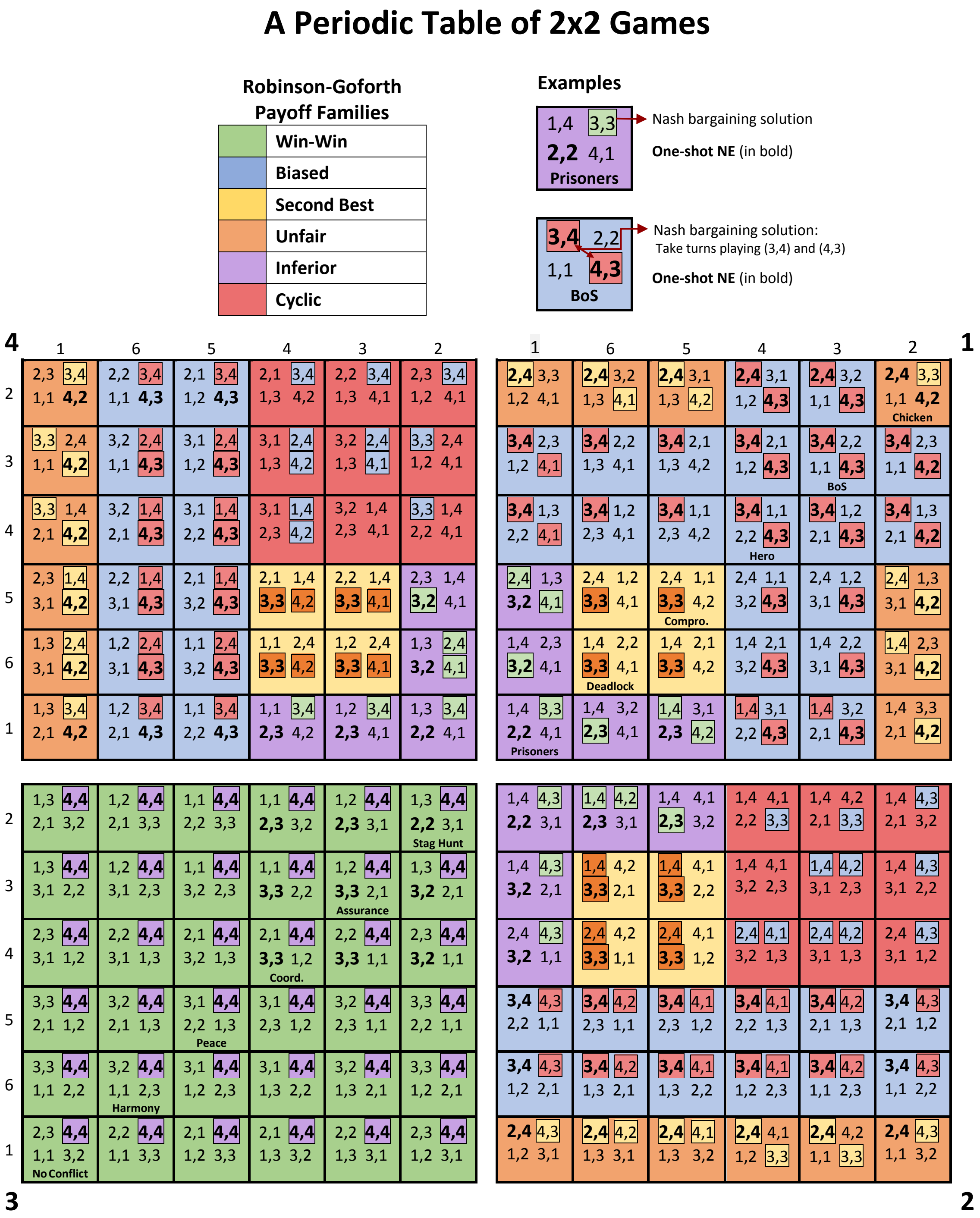

Supplementary Figure 1: A periodic table of 2x2 games based on the topology of Robinson and Goforth [31] for scenarios in which the players exhibit a strict ordinal preference ordering over the four game outcomes (specified by the values 1, 2, 3, and 4). Also adapted from the graphic developed by Bruns [32]. For each game, the pure-strategy one-shot Nash equilibria (NEs) are given in bold-face type. The solutions played in the Nash bargaining solution (NBS – i.e., the mutually cooperative solution) given the payoff values 1, 2, 3, and 4 are also highlighted, though the frequency at which each solution is played is not specified. Note that since the NBS depends on the actual payoffs and not just the preference ordering, other NBSs are possible for each game structure.

Name	Transformation from Ordinal Preferences				The 4 Scalar Values (Scaled between 0 and 1)			
	**1**	**2**	**3**	**4**				
Standard	$\mathbf{1}$	$\mathbf{2}$	$\mathbf{3}$	$\mathbf{4}$	0.000	0.333	0.667	1.000
Power-2	$\mathbf{1}^2$	$\mathbf{2}^2$	$\mathbf{3}^2$	$\mathbf{4}^2$	0.000	0.200	0.533	1.000
Power-3	$\mathbf{1}^3$	$\mathbf{2}^3$	$\mathbf{3}^3$	$\mathbf{4}^3$	0.000	0.111	0.413	1.000
Separated	$\frac{1}{2}(\mathbf{1}-1)$	$\frac{1}{2}(\mathbf{2}-1)$	$2(\mathbf{3}-1)$	$2(\mathbf{4}-1)$	0.000	0.083	0.667	1.000
Square-Root	$\sqrt{\mathbf{1}}$	$\sqrt{\mathbf{2}}$	$\sqrt{\mathbf{3}}$	$\sqrt{\mathbf{4}}$	0.000	0.414	0.732	1.000

Supplementary Table 1: The five different sets of scalar payoff values used in our comparison of algorithms. The payoff values are created using different transformations from the ordinal preferences, and are then scaled to the range $[0, 1]$, such that the lowest payoff is 0 and the highest payoff is 1. Only 3-decimal points of accuracy are shown for the scaled payoff values.

(a) Standard

	**c**	**d**
**a**	0.00, 1.00	0.67, 0.67
**b**	**0.33, 0.33**	1.00, 0.00

(b) Power-2

	**c**	**d**
**a**	0.00, 1.00	0.53, 0.53
**b**	**0.20, 0.20**	1.00, 0.00

(c) Power-3

	**c**	**d**
**a**	0.00, 1.00	0.41, 0.41
**b**	**0.11, 0.11**	1.00, 0.00

(d) Separated

	**c**	**d**
**a**	0.00, 1.00	0.67, 0.67
**b**	**0.08, 0.08**	1.00, 0.00

(e) Square-Root

	**c**	**d**
**a**	0.00, 1.00	0.73, 0.73
**b**	**0.41, 0.41**	1.00, 0.00

Supplementary Table 2: Five distinct games produced from the game structure known as the Prisoner's Dilemma (Game 111 in the Robinson-Goforth topology of 2x2 games) using the sets of payoff values shown in Supplementary Table 1. Payoffs are rounded to two decimal places. The one-shot NE is given in bold, while solutions played in the NBS are shaded in blue.

equilibrium (NE) assuming that strict preference orderings are maintained, they can, and often do, impact the behaviors of some algorithms in repeated games. Thus, for each of the 144 structurally-distinct games, we formed five different games using the sets of scalar payoff values shown in Supplementary Table 1. For example, for the structural game associated with prisoner's dilemmas (Game 111 in the Robinson-Goforth classification), we form the five different games shown in Supplementary Table 2. Creating different games in this manner creates a variety of different challenges. For example, in the game shown in Supplementary Table 2c, the players are both better off by alternating between the solutions `ac` and `bd` rather than always playing the solution `ad`, though defection (the second action for the row player and the first action for the column player) remains the dominant action for both players. Regardless, the unique one-shot NE solution (`bc`) is Pareto dominated by the NBS in each of these five games.

Given its basis on the 144 unique game structures, the resulting set of 720 2x2 games formed in this manner is a compelling and (in some sense, since it covers the full set of 2x2 games when only strict ordinal preference orderings are considered) comprehensive test-set against which algorithms can be evaluated. Thus, as a first test for evaluating algorithms, we conducted a large empirical study in which we compared the performance of 25 represented algorithms across all games in this test set. We seek an algorithm that has high and robust performance across this set of games regardless of the identity of its associate. Our evaluations in this regard are described in Supplementary Note 3.

Step 2: User Studies Evaluating Human-Algorithm Interactions in Selected Games Since an algorithm's interactions with people can only be evaluated via user study, we cannot conduct as comprehensive of a study assessing how well algorithms interact with people as we can against other algorithms. However, previous work in categorizing games based on similar equilibrium characteristics helps us to select representative (benchmark) games belonging to the more interesting payoff families. We reason that if an algorithm has consistently high performance both (1) across all games when paired with many other algorithms and (2) in the selected (benchmark) games when paired with people, we can be reasonably confident that it could have high performance across many other games when paired with people.

Several game-classification schemes for 2x2 games have been proposed in the literature [33, 34, 35, 31, 32]. One of the more compelling classification schemes is that of Robinson and Goforth [31], who group the games into six *payoff families* (Supplementary Figure 1). These payoff families are based on the one-shot NEs of the games. Of these six payoff families, some payoff families are considered more interesting than others. *Win-Win* games have consistently been categorized as uninteresting [34, 35, 32], as there is little conflict between the players (both desire the same solution). In these games, the NBS corresponds with the one-shot NE, making convergence to this solution very likely for most algorithms. Games from the *Second Best* payoff family have a similar characteristic, in that the solution played the most in the NBS corresponds to the unique one-shot NE of each game.

On the other hand, games belonging to the *Biased*, *Unfair*, *Inferior*, and *Cyclic* payoff families often present scenarios in which a one-shot NE does not correspond with the NBS. Games belonging to the *Biased* payoff family are typically characterized by one-shot NE in which one of the players gets its top choice, while the other player gets its second choice. Many of these games (particularly those shown in the upper-righthand corner of Supplementary Figure 1) have two pure one-shot NEs, with an NBS that requires the players to take turns playing these NEs. On the other hand, games grouped in the *Unfair* payoff family have one-shot NE in which one player gets its first choice while the other player gets its third choice. These NEs are Pareto optimal, but they are not always part of the NBS. This means that players may have incentives or tendencies to try to bully each other in these games. Finally, games in the *Inferior* and *Cyclic* payoff families tend to have the important similarity that the unique one-shot NE is Pareto dominated by the NBS (except in the case of the six zero-sum games). The difference between these payoff families is that games in the *Inferior* family each have a pure one-shot NE, while games in the *Cyclic* payoff family each have a mixed-strategy one-shot NE.

Though the Robinson-Goforth payoff families are compelling, alternative game classification schemes are also illuminating. For example, since we are interested in the ability of algorithms to develop cooperative relationships, our version of the periodic table of 2x2 games in Supplementary Figure 1 highlights the relationship between one-shot NEs and the NBS [21]. Based on this idea, Supplementary Table 3 presents a simple classification scheme (called *cooperative families*) in which games are grouped based on the relationship between one-shot NEs and the NBS. In this grouping, *Temptation Games* are interesting in that they cause players to decide between trying to bully their partner and playing the fair cooperative solution. On the other hand, games belonging to the *Dominated* cooperative family require players to avoid convergence to myopic (Pareto dominated) equilibria solutions to sustain cooperation (which can be enforced using trigger strategies).

Unlike the Robinson-Goforth game classification, the assignment of games to cooperative families depends on payoff values and not just preference orderings since the NBS changes based on payoff values. Supplementary Figure 2 illustrates groupings with respect to cooperative families given the five sets of payoff values provided in Supplementary Table 1.

Based on these game-classification schemes, we selected the representative games shown in Supple-

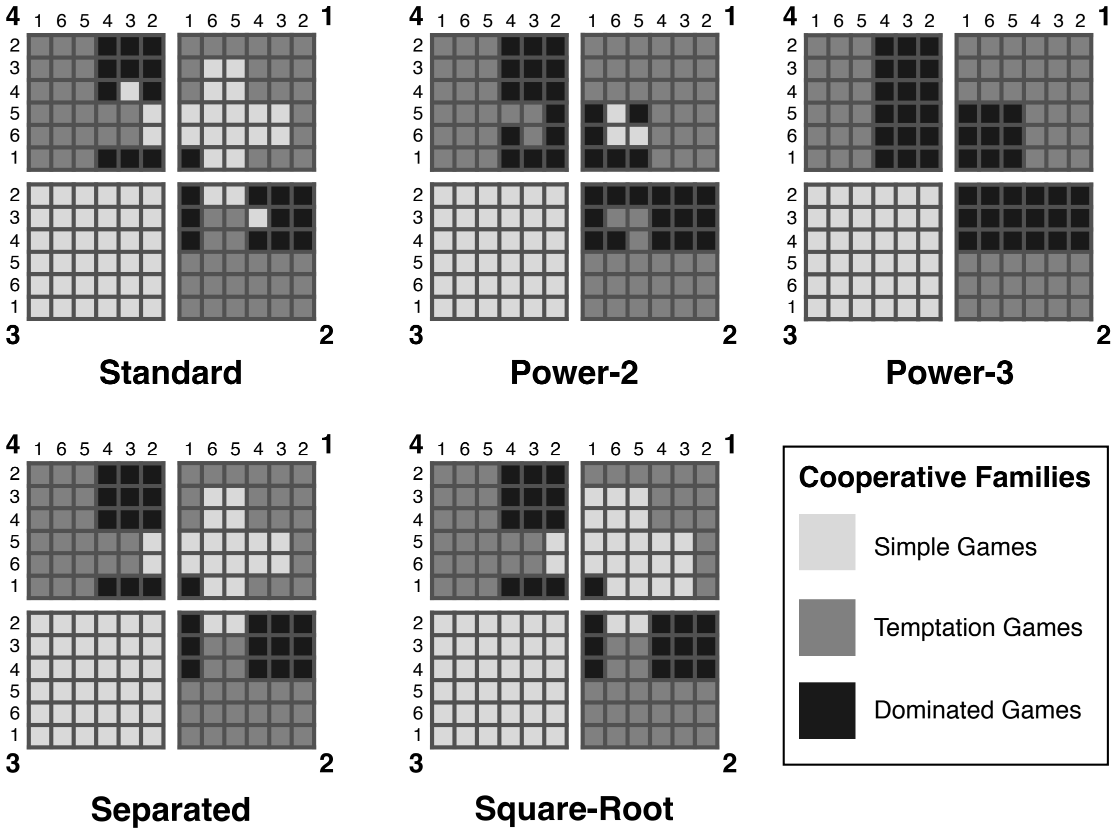

Supplementary Figure 2: The cooperative families (Supplementary Table 3) of the 144 structurally unique 2x2 games given the five different sets of payoff values given in Supplementary Table 1. For each set of payoff values, the games laid out using the Robinson-Goforth topology as in Supplementary Figure 1.

Cooperative Family	Description
Simple Games	Games in which the NBS corresponds with a one-shot NE.
Temptation Games	Games in which one player benefits (at the expense of the other player) if a one-shot NE is played instead of the NBS.
Dominated Games	Games in which the NBS Pareto dominates all one-shot NEs.

Supplementary Table 3: A simple game-classification scheme based on the comparison between one-shot Nash equilibria (NEs) and the Nash bargaining solution (NBS).

mentary Table 4 from among the more interesting[3] payoff and cooperative families. These games were each used in one or more of our user studies in which we paired algorithms with people in repeated games (see Supplementary Notes 5–7 for details). We reason that if an algorithm performs effectively when associating with a variety of different algorithms across the structurally distinct 2x2 games (under the assumption of strict ordinal preference orderings) and also establishes profitable relationships with people in these selected games, we have cause to be optimistic that the algorithm could also establish effective relationships with people in untested games that belong to the same families.

In short, while our user studies do not comprehensively evaluate the ability of algorithms to interact with people in all scenarios, this two-step approach to evaluating algorithms gives us some confidence that our results could generalize to many other scenarios.

Discussion of Selected Games

To better understand the games selected for our user studies, we discuss each games shown in Supplementary Table 4. In these games of conflicting interest, each player requires the help of the other to receive a high payoff, but each player can also potentially exploit or bully the other player. As such, to some extent, cooperation and self-interest appear to be in conflict in each of these games, though common ground can be established to give both players high payoffs. In fact, in each game, the NBS can be sustained as a NE of the repeated game if both players use trigger strategies [19].

Prisoner's Dilemma The first five games are normal-form games. The first of these games is the Prisoner's Dilemma, which is, without doubt, the most common game for studying cooperation. In this game, the dominant strategy for both players leads to the one-shot NE in which both players defect. This one-shot NE is Pareto dominated by the NBS (in which both players cooperate). Thus, it belongs to the *Inferior* payoff family and the *Dominated* cooperative family.

Chaos Like the Prisoner's Dilemma, Chaos belongs to the *Inferior* payoff family and the *Dominated* cooperative family, as the unique one-shot NE is Pareto dominated by the NBS. However, Chaos presents a distinct challenge because (a) it is a three-action game, (b) it is non-symmetric, and (c) only the row player has a dominant strategy in this game. In terms of the games shown in Supplementary Figure 1, Chaos is most like Game 431 (layer 4, row 3, column 1) in the Robinson-Goforth topology. We reason that Chaos will be more difficult for people to reason about than the Prisoner's Dilemma.

[3]Due to the perceived triviality of games belonging to the *Win-Win* and *Second Best* payoff families as well as the *Simple* cooperative family, we did not select any games from those families for our user studies.

<table>
<tr><th>Game Name</th><th>Payoff Matrix or
Graphical Representation</th><th>Robinson-Goforth
Payoff Family</th><th>Cooperative
Family</th></tr>
<tr><td>Prisoners' Dilemma</td><td><table><tr><th></th><th>c</th><th>d</th></tr><tr><th>a</th><td>0, 100</td><td>60, 60</td></tr><tr><th>b</th><td>20, 20</td><td>100, 0</td></tr></table></td><td>Inferior</td><td>Dominated</td></tr>
<tr><td>Chaos</td><td><table><tr><th></th><th>d</th><th>e</th><th>f</th></tr><tr><th>a</th><td>46, 67</td><td>24, 6</td><td>100, 0</td></tr><tr><th>b</th><td>0, 37</td><td>37, 7</td><td>1, 53</td></tr><tr><th>c</th><td>14, 69</td><td>20, 100</td><td>71, 90</td></tr></table></td><td>Inferior</td><td>Dominated</td></tr>
<tr><td>Shapley's Game</td><td><table><tr><th></th><th>d</th><th>e</th><th>f</th></tr><tr><th>a</th><td>0, 0</td><td>100, 0</td><td>0, 100</td></tr><tr><th>b</th><td>0, 100</td><td>0, 0</td><td>100, 0</td></tr><tr><th>c</th><td>100, 0</td><td>0, 100</td><td>0, 0</td></tr></table></td><td>Cyclic</td><td>Dominated</td></tr>
<tr><td>Chicken</td><td><table><tr><th></th><th>c</th><th>d</th></tr><tr><th>a</th><td>33, 100</td><td>84, 84</td></tr><tr><th>b</th><td>0, 0</td><td>100, 33</td></tr></table></td><td>Unfair</td><td>Temptation</td></tr>
<tr><td>Alternator Game</td><td><table><tr><th></th><th>d</th><th>e</th><th>f</th></tr><tr><th>a</th><td>0, 0</td><td>35, 70</td><td>100, 40</td></tr><tr><th>b</th><td>70, 35</td><td>10, 10</td><td>45, 30</td></tr><tr><th>c</th><td>40, 100</td><td>30, 45</td><td>40, 40</td></tr></table></td><td>Biased</td><td>Temptation</td></tr>
<tr><td>The SGPD</td><td>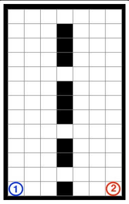</td><td>Inferior</td><td>Dominated</td></tr>
<tr><td>The Block Game</td><td>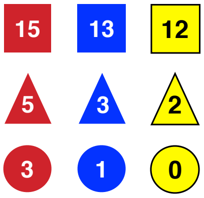
</td><td>Unfair</td><td>Temptation</td></tr>
</table>

Supplementary Table 4: Selected games and their classifications used in our user studies. The pure-strategy one-shot NEs are given in bold; the NBS for each game is highlighted in blue (multiple highlighted cells indicate that the players alternate between the specified joint actions). Note that the NBS in Shapley's Game is not unique, as many solutions produce the same value.

Shapley's Game Like Prisoner's Dilemma and Chaos, Shapley's game also belongs to the *Dominated* cooperative family since its one-shot NE (a mixed strategy that yields an expected payoff of 33.33 to both players) is Pareto dominated by the NBS solution (in which the players take turns getting 100 points). Unlike these games, however, Shapley's Game belongs to the *Cyclic* payoff family because it does not have a pure strategy one-shot NE. Additionally, the NBS requires the players to alternate between solution. Finally, neither player has a strict strict preference ordering over game outcomes, as there are ties in the players' preferences.

Chicken Though not without similarities to the Prisoner's Dilemma, Chicken has different attributes that can substantially change interaction dynamics. As such, we might expect cooperation to emerge differently in this game. Rather than having a unique one-shot NE, this game has three one-shot NE (two pure and one mixed). The row player can bully a (rational) column player by always playing action b, which essentially forces the column player to play d. The column player likewise can bully the row player by always playing c. However, these bully strategies are somewhat risky if each player tries to bully the other. The safe (maximin) strategy is to cooperate (actions a and d, respectively). Thus, in contrast to the Prisoner's Dilemma, the secure strategy leads to mutual cooperation in this game. Chicken belongs to the *Unfair* payoff family and *Temptation* cooperative family.

The Alternator Game The Alternator Game is a combination of two games: Battle of the Sexes (Game 133) and Hero (Game 144). If we remove the second option of both players, we have the game Battle of the Sexes (though with a tie between the 2nd and 3rd payoffs). If we remove the third option, we have the game Hero. Both games have two pure-strategy one-shot NEs. The full game likewise has two-pure strategy NEs, though the NBS requires the players to alternate between solutions that are not one-shot NEs. The resulting game, which belongs to the *Biased* payoff family and *Temptation* cooperative family (though note that the one-shot NEa are weakly, but not strictly, Pareto dominated by the NBS), provides a fascinating interaction in which players must find sufficient trust to coordinate between actions.

The SGPD: A Stochastic Game Prisoner's Dilemma Normal-form games have the advantage of presenting choices between cooperation and defection in simple terms. However, in many real-world scenarios, cooperation and defection are established by sequences of moves, rather than in a single step. Extensive-form and other multi-stage stochastic games can be used to model such scenarios. These games represent additional challenges for algorithm designers since they require algorithms to compute and reason about cooperation, defection, reciprocity, etc. over sequences of moves rather than in a single step.

One such multi-stage RSG is the stochastic game prisoner's dilemma (SGPD) [36], a maze game in which the high-level payoffs of the game equate to a standard prisoner's dilemma. As such, this game belongs to the same payoff and cooperative families as the Prisoner's Dilemma.

At the start of each round of the game, the players are placed in opposite corners of the maze as shown in Supplementary Figure 3. The players move (simultaneously) to adjacent cells (up, down, left, or right) with the goal of reaching the other player's start position in as few moves as possible. Each move costs a player one point, but a player receives 30 points when it reaches its goal. Once both players have arrived at their respective goals, a new round begins from the original start stage.

To reach their goals, the players must pass through one of four gates (Gates A-D). While the least-cost path to the goal is through Gate A, only one player can pass through Gate A in a round. When a player passes through Gate A, Gates A, B, and C close for the other player, and it must pass through Gate D. If both players attempt to pass through Gate A at the same time, neither is allowed passage and Gates A and

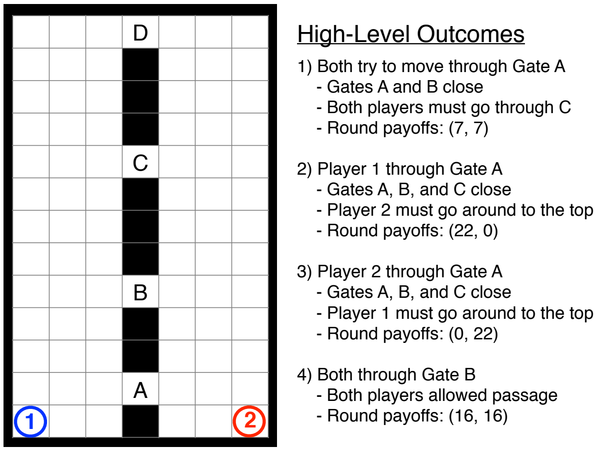

Supplementary Figure 3: The SGPD, a maze version of the prisoners' dilemma.

B close. On the other hand, both players can pass through Gates B, C, and D separately or at the same time, though Gate A closes when either player passes through Gate B.

We expect players to eventually converge to one of four solutions, which we list in descending collaborative order:

1. *(Mutual Cooperation) Both B* – Both players use Gate B, resulting in each receiving 16 points per round.

2. *Alternate between Gates A and B* – The players take turns going through Gate A, which results in an average payoff of $11 = \frac{22+0}{2}$ points per round to each player.

3. *Both go attempt to go through Gate A* – Both players attempt to go through Gate A, thus requiring each player to pass through Gate C. This gives both players 7 points.

4. *Exploitation* – One of the players always goes through Gate A, leaving the other to go through Gate D. This gives the players 22 and 0 points, respectively.

The sub-game perfect one-shot NE of this game is for both players to try to pass through Gate A, while the mutually cooperative solution (which can be sustained as a NE of the repeated game using trigger strategies) is for both players to move through Gate B.

The Block Game The Block Game, depicted in Supplementary Figure 4, is an extensive-form game. Extensive-form games have a tree-like structure in which player's take turns making moves. When played repeatedly, extensive-form games are RSGs in which the root node is the start stage and the leaf nodes are goal stages. In stages in which it is player i's turn to move, player $-i$'s action set has cardinality of 1 (i.e., $|A_{-i}(s)| = 1$); it has no ability to impact the outcome of that stage.

The Block Game is a turn-taking game in which the two players share a set of nine blocks. In each round, the players take turns selecting a block until they each have three blocks, with one player (typically the older sibling) going first in each round. If a player's three blocks form a valid set (i.e., she has all blocks of the same color, all blocks of the same shape, or none of her blocks have the same color or shape), then

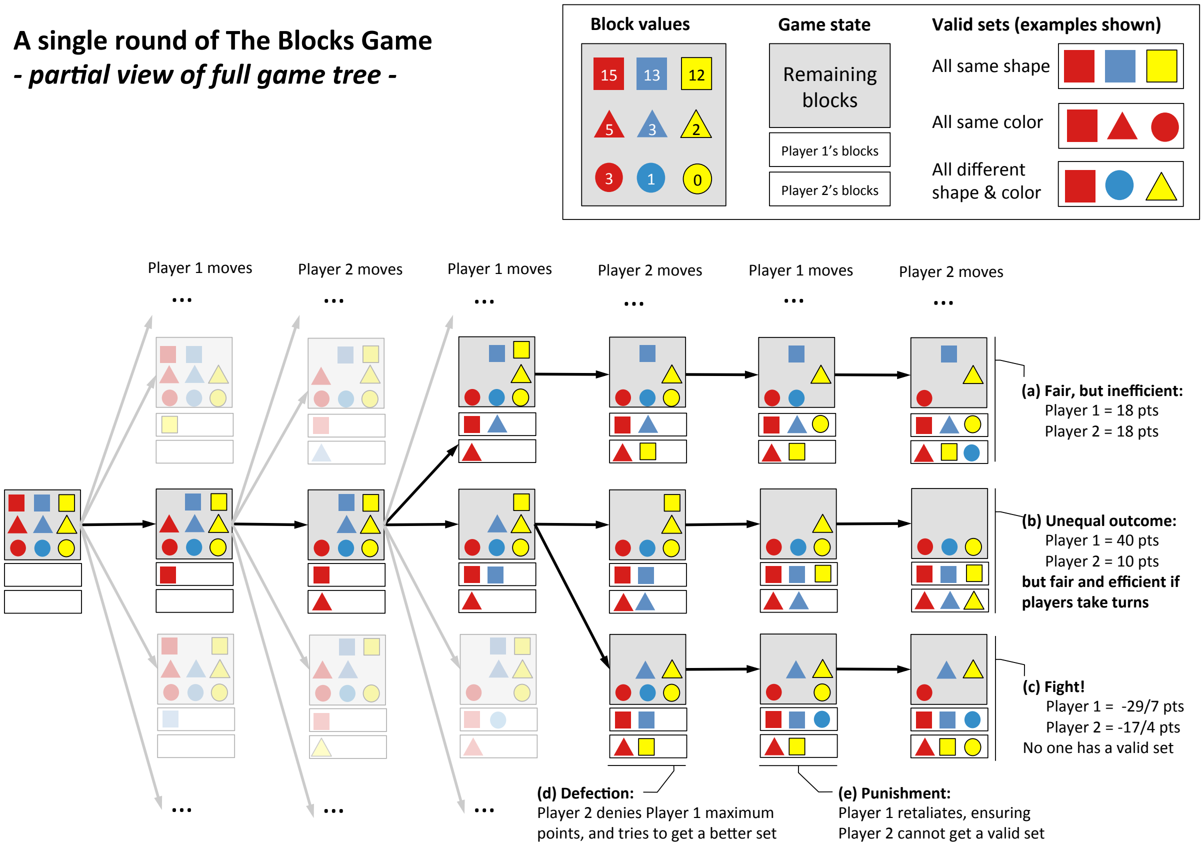

Supplementary Figure 4: An extensive-form game in which two players share a set of blocks. The two players take turns selecting blocks from a nine-piece blocks set until they each have three blocks. The goal of each player is to get a valid set of blocks with the highest value as possible, where the value of a set is determined by the sum of the numbers on the blocks. Invalid sets produce negative points, defined by the sum of the values on the blocks divided by four. (a) A fair, but inefficient outcome in which both players receive 18 points. (b) An unequal outcome in which one player receives 40 points, while the other player receives just 10 points. However, when the players take turns getting the higher payoff (selecting all the squares), this is the Nash bargaining solution of the game, producing an average payoff of 25 to both players. (c) An outcome in which neither player obtains a valid set, and hence both players lose points. (d) This particular negative outcome is brought about when player 2 defects against player 1 by taking the block that player 1 needs to complete its (most-valuable) set. (e) Player 1 then retaliates to ensure that player 2 does not get a valid set either.

her payoff in the round is the sum of the numbers on her blocks. If she fails to collect a valid set, she loses the sum of her blocks divided by 4.

Though rather simple, this game is strategically complex. Each player would like to collect all of the squares (40 points) or all of the red blocks (23 points). However, to reach either of these outcomes, the other player would have to accept either getting all of the triangles (10 points) or all of the blue blocks (17 points), respectively. Since the other player can get 18 points by taking blocks that all differ in shape and color, (s)he/it is unlikely to repeatedly accept either of those two outcomes. Thus, a player has to decide whether to try to bully the other player (possibly by punishing the other player, which might require the acceptance of outcomes producing negative payoffs in early rounds in order to get better outcomes in later rounds) or to collaborate with them in some way. This decision depends on the characteristics of the other player.

There are a number of different solutions the players can converge to in this game, which we list in descending collaborative order:

1. *(Mutual Cooperation) Alternate between* $\square$*'s and* $\triangle$*'s* – The players alternate between selecting all of the squares and all of the triangles. Thus, both players average 25 points per round ($(40 + 10)/2$).

2. *Alternate between red and blue* – The players take turns selecting the red and blue blocks (respectively) in alternating rounds, Both players average 20 points per round ($(23 + 17)/2$).

3. *All different* – The players both select blocks with no matching attributes. This always gives both players 18 points. This solution is fair, but not efficient, since alternating between $\square$'s and $\triangle$'s gives both players higher payoffs.

4. *Pure red-blue* – One of the players always takes all the red blocks (23 points), while the other player always takes the blue blocks (17 points).

5. *Pure* $\square$-$\triangle$ – One player always takes the squares (40 points), while the other player always gets the triangles (10 points).

The Block Game has many one-shot NE, including solutions that produce the *Pure* $\square$-$\triangle$ solution and the *All Different* solution (which is the sub-game perfect NE). The NBS occurs when players alternate between $\square$'s and $\triangle$'s. This solution can be sustained as a NE of the repeated game using trigger strategies. The Block game most closely equates with the *Unfair* payoff family and the *Temptation* cooperative family.

Supplementary Note 3

Evaluating Existing Algorithms in Repeated Games

Our goal is to find an algorithm that effectively establishes and maintains long-term relationships with both people and other machines in arbitrary repeated games. In this section, we address one aspect of this goal: finding an algorithm that establishes effective long-term relationships with other machines (algorithms). To do this, we analyze the capabilities of existing algorithms for playing repeated games to see if any of these algorithms meets this objective across the full set of 2x2 games in which players have strict ordinal preference orderings over the four game outcomes (Supplementary Figure 1).

Algorithms designed to play repeated games were first studied in the context of equilibrium attainment and computation. Example algorithms included Fictitious Play [37] and other forms of so-called "rational" learning [38, 8]. However, such algorithms were typically not intended to prescribe machine behavior. In the early 1980s, Axelrod conducted a computer tournament in which he compared algorithms in an iterated prisoner's dilemma [6], with the goal of finding a robust and effective way to play this game. Subsequent work in the development of algorithms for playing repeated games in evolutionary biology, economics, and the social sciences have continued to focus on strategies for playing specific games, such as prisoner's dilemmas or the Staghunt [39]. Popular algorithms studied in these disciplines have included tit-for-tat [6], win-stay-lose-shift [40], zero-determinant strategies [41], and stochastic memory-one and memory-two strategies [42].

It was not until the pioneering work of Littman, who combined reinforcement learning with concepts from game theory [43], that research in the area of learning in games was performed in mass among members of the AI community (e.g., [44, 45, 46, 47, 9, 48, 49, 11, 50, 51, 52, 53, 10, 54, 20, 55, 17, 56, 15, 57, 12, 58, 59, 60, 13, 61, 62, 63, 64, 18, 65, 1, 66, 67, 3]). As a result of this research emphasis, many general-purpose learning algorithms have been developed.

Given the goals of this paper, we are interested in determining if any of these algorithms are able to establish and maintain successful relationships in arbitrary repeated games when paired with a variety of unknown associates. Simultaneously, we are interested in understanding what characteristics of top-performing algorithms cause them to be successful. Ultimately, we seek to understand how to create an adaptive and robust (general-purpose) algorithm for repeated games that finds ways to maximize its payoffs in a wide variety of scenarios (that conform with the assumptions given in Supplementary Note 2) in which it might be placed.

Objective

As stated in Supplementary Note A, it is not possible to develop an algorithm that is guaranteed to act optimally (with respect to payoffs received) given an arbitrary game and (unknown) partner [6, 7, 1]. Despite this impossibility, our goal is to identify an algorithm (and the critical algorithmic mechanisms used by that algorithm) that performs effectively in arbitrary scenarios when paired with people and other algorithms it is likely to encounter. Thus, we seek algorithms with the following three properties:

1. *Game Independence.* The algorithm must have high performance in general, and not only in specific, targeted games. Our primary question: Is there an algorithm that has high performance across the periodic table of 2x2 games (Supplementary Figure 1)?

2. *Partner Independence.* The algorithm should be capable of establishing effective relationships with many different kinds of partners (including both itself, other algorithms, and people) whose behavioral

dynamics may or may not change over time (i.e., one's partner might be learning too!). There has been a tendency to focus on analyzing the performance of algorithms against stationary (non-adaptive) partners. While this focus lends itself to elegant theoretical results, such results say little about whether algorithms will be able to effectively interact with people and many other machines (whose strategies are typically non-stationary). In this chapter, we analyze algorithms when associating with 25 different algorithms drawn from a wide variety of families of algorithms.

3. *Fast and Sustainable.* Since people are unlikely to continue to utilize or interact with machines that do not quickly produce effective behavior, successful algorithms should learn effective behavior (in scenarios they have never seen before) within a small number of rounds of interaction. Likewise, a successful algorithm must be able to maintain a successful relationship over long periods of time.

To determine the ability of existing algorithms to obtain high empirical performance given different games, partners, and game lengths, we conducted a large empirical study in which we compared the performance of 25 representative algorithms from the literature. In so doing, we evaluated the algorithms across the full periodic table of 2x2 games at three different game lengths (100-round, 1000-round, and 50,000-round games, respectively). Comparisons were made with respect to the six metrics overviewed in Table 5, which we now formally define.

Evaluation Metrics

To measure the ability of algorithms to develop successful long-term relationships with other algorithms in arbitrary 2x2 games, we evaluated algorithms with respect to six different metrics (Supplementary Table 5). Collectively, these metrics are designed to assess the abilities of algorithms with respect to partner independence, game independence, and fast and sustainable behavior. We both score and rank algorithms with respect to each metric. We define each metric in turn.

Round-Robin Average Let J be a set of algorithms, G be a set of games, and T be the number of rounds in a game. The round-robin average is the average payoff obtained by an algorithm when it is paired with each algorithm $j \in J$ in each game $g \in G$ for T rounds. Formally, the average per-round payoff of algorithm i when it is paired with algorithm j in a T-round interaction is given by

$$v_i^j(g) = \frac{1}{T}\sum_{t=1}^{T} r_i^j(g,t), \qquad (1)$$

where $r_i^j(g,t)$ is the payoff received by algorithm i in round t of an interaction with player j in game g. Then, the round-robin average of algorithm i is given by

$$RRA_i(G,J) = \frac{1}{|G||J|}\sum_{g\in G}\sum_{j\in J} v_i^j(g), \qquad (2)$$

where $|G|$ and $|J|$ denote the cardinalities of the sets G and J, respectively.

To measure the game independence of algorithms, we observe the round-robin averages of algorithms across the full set of 2x2 games (Supplementary Figure 1), as well as across games that belong to individual payoff families. To help measure partner independence, we observe the round-robin averages of algorithms across pairings with all algorithms under consideration, as well as when associating only with partner algorithms from specific algorithm families (discussed in the subsection entitled *Algorithms* in this

Metric	Description
Round-Robin Average	The average per-round payoff of the players in the round-robin tournament when paired with all algorithms in all games.
% Best Score	The proportion of possible partner algorithms against which the algorithm has the highest per-round payoff averaged over all games.
Worst-Case Score	The average relative score of an algorithm when paired with the partner against which it has the lowest average relative score.
Replicator Dynamic	The usage rate of the algorithms over 10,000 generations of the application of the replicator dynamic.
Group-1 Tournament	A series of elimination tournaments in which algorithms are divided into four groups of equal size. The winner of the round-robin tournament played in each group advances to the championship round, which crowns the winner via another round-robin tournament.
Group-2 Tournament	A series of elimination tournaments in which algorithms are divided into four groups of equal size. The top two performers in the round-robin tournament conducted in each group advance to the semi-finals. The top two performers in the round-robin tournament involving semi-finalists advance to the championship round, which consists of a two-player round-robin tournament, the winner of which is crowned champion.

Supplementary Table 5: An overview of the metrics used to compare and contrast 25 existing algorithms for playing 2x2 games.

Supplementary Note). To measure fast and sustainable behavior, we observe round-robin averages at different game lengths. In short, we vary G, J, and T to assess the robustness of algorithms with respect to the round-robin average.

We also rank the algorithms with respect to their round-robin averages. The rank of algorithm i with respect to the round-robin average is give by

$$rank_{RRA}(i, J, G) = 1 + \sum_{j \in J} ssig(j, i, G), \quad 3$$

where $ssig(j, i, G) = 1$ if the payoffs of algorithm j are statistically higher than those of algorithm i, and $ssig(j, i, G) = 0$ otherwise. Statistical comparisons are made via pairwise comparisons (two-way t-tests) of the *relative scores* of the players ($\alpha = 0.05$). The relative score is used to reduce the variance across games so that we can focus on differences between players, while preserving the relative payoffs of the players. Formally, the relative score of algorithm i when paired with algorithm j in game g is

$$s_i^j(g) = v_i^j(g) - \bar{v}^J(g), \quad 4$$

where

$$\bar{v}^J(g) = \frac{1}{|J||J|} \sum_{i \in J} \sum_{j \in J} \bar{v}_i^j(g). \quad 5$$

In words, the relative score in game g is an algorithm's average payoff in game g relative to the performance of all algorithms in that game.

For visualization purposes, it is also useful to consider the *normalized relative score*. Formally, the normalize relative score of algorithm i when paired with algorithm j in game g is given by

$$n_i^j(g) = \frac{s_i^j(g)}{\max_{k \in J} \bar{s}_k}, \tag{6}$$

where $\bar{s}_k$ is the average relative score of algorithm k, given by

$$\bar{s}_k = \frac{1}{|G||J|} \sum_{g \in G} \sum_{j \in J} s_k^j(g). \tag{7}$$

The normalized relative score is useful for visualization purposes since the top-ranked algorithm in the set J will have an average normalized relative score of 1, while an average algorithm will have a average normalized relative score of 0 and a low-performing algorithms will have a negative average normalized relative score.

Select the Best The *% best score* is the proportion of partner algorithms (from the set J) against which an algorithm has the highest average per-round payoff across all games. This metric is based on the answer to the question: which algorithm $j \in J$ would you choose if you knew the identify of the partner algorithm? Formally, let

$$best(i, j, G) \leftarrow \begin{cases} 1 & \text{if } rank_{RRA}(i, \{j\}, G) = 1 \\ 0 & \text{otherwise} \end{cases} \tag{8}$$

Then, the *% best score* of algorithm i with respect to J and G is given by

$$best\_score_i(J, G) = \frac{1}{|J|} \sum_{j \in J} best(i, j, G). \tag{9}$$

Algorithms are ranked by this metric based on these best scores. Ties are broken by the number of rank-2 finishes, rank-3 finishes, etc.

Worst-Case Score An algorithms *worst-case score* is its average relative score when paired with the partner algorithm $j \in J$ against which it has the lowest average relative score. This metric answers the question, if you do not know the partner algorithm's identity, which algorithm should you choose in order to minimize your lowest possible average relative score[4]? Formally, let the average relative score of algorithm i when paired with algorithm j across the games in G be

$$\bar{s}_i^j = \frac{1}{|G|} \sum_{g \in G} s_i^j(g). \tag{10}$$

[4]In theory, algorithms can guarantee a payoff no better than their maximin value against an omnipotent adversary (i.e., an agent that seeks only to lower the other player's payoffs). Algorithms that are guaranteed to not perform significantly worse than their maximin value (in expectation) are known to be *secure* [8, 12, 13, 1]. While we find this to be a useful metric, many algorithms exist that have this property, including many of the algorithms chosen for inclusion in our study. Furthermore, we value a more practical metric. Thus, in this work, we measure worst-case performance when paired with represented algorithms chosen for inclusion in our study rather than when paired against omnipotent opponents.

Then, the *Worst-Case Score* of algorithm i with respect to the set J is

$$worst_i(J) = \min_{j \in J} \bar{s}_i^j. \quad 11$$

Algorithms are ranked based on these worst-case scores. The top-ranked algorithm is $\arg\max_{i \in J} worst_i(J)$.

Usage Rate – Replicator Dynamic Another metrics of success is how often an algorithm would be used over a series of generations (we use 10,000 generations) when the population is free to choose the algorithm they desire. Evolutionary dynamics, such as the replicator dynamic [68], provide a way to model population dynamics, from which we can predict the usage rates of the algorithms.

The replicator dynamic proceeds as follows. Let x_i^t be the proportion of the population that uses algorithm i in generation t, such that $(x_1^t, ..., x_{|J|}^t)$ describes the distribution of algorithms used in the population, subject to the constraint that $\sum_{i \in J} x_i^t = 1$. In this work, the population is initially equally divided among the algorithms in J, such that $x_i^0 = \frac{1}{|J|}$. Then, the fitness of algorithm i in generation t is given by

$$f_i^t = \sum_{j \in J} x_j^t RRA_i(G, \{j\}). \quad 12$$

Finally, the proportion of the population using algorithm i in generation $t+1$ is given by:

$$x_i^{t+1} = x_i^t + x_i^t \left(f_i^t - \phi_t \right), \quad 13$$

where ϕ_t is the average fitness of the population in generation t, given by $\phi_t = \sum_{j=J} x_j^t f_j^t$.

Thus, algorithm i's usage rate $U(i)$ over 10,000 generations is given by

$$U_i = \frac{1}{10,000} \sum_{t=1}^{10,000} x_i^t, \quad 14$$

where x_i^t is the proportion of the population using algorithm i in generation t. The algorithm with the highest usage rate (i.e., $\arg\max_i U(i)$) is ranked highest with respect to this metric. To fully rank all algorithms, we iteratively removed the winner and re-evolve the population (using the replicator dynamic).

Elimination Tournaments The final two metrics, which are motivated by Rapoport et al. [69], are elimination tournaments. In these tournaments, algorithms are eliminated via a series of round-robin tournaments. In the first tournament, which we refer to as the *Group-1 Tournament*, the algorithms in J are randomly divided into equally-sized groups (in our case, four groups of size 6, 6, 6, and 7). A round-robin tournament is conducted in each group. The winner from each group advances to the championship round, which consists of another round-robin tournament. Algorithms are ranked by the percentage of trials (out of 10,000) that they win the championship round, with ties broken by the percentage of trials the algorithms advance to the championship round.

In the second elimination tournament, the algorithms in J are, again, randomly divided into equally-sized groups (in our case, four groups of size 6, 6, 6, and 7). A round-robin tournament is conducted in each group. The top two performers in each group advance to the semi-finals, which consists of another round-robin tournament. The top two performers in the semi-final tournament are then paired in a two-player round-robin tournament, the winner of which is crowned the champion. Algorithms are ranked by the percentage of trials (out of 10,000) that they win the championship round, with ties broken by the percentage of trials the algorithms advance to the championship round and then the semi-final round.

Algorithms

In attempt to find an algorithm that meets the previously stated objectives, we compared the performance of the 25 algorithms listed in Supplementary Table 6 using the metrics presented in the previous subsection. While certainly not inclusive of all algorithms created for repeated games, these algorithms represent many different genres of algorithms from various academic disciplines, including computer science, economics, evolutionary biology, and other social sciences. Since many of these algorithms have also been shown to have high performance characteristics in previous empirical evaluations [12, 62, 13, 1], this evaluation helps us obtain a good understanding of the current state-of-the-art for playing repeated normal-form games.

We categorize the algorithms in Supplementary Table 6 into four broad families of algorithms: static algorithms, reinforcement-learning algorithms, expert algorithms, and belief-based/other algorithms. The table provides a color coding to specify this categorization. The static algorithms included in our study were RANDOM, BULLY, GODFATHER, WSLS, MEM-1, and MEM-2. These algorithms have been commonly studied in evolutionary biology and the social sciences in prisoner's dilemmas. While these algorithms have their differences, they all share an important characteristic: they do not change their strategies over the course of an interaction (though they do, with the exception of RANDOM, condition their behavior on the results of previous rounds).

The next six algorithms listed in Supplementary Table 6 all use reinforcement learning [70]. These algorithms are Q-LEARNING, WOLF-PHC, CJAL, MBRL-1, MBRL-2, and M-QUBED. MBRL-1 and MBRL-2 use model-based reinforcement learning while the other four algorithms use model-free reinforcement learning. With the exception of WOLF-PHC, each of these algorithms uses ε-greedy exploration (based on current Q-estimates) to select actions.

Expert algorithms are represented by the next eight algorithms listed in Supplementary Table 6. Each of these algorithms attempts to learn from experience which expert (i.e., strategy or algorithm), from among a particular set of pre-selected strategies or algorithms, produces the highest payoff in the given scenario. Thus, these algorithms differ from each in other with regards to (a) the set of experts they consider and (b) the method for selecting which expert to follow (which we refer to as the *expert-selection mechanism*).

We group the last four algorithms, consisting of belief-based algorithms and algorithms that otherwise use a combination of methods into a final family called *other algorithms*. Belief-based algorithms are represented in our evaluation by FICTITIOUS PLAY and STOCHASTIC FICTITIOUS PLAY. These algorithms use their past experiences to formulate beliefs about their partner's future behavior. The algorithms then compute and play a best-response strategy under the assumption that the algorithm's beliefs are correct. Finally, the last two algorithms, MANIPULATOR and MANIPULATOR-GODFATHER, combine several different genres of algorithms. Both of these algorithms initially compute and play static strategies designed to force a rational associate toward a particular solution. However, if these strategies are unsuccessful in teaching their partner the desired behavior, both algorithms switch to model-based reinforcement learning. If still unsuccessful, these algorithms eventually switch to playing their maximin strategies.

In selecting parameter values for these algorithms, we sought to remain consistent with the settings used in the published literature, while also seeking to optimize each algorithm's performance. However, it was not possible for us to test all combinations of parameter values for all algorithms. In any event, this comparison is not intended to identify an absolute *best* algorithm for repeated games, but rather to find a robust and effective algorithm that meets the previously stated criteria when associating with other algorithms.

Supplementary Table 6: Representative algorithms and implementation details.

Algorithm	Implementation Details
RANDOM	Randomly chooses an action from a uniform distribution in each round.
BULLY	By Littman and Stone [52, 53]. The algorithm is similar to a zero-determinant strategy [41] (but discovered a decade earlier). The version of the algorithm we use enforces (by punishment) the target solution that can produce the highest payoff to the player when the associate maximizes its own payoffs given that it (1) remembers only the last joint action played and (2) plays only pure strategies.
GODFATHER (GTFT)	By Littman and Stone [52, 53]. This algorithm is essentially a generalized generous tit-for-tat strategy strategy. The algorithm is equivalent to BULLY with a different target solution. GODFATHER seeks to enforce the target solution that maximizes the product of the players' advantages.
WSLS	Win-stay-lose-shift, or Pavlov [40]. In our implementation, the algorithm repeats the action played in the previous round if the previous round's payoff was greater than or equal to the player's expected payoff if all solutions were equally likely. Otherwise, the algorithm plays the opposite action from the one it played in the previous round. It randomly selects an action in the first round.
MEM-1	Evolutionarily evolved stochastic memory-1 strategies. A separate strategy was evolved over 500,000 generations for each game using the technique described by Iliopoulous [42]. We used a 32 × 32 grid, with a replacement rate of 0.01 and a mutation rate of 0.005.
MEM-2	Evolutionarily evolved stochastic memory-2 strategies. A separate strategy was evolved over 1,000,000 generations for each game using the technique described by Iliopoulous [42]. We used a 32 × 32 grid, with a replacement rate of 0.01 and a mutation rate of 0.005.
Q-LEARNING (QL)	A model-free reinforcement learning algorithm proposed by Watkins [71]. Our implementation encodes state as the previous joint action and uses ε-greedy exploration. Q-values were initialized randomly in the interval $[0, \frac{1}{1-\gamma}]$. $\varepsilon = \frac{1}{10+t/10}$, $\gamma = 0.95$, $\alpha = \frac{1}{10+t/100}$.
WOLF-PHC	By Bowling and Veloso [11]. A reinforcement learning algorithm that uses policy-hill climbing and a variable learning rate. We used $\alpha = \frac{1}{10+t/100}$, $\varepsilon = \frac{1}{10+t/100}$, $\delta_l = \frac{2}{100+t/100}$, $\delta_w = \frac{1}{100+t/100}$.
CJAL	By Banerjee and Sen [72]. A conditional joint-action learner. $N = 400$ and $\epsilon = 0$.
MBRL-1	A model-based reinforcement learning algorithm that encodes its state as the previous joint action. The algorithm estimates the associate's behavior using the fictitious-play assessment conditioned on the current state, and then computes a best response using value iteration (discount factor $\gamma = 0.95$). It uses ε-greedy exploration, $\varepsilon = \frac{1}{10+t/10}$, and $\kappa^0(s,a) = 1$ for all $s \in S$ and $a \in A_i(s)$.
MBRL-2	Same as MBRL-1, except that it encodes state as the previous two joint actions.

Continued on the next page

Supplementary Table 6 (continued) Representative algorithms and implementation details.

Algorithm	Implementation Details
M-QUBED	By Crandall and Goodrich [13]. A model-free reinforcement learning algorithm. Parameters set as specified in Table 7 of the cited paper.
GIGA-WOLF	By Bowling [15]. A no-regret algorithm. We used $\eta_i = \frac{1}{\sqrt{t}}$.
WMA	Weighted Majority Algorithm, by Freund and Shapire [73]. We used $\beta_i = 0.999$.
S++	By Crandall [1]. Our implementation was identical to that of the published work.
S++/SIMPLE	Identical to S++ except that the algorithm selects from a simpler set of experts. This simplified set of experts consists of each of the pure strategies, the maximin strategy, and a strategy that alternates between the two actions.
EXP3	A no-regret algorithm by Auer et al. [44]. In our implementation, the algorithm selects from the same set of experts as S++. We used $\eta_i = \frac{\lambda}{\lvert A_i \rvert}$, where $\lvert A_i \rvert$ is the number of actions for player i, and $g_i = 50000$.
EXP3/SIMPLE	Identical to Exp3 except that the algorithm selects from a simpler set of experts. This simplified set of experts consists of each of the pure strategies, the maximin strategy, and a strategy that alternates between the two actions.
EEE	By de Farias and Meggido [17]. An ε-greedy expert algorithm. In our implementation, the algorithm selects from the same set of experts as S++. We used $\omega = \lvert A_i \rvert \lvert A_{-i} \rvert$ and $\varepsilon = \frac{1}{10 + t/10}$.
EEE/SIMPLE	Identical to EEE except that the algorithm selects from a simpler set of experts. This simplified set of experts consists of each of the pure strategies, the maximin strategy, and a strategy that alternates between the two actions.
S	Derived from Karandikar et al. [47]. Consistent with the work of Karandikar, the implementation we use [1] is identical to S++ except it does not prune the set of selectable experts or implement best-response and security overrides (as is done with S++). We used $\lambda = 0.99$.
FICTITIOUS PLAY (FP)	Originally proposed by Brown [37]. See also Fudenberg and Levine [8]. Fictitious play is a special case of "rational" learning with a Dirichlet-distributed prior [8]. We used $\kappa^0(a) = 0$ for all a. Typically studied for the computation of Nash equilibria, it is still interesting as an online-learning algorithm.
STOCHASTIC FICTITIOUS PLAY (sFP)	See Fudenberg and Levine [8]. A variant of Fictitious Play that can learn to play mixed strategies. We used $\kappa^0(a) = 0$ for all a, along with Boltzmann exploration with temperature parameter set to $\tau = \frac{100}{t}$.
MANIPULATOR	By Powers and Shoham [12]. This algorithm serially follows three different algorithms. In our implementation, it follows BULLY for the first 150 rounds. Thereafter, if its payoffs become lower than expected, it switches to MBRL-1. After 300 rounds, if its average payoff ever drops substantially below its maximin value (i.e., $v_i^{\mathrm{mm}} - \varepsilon$), it plays its maximin strategy thereafter.
MANIPULATOR-GODFATHER	By Powers and Shoham [12]. Identical to MANIPULATOR, except that it follows GODFATHER instead of BULLY over the first 150 rounds.

Games

We compared the 25 selected algorithms across the set of games described in Supplementary Note 2 (see the subsection entitled *Evaluation Methods*). This set of 720 2x2 games forms a compelling test-set against which none of the selected algorithms has previously been evaluated. Furthermore, we are not aware of any other study in which algorithms have been evaluated against such a comprehensive set of games and associates.

Outcomes

An overview of the performance comparison of the 25 algorithms selected for our study with respect to the six metrics (at each game length) is given in Supplementary Table 7. The average payoffs received by each algorithm in each pairing at each game length and the scores of each algorithm with respect to each metric are provided at the end of this Supplementary Note in Supplementary Tables 12–17. In addition, Supplementary Tables 8 and 9 show the rankings of the algorithms with respect to the round-robin averages when restricted to interactions with specific partner families and classes of games, respectively. Supplementary Figure 5 gives a visual comparison of the relative performance of selected algorithms given the different payoff families, game lengths, and partner families. Finally, Supplementary Figure 6 shows the evolution of the population when subject to the replicator dynamic for each game length.

We first discuss high-level observations about these collective results. We then further illuminate these observations by discussing the performance profiles of several of the algorithms.

General Observations Several interesting high-level observations can be made from Supplementary Tables 7–9 and Supplementary Figure 5, which we discuss as a series of bullet points.

1. It is interesting to observe which algorithms did not perform especially well in this evaluation. These algorithms include:

    - *Static algorithms that have been shown to be robust algorithms for prisoner's dilemmas*. These algorithms include generalized (generous) tit-for-tat (i.e., GODFATHER) [74], WSLS [40], and evolutionarily evolved memory-one and memory-two stochastic strategies (e.g., MEM-1 and MEM-2). While some of these algorithms are somewhat effective in shorter interactions in some scenarios, they have relatively poor performance in longer interactions. One important drawback of these algorithms is that they do not adapt to their partner's behaviors. Thus, they are not partner independent. The results also show that these algorithms are better suited in games belonging to some payoff families than those belonging to others, thus demonstrating a lack of game independence.
    - *Algorithms designed to minimize regret*. These algorithms include GIGA-WoLF, WMA, and EXP3. Regret minimization is a central component of world-champion computer poker algorithms [3]. Though regret minimization appears to be effective in zero-sum games, it is not guaranteed to correlate positively with payoff maximization in general-sum games. Indeed, previous work has shown that regret minimization often does not correlate with payoff maximization when associates are adaptive [17, 18, 65, 1]. In fact, these algorithms were among the weaker algorithms in our evaluations across the six performance metrics.

Algorithm	Round-Robin Average	% Best Score	Worst-Case Score	Replicator Dynamic	Group-1 Tourney	Group-2 Tourney	Rank Summary Best – Mean – Worst
S++	1, 1, 1	2, 1, 2	1, 1, 1	1, 1, 1	1, 1, 2	1, 1, 1	1 – 1.2 – 2
MANIPULATOR	3, 2, 3	4, 3, 8	5, 2, 4	6, 4, 3	5, 3, 3	5, 2, 2	2 – 3.7 – 8
BULLY	3, 2, 1	3, 2, 1	7, 13, 20	7, 3, 2	6, 2, 1	6, 3, 5	1 – 4.8 – 20
S++/SIMPLE	5, 4, 4	8, 5, 9	4, 6, 10	10, 2, 6	8, 4, 6	9, 4, 6	2 – 6.1 – 10
S	5, 5, 8	6, 7, 10	3, 3, 8	5, 5, 8	7, 5, 9	7, 5, 9	3 – 6.4 – 10
FICT. PLAY	2, 8, 14	1, 6, 10	2, 8, 16	3, 12, 15	2, 8, 12	4, 9, 14	1 – 8.1 – 16
MBRL-1	6, 6, 10	5, 4, 7	8, 7, 14	11, 11, 13	9, 7, 10	8, 7, 10	4 – 8.5 – 14
EEE	11, 8, 7	14, 9, 5	9, 4, 2	14, 10, 9	13, 9, 8	13, 10, 8	2 – 9.1 – 14
MBRL-2	14, 5, 5	13, 8, 6	19, 5, 3	18, 9, 4	18, 6, 5	18, 6, 4	3 – 9.2 – 19
MEM-1	6, 9, 13	7, 10, 21	6, 9, 17	2, 6, 10	3, 10, 17	2, 8, 15	2 – 9.5 – 21
M-QUBED	14, 20, 4	15, 20, 3	15, 19, 5	17, 19, 5	17, 21, 4	16, 21, 3	3 – 13.2 – 21
MEM-2	9, 11, 20	9, 11, 22	13, 17, 22	4, 13, 19	4, 13, 25	3, 12, 20	3 – 13.7 – 25
MANIP-GF	11, 11, 21	12, 12, 19	12, 11, 19	9, 7, 20	12, 14, 20	11, 13, 21	7 – 14.2 – 21
WOLF-PHC	17, 11, 13	18, 14, 14	18, 14, 18	16, 14, 14	16, 11, 11	15, 11, 11	11 – 14.2 – 18
QL	17, 17, 7	19, 19, 4	17, 18, 7	19, 18, 7	19, 20, 7	19, 18, 7	4 – 14.4 – 20
GF (GTFT)	11, 14, 22	11, 15, 20	11, 16, 23	8, 8, 22	10, 16, 21	10, 15, 22	8 – 15.3 – 23
EEE/SIMPLE	20, 15, 11	20, 17, 12	20, 10, 9	20, 16, 11	24, 15, 14	20, 16, 13	9 – 15.7 – 24
EXP3	19, 23, 11	16, 23, 15	16, 23, 6	15, 23, 12	15, 25, 13	17, 25, 12	6 – 17.2 – 25
CJAL	24, 14, 14	25, 14, 13	24, 12, 15	24, 17, 16	20, 12, 16	22, 14, 16	12 – 17.3 – 25
WSLS	9, 17, 24	10, 16, 24	10, 20, 24	12, 20, 24	11, 17, 24	12, 17, 25	9 – 17.6 – 25
GIGA-WOLF	14, 19, 23	17, 18, 23	14, 15, 21	13, 15, 23	14, 18, 22	14, 19, 23	13 – 18.1 – 23
WMA	21, 21, 15	21, 21, 16	22, 21, 12	22, 21, 17	21, 19, 15	23, 20, 17	12 – 19.2 – 23
STOCH. FP	21, 21, 15	22, 22, 17	23, 22, 11	23, 22, 18	25, 24, 18	25, 22, 18	11 – 20.5 – 25
EXP3/SIMPLE	21, 24, 16	23, 24, 18	21, 24, 13	21, 24, 21	22, 22, 19	21, 23, 19	13 – 20.9 – 24
RANDOM	24, 25, 25	24, 25, 25	25, 25, 25	25, 25, 25	23, 23, 23	24, 24, 24	23 – 24.4 – 25

Supplementary Table 7: We compared and ranked 25 algorithms with respect to the six performance metrics described in Supplementary Table 5 across the periodic table of 2x2 games (Supplementary Figure 1). A lower rank indicates higher performance. For each metric, the algorithms are ranked at three different game lengths: 100-round, 1000-round, and 50,000-round games, respectively. For example, the 3-tuple $(3, 2, 1)$ means the algorithm was ranked 3rd in 100-round games, 2nd in 1000-round games, and 1st in 50,000-round games.

Algorithm	Rank by Partner Family: vs. Static Algs	vs. RL Algs	vs. Expert Algs	vs. Other Algs	Rank Summary Best – Mean – Worst
S++	1, 1, 1	3, 2, 2	1, 2, 2	1, 1, 1	1 – 1.5 – 3
MANIPULATOR	6, 6, 10	1, 1, 2	3, 2, 2	5, 3, 3	1 – 3.7 – 10
S++/SIMPLE	4, 3, 7	4, 4, 4	5, 4, 4	4, 3, 3	3 – 4.1 – 7
BULLY	7, 11, 19	1, 1, 1	3, 1, 1	5, 1, 1	1 – 4.3 – 19
S	3, 2, 7	9, 5, 9	5, 5, 6	4, 3, 3	2 – 5.1 – 9
MBRL-1	3, 1, 1	10, 8, 9	5, 7, 11	1, 3, 4	1 – 5.2 – 11
MBRL-2	11, 2, 1	18, 5, 4	14, 5, 6	9, 4, 3	1 – 6.8 – 18
FICT. PLAY	1, 7, 12	4, 5, 14	1, 7, 11	1, 7, 12	1 – 6.8 – 14
EEE	11, 6, 1	10, 5, 4	11, 10, 9	9, 8, 5	1 – 7.4 – 11
MEM-1	3, 9, 18	6, 5, 11	5, 7, 9	4, 8, 11	3 – 8.0 – 18
QL	16, 10, 1	18, 19, 8	17, 16, 6	15, 12, 5	1 – 11.9 – 19
MEM-2	6, 9, 18	13, 13, 17	9, 11, 11	9, 11, 18	6 – 12.1 – 18
M-QUBED	11, 17, 1	17, 19, 6	14, 19, 4	15, 20, 4	1 – 12.2 – 20
MANIP-GF	12, 13, 19	6, 11, 17	11, 11, 18	9, 11, 20	6 – 13.2 – 20
WOLF-PHC	18, 10, 12	15, 12, 14	17, 14, 11	18, 11, 11	10 – 13.6 – 18
GF (GTFT)	12, 19, 23	6, 11, 16	11, 11, 11	9, 14, 22	6 – 13.8 – 23
EEE/SIMPLE	20, 10, 10	18, 14, 9	20, 16, 11	20, 14, 11	9 – 14.4 – 20
WSLS	8, 12, 19	10, 17, 24	9, 16, 24	8, 16, 22	8 – 15.4 – 24
EXP3	18, 23, 7	15, 23, 9	19, 23, 11	19, 23, 11	7 – 16.8 – 23
CJAL	24, 9, 11	24, 14, 14	24, 15, 20	24, 11, 11	9 – 16.8 – 24
GIGA-WOLF	16, 19, 23	14, 15, 21	14, 17, 23	15, 18, 22	14 – 18.1 – 23
STOCH. FP	21, 19, 11	22, 19, 16	21, 21, 11	21, 20, 16	11 – 18.2 – 22
WMA	21, 19, 11	22, 20, 16	21, 21, 11	21, 20, 16	11 – 18.2 – 22
EXP3/SIMPLE	21, 24, 12	21, 24, 14	21, 24, 20	23, 24, 17	12 – 20.4 – 24
RANDOM	24, 25, 25	24, 25, 25	24, 25, 25	24, 25, 25	24 – 24.7 – 25

Supplementary Table 8: Algorithm rank based on the round-robin average when restricted to interactions with individual partner families (see Supplementary Table 6). Lower rank indicates higher performance, and is determined by the number of algorithms that had statistically higher payoffs than that algorithm (Supplementary Equation 3). For each category, the algorithms are ranked at three different game lengths: 100-round, 1000-round, and 50,000-round games, respectively. For example, the 3-tuple $(1, 2, 3)$ means the algorithm was ranked 1st in 100-round games, 2nd in 1000-round games, and 3rd in 50,000-round games.

Algorithm	Rank by Cooperative Family			Rank by Payoff Family						Rank Summary
	Simple Games	Temptation Games	Dominated Games	Win-Win 4, 4	Biased 4, 3	Second Best 3, 3	Unfair 4, 2	Inferior	Cyclic	Best – Mean – Worst
S++	1, 1, 1	1, 1, 1	1, 1, 2	1, 2, 1	2, 1, 1	1, 1, 1	1, 1, 2	1, 1, 2	1, 1, 2	1 – 1.2 – 2
BULLY	1, 1, 1	3, 1, 1	5, 4, 5	1, 1, 1	4, 3, 1	6, 9, 15	1, 1, 1	2, 1, 1	9, 6, 8	1 – 3.4 – 15
MANIPULATOR	1, 1, 1	3, 1, 1	5, 4, 10	1, 1, 1	4, 2, 1	6, 5, 2	1, 1, 2	2, 2, 7	9, 8, 11	1 – 3.4 – 11
S	8, 6, 1	7, 7, 18	3, 1, 2	9, 8, 6	6, 8, 18	3, 1, 1	7, 8, 18	2, 2, 3	2, 1, 2	1 – 5.8 – 18
S++/SIMPLE	7, 6, 1	3, 4, 4	19, 6, 6	1, 6, 6	3, 3, 4	11, 5, 2	5, 4, 4	11, 8, 8	23, 4, 6	1 – 6.3 – 23
MBRL-1	11, 6, 1	6, 6, 5	2, 8, 12	12, 9, 6	6, 6, 6	6, 2, 2	5, 5, 7	2, 8, 12	2, 8, 13	1 – 6.4 – 13
MBRL-2	16, 10, 1	12, 7, 5	3, 2, 3	15, 11, 4	12, 8, 6	16, 2, 1	11, 5, 4	11, 3, 7	3, 1, 2	1 – 6.7 – 16
EEE	14, 12, 5	9, 6, 4	8, 6, 9	15, 16, 14	10, 7, 5	4, 2, 2	8, 6, 4	10, 8, 11	9, 4, 6	2 – 7.9 – 16
FICT. PLAY	1, 3, 8	1, 4, 4	8, 22, 24	1, 6, 14	1, 3, 4	1, 5, 14	1, 5, 7	5, 15, 19	15, 21, 23	1 – 8.7 – 24
MANIP-GF	1, 1, 1	18, 17, 23	4, 4, 6	1, 1, 1	18, 17, 23	11, 16, 22	12, 15, 22	2, 2, 3	4, 4, 8	1 – 9.5 – 23
MEM-1	7, 6, 5	7, 8, 14	5, 11, 14	8, 6, 7	6, 8, 13	8, 10, 19	6, 9, 18	3, 8, 12	9, 13, 17	3 – 9.5 – 19
GF (GTFT)	1, 1, 1	18, 23, 24	4, 4, 5	1, 1, 1	18, 23, 24	11, 17, 22	13, 15, 22	2, 2, 1	5, 4, 8	1 – 10.0 – 24
M-QUBED	14, 15, 1	15, 20, 6	8, 11, 1	12, 12, 3	14, 19, 8	18, 22, 2	15, 22, 4	17, 21, 2	5, 9, 1	1 – 11.0 – 22
QL	17, 15, 1	15, 16, 11	7, 11, 2	16, 15, 4	16, 16, 16	16, 21, 1	15, 17, 8	17, 17, 4	5, 8, 2	1 – 11.4 – 21
MEM-2	10, 12, 12	11, 14, 20	8, 11, 14	11, 10, 7	11, 14, 20	11, 16, 21	8, 12, 19	10, 12, 12	9, 13, 16	7 – 12.7 – 21
WOLF-PHC	18, 15, 17	12, 9, 5	10, 15, 17	18, 17, 20	13, 11, 6	15, 10, 5	11, 9, 9	12, 15, 19	12, 14, 15	5 – 12.9 – 20
EXP3	19, 23, 12	12, 19, 7	16, 14, 12	19, 23, 18	12, 20, 8	6, 11, 4	14, 22, 9	12, 13, 12	14, 13, 11	4 – 13.9 – 23
EEE/SIMPLE	20, 20, 18	15, 11, 5	20, 14, 13	19, 20, 21	16, 13, 7	18, 15, 5	11, 7, 4	17, 12, 14	23, 16, 13	4 – 14.3 – 23
WSLS	10, 15, 24	7, 15, 21	15, 17, 20	12, 17, 24	6, 15, 21	2, 6, 18	11, 16, 24	11, 12, 13	18, 21, 23	2 – 15.3 – 24
GIGA-WOLF	10, 13, 22	18, 19, 21	16, 15, 15	9, 9, 15	15, 17, 21	18, 22, 24	15, 15, 20	17, 19, 19	9, 14, 15	9 – 16.4 – 24
CJAL	24, 15, 14	24, 10, 5	23, 20, 20	24, 17, 18	24, 11, 6	24, 10, 9	24, 8, 5	24, 13, 18	20, 21, 20	5 – 16.7 – 24
STOCH. FP	21, 21, 10	22, 16, 6	20, 20, 20	21, 21, 14	22, 17, 8	22, 18, 3	22, 17, 7	21, 19, 19	18, 19, 20	3 – 17.2 – 22
WMA	22, 21, 10	22, 16, 6	20, 19, 20	21, 21, 14	22, 17, 8	22, 18, 5	21, 18, 7	21, 19, 19	18, 19, 20	5 – 17.3 – 22
EXP3/SIMPLE	21, 24, 20	21, 24, 9	23, 24, 18	22, 24, 22	21, 24, 13	21, 24, 16	21, 24, 7	21, 24, 18	23, 22, 18	7 – 20.3 – 24
RANDOM	24, 25, 25	24, 25, 25	23, 24, 25	24, 25, 25	24, 25, 25	24, 25, 25	24, 25, 25	24, 25, 25	20, 21, 23	20 – 24.2 – 25

Supplementary Table 9: Algorithm rank based on the round-robin average when restricted to games belonging to the individual cooperative families (Supplementary Figure 2) and the individual payoff families [31] (see Supplementary Figure 1). Lower rank indicates higher performance, and is determined by the number of algorithms that had statistically higher payoffs than that algorithm (Supplementary Equation 3). For each category, the algorithms are ranked at three different game lengths: 100-round, 1000-round, and 50,000-round games, respectively. For example, the 3-tuple $(1, 2, 3)$ means the algorithm was ranked 1st in 100-round games, 2nd in 1000-round games, and 3rd in 50,000-round games.

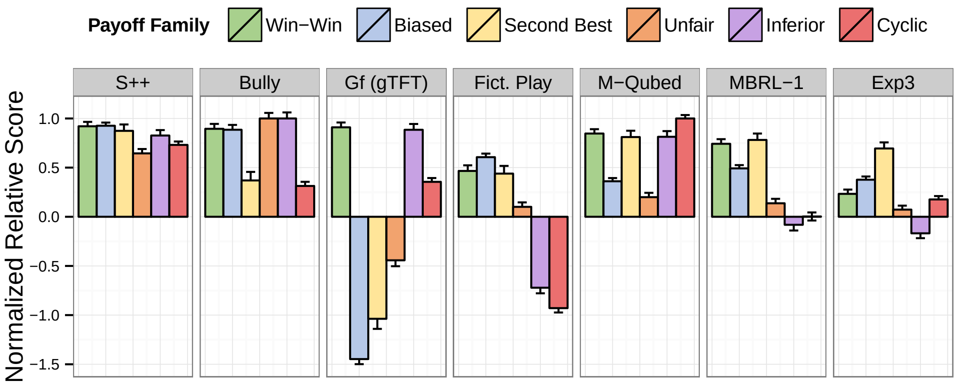

(a) Performance in games specified by the various payoff families in 50,000-round games.

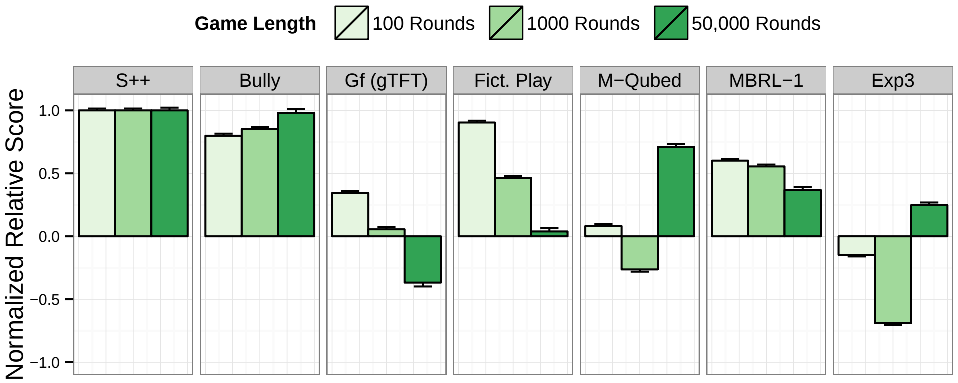

(b) Performance based on game length across all games and partners.

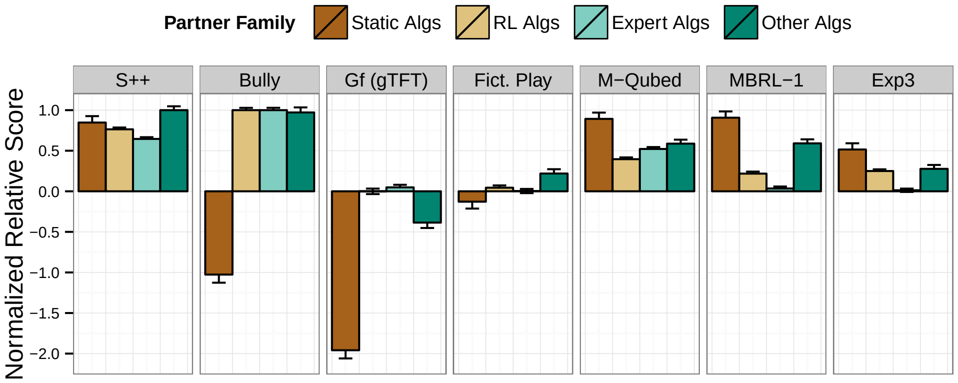

(c) Performance when paired with each partner family in 50,000-rounds games.

Supplementary Figure 5: The average *normalized relative score* (Supplementary Equation 6) of seven selected algorithms in interactions categorized by (a) payoff family (50,000-round interactions only), (b) game length, and (c) partner family (50,000-round interactions only). A normalized relative score of 0 indicates average performance, a normalized relative score of 1 indicates the highest relative performance, and a normalized relative score less than 0 indicates performance that is less than average (among the 25 selected algorithms). Error bars show the standard error of the mean.

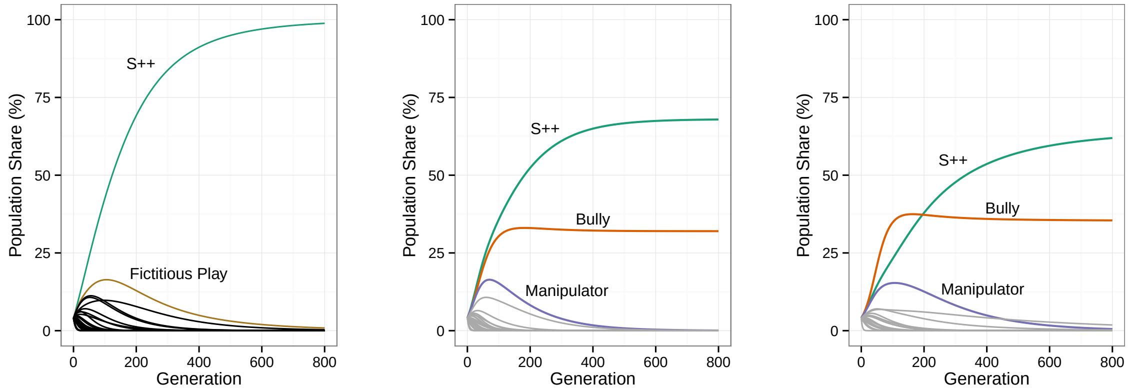

(a) 100-round interactions (b) 1000-round interactions (c) 50,000-round interactions

Supplementary Figure 6: Evolutionary dynamics based on the replicator dynamic.

2. It is also interesting to observe which algorithms performed on average. We focus on the algorithms that ranked in the top five across our evaluations. These algorithms can be categorized into two groups:

   - *Algorithms whose principle strategy is to try to bully their associate*. Averaged across all metrics we considered, MANIPULATOR and BULLY ranked second and third, respectively (Supplementary Table 7). Both of these algorithms initially compute and then play a strategy that enforces the solution that gives it the highest payoff subject to its partner's payoff also exceeding its maximin value. BULLY perpetually seeks to play this (zero-determinant [41]) strategy, while MANIPULATOR only continues to play this strategy if doing so produces high payoffs. As a result, these algorithms tend to be able to exploit adaptive partners that eventually learn to play a best response. However, the bully strategy often performs poorly when its partner does not learn.
   - *Algorithms that utilize various forms of aspiration learning* [75, 47]. Across all game lengths and all metrics, the algorithms that ranked first (S++), fourth (S++/SIMPLE), and fifth (S) were expert algorithms that used various forms of aspiration learning. The top-rated algorithm, S++ [1], was consistently one of the top performers across all metrics (Supplementary Table 7). It also interacted effectively when paired with all partner families (Supplementary Table 8) and in games belonging to each payoff and cooperative family (Supplementary Table 9), thus displaying both partner and game independence. Furthermore, it maintained its superior performance in both shorter and longer interactions, thus demonstrating fast and sustainable learning. Due to the superiority and robustness of S++, we discuss it at greater length later in this Supplementary Note and, indeed, the remainder of the paper.

To better understand these observations and other trends, we discuss the performance profiles of a number of algorithms in more detail. This discussion is intended to highlight the strengths and weaknesses of various algorithms and algorithmic families with respect to partner independence, game independence, and fast and sustainable behavior.

Performance Profiles of Several Selected Algorithms The algorithms we selected to discuss in more detail are GODFATHER (i.e., GF (GTFT), an algorithm that computes and plays a generalized, generous tit-for-tare strategy), FICTITIOUS PLAY, EXP3, MBRL-1, M-QUBED, BULLY, and S++.

Godfather (gTFT): One of the more surprising results from our study was the relatively low performance of GODFATHER, which implements a form of generalized (generous) tit-for-tat. Tit-for-tat has repeatedly been shown to be a robust strategy in prisoner's dilemmas [74, 40]. The algorithm is very simple: reciprocate both cooperation and defection. In other words, GODFATHER plays its part of the mutually cooperative solution until the other player fails to play its part. Once the partner "defects", GODFATHER punishes its partner by playing its minimax (attack) strategy until its partner's payoffs are less than they would have been had it always played the mutually cooperative solution. Once the punishment is complete, it returns to playing the strategy corresponding to mutual cooperation.

Supplementary Figure 5a shows that, averaged over all pairings, GODFATHER is relatively successful in games belonging to three payoff families: Win-Win, Inferior, and Cyclic. Games in the Inferior (which includes the prisoner's dilemma) and Cyclic payoff families have Pareto-dominated one-shot NE, scenarios for which the tit-for-tat strategy was originally designed. However, GODFATHER is highly unsuccessful in the other three game classes. A primary take-away from this result is that, because of the almost exclusive focus on prisoner's dilemma, a general understanding of what strategic behavior leads to effective long-term relationships has not been identified by this past research (even though that research has been illuminating for specific types of scenarios).

Fictitious Play: Another widely studied algorithm is FICTITIOUS PLAY, a simple belief-based algorithm whose dynamics are now highly understood. Supplementary Table 7 shows that this algorithm is very successful (at least with respect to average, best, and worst-case performance) in shorter interactions (i.e., 100 rounds). However, Supplementary Figure 5b shows that the relatively performance of FICTITIOUS PLAY tails off substantially in longer interactions.

These performance trends are due to the myopic nature of FICTITIOUS PLAY. Due to its simplicity, FICTITIOUS PLAY often quickly learns behavior that maximizes its immediate payoffs, whereas algorithms that seek to establish cooperative relationships sometimes require more time to learn. This allows it to have high relative performance in early rounds of a repeated interaction. However, it is unable to sustain this early (relative) success since such behavior leads to convergence to local maxima in games in which there is no Pareto optimal (pure-strategy) NE. As such, it does not establish cooperative relationships and hence receives relatively poor payoffs in these games (Supplementary Figure 5a).

Exp3: Another algorithmic mechanism that has been studied repeatedly is that of multiplicative weight updates [76]. Both WMA and EXP3 are versions of this general algorithm. In stationary environments and environments with unique equilibrium characteristics, this algorithm asymptotically approaches optimal behavior. However, despite frequent misconceptions to the contrary, such guarantees do hold in general-sum games in which the associate adapts its behavior over time [17, 18, 65, 1]. Our results show that EXP3 learns rather slowly, but it does improve itself over time, such that by 50,000-rounds its average payoffs are better than average across all pairings and all games in our study (Supplementary Figure 5b). However, even in these longer interactions, it is not among the top-performing algorithms in most games and when paired with each partner type.

MBRL-1: Model-based reinforcement learning, with the last one or two previous joint actions defining its state, was a relatively successful algorithm in our evaluations at all game lengths. However, it was not among the top-performing algorithms. Supplementary Figure 5 shows why: MBRL-1 had only average performance in Unfair, Inferior, and Cyclic games, and when associating with other reinforcement learning algorithms and expert algorithms. This last result is perhaps unsurprising, since reinforcement learning of

this form has effective performance guarantees only in stationary settings.

M-Qubed: As is the case with most model-free reinforcement learning algorithms, M-QUBED learns rather slowly. Thus, for the first few thousands of rounds of interaction, its relative performance is typically quite low. However, in 50,000-round interactions, it was one of the top-three performing algorithms in our study averaged across all performance metrics (Supplementary Table 17). At these longer game lengths, M-QUBED has been noted for its ability to balance cooperation, compromise, and security, which allows it to learn effectively against a wide-variety of associates [13]. In fact, in 50,000-round interactions, it was one of the few algorithms to have better-than average performance against each of the four partner families and in games corresponding to each of the six payoff families (Supplementary Figure 5). However, it did not have elite performance in our study due to its poor performance in shorter interactions (Supplementary Tables 15 and 16).

Bully: With respect to most metrics used in our evaluations, BULLY was one of the top-performing algorithms. Averaged across pairings with all algorithms in our study, it had high performance in games corresponding to each payoff family (Supplementary Figure 5a). It was also effective, on average, at all game lengths (Supplementary Figure 5b). Furthermore, it had extraordinary performance when associating with learning algorithms, particularly reinforcement learning algorithms and expert algorithms (Supplementary Figure 5c). However, BULLY performed very poorly when paired with static algorithms (Supplementary Figure 5c), the partner family to which it belongs.

BULLY's excellent performance when associating with partners that adapt makes it an intriguing algorithm. However, its inability to establish effective relationships with itself and other static algorithms is a substantial weakness. In fact, this weakness causes it to fail to obtain a majority population share under evolutionary dynamics at all game lengths (Supplementary Figure 6), though it is able to maintain a sizable minority population share in 1000- and 50,000-round interactions. BULLY's excellent average performance across all associates allows it to perform well in early generations. However, once its population share reaches a certain threshold, it can no longer progress because of its low performance when paired with like-minded associates. Thus, S++ takes the majority proportion of the population.

BULLY's inability to cooperate with itself likely makes it a poor candidate for learning to cooperate with people, especially in light of people's perceived obsession for fairness [77]. Thus, we turn our attention to the top-performing algorithm across our evaluations: S++.

S++: A recently developed expert algorithm [1], S++ had high performance with respect to each of the six metrics we considered at each game length (Supplementary Table 7). Its high performance was due to its ability to substantially outperform the average when paired with each of the other algorithms (Supplementary Figure 5c), in games belonging to each payoff family (Supplementary Figure 5a), and at each game length (Supplementary Figure 5b). As a result, in addition to having high best-, average-, and worst-case performance, it also performed very well in the evolutionary tournaments (Supplementary Figure 6 and Supplementary Table 7). In short, S++ was the only algorithm to substantially outperform the average regardless of payoff family, game length, and partner family.

Several examples illustrate the behavior of S++ and show why it is successful. First, Supplementary Figure 7 demonstrates that S++ quickly learns to cooperate with a copy of itself in the prisoner's dilemma, whereas other machine learning algorithms are either unable to do so, or do so only after hundreds (S) or thousands (M-Qubed) of rounds of interaction. Similar results are observable in many different games.

Second, S++ has the ability to adapt its behavior in order to quickly establish cooperative relationships with itself and other associates. For example, consider Supplementary Figure 8, which shows the kinds of strategies that S++ learns to play against various associates in the game Chicken. S++ is an expert algorithm (see Supplementary Note 4) that selects among a set of expert strategies. These strategies include another

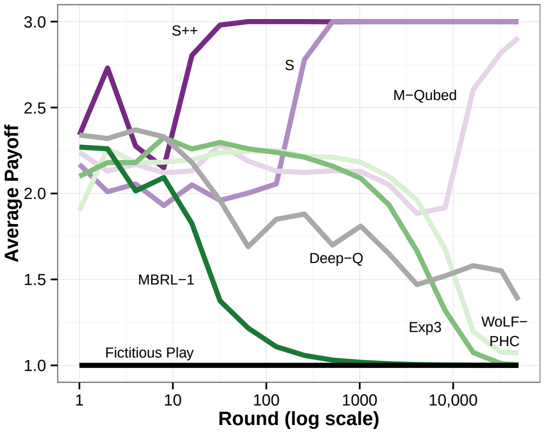

Supplementary Figure 7: Average payoffs over time of eight different machine-learning algorithms in self play in a standard (0-1-3-5)-prisoner's dilemma (Supplementary Table 10a). Results are an average of 50 trials each. Some of the algorithms learn to cooperate (the purple-shaded curves), while others learn to defect (green/grey-shaded curves). Only S++ learns to cooperate within timescales likely to support interactions with people.

(a) (0, 1, 3, 5)-Prisoner's Dilemma

	**cooperate**	**defect**
**cooperate**	3, 3	0, 5
**defect**	5, 0	1, 1

(b) Chicken

	**Swerve**	**Straight**
**Swerve**	84, 84	33, 100
**Straight**	100, 33	0, 0

Supplementary Table 10: Two different normal-form games in which cooperation and self-interest are in conflict.

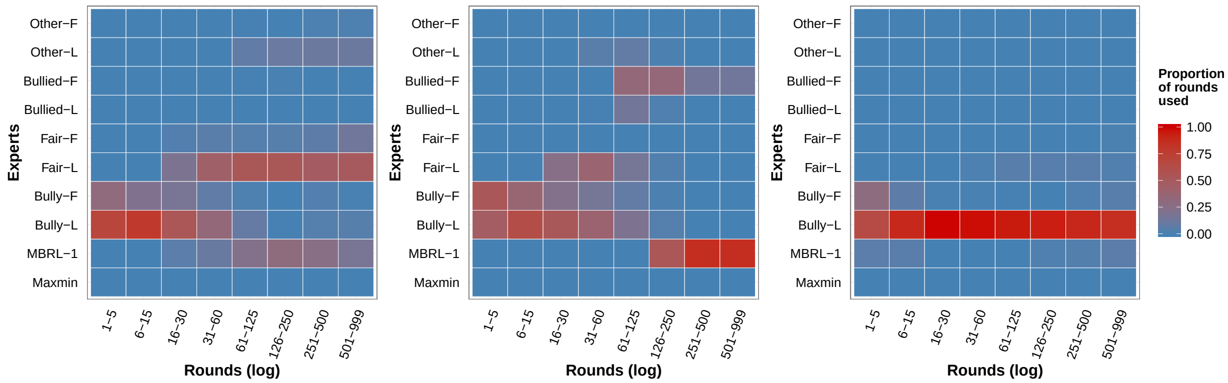

(a) S++ vs. S++ (b) S++ vs. Bully (c) S++ vs. MBRL-2

Supplementary Figure 8: An illustration of the learning dynamics of S++ in Chicken (Supplementary Table 10b). For ease of understanding, experts are categorized into groups (see text). (a) The proportion of time that S++ selects each group of experts over time when paired with a copy of itself. S++ initially seeks to bully its associate, but then switches to fair, cooperative experts when attempts to exploit are unsuccessful. (b) When paired with BULLY, S++ learns the best response, which is to be bullied, achieved by playing MBRL-1, Bully-L, or Bully-F. (c) S++ quickly learns to play experts that bully MBRL-2.

Algorithm	Implementation Details
DEEP-Q	An implementation of the Q-learning algorithm that used an artificial neural network to approximate the underlying Q-values. We used the Encog Java implementation (http://www.heatonresearch.com/encog) with resilient propagation (RPROP) applied as a learning rule [78]. To speedup the learning process, we followed the steps of the Deep-Q-Network [79], and used experience replay [80]. The state was encoded as the previous joint action, one node for each player. Output nodes represented the Q-values, one for each action. For two-player, two-action normal-form games, we used a feedforward network with a 2-4-4-2 architecture (two input nodes, two output nodes, and two hidden layers of 4 nodes each). ε-greedy exploration was used with $\varepsilon = 0.1$ and $\gamma = 0.9$.

Supplementary Table 11: The DEEP-Q algorithm, modeled after DeepMind [79]. We did not include DEEP-Q in the set of 25 algorithms selected for our study since (1) we had other representative reinforcement-learning algorithms and (2) it has a slow runtime, making comparisons across manage games and algorithms very difficult. From what we have observed, it does not appear to have more effective behavior than the other reinforcement learning algorithms we considered in these repeated games.

learning algorithm (MBRL-1), the maximin strategy (Maxmin), and many leader and follower strategies [52] that attempt to establish different kinds of joint solutions. Rather than enumerate all strategies in the figure, we group them into eight categories. Bully-L and Bully-F consist of leader and follower experts, respectively, that advocate Pareto optimal solutions that give S++ a higher payoff than its partner. Fair-L and Fair-F similarly consist of leader and follower experts, respectively, that advocate Pareto optimal solutions that give S++ and it partner the same payoff. Bullied-L and Bullied-F consist of Pareto optimal strategies that give S++'s partner higher payoffs than S++, and Other-F and Other-L consist of the remaining strategies, which advocate Pareto-dominated outcomes.

Supplementary Figure 8 shows that S++ learns widely different strategies when associating with itself, BULLY, and MBRL-2 in the game Chicken (Supplementary Table 10b). In self play, S++ learns to play fair strategies (Supplementary Figure 8a). On the other hand, when paired with BULLY it learns to play a best response (Supplementary Figure 8b) to BULLY's strategy. Alternatively, S++ learns to bully MBRL-2 (Supplementary Figure 8c).

As an additional example showing the ability of S++ to adapt to the current scenario, we consider its behavior when paired with DEEP-Q[5]. DEEP-Q (Supplementary Table 11) defects most of the time in self play in the Prisoner's Dilemma, with occasional short runs of mutual cooperation. This results in relatively low average payoffs that tend toward 1 over time (Supplementary Figure 7). S++ obtains much higher payoffs against DEEP-Q than DEEP-Q does (Supplementary Figure 9a). The reason for this success is that S++ is able to elicit more frequent cooperative behavior from DEEP-Q (giving both players higher payoffs) such that the algorithms eventually engage in mutually cooperation in about half of the rounds (Supplementary Figure 9b). Thus, when paired with DEEP-Q in the Prisoner's Dilemma, S++ is preferable to DEEP-Q due to its ability to establish a cooperative relationship.

In Chicken, S++ is also preferable to DEEP-Q (when paired with DEEP-Q), but for a different reason. In self play, DEEP-Q often learns to cooperate (wherein both players `swerve`) in Chicken, and on average obtains a per-round payoff of approximately 70 (Supplementary Figure 9c). However, when S++ is paired with

[5]Note that DEEP-Q was not among the 25 selected algorithms used in our primary evaluation due to its slow running time.

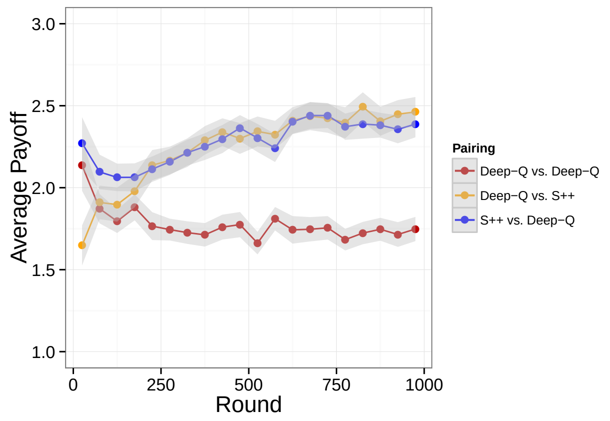

(a) Prisoner's Dilemma – Average Payoffs

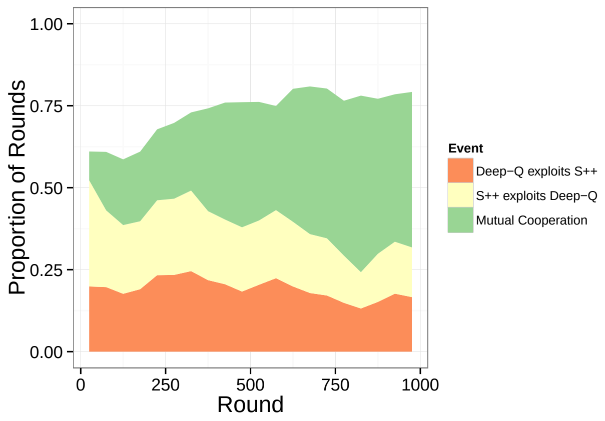

(b) Prisoner's Dilemma – Joint Outcomes

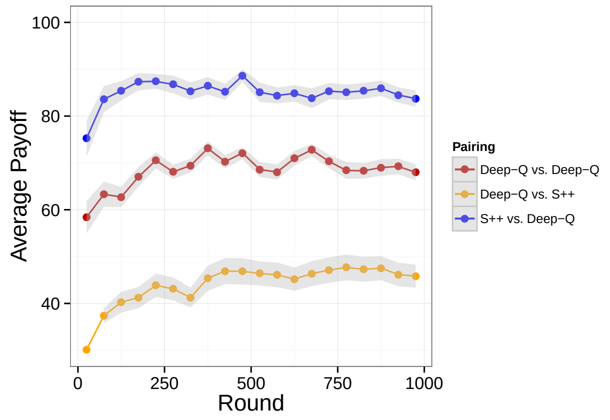

(c) Chicken – Average Payoffs

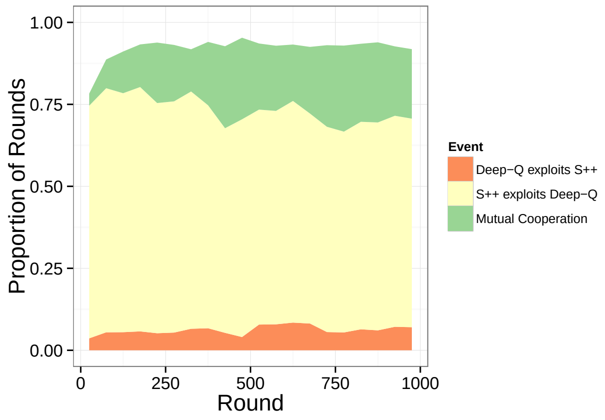

(d) Chicken – Joint Outcomes

Supplementary Figure 9: Analysis of the interaction between S++ and DEEP-Q in a Prisoner's Dilemma and in Chicken (Supplementary Table 10). All results are an average of 50 trials. (a) Average payoffs obtained by DEEP-Q in self play, and by DEEP-Q and S++ when they are paired with each other in the Prisoner's Dilemma. Error bands show the standard error of the mean. (b) The proportion of rounds that DEEP-Q and S++ cooperate together and exploit each other in the Prisoner's Dilemma. (c - d) Repeat of the evaluations presented in (a) and (b) in the game Chicken. These results demonstrate that S++ performs substantially better against DEEP-Q than DEEP-Q does against itself in both games. However, it does so for different reasons. In the Prisoner's Dilemma, S++'s superior performance is caused by its ability to elicit cooperation from DEEP-Q, whereas its superior performance in Chicken is brought about by its ability to exploit DEEP-Q. This illustrates the ability of S++ to adapt to its partner and the game in order to obtain high payoffs.

DEEP-Q in Chicken, it often exploits DEEP-Q (Supplementary Figure 9d) wherein S++ plays `straight` while DEEP-Q plays `swerve`. This results in increased payoffs to S++. Thus, the performance advantage of S++ over DEEP-Q in Chicken is due to its ability to learn to exploit DEEP-Q.

Which Algorithmic Mechanisms Makes S++ Successful? Of the 25 algorithms we considered, only S++ demonstrated each of the three desirable properties we articulated at the beginning of this Supplementary Note: game independence, partner independence, and fast and sustainable behavior. Given the many different algorithmic mechanisms used by these 25 algorithms, it is interesting to study exactly what about S++ brings about its superior performance. To better explain this analysis, we briefly overview S++ in order to give a general understanding of its algorithmic mechanisms. A detailed review of S++ is provided in Supplementary Note 4.

S++ is an expert algorithm. In each round, an expert algorithm uses a set of experts to determine which action to take. Thus, in creating an expert algorithm, an algorithm designer must do two things: (1) specify a set of experts and (2) define a mechanism for selecting which action to take given the advice provided by the experts.

Several different expert-selection mechanisms have been advocated in the literature. These mechanisms include the multiplicative weights update (represented here by WMA and Exp3), ε-greedy (represented by EEE), and aspiration learning (represented by S). S++ differs from these algorithms in the expert-selection mechanism. Like S, it uses aspiration learning. However, unlike S, it uses an additional pruning mechanism, based on an estimate of the potential payoffs that each expert could obtain, to more intelligently select experts. Thus, the first distinguishing attribute of S++ is its expert-selection mechanism.

S++ also differs from traditional expert algorithms in the set of experts it selects from. In the literature, the set of experts used by expert algorithms has typically been the set of pure strategies available to the player. This practice is not generally required, however, as most existing expert algorithms can be used with more sophisticated experts (as we did in our study). However, S++ was specifically created with a more sophisticated set of experts (though, again, it can be used with a simpler set). In our study, we implemented many of the expert algorithms with two different sets of experts: the SIMPLE set of experts (indicated by the postfix SIMPLE) and the SOPHISTICATED set of experts (no postfix). The SIMPLE set of experts consisted of the player's pure strategies, its maximin strategy, and the strategy in which the player alternates between actions.

Since expert algorithms differ from each other with respect to the set of experts and the expert-selection mechanism, we are interested in observing which of these algorithmic mechanisms has the most impact on performance. Supplementary Figure 10 shows the average normalized relative score (Supplementary Equation 6) averaged over all games and pairings of four expert-selection mechanisms (S++, S[6], EXP3, and EEE) given the two different sets of experts. The figure clearly demonstrates that, at all game lengths, both the expert-selection mechanism and the set of experts had a significant impact on the performance of the algorithms. All expert-selection mechanisms benefitted from having the more sophisticated set of experts at each game length. Furthermore, for both sets of experts, S++ substantially outperformed the other expert-selection mechanisms.

Supplementary Figure 11 shows that both the set of experts and the expert-selection mechanism are important to S++'s *generality* (i.e., the ability to adapt to many different game types) and *flexibility* (i.e., the ability to associate effectively with many different associates). The more sophisticated set of experts permits S++ to adapt to a variety of partners and game types, whereas algorithms that rely on a single strategy or a less sophisticated set of experts are only successful in particular kinds of games played with

[6]Note that S/SIMPLE was not among the original 25 algorithms included in our comparison.

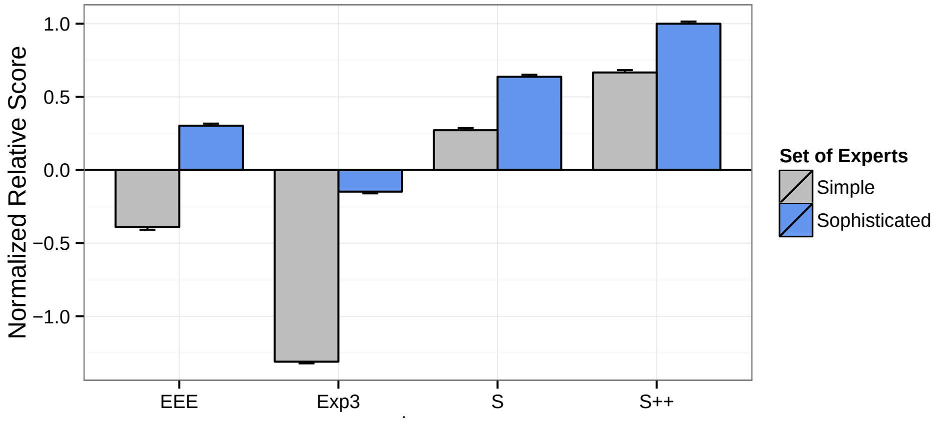

(a) 100-round games

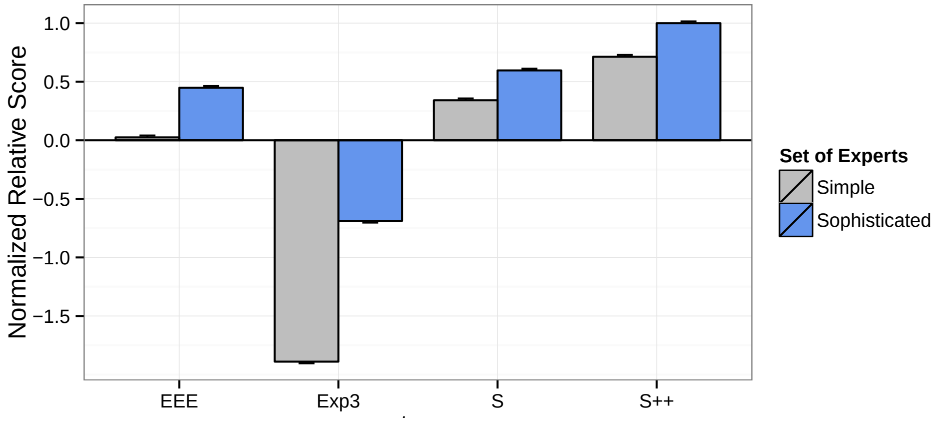

(b) 1000-round games

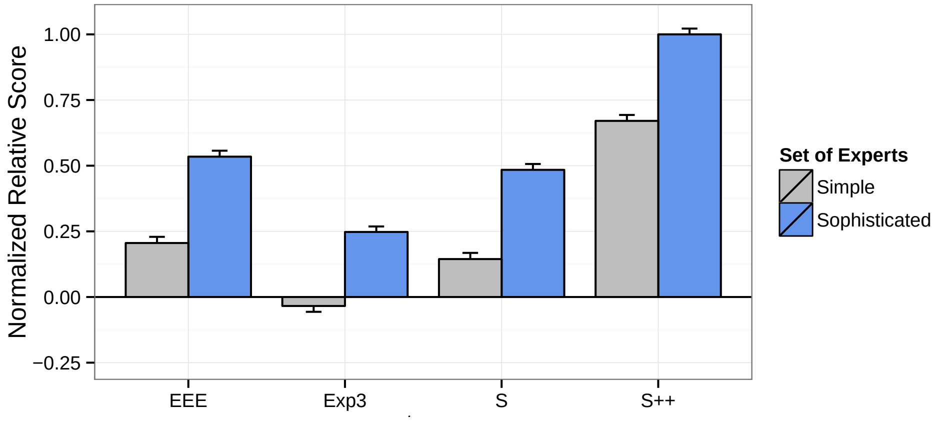

(c) 50,000-round games

Supplementary Figure 10: The average normalized relative score (Supplementary Equation 6) achieved by various expert algorithms achieved above the average across all games and pairings. The expert algorithms that operated on the sophisticated set of experts perform consistently better than algorithms that operated on the simple set of experts. Furthermore, the S++ expert-selection mechanism consistently outperformed the other three expert-selection mechanisms for both set of experts and at both game lengths. Thus, both the expert-selection mechanism and the set of experts used by S++ contributed substantially to its success.

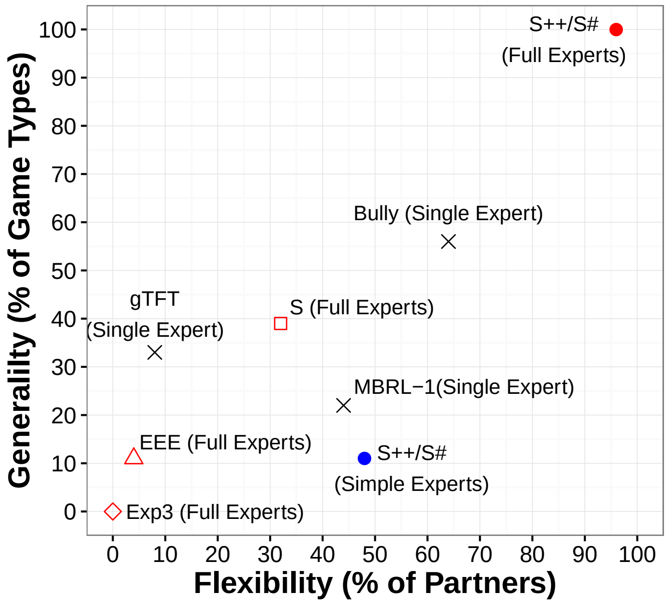

Supplementary Figure 11: The percentage of game types and partners against which various algorithms were ranked in the top-2 with respect to payoffs received among the 25 different algorithms evaluated. Flexibility (the x-axis) measures the percentage of the 25 algorithms against which an algorithm has the highest or second highest average payoff across the set of 2x2 games, as determined by two-way t-tests. Generality (the y-axis) measures the percentage of games types in which an algorithm has the highest or second highest average payoffs, as determined by two-way t-tests. Here, a game type defined by the combination of Robinson's and Goforth's payoff families [31] and the game length (100-rounds, 1000-rounds, or 50,000-rounds). Note that observing these percentages for top-3, top-4, or top-5 performance (rather than top-2 performance) returns similar relative values, though points tend to creep in the direction of the upper right-hand corner of the plot.

particular partners. Thus, in general, simplifying S++ by removing experts from this set will tend to limit the algorithm's flexibility and generality, though doing so will not always negatively impact its performance when paired with particular associates in particular games.

In the comparisons shown in Supplementary Figure 11, *Flexibility* was measured by comparing how well each algorithm did against each of the 25 selected algorithms across the set of 2x2 games. For each of the 25 partner algorithms, the average payoffs of each algorithm were compared with each other via two-way t-tests. Let $y_i(j) = 1$ if algorithm i had the either the first or second highest average payoff across all 2x2 games (as determined by two-way-t-tests) when associating with algorithm j. Otherwise, $y_i(j) = 0$. Then, algorithm i's flexibility is given by $\frac{1}{25}\sum_{j=1}^{25} y_i(j)$.

Generality is measured in a similar way, except that the algorithms were compared with respect to their ability to forge profitable relationships across all games of a particular type (averaged across all associates) rather than when paired with particular algorithms. Here, game types were defined by the combination of Robinson's and Goforth's payoff families [31] and the game length (100-rounds, 1000-rounds, or 50,000-rounds).

Reflections

While there are many strong algorithms for playing repeated games, S++ [1] was the only algorithm that we considered that demonstrated game independence, partner independence, and fast and sustainable behavior in our study. It was consistently among the top-performing algorithms regardless of the game length, payoff family, or partner family. Based on these results, we can conclude that S++ is a robust and effective algorithm when associating with other machines. Thus, after reviewing this algorithm in detail in Supplementary Note 4, we consider the ability of S++ to meet our second goal: that of establishing cooperative long-term relationships with people.

	S++	Fict. Play	Manip-ulator	Bully	S++/Simp	S	Mem-1	MBRL-1	WSLS	Mem-2	Gf (gTFT)	Manip-Gf	EEE	GIGA-WoLF	M-Qubed	MBRL-2	QL	WoLF-PHC	Exp3	EEE/Simp	Exp3/Simp	WMA	Stoch. FP	CJAL	Random	Ave
S++	0.680	0.744	0.650	0.651	0.696	0.722	0.729	0.766	0.767	0.733	0.740	0.740	0.728	0.696	0.685	0.737	0.701	0.679	0.665	0.730	0.644	0.624	0.624	0.612	0.610	**0.694**
Fict. Play	0.675	0.699	0.678	0.679	0.708	0.696	0.746	0.684	0.691	0.746	0.725	0.725	0.701	0.705	0.672	0.673	0.676	0.676	0.671	0.700	0.651	0.645	0.645	0.642	0.640	**0.686**
Manipulator	0.668	0.729	0.615	0.613	0.675	0.725	0.723	0.788	0.726	0.725	0.696	0.699	0.714	0.680	0.683	0.764	0.715	0.681	0.652	0.690	0.608	0.605	0.606	0.588	0.585	**0.678**
Bully	0.668	0.728	0.613	0.612	0.673	0.721	0.723	0.789	0.725	0.723	0.696	0.696	0.711	0.678	0.684	0.765	0.716	0.679	0.654	0.685	0.605	0.607	0.605	0.585	0.584	**0.677**
S++/simple	0.629	0.676	0.634	0.633	0.663	0.679	0.717	0.687	0.699	0.728	0.711	0.711	0.665	0.660	0.664	0.688	0.669	0.660	0.648	0.664	0.640	0.632	0.632	0.630	0.627	**0.666**
S	0.666	0.703	0.660	0.660	0.679	0.687	0.716	0.680	0.756	0.715	0.734	0.735	0.684	0.663	0.656	0.636	0.639	0.633	0.641	0.667	0.627	0.590	0.590	0.583	0.586	**0.663**
Mem-1	0.674	0.712	0.662	0.664	0.678	0.697	0.746	0.697	0.733	0.731	0.704	0.704	0.678	0.666	0.662	0.651	0.657	0.653	0.646	0.648	0.605	0.577	0.578	0.565	0.562	**0.662**
MBRL-1	0.658	0.714	0.671	0.672	0.673	0.660	0.701	0.658	0.754	0.685	0.738	0.739	0.672	0.669	0.623	0.616	0.620	0.626	0.641	0.680	0.643	0.600	0.600	0.597	0.600	**0.660**
WSLS	0.624	0.653	0.663	0.661	0.635	0.668	0.726	0.646	0.645	0.722	0.714	0.715	0.646	0.649	0.650	0.642	0.629	0.618	0.636	0.664	0.650	0.571	0.572	0.570	0.570	**0.646**
Mem-2	0.669	0.693	0.660	0.661	0.668	0.689	0.721	0.652	0.723	0.749	0.703	0.704	0.677	0.651	0.643	0.615	0.618	0.611	0.627	0.637	0.585	0.543	0.543	0.534	0.536	**0.645**
Gf (gTFT)	0.661	0.633	0.622	0.622	0.635	0.682	0.649	0.708	0.659	0.657	0.746	0.747	0.649	0.655	0.663	0.700	0.646	0.606	0.597	0.615	0.566	0.570	0.567	0.553	0.554	**0.638**
Manip-Gf	0.661	0.633	0.624	0.622	0.635	0.682	0.649	0.709	0.659	0.658	0.746	0.741	0.647	0.655	0.662	0.698	0.647	0.602	0.595	0.615	0.564	0.570	0.567	0.553	0.552	**0.638**
EEE	0.631	0.665	0.634	0.628	0.647	0.634	0.664	0.644	0.679	0.663	0.692	0.693	0.637	0.629	0.630	0.620	0.630	0.626	0.622	0.637	0.609	0.592	0.596	0.593	0.583	**0.635**
GIGA-WoLF	0.637	0.668	0.623	0.625	0.642	0.646	0.664	0.633	0.638	0.653	0.673	0.671	0.647	0.651	0.611	0.602	0.605	0.607	0.592	0.635	0.554	0.541	0.546	0.541	0.543	**0.618**
M-Qubed	0.623	0.631	0.617	0.617	0.634	0.648	0.682	0.604	0.726	0.683	0.698	0.699	0.636	0.610	0.597	0.579	0.577	0.581	0.601	0.635	0.593	0.538	0.534	0.535	0.533	**0.616**
MBRL-2	0.640	0.691	0.655	0.654	0.661	0.634	0.649	0.590	0.730	0.633	0.718	0.718	0.648	0.631	0.585	0.555	0.563	0.559	0.589	0.652	0.557	0.520	0.522	0.522	0.522	**0.616**
QL	0.618	0.654	0.628	0.629	0.637	0.618	0.649	0.582	0.681	0.635	0.685	0.685	0.624	0.606	0.564	0.558	0.556	0.565	0.592	0.625	0.576	0.525	0.526	0.522	0.524	**0.603**
WoLF-PHC	0.603	0.615	0.604	0.603	0.621	0.605	0.634	0.598	0.596	0.622	0.656	0.655	0.604	0.619	0.587	0.581	0.582	0.593	0.599	0.606	0.574	0.573	0.569	0.571	0.569	**0.602**
Exp3	0.592	0.605	0.584	0.586	0.598	0.604	0.617	0.599	0.620	0.610	0.649	0.648	0.609	0.586	0.586	0.582	0.582	0.592	0.603	0.600	0.581	0.575	0.576	0.570	0.571	**0.597**
EEE/simple	0.571	0.602	0.584	0.580	0.603	0.565	0.604	0.568	0.604	0.602	0.633	0.630	0.586	0.581	0.551	0.562	0.554	0.561	0.569	0.591	0.553	0.538	0.540	0.538	0.540	**0.576**
Exp3/simple	0.478	0.497	0.480	0.480	0.495	0.497	0.524	0.498	0.490	0.523	0.532	0.529	0.507	0.490	0.495	0.495	0.495	0.492	0.506	0.494	0.493	0.494	0.495	0.494	0.491	**0.499**
WMA	0.494	0.504	0.492	0.492	0.500	0.503	0.508	0.489	0.487	0.493	0.553	0.553	0.507	0.481	0.486	0.487	0.486	0.492	0.501	0.493	0.482	0.485	0.485	0.483	0.485	**0.497**
Stoch. FP	0.492	0.507	0.492	0.492	0.501	0.502	0.509	0.494	0.487	0.497	0.553	0.553	0.507	0.480	0.486	0.485	0.485	0.490	0.501	0.494	0.481	0.482	0.484	0.484	0.481	**0.497**
CJAL	0.450	0.461	0.452	0.452	0.458	0.469	0.472	0.460	0.459	0.467	0.512	0.511	0.466	0.456	0.458	0.460	0.457	0.460	0.473	0.456	0.455	0.459	0.460	0.460	0.459	**0.464**
Random	0.453	0.460	0.453	0.450	0.458	0.467	0.474	0.458	0.455	0.466	0.512	0.512	0.472	0.462	0.456	0.457	0.459	0.455	0.468	0.461	0.458	0.460	0.460	0.459	0.455	**0.464**

Supplementary Table 12: The average per-round payoff for each pairing averaged over all 720 2x2 games in 100-round interactions. The values show the average payoff of the algorithm indicated on the row when it was paired with the algorithm listed in the column. As points of comparison, the average Nash bargaining solution over all games was 0.777, and the average payoff over all outcomes of all games to each player was 0.458.

	S++	Manip-ulator	Bully	S++/Simp	S	MBRL-2	MBRL-1	Fict. Play	EEE	Mem-1	Mem-2	Manip-Gf	WoLF-PHC	CJAL	Gf (gTFT)	EEE/Simp	WSLS	QL	GIGA-WoLF	M-Qubed	WMA	Stoch. FP	Exp3	Exp3/Simp	Random	Ave
S++	0.760	0.729	0.697	0.752	0.777	0.776	0.788	0.770	0.759	0.753	0.759	0.786	0.732	0.738	0.770	0.768	0.800	0.737	0.721	0.707	0.682	0.681	0.678	0.679	0.623	**0.737**
Manipulator	0.760	0.711	0.669	0.759	0.774	0.793	0.797	0.745	0.767	0.739	0.739	0.765	0.727	0.736	0.745	0.757	0.764	0.750	0.716	0.696	0.687	0.687	0.672	0.650	0.601	**0.728**
Bully	0.800	0.755	0.612	0.796	0.803	0.810	0.814	0.730	0.787	0.724	0.728	0.784	0.716	0.725	0.695	0.765	0.726	0.756	0.683	0.700	0.688	0.687	0.670	0.634	0.585	**0.727**
S++/simple	0.748	0.709	0.692	0.747	0.766	0.744	0.743	0.746	0.731	0.744	0.754	0.776	0.722	0.713	0.766	0.731	0.783	0.712	0.728	0.678	0.666	0.665	0.652	0.620	0.596	**0.717**
S	0.734	0.708	0.695	0.736	0.755	0.705	0.730	0.738	0.720	0.742	0.748	0.780	0.690	0.709	0.769	0.712	0.800	0.683	0.701	0.684	0.656	0.655	0.651	0.641	0.592	**0.709**
MBRL-2	0.707	0.695	0.694	0.724	0.755	0.696	0.707	0.748	0.710	0.735	0.745	0.767	0.667	0.702	0.763	0.709	0.794	0.704	0.685	0.710	0.662	0.662	0.669	0.665	0.622	**0.708**
MBRL-1	0.688	0.701	0.695	0.692	0.707	0.694	0.704	0.745	0.702	0.746	0.741	0.774	0.692	0.719	0.764	0.716	0.797	0.672	0.694	0.673	0.676	0.676	0.675	0.685	0.634	**0.707**
Fict. Play	0.689	0.705	0.679	0.707	0.713	0.693	0.693	0.699	0.695	0.748	0.752	0.754	0.708	0.722	0.725	0.706	0.693	0.697	0.706	0.678	0.686	0.685	0.676	0.655	0.643	**0.700**
EEE	0.695	0.694	0.683	0.695	0.717	0.704	0.713	0.718	0.699	0.732	0.733	0.764	0.686	0.701	0.753	0.697	0.763	0.694	0.679	0.684	0.666	0.665	0.662	0.660	0.622	**0.699**
Mem-1	0.730	0.718	0.664	0.729	0.734	0.713	0.724	0.714	0.720	0.746	0.733	0.744	0.697	0.702	0.704	0.709	0.737	0.684	0.670	0.663	0.668	0.667	0.657	0.626	0.563	**0.697**
Mem-2	0.730	0.703	0.664	0.732	0.740	0.691	0.690	0.699	0.714	0.722	0.751	0.733	0.662	0.678	0.705	0.693	0.736	0.661	0.655	0.655	0.624	0.624	0.637	0.603	0.532	**0.681**
Manip-Gf	0.718	0.685	0.659	0.717	0.728	0.728	0.723	0.675	0.704	0.674	0.681	0.756	0.652	0.662	0.760	0.691	0.702	0.676	0.682	0.673	0.635	0.634	0.622	0.614	0.573	**0.681**
WoLF-PHC	0.678	0.688	0.661	0.679	0.690	0.673	0.680	0.685	0.682	0.717	0.708	0.738	0.687	0.699	0.710	0.681	0.664	0.660	0.679	0.659	0.667	0.667	0.657	0.632	0.624	**0.679**
CJAL	0.679	0.687	0.663	0.667	0.696	0.679	0.672	0.669	0.666	0.723	0.722	0.742	0.673	0.677	0.707	0.651	0.680	0.660	0.657	0.660	0.658	0.658	0.656	0.632	0.625	**0.674**
Gf (gTFT)	0.740	0.703	0.622	0.741	0.748	0.737	0.728	0.634	0.719	0.649	0.661	0.759	0.633	0.638	0.746	0.682	0.660	0.676	0.657	0.675	0.624	0.624	0.613	0.588	0.552	**0.672**
EEE/simple	0.669	0.679	0.663	0.667	0.681	0.681	0.688	0.676	0.670	0.712	0.711	0.729	0.656	0.671	0.715	0.659	0.687	0.667	0.662	0.659	0.645	0.640	0.638	0.617	0.612	**0.670**
WSLS	0.662	0.694	0.663	0.656	0.673	0.666	0.667	0.655	0.657	0.732	0.733	0.729	0.639	0.657	0.715	0.669	0.647	0.653	0.664	0.660	0.620	0.621	0.640	0.653	0.572	**0.664**
QL	0.684	0.683	0.673	0.703	0.711	0.630	0.637	0.712	0.667	0.701	0.700	0.743	0.636	0.631	0.726	0.659	0.748	0.631	0.657	0.629	0.603	0.604	0.625	0.606	0.542	**0.662**
GIGA-WoLF	0.685	0.670	0.630	0.663	0.688	0.663	0.676	0.679	0.678	0.672	0.661	0.697	0.678	0.697	0.677	0.668	0.661	0.639	0.670	0.629	0.632	0.632	0.606	0.569	0.549	**0.655**
M-Qubed	0.676	0.647	0.630	0.706	0.715	0.628	0.635	0.654	0.653	0.691	0.704	0.724	0.640	0.637	0.709	0.636	0.745	0.628	0.636	0.634	0.600	0.599	0.612	0.590	0.535	**0.651**
WMA	0.638	0.647	0.632	0.636	0.648	0.633	0.634	0.636	0.643	0.671	0.649	0.696	0.627	0.638	0.687	0.636	0.629	0.617	0.627	0.617	0.619	0.620	0.624	0.599	0.587	**0.636**
Stoch. FP	0.637	0.646	0.631	0.637	0.646	0.630	0.638	0.638	0.640	0.670	0.649	0.696	0.631	0.639	0.687	0.629	0.629	0.617	0.620	0.617	0.618	0.618	0.623	0.598	0.587	**0.635**
Exp3	0.623	0.621	0.605	0.610	0.623	0.623	0.633	0.630	0.629	0.638	0.629	0.677	0.617	0.622	0.669	0.622	0.650	0.604	0.608	0.604	0.604	0.603	0.615	0.597	0.580	**0.621**
Exp3/simple	0.529	0.534	0.525	0.529	0.538	0.536	0.543	0.545	0.537	0.568	0.564	0.580	0.536	0.536	0.577	0.537	0.537	0.530	0.536	0.528	0.530	0.529	0.535	0.523	0.519	**0.539**
Random	0.454	0.470	0.451	0.458	0.468	0.455	0.459	0.458	0.466	0.471	0.466	0.511	0.455	0.459	0.512	0.462	0.458	0.458	0.453	0.457	0.459	0.458	0.469	0.458	0.457	**0.464**

Supplementary Table 13: The average per-round payoff for each pairing averaged over all 720 2x2 games in 1000-round interactions. The values show the average payoff of the algorithm indicated on the row when it was paired with the algorithm listed in the column. As points of comparison, the average Nash bargaining solution over all games was 0.777, and the average payoff over all outcomes of all games to each player was 0.458.

	S++	Bully	Manip-ulator	M-Qubed	S++/Simp	MBRL-2	QL	EEE	S	MBRL-1	Exp3	EEE/Simp	Mem-1	WoLF PHC	Fict. Play	WMA	CJAL	Stoch. FP	Exp3/Simp	Mem-2	Manip-Gf	Gf (gTFT)	GIGA-WoLF	WSLS	Random	Ave
S++	0.773	0.703	0.750	0.757	0.766	0.793	0.775	0.781	0.795	0.793	0.721	0.785	0.757	0.755	0.775	0.765	0.733	0.730	0.734	0.763	0.797	0.775	0.724	0.804	0.626	**0.757**
Bully	0.822	0.612	0.789	0.816	0.822	0.822	0.818	0.818	0.823	0.822	0.778	0.799	0.724	0.731	0.730	0.745	0.727	0.727	0.766	0.728	0.800	0.695	0.683	0.726	0.585	**0.756**
Manipulator	0.775	0.682	0.734	0.775	0.770	0.804	0.777	0.780	0.789	0.799	0.736	0.779	0.743	0.746	0.749	0.760	0.730	0.729	0.740	0.743	0.785	0.757	0.721	0.774	0.605	**0.751**
M-Qubed	0.772	0.701	0.719	0.762	0.806	0.725	0.763	0.741	0.811	0.716	0.707	0.742	0.755	0.752	0.743	0.741	0.715	0.715	0.717	0.764	0.790	0.773	0.720	0.807	0.637	**0.744**
S++/simple	0.768	0.701	0.725	0.718	0.765	0.767	0.755	0.763	0.786	0.759	0.700	0.766	0.748	0.754	0.758	0.738	0.716	0.715	0.700	0.758	0.791	0.775	0.740	0.795	0.590	**0.742**
MBRL-2	0.730	0.703	0.712	0.779	0.747	0.734	0.781	0.738	0.798	0.740	0.707	0.735	0.758	0.713	0.760	0.749	0.719	0.719	0.713	0.772	0.781	0.773	0.706	0.808	0.642	**0.741**
QL	0.749	0.703	0.716	0.757	0.754	0.714	0.757	0.734	0.792	0.713	0.703	0.727	0.756	0.746	0.739	0.740	0.710	0.712	0.719	0.764	0.790	0.772	0.710	0.807	0.638	**0.737**
EEE	0.730	0.703	0.719	0.761	0.730	0.752	0.760	0.727	0.763	0.735	0.710	0.730	0.757	0.720	0.731	0.742	0.716	0.719	0.722	0.763	0.786	0.773	0.696	0.802	0.641	**0.736**
S	0.748	0.702	0.722	0.723	0.751	0.727	0.735	0.742	0.773	0.747	0.706	0.737	0.745	0.725	0.744	0.737	0.715	0.715	0.713	0.752	0.789	0.775	0.709	0.806	0.594	**0.733**
MBRL-1	0.699	0.702	0.711	0.732	0.703	0.720	0.730	0.718	0.725	0.715	0.699	0.733	0.758	0.717	0.752	0.757	0.720	0.721	0.722	0.756	0.782	0.771	0.702	0.809	0.643	**0.728**
Exp3	0.732	0.690	0.719	0.741	0.715	0.740	0.743	0.721	0.738	0.733	0.709	0.719	0.746	0.705	0.717	0.726	0.692	0.691	0.700	0.746	0.773	0.759	0.680	0.790	0.633	**0.722**
EEE/simple	0.712	0.694	0.719	0.742	0.707	0.742	0.744	0.724	0.736	0.740	0.717	0.704	0.752	0.706	0.704	0.721	0.706	0.710	0.695	0.757	0.768	0.747	0.696	0.726	0.640	**0.720**
Mem-1	0.737	0.664	0.729	0.730	0.740	0.735	0.731	0.734	0.744	0.732	0.719	0.736	0.746	0.715	0.713	0.718	0.714	0.714	0.719	0.734	0.751	0.704	0.669	0.737	0.563	**0.717**
WoLF-PHC	0.703	0.681	0.718	0.714	0.706	0.709	0.715	0.711	0.727	0.705	0.712	0.706	0.746	0.727	0.707	0.742	0.728	0.731	0.715	0.748	0.767	0.731	0.694	0.691	0.642	**0.715**
Fict. Play	0.693	0.679	0.711	0.705	0.713	0.696	0.709	0.699	0.724	0.694	0.706	0.725	0.749	0.729	0.699	0.753	0.719	0.719	0.713	0.752	0.760	0.725	0.708	0.693	0.643	**0.713**
CJAL	0.712	0.687	0.720	0.719	0.699	0.715	0.717	0.697	0.729	0.701	0.707	0.688	0.750	0.716	0.693	0.722	0.718	0.718	0.704	0.751	0.774	0.730	0.681	0.709	0.643	**0.712**
WMA	0.713	0.681	0.709	0.722	0.709	0.703	0.723	0.709	0.723	0.697	0.714	0.713	0.748	0.706	0.696	0.717	0.705	0.713	0.714	0.748	0.750	0.729	0.688	0.723	0.642	**0.712**
Stoch. FP	0.711	0.681	0.708	0.726	0.711	0.702	0.721	0.713	0.724	0.700	0.715	0.714	0.748	0.705	0.700	0.716	0.707	0.707	0.713	0.748	0.748	0.729	0.682	0.722	0.642	**0.712**
Exp3/simple	0.725	0.682	0.716	0.739	0.704	0.726	0.735	0.720	0.723	0.729	0.719	0.693	0.740	0.692	0.684	0.698	0.685	0.684	0.688	0.742	0.757	0.734	0.674	0.713	0.632	**0.709**
Mem-2	0.739	0.664	0.710	0.729	0.748	0.709	0.726	0.728	0.752	0.702	0.703	0.718	0.723	0.693	0.700	0.694	0.690	0.688	0.697	0.751	0.737	0.705	0.655	0.738	0.531	**0.705**
Manip-Gf	0.730	0.667	0.699	0.721	0.727	0.735	0.716	0.721	0.740	0.725	0.676	0.716	0.679	0.671	0.684	0.685	0.662	0.660	0.691	0.686	0.762	0.764	0.689	0.713	0.578	**0.700**
Gf (gTFT)	0.761	0.622	0.726	0.751	0.765	0.745	0.742	0.749	0.766	0.734	0.698	0.712	0.650	0.645	0.634	0.649	0.639	0.640	0.682	0.661	0.763	0.746	0.658	0.660	0.551	**0.694**
GIGA-WoLF	0.697	0.630	0.682	0.692	0.669	0.694	0.697	0.701	0.699	0.688	0.691	0.688	0.671	0.714	0.680	0.725	0.692	0.696	0.689	0.662	0.704	0.677	0.673	0.665	0.550	**0.681**
WSLS	0.668	0.663	0.702	0.672	0.658	0.673	0.672	0.666	0.672	0.673	0.663	0.665	0.733	0.645	0.655	0.670	0.659	0.659	0.659	0.735	0.733	0.715	0.666	0.647	0.572	**0.672**
Random	0.455	0.451	0.473	0.458	0.458	0.456	0.458	0.464	0.468	0.457	0.462	0.459	0.471	0.458	0.455	0.459	0.459	0.458	0.458	0.466	0.510	0.512	0.451	0.458	0.458	**0.464**

Supplementary Table 14: The average per-round payoff for each pairing averaged over all 720 2x2 games in 50,000-round interactions. The values show the average payoff of the algorithm indicated on the row when it was paired with the algorithm listed in the column. As points of comparison, the average Nash bargaining solution over all games was 0.777, and the average payoff over all outcomes of all games to each player was 0.458.

Algorithm	Round-Robin Average	% Best Score	Worst-Case Score	Replicator Dynamic	Group-1 Tourney (adv/win)	Group-2 Tourney (adv1/adv2/win)	Average Rank
S++	0.694 (1)	56% (2)	0.048 (1)	82% (1)	98% / 83% (1)	100% / 100% / 92% (1)	1.17
FICT. PLAY	0.686 (2)	56% (1)	0.043 (2)	6% (3)	71% / 9% (2)	98% / 74% / 0% (4)	2.33
MEM-1	0.662 (6)	16% (7)	0.011 (6)	4% (2)	35% / 7% (3)	84% / 8% / 8% (2)	4.33
MANIPULATOR	0.678 (3)	36% (4)	0.011 (5)	2% (6)	50% / 0% (5)	87% / 13% / 0% (5)	4.67
BULLY	0.677 (3)	36% (3)	0.011 (7)	2% (7)	41% / 0% (6)	77% / 4% / 0% (6)	5.33
S	0.663 (5)	16% (6)	0.028 (3)	1% (5)	27% / 0% (7)	72% / 1% / 0% (7)	5.50
MEM-2	0.645 (9)	12% (9)	-0.017 (13)	1% (4)	8% / 1% (4)	42% / 0% / 0% (3)	7.00
S++/SIMPLE	0.666 (5)	12% (8)	0.020 (4)	0% (10)	34% / 0% (8)	67% / 0% / 0% (9)	7.33
MBRL-1	0.660 (6)	24% (5)	0.008 (8)	1% (11)	13% / 0% (9)	49% / 0% / 0% (8)	7.83
GF (GTFT)	0.638 (11)	8% (11)	-0.008 (11)	0% (8)	9% / 0% (10)	33% / 0% / 0% (10)	10.17
WSLS	0.646 (9)	12% (10)	-0.003 (10)	0% (12)	4% / 0% (12)	24% / 0% / 0% (12)	10.83
MANIP-GF	0.638 (11)	8% (12)	-0.010 (12)	0% (9)	4% / 0% (11)	25% / 0% / 0% (11)	11.00
EEE	0.635 (11)	0% (14)	0.006 (9)	0% (14)	3% / 0% (13)	19% / 0% / 0% (13)	12.33
GIGA-WOLF	0.618 (14)	0% (17)	-0.020 (14)	0% (13)	1% / 0% (14)	9% / 0% / 0% (14)	14.33
M-QUBED	0.616 (14)	0% (15)	-0.030 (15)	0% (17)	0% / 0% (17)	4% / 0% / 0% (16)	15.67
EXP3	0.597 (19)	0% (16)	-0.034 (16)	0% (15)	1% / 0% (15)	3% / 0% / 0% (17)	16.33
WOLF-PHC	0.602 (17)	0% (18)	-0.051 (18)	0% (16)	0% / 0% (16)	4% / 0% / 0% (15)	16.67
MBRL-2	0.616 (14)	0% (13)	-0.053 (19)	0% (18)	0% / 0% (18)	1% / 0% / 0% (18)	16.67
QL	0.603 (17)	0% (19)	-0.051 (17)	0% (19)	0% / 0% (19)	1% / 0% / 0% (19)	18.33
EEE/SIMPLE	0.576 (20)	0% (20)	-0.063 (20)	0% (20)	0% / 0% (19)	0% / 0% / 0% (20)	19.83
EXP3/SIMPLE	0.499 (21)	0% (23)	-0.157 (21)	0% (21)	0% / 0% (19)	0% / 0% / 0% (21)	21.00
WMA	0.497 (21)	0% (21)	-0.160 (22)	0% (22)	0% / 0% (19)	0% / 0% / 0% (22)	21.17
STOCH. FP	0.497 (21)	0% (22)	-0.160 (23)	0% (23)	0% / 0% (19)	0% / 0% / 0% (22)	21.67
CJAL	0.464 (24)	0% (25)	-0.189 (24)	0% (24)	0% / 0% (19)	0% / 0% / 0% (22)	23.00
RANDOM	0.464 (24)	0% (24)	-0.192 (25)	0% (25)	0% / 0% (19)	0% / 0% / 0% (22)	23.17

Supplementary Table 15: Performance in 100-round games with respect to six metrics (Supplementary Table 5) derived from the data shown in Supplementary Table 12. See the subsection entitled *Evaluation Metrics* in this Supplementary Note for a description of the metrics. The rank of each algorithm with respect to each metric is given in parenthesis (lower is better). In the *Group-1 Tournament*, two numbers are supplied: the percentage of trials (out of 10,000) that the algorithm advanced to the final round and the percentage of trials the algorithm won. Similarly, in the *Group-2 Tourneyament*, the three numbers are the percentage of trials the algorithm advanced to the second round, advanced to the final round, and won the tournament, respectively.

Algorithm	Round-Robin Average	% Best Score	Worst-Case Score	Replicator Dynamic	Group-1 Tourney (adv/win)	Group-2 Tourney (adv1/adv2/win)	Average Rank
S++	0.737 (1)	68% (1)	0.042 (1)	60% (1)	98% / 59% (1)	100% / 100% / 100% (1)	1.00
MANIPULATOR	0.728 (2)	32% (3)	0.020 (2)	3% (4)	73% / 8% (3)	97% / 8% / 0% (2)	2.67
BULLY	0.727 (2)	56% (2)	-0.034 (13)	31% (3)	45% / 32% (2)	92% / 91% / 0% (3)	4.17
S++/SIMPLE	0.717 (4)	28% (5)	0.004 (6)	2% (2)	53% / 0% (4)	91% / 1% / 0% (4)	4.17
S	0.709 (5)	24% (7)	0.011 (3)	1% (5)	34% / 0% (5)	79% / 0% / 0% (5)	5.00
MBRL-2	0.708 (5)	20% (8)	0.009 (5)	0% (9)	26% / 0% (8)	66% / 0% / 0% (6)	6.83
MBRL-1	0.707 (6)	32% (4)	0.004 (7)	0% (11)	26% / 0% (6)	61% / 0% / 0% (7)	6.83
MEM-1	0.697 (9)	4% (10)	-0.018 (9)	0% (6)	10% / 0% (7)	47% / 0% / 0% (8)	8.17
EEE	0.699 (8)	4% (9)	0.010 (4)	0% (10)	11% / 0% (10)	42% / 0% / 0% (10)	8.50
FICT. PLAY	0.700 (8)	28% (6)	-0.007 (8)	0% (12)	15% / 0% (9)	45% / 0% / 0% (9)	8.67
MANIP-GF	0.681 (11)	4% (12)	-0.022 (11)	0% (7)	2% / 0% (12)	16% / 0% / 0% (13)	11.00
WOLF-PHC	0.679 (11)	4% (14)	-0.036 (14)	0% (14)	4% / 0% (11)	20% / 0% / 0% (11)	12.50
MEM-2	0.681 (11)	4% (11)	-0.049 (17)	0% (13)	2% / 0% (14)	18% / 0% / 0% (12)	13.00
CJAL	0.674 (14)	4% (14)	-0.022 (12)	0% (17)	2% / 0% (13)	12% / 0% / 0% (14)	14.00
GF (GTFT)	0.672 (14)	0% (15)	-0.047 (16)	0% (8)	0% / 0% (16)	6% / 0% / 0% (15)	14.00
EEE/SIMPLE	0.670 (15)	0% (17)	-0.019 (10)	0% (16)	1% / 0% (15)	6% / 0% / 0% (16)	14.83
GIGA-WOLF	0.655 (19)	0% (18)	-0.047 (15)	0% (15)	0% / 0% (20)	1% / 0% / 0% (19)	17.67
WSLS	0.664 (17)	0% (16)	-0.052 (20)	0% (20)	0% / 0% (17)	2% / 0% / 0% (17)	17.83
QL	0.662 (17)	0% (19)	-0.049 (18)	0% (18)	0% / 0% (18)	2% / 0% / 0% (18)	18.00
M-QUBED	0.651 (20)	0% (20)	-0.051 (19)	0% (19)	0% / 0% (20)	0% / 0% / 0% (21)	19.83
WMA	0.636 (21)	0% (21)	-0.070 (21)	0% (21)	0% / 0% (19)	0% / 0% / 0% (20)	20.50
STOCH. FP	0.635 (21)	0% (22)	-0.071 (22)	0% (22)	0% / 0% (20)	0% / 0% / 0% (22)	21.50
EXP3	0.621 (23)	0% (23)	-0.077 (23)	0% (23)	0% / 0% (20)	0% / 0% / 0% (23)	22.50
EXP3/SIMPLE	0.539 (24)	0% (24)	-0.162 (24)	0% (24)	0% / 0% (20)	0% / 0% / 0% (23)	23.17
RANDOM	0.464 (25)	0% (25)	-0.242 (25)	0% (25)	0% / 0% (20)	0% / 0% / 0% (23)	23.83

Supplementary Table 16: Performance in 1000-round games with respect to six metrics (Supplementary Table 5) derived from the data shown in Supplementary Table 12. See the subsection entitled *Evaluation Metrics* in this Supplementary Note for a description of the metrics. The rank of each algorithm with respect to each metric is given in parenthesis (lower is better). In the *Group-1 Tournament*, two numbers are supplied: the percentage of trials (out of 10,000) that the algorithm advanced to the final round and the percentage of trials the algorithm won. Similarly, in the *Group-2 Tourneyament*, the three numbers are the percentage of trials the algorithm advanced to the second round, advanced to the final round, and won the tournament, respectively.

Algorithm	Round-Robin Average	% Best Score	Worst-Case Score	Replicator Dynamic	Group-1 Tourney (adv/win)	Group-2 Tourney (adv1/adv2/win)	Average Rank
S++	0.757 (1)	56% (2)	0.021 (1)	49% (1)	89% / 30% (2)	100% / 87% / 87% (1)	1.33
MANIPULATOR	0.751 (3)	24% (8)	0.000 (4)	4% (3)	65% / 18% (3)	95% / 29% / 12% (2)	3.83
M-QUBED	0.744 (4)	40% (3)	-0.002 (5)	5% (5)	49% / 9% (4)	86% / 2% / 2% (3)	4.00
MBRL-2	0.741 (5)	28% (6)	0.002 (3)	1% (4)	37% / 0% (6)	76% / 0% / 0% (4)	4.67
BULLY	0.756 (1)	60% (1)	-0.058 (20)	34% (2)	47% / 44% (1)	82% / 82% / 0% (5)	5.00
QL	0.737 (7)	32% (4)	-0.007 (7)	1% (7)	22% / 0% (7)	61% / 0% / 0% (7)	6.50
EEE	0.736 (7)	32% (5)	0.007 (2)	0% (9)	16% / 0% (8)	54% / 0% / 0% (8)	6.50
S++/SIMPLE	0.742 (4)	24% (9)	-0.015 (10)	2% (6)	38% / 0% (5)	77% / 0% / 0% (6)	6.67
S	0.733 (8)	16% (10)	-0.011 (8)	1% (8)	15% / 0% (9)	49% / 0% / 0% (9)	8.67
MBRL-1	0.728 (10)	28% (7)	-0.023 (14)	0% (13)	8% / 0% (10)	31% / 0% / 0% (10)	10.67
EXP3	0.722 (11)	12% (15)	-0.007 (6)	0% (12)	3% / 0% (13)	16% / 0% / 0% (12)	11.50
EEE/SIMPLE	0.720 (11)	12% (12)	-0.014 (9)	0% (11)	1% / 0% (14)	13% / 0% / 0% (13)	11.67
WOLF-PHC	0.715 (13)	12% (14)	-0.042 (18)	0% (14)	4% / 0% (11)	19% / 0% / 0% (11)	13.50
FICT. PLAY	0.713 (14)	16% (10)	-0.040 (16)	0% (15)	3% / 0% (12)	12% / 0% / 0% (14)	13.50
CJAL	0.712 (14)	12% (13)	-0.028 (15)	0% (16)	1% / 0% (16)	7% / 0% / 0% (16)	15.00
WMA	0.712 (15)	4% (16)	-0.021 (12)	0% (17)	1% / 0% (15)	6% / 0% / 0% (17)	15.33
MEM-1	0.717 (13)	0% (21)	-0.042 (17)	0% (10)	1% / 0% (17)	10% / 0% / 0% (15)	15.50
STOCH. FP	0.712 (15)	4% (17)	-0.019 (11)	0% (18)	0% / 0% (18)	4% / 0% / 0% (18)	16.17
EXP3/SIMPLE	0.709 (16)	4% (18)	-0.022 (13)	0% (21)	0% / 0% (19)	1% / 0% / 0% (19)	17.67
MANIP-GF	0.700 (21)	4% (19)	-0.047 (19)	0% (20)	0% / 0% (20)	1% / 0% / 0% (20)	19.83
MEM-2	0.705 (20)	0% (22)	-0.074 (22)	0% (19)	0% / 0% (21)	0% / 0% / 0% (21)	20.83
GF (GTFT)	0.694 (22)	0% (20)	-0.076 (23)	0% (22)	0% / 0% (21)	0% / 0% / 0% (22)	21.67
GIGA-WOLF	0.681 (23)	0% (23)	-0.068 (21)	0% (23)	0% / 0% (21)	0% / 0% / 0% (22)	22.17
WSLS	0.672 (24)	0% (24)	-0.085 (24)	0% (24)	0% / 0% (21)	0% / 0% / 0% (22)	23.17
RANDOM	0.464 (25)	0% (25)	-0.275 (25)	0% (25)	0% / 0% (21)	0% / 0% / 0% (22)	23.83

Supplementary Table 17: Performance in 50,000-round games with respect to six metrics (Supplementary Table 5) derived from the data shown in Supplementary Table 12. See the subsection entitled *Evaluation Metrics* in this Supplementary Note for a description of the metrics. The rank of each algorithm with respect to each metric is given in parenthesis (lower is better). In the *Group-1 Tournament*, two numbers are supplied: the percentage of trials (out of 10,000) that the algorithm advanced to the final round and the percentage of trials the algorithm won. Similarly, in the *Group-2 Tourneyament*, the three numbers are the percentage of trials the algorithm advanced to the second round, advanced to the final round, and won the tournament, respectively.

Supplementary Note 4

Descriptions of S++ and S#

The results of the empirical evaluation of algorithms in Supplementary Note 3 demonstrate that S++ [1] establishes effective strategies in two-player repeated games. Relative to the performance of the other 24 algorithms tested, S++ was a top-performing algorithm across the full set structurally distinct 2x2 games (assuming players have strict preference orderings over the four game outcomes) at all game lengths. Given its success in these evaluations, S++, along with an extended version of the algorithm called S# (pronounced 'S sharp'), became the focus of the remainder of this paper.

In this section, we first review S++ in detail. We then describe how S++ is extended to form S#.

A Review of S++

S++ was originally designed for repeated normal-form games [1], and was later generalized to repeated stochastic games (RSGs) [2][7]. S++ is an expert algorithm[8]. In each round t, an expert algorithm (employed by player i) selects an expert ϕ from a set of experts Φ_i. This expert then dictates the strategy executed by player i in round t. The set Φ_i essentially reduces the strategy space of the game to a handful of high-level strategies. Each expert defines a strategy over the entire state-space of the game. Thus, rather than learning a separate policy in each state $\sigma \in \Sigma$, the player must learn which high-level strategies or algorithms in Φ_i are the most profitable in the current scenario.

Thus, S++ is defined by two things: (1) its set of experts Φ_i (which are automatically generated from the description of the game) and (2) its mechanism for selecting which expert to execute in each round (called its expert-selection mechanism). We discuss both aspects of S++ in turn.

Computing the Set of Experts Φ_i Littman and Stone [52] classified algorithms for repeated games into two categories: *leaders* and *followers*[9], each of which is successful in different circumstances. A good set of experts must contain both leader and follower experts. Such experts can be computed automatically for any RSG [2]. For simplicity, our review focuses only on normal-form games, borrowing heavily from the work of Crandall [1]. A method for computing a similar set of experts for multi-stage RSGs has also been defined by Crandall [2].

Leader Experts. Leader strategies are designed to encourage the associate to play its portion of a *target solution* [52], which is a sequence of joint actions that are played repetitively. Each solution χ produces an expected payoff profile $\mathbf{v}(\chi) = (v_i(\chi), v_{-i}(\chi))$. For example, in the Prisoner's Dilemma (Supplementary Table 4), the solution $\chi = <(a,d),(b,d)>$ indicates that the column player always cooperates while the row player alternates between cooperating and defecting. This produces the expected payoff profile $\mathbf{v}(\chi) = (80, 30)$.

S++ derives a separate leader expert corresponding to each member of a set Ω of target solutions. Each leader expert is coupled with a trigger-mechanism that incentivizes the partner to play its portion of χ. When

[7] The generalization of S++ to RSGs was named MEGA-S++ [2]. MEGA-S++ is identical to S++ except that the set of experts defined for MEGA-S++ are designed to produce strategies in general (multi-stage) RSGs rather than just normal-form games. In this work, we simply refer to both versions as S++.

[8] We adopt the notation used in the published literature [1, 2]. This notation differs from the notation used in the main body of this paper, which was used in attempt to be more assessable to a general audience.

[9] Crandall [2] identified a third category called *preventative strategies*. Preventative strategies are sometimes successful when $|S| > 1$.

No.	Target solution (χ)	$v_1(\chi)$	Strategy
1	$<(a,d),(b,d)>$	80	If $g_2^t > 0$, play π_i^{attack}. Otherwise, play own portion of the current joint action in the sequence χ.
2	$<(a,d)>$	60	
3	$<(b,d),(a,c)>$	50	
4	$<(a,d),(b,c)>$	40	
5	$<(a,d),(a,c)>$	30	
6	$<(b,c)>$	20	

Supplementary Table 18: Leader experts generated for player 1 in the Prisoner's Dilemma (Supplementary Table 4). Adapted from Crandall [1].

its partner has conformed with χ or has not profited by deviating from it, the leader expert plays its portion of χ. On the other hand, if the partner has increased its payoffs by deviating from χ, the leader plays its attack policy, given by

$$\pi_i^{\text{attack}} = \arg\min_{\pi_i}\max_{\pi_{-i}} u_{-i}(\pi_i, \pi_{-i}), \qquad 15$$

where $u_{-i}(\pi_i, \pi_{-i})$ is player $-i$'s expected payoff when the players use strategies π_i and π_{-i}, respectively.

Formally, each leader expert keeps track of a guilt parameter g_{-i}^t that defines how much its associate has benefited from deviating from χ. Initially, $g_{-i}^1 = 0$, and is subsequently updated as follows:

$$g_{-i}^{t+1} \leftarrow \begin{cases} 0 & \text{if } a_{-i}^t = b_{-i}^t \text{ and } g_{-i}^t = 0 \\ \max\left(0, g_{-i}^t + r_{-i}(\mathbf{a}^t) - v_{-i} + \delta_t\right) & \text{otherwise} \end{cases} \qquad 16$$

where b_{-i}^t is player $-i$'s current action defined by the target solution χ and δ_t is a small nonnegative value. We use $\delta_t = 0.1$ if $g_{-i}^t = 0$ and $\delta_t = 0.0$ otherwise. When $g_{-i}^t > 0$ (i.e., the partner is guilty of deviating), the leader expert plays its attack policy π_i^{attack}. When $g_{-i}^t = 0$, the leader expert plays its portion of χ.

Supplementary Table 18 lists possible leader experts (for a particular set Ω) for a Prisoner's Dilemma. Depending on the implementation details (see Supplementary Table 20), some of these experts are not always included in Φ_i.

Follower Experts. Followers (also called expectant followers in the main paper) play a best response to the strategy they ascribe to their associate. S++ [1] includes three kinds of follower experts in the set Φ_i, each of which estimates its associate's strategy differently. The first expert models its associate using the fictitious-play assessment, conditioned on the last executed joint action. Let $\kappa_{-i}^t(\mathbf{a}, a_{-i})$ be the number of times that player $-i$ has played a_{-i} given the previous joint action $\mathbf{a}$ up to round t. Then, the estimated probability that player $-i$ plays a_{-i} is

$$\gamma_{-i}^t(\mathbf{a}, a_{-i}) = \frac{\kappa_{-i}^t(\mathbf{a}, a_{-i})}{\sum_{b_{-i}\in A_{-i}} \kappa_{-i}^t(\mathbf{a}, b_{-i})}. \qquad 17$$

The expert then computes the automaton that maximizes the player's future discounted reward (we used discount factor $\gamma = 0.95$) given γ_{-i}^t. We denote this expert as ϕ_i^*.

A second follower expert, denoted ϕ_i^{mm}, assumes that its associate is seeking to exploit it. Under this assumption, its best response is to play its maximin strategy, given by

$$\pi_i^{\text{mm}} = \arg\max_{\pi_i}\min_{\pi_{-i}} u_i(\pi_i, \pi_{-i}). \qquad 18$$

No.	Target solution (χ)	$v_1(\chi)$	Strategy
1	(none)	$\mu_1(br_1(\gamma_2^t), \gamma_2^t)$	$br_1(\gamma_2^t)$
2	(none)	$v_1^{\text{mm}} = 20$	π_1^{mm}
3	$<(a,d),(b,d)>$	80	
4	$<(a,d)>$	60	If $\mathbf{a}^{t-1}$ is in χ, play own portion of the next
5	$<(b,d),(a,c)>$	50	joint action in χ. Otherwise, randomly select a
6	$<(a,d),(b,c)>$	40	joint action from χ and play own portion of that
7	$<(a,d),(a,c)>$	30	joint action.
8	$<(b,c)>$	20	

Supplementary Table 19: Follower experts generated for player 1 in the Prisoner's Dilemma (Supplementary Table 4). $br_1(\gamma_2^t)$ denotes player 1's best response to the fictitious-play assessment γ_2^t. v_1^{mm} is player 1's maximin value. Adapted from Crandall [1].

Finally, the associate could also be implementing a leader strategy. Thus, a follower expert for each target solution $\chi \in \Omega$ is also included in Φ_i. Such experts always play their portion of the target solution χ. If a joint action in the target solution was not played in the previous round, these experts randomly select a joint action from the solution sequence and play player's corresponding part of the action.

Supplementary Table 19 lists eight potential follower experts that are computed for a Prisoner's Dilemma.

Selecting an Expert As described by Crandall [1], S++ selects which expert to follow in each round t using aspiration learning [75, 47, 54], along with a method for pruning the set Φ_i[10]. Aspiration learning has proven to be effective when learning to select among learning experts [81]. In aspiration learning, the player learns an achievable *aspiration level*, and simultaneously searches for an expert that produces payoffs that satisfy this threshold. Let α_i^t denote player i's aspiration level in round t. Initially, α_i^1 is initialized to a high value [82], and the set H_c, the set of joint actions played in the current cycle so far, is initialized to a random joint action. S++ then randomly selects some expert $\phi_i \in \Phi_i$, and follows ϕ_i until a particular joint action is repeated[11]. This is determined by evaluating whether the last joint-action played is already in H_c.

Once a cycle completes, α_i^t is updated as follows:

$$\alpha_i^t \leftarrow (\lambda_i)^{|H_c|}\alpha_i^{t-|H_c|} + (1 - (\lambda_i)^{|H_c|})\bar{r}_i^t, \quad 19$$

where $\lambda_i \in (0,1)$ is the learning rate and $\bar{r}_i^t$ is the average payoff obtained by player i in the cycle. After updating α_i^t, a new expert is selected as follows:

$$\phi_i^t \leftarrow \begin{cases} \phi_i^{(t-|H_c|)} & \text{with prob. } f(\alpha_i^t, \bar{r}_i^t) \\ \text{random}(\Phi_i'(t)) & \text{otherwise} \end{cases} \quad 20$$

Here, $\text{random}(\Phi_i'(t))$ denotes a random selection from the set $\Phi_i'(t) \subseteq \Phi_i'$ (described below), and $f(\alpha_i^t, \bar{r}_i^t)$ is the player's inertia, which specifies how likely the player is to select the expert that it played in the previous episode if $\bar{r}_i^t$ fails to meet α_i^t. If not, it randomly selects a new expert with probability $f(\alpha_i^t, \bar{r}_i^t)$. H_c is then reset to include only the last joint solution played, and a new cycle begins.

[10] S++ is essentially the same for multi-stage RSGs, the only difference being that the joint-action $\mathbf{a}^t$ must be replaced by an assessment of the joint strategy played throughout the round.

[11] For simplicity, we noted that S++ plays the selected expert for m rounds in Figure 3 of the main paper. This is technically correct, though the length m is variable as explained herein.

Algorithm 1 S++ for normal-form games.

Input:
Description of the game
Initialize:
Compute the set of experts Φ_i
Initialize the aspiration level α_i
$\mathbf{a}^0 \leftarrow \mathrm{random}(\mathrm{A})$
$H_c \leftarrow \{\mathbf{a}_0\}$, $t \leftarrow 1$, and $\bar{r}_i \leftarrow 0$
Select an initial expert $\phi_i^t \in \Phi_i$
repeat
  Select and execute action a_i^t (as dictated by ϕ_i^t)
  Observe $\mathbf{a}^t$ and r_i^t
  $\bar{r}_i \leftarrow \bar{r}_i + r_i^t$
  if $\mathbf{a}_t \in H_c$ **then**
    $\bar{r}_i = \bar{r}_i / |H_c|$
    Update α_i using Supplementary Equation 19
    Update each expert $\phi \in \Phi_i$ as necessary
    Select ϕ_i^{t+1} using Supplementary Equation 20
    $H_c \leftarrow \varnothing$ and $\bar{r}_i \leftarrow 0$
  else
    $\phi_i^{t+1} = \phi_i^t$
  end if
  $H_c \leftarrow H_c \cup \{\mathbf{a}_t\}$
  $t \leftarrow t + 1$
until Game Over

The set Φ_i can be rather large. Furthermore, some of the experts in Φ_i are unlikely to produce outcomes that meet the player's current aspiration level α_i^t. As such, the selection of these experts is unlikely to produce effective behavior and, thus, should be avoid. Thus, S++ prunes the set of selectable experts such that only experts that can potentially meet the aspiration level are considered. Formally, let $z_i^t(\phi)$ be the estimated potential (highest expected payoff) to be produced by expert $\phi \in \Phi_i$ at time t. Then, $\Phi_i'(t)$ is given by:

$$\Phi_i'(t) = \{\phi \in \Phi_i : z_i^t(\phi) \geq \alpha_i^t\}. \tag{21}$$

The potential $z_i^t(\phi)$ for each expert is computed as in the work by Crandall [1].

Pseudo-code for the complete S++ algorithm is given in Algorithm 1.

Parameter Choices S++ has several parameters that can be set to address various trade-offs. Implementation choices include (1) deciding how many leader and follower experts (i.e., the size of the set Ω) to include in the set Φ_i, (2) determining the inertia function (see Supplementary Equation 20), (3) determining which expert to follow first, (4) setting the initial aspiration level (α_i^1), and (5) setting the learning rate (λ_i). We briefly discuss these implementation choices.

- *Number of leader and follower experts.* Ideally, the number of target solutions should include a wide variety of different strategies, but be small enough that S++ can find a good expert within a small amount of time. Algorithm designers must also decide whether target solutions that are not enforceable when one's partner tends to forget events in the more distant past should be included in Φ_i.

- *Inertia function.* Inertia was previously considered for use in aspiration learning by Karandikar et al. [47]. The inertia function defines how likely the algorithm is to re-select the last expert when it does not produce sufficiently high payoffs in a cycle. In the initial implementation of S++ [1], the inertia function was:

$$f(\alpha_i^t, \bar{r}_i^t) = \min(1, \left(\bar{r}_i^t / \alpha_i^t\right)^{|H_c|}). \qquad 22$$

  However, it may be desirable to give the algorithm more memory of past events, thus making the player more likely to continue to follow an expert that has yielded better results in the past. To capture this idea, an alternative inertia function can be used, defined as follows:

$$\begin{aligned} \bar{v}_i^0 &= 0 \\ \bar{v}_i^t &= \beta \bar{r}_i^t + (1 - \beta)\bar{v}_i^{t-1} \\ \beta &= \max\left(0.2, \frac{1}{\kappa_i^t(\phi_i^t)}\right) \\ f(\alpha_i^t, \bar{r}_i^t) &= \min(1, \left(\max(\bar{r}_i^t, \bar{v}_i^t)/\alpha_i^t\right)^{|H_c|}). \end{aligned} \qquad 23$$

  We also set $f(\alpha_i^t, \bar{r}_i^t)$ to unity if both players always adhered to the target solution during the cycle.

- *Initial expert.* In initial implementations, S++, after taking a single random action, selected an expert using its standard selection. In some subsequent implementations, ϕ_i^* was automatically selected in the first round. This decision can potentially impact the *first impression* given by the algorithm to its associate.

- *Initial aspiration level.* In aspiration learning, higher initial aspiration levels tend to produce better long-term outcomes [54]. In initial implementations of S++, α_i^t was initialized to the value of the expert with the highest initial potential. In later studies, we set the initial aspiration level based on a fair, Pareto optimal, target solution. Higher initial aspirations make the algorithm more selective in early rounds, sometimes causing it to "insist" on unequal solutions in its favor. On the other hand, setting the aspiration level to what is expected in a fair solution tends to lead to faster convergence to cooperative solutions when one's partner is inclined to cooperate.

- *Learning rate.* A faster learning rate (i.e., lower λ_i) causes S++ to lower its aspiration level more quickly. This helps the algorithm to converge faster, but makes the algorithm more vulnerable to accepting lower payoffs than it otherwise might obtain [82]. Slower learning rates (i.e., high λ_i) cause S++ to adapt slower, but also make it more likely to find more profitable, Pareto optimal, outcomes.

Over the course of the studies that we have conducted, we have made small adjustments to these parameters as we have sought to develop an algorithm that consistently learns to cooperate with people (though we have observed that these changes often appear to have had little or no impact on the results in practice). Supplementary Table 20 summarizes the implementation choices used in each study.

S#

Despite the success of S++ when interacting with other algorithms (Supplementary Note 3), our results also show that S++ fails to consistently establishing cooperative long-term relationships with people (see Supplementary Notes 5 and 6). However, since humans appear to cooperate with each other much more

**The set Ω**	
Axelrod-style tournaments (Supp. Note 3)	As described in [1]
User Study 1 (Supp. Note 5)	As described in [1]
User Study 2 (Supp. Note 6)	As described in [2]
User Study 3 (Supp. Note 7)	As described in [2]; however, leader strategies were also constructed for target solutions that were unenforceable given an associate that only remembered a single joint action.
**Inertia function $f(\alpha_i^t, \bar{r}_i^t)$**	
Axelrod-style tournaments (Supp. Note 3)	Set according to Supplementary Equation 22
User Study 1 (Supp. Note 5)	Set according to Supplementary Equation 22
User Study 2 (Supp. Note 6)	Set according to Supplementary Equation 23
User Study 3 (Supp. Note 7)	Set according to Supplementary Equation 23
**Initial expert ϕ_i^1**	
Axelrod-style tournaments (Supp. Note 3)	Selects a random action in round 1, then selects randomly from $\Phi_i'(t)$
User Study 1 (Supp. Note 5)	Selects a random action in round 1, then selects randomly from $\Phi_i'(t)$
User Study 2 (Supp. Note 6)	ϕ_i^*
User Study 3 (Supp. Note 7)	ϕ_i^*
**Initial aspiration level α_i^1**	
Axelrod-style tournaments (Supp. Note 3)	$\alpha_i^1 = \max_{\phi \in \Phi_i} z_i^1(\phi)$
User Study 1 (Supp. Note 5)	$\alpha_i^1 = \max_{\phi \in \Phi_i} z_i^1(\phi)$
User Study 2 (Supp. Note 6)	$\alpha_i^1 = \max_{\phi \in \Phi_i} \min\left(u_i(\phi, br_{-i}(\phi)), u_{-i}(\phi, br_{-i}(\phi))\right)$
User Study 3 (Supp. Note 7)	$\alpha_i^1 = \max_{\phi \in \Phi_i} \min\left(u_i(\phi, br_{-i}(\phi)), u_{-i}(\phi, br_{-i}(\phi))\right)$
**Learning rate λ_i**	
Axelrod-style tournaments (Supp. Note 3)	$\lambda_i = 0.99$
User Study 1 (Supp. Note 5)	$\lambda_i = 1 - \frac{0.04}{\lvert\Phi_i'(t)\rvert}$
User Study 2 (Supp. Note 6)	$\lambda_i = 0.99$
User Study 3 (Supp. Note 7)	$\lambda_i = 0.99$

Supplementary Table 20: Parametric choices for S++ that were used in the four investigations presented in this paper.

Speech ID	Text	Speech ID	Text
0	Do as I say, or I'll punish you.	10	We can both do better than this.
1	I accept your last proposal.	11	Curse you.
2	I don't accept your proposal.	12	You betrayed me.
3	That's not fair.	13	You will pay for this!
4	I don't trust you.	14	In your face!
5	Excellent!	15	Let's always play <action pair>.
6	Sweet. We are getting rich.	16	This round, let's play <action pair>.
7	Give me another chance.	17	Don't play <action>.
8	Okay. I forgive you.	18	Let's alternate between <action pair> and <action pair>.
9	I'm changing my strategy.		

Supplementary Table 21: List of speech acts available to the players in the third user study.

consistently when they are able to signal to each other [4, 5], we consider adding to S++ the ability to communicate via cheap talk. That is, we use S++ to generate speech acts. We also use speech acts from S++'s partner to improve the way the algorithm selects experts. We call the resulting algorithm S# (pronounced 'S-sharp').

Most machine-learning algorithms have representations that are difficult to understand. Furthermore, these representations are very low-level, which makes them largely incapable of generating signals at a level that will be beneficial in interactions with people. On the other hand, S++ has a hierarchical internal representation which makes it possible to use the algorithm to generate and interpret cheap talk at a level conducive to interaction with a human partner. This capability means that S++ can potentially use cheap talk to help establish cooperative relationships with people.

The ability to engage in two-way cheap talk (so as to enhance one's own payoffs) requires two skills: (1) the ability to generate cheap talk and (2) the ability to act on the cheap talk of others. In this section, we describe how these abilities are addressed in S#.

Generating Cheap Talk The speech-generation system used by S# consists of three parts: (1) a language (i.e., a set of speech acts), (2) a method for selecting speech acts, and (3) a method for determining when to speak and when to remain silent. We define each in turn for S# in normal form games. A similar (but preliminary) method is defined for multi-stage RSGs in Supplementary Note 6.

A Set of Speech Acts. The language used by S# in the third user study (Supplementary Note 7) consisted of the set of phrases (i.e., *speech acts*) shown in Supplementary Table 21. These speech acts can be combined to propose plans, express sentiments, etc. The same set of phrases is used regardless of the game scenario, though the description of the game must include actions labels, as some speech acts utilize these action labels. We also limited participants to the same set of speech acts, which allows us to more easily determine how humans and machines use cheap talk in long-term repeated interactions. With the goal of not overwhelming participants, we kept the set of speech acts small, but sufficiently large to allow them to effectively plan and express a wide range of sentiments[12].

Though small, the speech acts shown in Supplementary Table 21 cover a range of different ideas a player may want to convey to its partner. For example, speech acts 15-18 provide means to propose desired

[12]A larger set of speech acts was used for the multi-stage games used in the second user study (Supplementary Note 6).

Symbol	Event descriptions
n	S# selected a new expert (distinct from the previous cycle) to follow.
c	An expert has been executed for a complete cycle.
x	S# accepts its partner's proposed plan of action.
y	S# rejects its partner's proposed plan of action because it does not trust its partner.
z	S# rejects its partner's proposed plan because it does not deem the proposal to be satisfactory.
b	S# believes the players can both receive higher payoffs than what they received in the previous cycle. This event is not generated if the new expert is MBRL.
s	The current expert is *satisfied* with last round's payoff.
f	The current expert *forgives* the other player.
d	The other player *defected* against S#.
g	The other player profited from its defection (and is *guilty*).
p	The expert has *punished* its guilty partner.
u	S# did not succeed in punishing its guilty partner.

Supplementary Table 22: A list of event symbols and event descriptions. The first six events correspond to master-level (i.e., expert selection) events of S++, while the last six events correspond to events relevant to individual experts.

solutions to an associate. Other speech acts allow the players to voice threats (speech acts 1 and 13), accept or reject proposals (1 and 2), explain decisions to reject proposals (3-4), manage the relationship (7-10), express pleasure (5-6), express displeasure (11-12), and to gloat (14). Combined together, these speech acts provide players with a surprisingly rich communication base.

In user study 3, when players were allowed to engage in cheap talk, each round of the repeated game began with the players independently selecting an ordered set of speech acts (from the prescribed list) that they wished to communicate to their partner. When both players had committed to a set of speech acts (and not before), the speech acts were communicated to the other player both visually and aurally (through a computer voice heard through headsets). The players then simultaneously selected an action. Once both players had selected an action, the joint outcome was displayed to the players, and a new round then began (starting with another round of cheap talk).

Speech Generation. S# uses the internal state of S++ and events in the game to determine which speech acts to voice. Thus, to generate speech acts, a finite state machine with output can be created in which the states are the states of S++, the events are outcomes that affect S#'s decision making, and the output of the finite state machine are speech acts. Because each of these items are defined for arbitrary repeated games, the speech-generation system is also game-independent. Thus, to create a game-independent speech system for S#, we need only define (a) the events of the game relevant to S++, (b) the underlying internal states of S++, and (c) the state transition function, which maps events and internal states to new internal states and speech output.

The events of the game that are relevant to S++ are listed in Supplementary Table 22. The first six events correspond to expert selection, while the last six events are events that impact the individual experts. Curiously, this set of events is rather small, which is due to the simple, hierarchical representation of S++. Though the underlying architecture of individual experts can be sophisticated, the dynamics of each expert embodies high-level principles that are understandable to people, and are governed by these high-level events.

Details of how these events are identified are as follows:

n This event is triggered whenever S# selects an expert that has a distinct offer (or target solution) from the expert it followed in a previous round.

c The current *cycle* begins at the end of the previous cycle. The current cycle continues as long as no joint action is played more than once in the cycle. When a joint action is repeated in a cycle, the cycle ends.

x S# selects an expert that implements its partners last proposed solution (which will be a sequence of joint actions).

y S# turns down a proposal made by its partner since its partner has not demonstrated enough reliability. Supplementary Equation 27 specifies how this is determined.

z S# turns down a proposal made by its partner since its expected payoff in the proposed solution is less than its current aspiration level α_i^t.

b There exists an expert with a target solution χ used by one of S#'s experts that produces higher expected payoffs to both players than the payoffs received by the players in the last round.

s S#'s payoff in round t (r_i^t) as at least as great as its current aspiration level α_i^t in the case that it is following an expert that is not a leader or follower expert. If the strategy is a leader expert or a follower expert, then r_i^t is at least as great as its payoff would have been if the current (in sequence) joint action of the target solution had been played.

f S#'s partner's guilt g_{-i}^t returns to 0 after Event g had been generated in a previous round.

d S#'s partner deviated from the target solution (χ) when S# is following a leader or follower expert. However, as a result of the deviation, r_{-i}^t was less than the payoff its partner would have received from the current (in sequence) joint action of the target solution (χ). Hence, its partner did not profit by deviating from playing its portion of χ.

g S#'s partner deviated from the target solution (χ) when S# is following a leader or follower expert. As a result of the deviation, r_{-i}^t is greater than the payoff its partner would have received in the current (in sequence) joint action of the target solution (χ). Hence, its partner has temporarily profited by deviating from playing its portion of χ.

p S# previously determined that its partner had benefitted by deviating from the target solution (χ) when it is following a leader expert (i.e., Event g has been generated, and Event f has not yet followed). Hence, its partner is guilty. Thus, S# seeks to punish its partner. This event is generated when its partner's payoff is less than the expected payoff it would have received when playing the target solution (i.e., $r_{-i}^t < v_{-i}(\chi)$).

u S# previously determined that its partner had benefitted by deviating from the target solution (χ) when it is following a leader expert (i.e., Event g has been generated, and Event f has not yet followed). Hence, its partner is guilty. Thus, S# seeks to punish its partner. This event is generated when the payoff obtained by its partner in the round was at least as high as the expected payoff it would have received when playing the target solution (i.e., $r_{-i}^t \geq v_{-i}(\chi)$).

The internal state of S++ is determined by (a) its aspiration level, (b) the currently selected expert, and (c) the state of the currently selected expert. Due to the hierarchical nature of S++, it is easiest to describe the internal states of S++ in a distributed fashion. Specifically, a finite-state machine (FSM) with output is created at both the master level and expert level. A separate FSM is formulated for each type of expert. These FSMs govern S#'s speech output only when a corresponding expert is selected. The master-level FSM governs speech output related to transitions between experts. After each transition in an expert-level FSM, control is transferred to the master-level FSM, which generates appropriate speech (if any) related to changes (or transitions) in the algorithms strategy. It then either returns control to the previous FSM or selects a new FSM (if it selects a new expert). The master and expert FSMs used by S# to produce speech acts are defined by the state transition tables shown in Supplementary Table 23.

As an example of how S#'s individual experts encode high-level strategies that can be easily articulated to people, consider a pure leader expert that targets the Nash bargaining solution of the game. This leader expert generates speech using the FSM depicted in Supplementary Table 23b. When initiated, the FSM outputs speech acts 15 and 0 – it proposes a fair plan (indicated by the corresponding joint action) and also issues the threat that its partner must conform with the plan or he/she/it will be punished. As the partner conforms with the proposed target solution thereafter, S# responds by saying '`Excellent`' for several rounds. If mutual cooperation (with the target solution) continues for five rounds, S# says: '`Sweet. We are getting rich`.' It then ceases talking as long as its partner continues to conform with the target solution. If its partner deviates from the target solution, S# responds by saying either '`Curse you`' or '`You betrayed me`.' If the partner benefited from the deviation, S# also adds the phrase: '`You will pay for this`,' and then proceeds to carry out the threat in subsequent rounds. Once it has sufficiently punished its partner, S# says '`I forgive you`,' and repeats the proposed plan and threat. In this way, it uses words to reinforce the understanding that its partner needs to conform with the target solution in order to obtain high payoffs.

When to Talk. While signaling (using, in this case, cheap talk) has great potential to increase cooperation, signaling intended actions exposes a player to the potential of being exploited. Furthermore, the *silent treatment* is often used by humans as a means of punishment and an expression of displeasure. For these two reasons, S# also chooses not to speak under certain circumstances. In this work, we offer a heuristic-based rule for deciding when to speak and when not to speak. This rule seems to work well in practice, though future work could potentially focus on developing alternative rules.

Formally, let b_i^t be the number of times up to round t that player i has been *betrayed* by player $-i$. That is, b_i^t is the number of times up to round t that player $-i$ did not play its part of the target solution proposed by player i. Then, the probability that player i voices speech acts in round t is given by

$$P_i^{SPEAK}(t) \leftarrow \begin{cases} 1 - 0.1 b_i^t & \text{if } 0 \leq b_i^t \leq 4 \\ \frac{2}{5 \cdot 2^{b_i^t - 3}} & \text{if } b_i^t > 4 \end{cases} \qquad 24$$

Note that the choice of whether or not to speak is made at the beginning of the cycle, and is held constant throughout the cycle.

Listening to Cheap Talk When players can communicate, S++ uses its partner's signals (in this case, speech acts) to alter which it expert it selects. This revised mechanism requires that we address two questions. First, how can S# use its partner's speech acts to improve which experts it selects? Second, when should S# listen to its partner.

Expert Selection. S#'s expert-selection mechanism differs in two ways from that of S++. First, S# uses the last proposal made by its partner to refine the set of experts it selects from at the end of each cycle.

(a) Master-level state transition table with output

State	State Transitions						Speech Acts (Output)					
	Events						Events					
	x, n	y	z	c	c, n	c, n, b	x, n	y	z	c	c, n	c, n, b
s_0	s_1	s_2	s_2	s_2	s_1	s_1	1	2+4	2+3	ε	9	9+10
s_1	Go to state s_0 of the FSM corresponding to the newly selected expert.						ε	ε	ε	ε	ε	ε
s_2	Return to the current state of the FSM corresponding to the currently selected expert.						ε	ε	ε	ε	ε	ε

(b) Pure leader expert

State	State Transitions							Speech Acts (Output)						
	Events							Events						
	NUL	d	g	p	u	f	s	NUL	d	g	p	u	f	s
s_0	s_1	–	–	–	–	–	–	15+0	–	–	–	–	–	–
s_1	–	s_2	s_7	–	–	–	s_2	–	$r(\{11,12\})$	$r(\{11,12\})$+13	–	–	–	5
s_2	–	s_2	s_7	–	–	–	s_3	–	$r(\{11,12\})$	$r(\{11,12\})$+13	–	–	–	5
s_3	–	s_2	s_7	–	–	–	s_4	–	$r(\{11,12\})$	$r(\{11,12\})$+13	–	–	–	5
s_4	–	s_2	s_7	–	–	–	s_5	–	$r(\{11,12\})$	$r(\{11,12\})$+13	–	–	–	ε
s_5	–	s_2	s_7	–	–	–	s_6	–	$r(\{11,12\})$	$r(\{11,12\})$+13	–	–	–	6
s_6	–	s_2	s_7	–	–	–	s_6	–	$r(\{11,12\})$	$r(\{11,12\})$+13	–	–	–	ε
s_7	–	–	–	s_7	s_7	s_8	–	–	–	–	14	ε	14+8	–
s_8	s_3	–	–	–	–	–	–	15+0	–	–	–	–	–	–

(c) Alternating leader expert

State	State Transitions							Speech Acts (Output)						
	Events							Events						
	NUL	d	g	p	u	f	s	NUL	d	g	p	u	f	s
s_0	s_1	–	–	–	–	–	–	18+16+0	–	–	–	–	–	–
s_1	–	s_2	s_7	–	–	–	s_2	–	$r(\{11,12\})$+16	$r(\{11,12\})$+13	–	–	–	5+16
s_2	–	s_2	s_7	–	–	–	s_3	–	$r(\{11,12\})$+16	$r(\{11,12\})$+13	–	–	–	5+16
s_3	–	s_2	s_7	–	–	–	s_4	–	$r(\{11,12\})$+16	$r(\{11,12\})$+13	–	–	–	5+16
s_4	–	s_2	s_7	–	–	–	s_5	–	$r(\{11,12\})$+16	$r(\{11,12\})$+13	–	–	–	16
s_5	–	s_2	s_7	–	–	–	s_6	–	$r(\{11,12\})$+16	$r(\{11,12\})$+13	–	–	–	6
s_6	–	s_2	s_7	–	–	–	s_6	–	$r(\{11,12\})$+16	$r(\{11,12\})$+13	–	–	–	ε
s_7	–	–	–	s_7	s_7	s_8	–	–	–	–	14	ε	14+8	–
s_8	s_3	–	–	–	–	–	–	18+16+0	–	–	–	–	–	–

(d) Pure follower expert

State	State Transitions			Speech Acts (Output)		
	Events			Events		
	NUL	d	s	NUL	d	s
s_0	s_1	–	–	15	–	–
s_1	–	s_2	s_2	–	$r(\{11,12\})$	5
s_2	–	s_2	s_3	–	$r(\{11,12\})$	5
s_3	–	s_2	s_4	–	$r(\{11,12\})$	5
s_4	–	s_2	s_5	–	$r(\{11,12\})$	ε
s_5	–	s_2	s_6	–	$r(\{11,12\})$	6
s_6	–	s_2	s_6	–	$r(\{11,12\})$	ε

(e) Alternating follower expert

State	State Transitions			Speech Acts (Output)		
	Events			Events		
	NUL	d	s	NUL	d	s
s_0	s_1	–	–	18+16	–	–
s_1	–	s_2	s_2	–	$r(\{11,12\})$+16	5+16
s_2	–	s_2	s_3	–	$r(\{11,12\})$+16	5+16
s_3	–	s_2	s_4	–	$r(\{11,12\})$+16	5+16
s_4	–	s_2	s_5	–	$r(\{11,12\})$+16	16
s_5	–	s_2	s_6	–	$r(\{11,12\})$+16	6
s_6	–	s_2	s_6	–	$r(\{11,12\})$+16	ε

(f) FSM for the MBRL expert.

State	State Transitions		Speech Acts (Output)	
	Events		Events	
	¬s	s	¬s	s
s_0	s_0	s_0	–	5

(g) FSM for the maxmin expert.

State	State Transitions			Speech Acts (Output)		
	Events			Events		
	NUL	¬s	s	NUL	¬s	s
s_0	s_1	–	–	4	–	–
s_1	–	s_1	s_1	–	–	–

Supplementary Table 23: Finite state machines (FSMs) with output define the speech acts generated by S#. The master-level FSM (a) is executed beginning in state s_0 after each transition in an expert-level FSM (b–g). The master-level FSM returns control to an expert-level FSM once a terminal state is reached (s_1 and s_2). If state s_1 is reached in the master-level FSM, control over speech output is returned to the FSM corresponding to the currently selected expert (in its current state). If state s_2 is reached, control over speech generation is moved to the FSM corresponding to the newly selected expert, beginning in state s_0. The symbol '–' as output indicates the state-event combination is not possible. ε indicates the empty string. Numbers in the output portion of the FSM indicate numbers of the speech acts (Supplementary Table 21) generated by S#; '+' indicates concatenation of speech acts. NUL indicates an automatic transition; no event input is considered. Unspecified events do not produce any transitions in the FSM.

Algorithm 2 S# for normal-form games.

Input:
Description of the game
Initialize:
Compute the set of experts Φ_i
Initialize the aspiration level α_i
$\mathbf{a}^0 \leftarrow \mathrm{random}(\mathrm{A})$
$H_c \leftarrow \{\mathbf{a}_0\}$, $t \leftarrow 1$, and $\bar{r}_i \leftarrow 0$
Select an initial expert $\phi_i^t \in \Phi_i$
repeat
Produce speech acts as dictated by the FSMs
Observe player $-i$'s speech acts and compute $\Psi_i(t)$
if $\Psi_i(t) \neq \Psi_i(t-1)$ and $\Phi_i' \cap \Psi_i(t) \not\equiv \varnothing$ and $(n_t < P_i^{LISTEN}(t))$ **then**
$\bar{r}_i = \bar{r}_i / |H_c|$
Update α_i using Supplementary Equation 19
Select ϕ_i^t using Supplementary Equation 25
$H_c \leftarrow \{\mathbf{a}_{t-1}\}$ and $\bar{r}_i \leftarrow 0$
end if
Select and execute action a_i^t (as dictated by ϕ_i^t)
Observe $\mathbf{a}^t$ and r_i^t
$\bar{r}_i \leftarrow \bar{r}_i + r_i^t$
if $\mathbf{a}_t \in H_c$ **then**
$\bar{r}_i = \bar{r}_i / |H_c|$
Update α_i using Supplementary Equation 19
Update each expert $\phi \in \Phi_i$ as necessary
Select ϕ_i^{t+1} using Supplementary Equation 25
$H_c \leftarrow \varnothing$ and $\bar{r}_i \leftarrow 0$
else
$\phi_i^{t+1} = \phi_i^t$
end if
$H_c \leftarrow H_c \cup \{\mathbf{a}_t\}$
$t \leftarrow t + 1$
until Game Over

Second, S# considers switching which expert it follows mid-cycle if its partner proposes a desirable plan. The resulting algorithm is summarized in Algorithm 2. We discuss these two modifications in turn.

S# uses the last proposed plan made by its associate to modify its expert-selection mechanism. Rather than selecting experts using Supplementary Equation 20, this modified version selects experts using the following selection rule:

$$\phi_i^t \leftarrow \begin{cases} \phi_i^{(t-|H_c|)} & \text{with prob. } f(\alpha_i^t, \bar{r}_i^t) \\ \mathrm{random}(\Phi_i''(t)) & \text{otherwise} \end{cases} \qquad 25$$

This selection rule is identical to Supplementary Equation 20 except that random selections are made from the set $\Phi_i''(t) \subseteq \Phi_i'(t)$ rather than $\Phi_i'(t)$.

In normal-form games[13], proposed plans involve the specification of a solution, which consists of either

[13]Multi-stage RSGs are slightly more difficult, though we describe a solution in Supplementary Note 6 that worked well in practice for the two distinct games tested.

a joint action or sequence of joint actions (see Supplementary Table 21, speech IDs 15, 16, and 18[14]). S# compares this solution with the target solutions of each of its leader and follower experts. Let $\Psi_i(t)$ denotes the set of experts that have target solutions that are *congruent* (i.e., in the context of normal-form games, *equivalent*) with its partner's last proposed solution.

Given $\Psi_i(t)$, the set $\Phi''_i(t)$ is derived as follows:

$$\Phi''_i(t) \leftarrow \begin{cases} \Phi'_i \cap \Psi_i(t) & \text{if } (\Phi'_i \cap \Psi_i(t) \neq \emptyset) \text{ and } (n_t < P_i^{LISTEN}(t)) \\ \Phi'_i(t) & \text{otherwise} \end{cases} \quad (26)$$

where n_t is a randomly selected number from the range $[0, 1]$. In words, if the set formed by the intersection of the set of potentially satisficing experts (Φ'_i) and the set of experts congruent with player i's last proposal ($\Psi_i(t)$) is not empty, then with probability $P_i^{LISTEN}(t)$ (the probability that player i will listen to a satisfactory proposal made by player $-i$), the algorithm selects an expert from the intersection of those two sets. Otherwise, the algorithm selects experts from the set Φ'_i (as with S++).

The second difference between the expert-selection mechanisms of S# and S++ is that S# considers switching its expert in mid-cycle if its partner makes a new proposal. That is, if the new proposal made by its partner is congruent with some expert $\phi \in \Phi'_i$, then S# immediately switches its expert with probability $P_i^{LISTEN}(t)$. This alteration is reflected in the first *if*-block of Algorithm 2.

When to Listen. The probability $P_i^{LISTEN}(t)$ specifies the probability that player i will listen to a desirable proposal made by player $-i$. As in the case of speech generation, the act of listening to an associate opens one to the possibility of being exploited. For example, in the prisoner's dilemma, an associate could continually propose that both players cooperate, a proposal S# would continually accept and act on when $\alpha_i^t \leq 60$). However, if the partner did not follow through with its proposal, it could potentially exploit S# to some degree (for a period of time).

To avoid this, S# listens to its partner less frequently the more the partner fails to follow through with its own proposals. Let d_i^t be the number of times up to round t that player i has been *betrayed* while *listening* to player $-i$. Player i is considered to be *listening* to player $-i$ if the current expert ϕ_i^t, which was selected in round τ, was in the set $\Psi_i(\tau)$. Then, $P_i^{LISTEN}(t)$ is given by

$$P_i^{LISTEN}(t) \leftarrow \begin{cases} 1 & \text{if } d_i^t = 0 \\ 1 - \frac{2^{d_i^t}}{20} & \text{if } 1 \leq d_i^t \leq 3 \\ \frac{2}{5 \cdot 2^{d_i^t - 4}} & \text{if } d_i^t \geq 4 \end{cases} \quad (27)$$

We note that the equations specifying when to talk (Supplementary Equation 24) and when to listen (Supplementary Equation 27) are heuristics derived from experimentation. While these heuristics worked well in practice, future work could explore these functions more carefully to further improve the way that S# utilizes cheap talk.

Performance Properties

S# differs from S++ in two ways: (1) it produces cheap talk (i.e., it talks) and (2) it responds to the cheap talk of others (i.e., it listens). In the second and third user studies reported in this paper (see Supplementary Notes 6 and 7, respectively), we evaluate the ability of S#- (an early version of S# that talked but did not

[14] In particular, phrases 15 and 18 are used to specify a particular target solutions. Phrase 16 could also be used to specify a target solution, though it may also be used to specify the next solution in the sequence. In this work, we assume that phrase 16 specifies a target solution only if the solution specified in this speech act is not part of the last target solution proposed by the partner.

listen) and S# to forge successful relationships in repeated games with people. Before reporting these user studies, we discuss how talking and listening impacts S#'s ability to learn to forge successful relationships. We focus on two topics. First, we discuss how talking and listening impacts S#'s ability to avoid being exploited, a property that has been called *security*. Second, we evaluate how S#'s ability to talk and listen impacts its ability to cooperate when paired with a partner that follows S#.

Security Security refers to the guarantee that an algorithm will not, in expectation, achieve an average payoff more than ε below its maximin value v_i^{mm}, regardless of the behavior of its associate. S++ is guaranteed to be secure [1]. Since S# uses S++, it also has the potential to be secure. However, because S# voices its strategy and listens to plans proposed by its partner, it potentially exposes itself to exploitation. Fortunately, Eqs. (24) and (27), which define the variables P_i^{SPEAK} and P_i^{LISTEN}, ensure that the probability of exploitation due to voicing and to listening to cheap talk becomes vanishingly small over time. Thus, like S++, S# is secure as $t \to \infty$ when the payoffs in the game are finite.

The Impact of Talking and Listening We gave S# the ability to generate cheap talk and respond to the cheap talk of others in hopes of helping it to forge more successful relationships. To begin to analyze the importance of these two mechanisms, we consider a simulation study in which we compare the performance of three different algorithms when they are paired with S# in repeated games. These algorithms are:

1. *S++ (No communication via cheap talk).* S++ neither talks nor listens to S#. As such, the two players do not communicate via cheap talk.

2. *S#- (One-way communication via cheap talk).* S#- is identical to S#, except that it either does not talk or does not listen. Thus, it is half way between S++ and S#. When S#- is paired with S#, communication via cheap talk flows in only a single direction. In the case that S#- just talks, communication via cheap talk flows from S#- to S#. In the case that S#- just listens, it flows from S# to S#-. When paired with S#, these two conditions are identical (effectually, one player listens while the other talks).

3. *S# (Two-way communication via cheap talk).* When paired with another player that follows S#, communication via cheap talk flows in both directions. Both players both listen to and talk to their partner using cheap talk.

Supplementary Figure 12(top) shows the results of this comparison with respect to the ability of the algorithms to establish mutually cooperative relationships in three different games. Both talking and listening are important to S#'s success. In all three games, S#- forged mutually cooperative relationships more quickly than S++. However, S# did so more quickly than S#- in each game. These observations are supported by statistical analysis. For each game, we tested a generalized linear mixed model of mutual cooperation (binomial family) as a function of communication type, round number, and simulation session. We used backward coding difference for the contrasts of the communication variable, so that two-way communication was compared to one-way communication, which was in turn compared to no communication. Results were the same across all games: mutual cooperation increased with time (all $z > 28$, all $p <$ 10e-16), two-way communication was an improvement over one-way communication (all $z > 5.8$, all $p <$ 10e-8), and one-way communication was an improvement over no communication (all $z > 10.4$, all $p <$ 10e-16). The ability to quickly establish mutually cooperative solutions translates directly into higher payoffs (Supplementary Figure 12(bottom)).

These results confirm our observations in carrying out user studies 2 and 3, which are reported in Supplementary Notes 6 and 7. Initially, for user study 2, we created and evaluated S#-, which generated cheap talk,

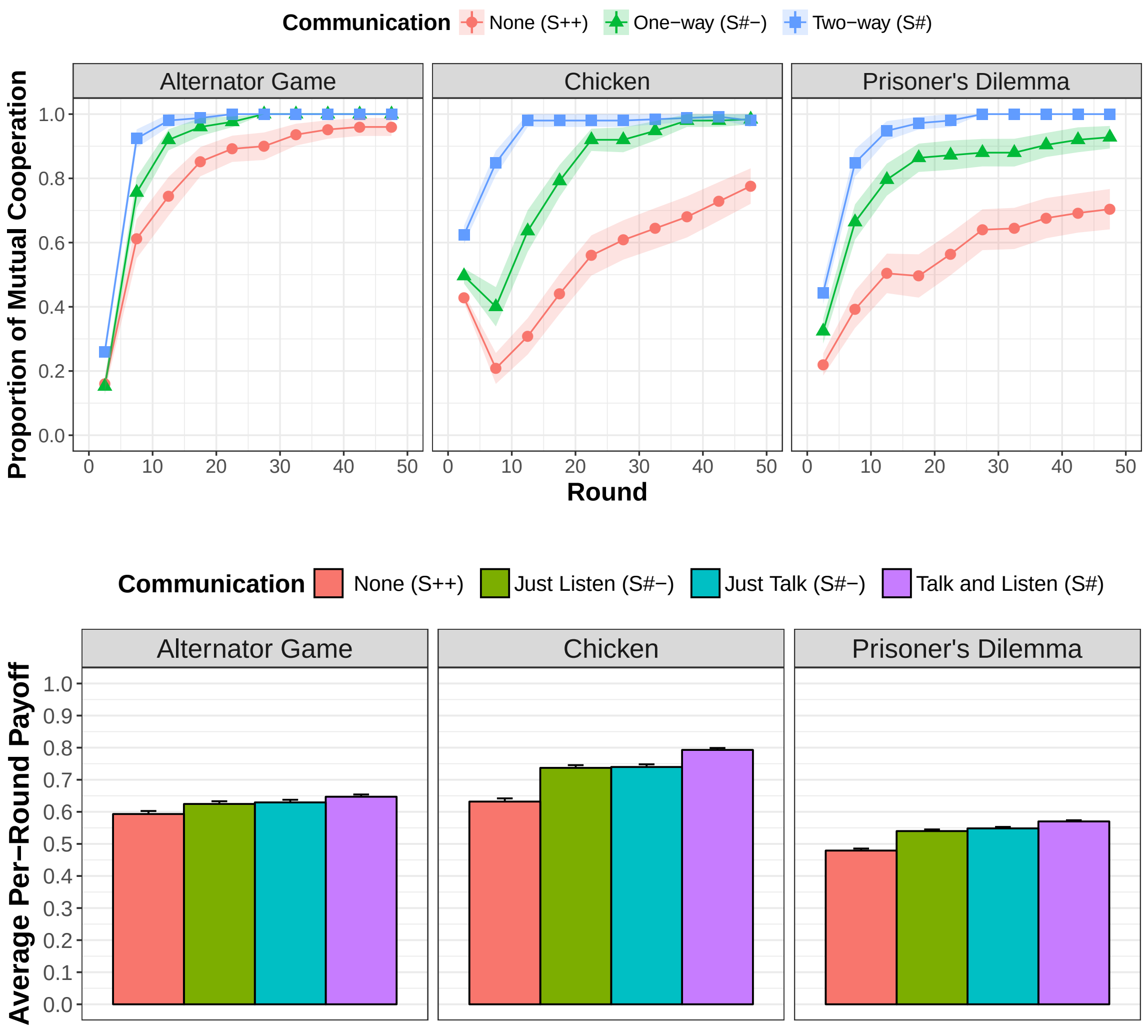

Supplementary Figure 12: The impact of generating and responding to cheap talk in three different repeated games. In this simulation study, three different algorithms were paired with S#: S++ (which does not talk or listen), S#- (which just talks or just listens but not both), and S# (which both talks and listens). Parameters for the algorithms were set as in User Study 3 (see Supplementary Table 20). (top) The proportion of mutual cooperation forged by the pairings. Results are an average of 50 trials for each game and condition. Points represent the mean payoff over all trials (averaged over 5-round increments), while error ribbons show the standard error of the mean. (bottom) Average per-round payoffs obtained by the players over all 50 rounds of the game. The bars show the mean over all trials, while error bars show the standard error of the mean.

but did not respond to its partner's cheap talk. While mutual cooperation tended to emerge more frequently in interactions between S#- and people than in interactions between S++ and people, we observed that it did not emerge as consistently or as quickly as we would have liked. Study participants often became frustrated as they waited for the machine to propose a solution they could agree to. Thus, we added the ability to also respond to a partner's proposal, thus forming S#. Results described in Supplementary Note 7 (the third user study) show that people established cooperative relationships with S# as consistently as they did with other people.

Supplementary Note 5

User Study – Human vs. Machine 1

In this paper, we are interested in developing algorithms that can effectively learn to interact with people in arbitrary repeated games. Despite the large amount of research devoted to algorithmic development in the area of repeated games, very little work has addressed how well these algorithms are capable of playing repeated general-sum games with people, though game-specific research has been done in one-shot games [83]. However, with the growing presence of autonomous machines in society that interact repeatedly with us (i.e., repeated games), developing algorithms that establish cooperative relationships with people, and do so in arbitrary scenarios under the threat of being exploited, is critical.

In the analysis performed in Supplementary Note 3, we observed that S++ [1], a recently developed machine-learning algorithm for repeated games, was a top-performing algorithm among 25 representative algorithms. The results of that analysis showed that S++ was able to learn to interact effectively with other AI algorithms across an extensive set of 2x2 games. Equally importantly, the results showed that S++ learns within time-scales that would support interactions with people.

In this section, we describe a user study designed to investigate whether S++ is able to learn to establish and maintain cooperative relationships with people in a variety of scenarios. In this study, we paired S++ with people in four repeated normal-form games (Prisoner's Dilemma, Chicken, Chaos, and Shapley's Game). As part of this analysis, we benchmark the performance of S++ in these games with the performance of people.

Experimental Setup

We describe, in turn, the experimental protocol carried out in the user study, the games used in the study, and the user interface through which participants played the games.

Protocol In the user study, we paired study participants with other Humans, S++, MBRL-1, and BULLY in four repeated normal-form games (Prisoner's Dilemma, Chicken, Chaos, and Shapley's Game). Each of the 58 participants played each of the four games in a random order. Over these four games, each participant was paired with each of the four partner types in a random order[15]. Assignment of participants to orders was pre-determined by a schedule based on subject ID number, which as assigned based on the order subjects arrived to the study. The participants were volunteers from the Masdar Institute community, comprised primarily of graduate students and postdoctoral associates. The study was approved by Masdar Institute's Human Subjects Research Ethics Committee. Informed consent was obtained from each participant.

Groups of between four and eight (typically six) people participated in the study at a time in a computer lab at Masdar Institute. The following procedure was followed for each group of participants:

1. Each participant was assigned a random ID, which defined the order that the participant played the four games as well as the partners they were paired with.

[15] A number of errors occurred in the administration of the user study, such that in a few instances subjects played the same game twice or were paired with a different player type twice. Additionally, several data samples were lost due to system malfunctions during the user study. Given that this was an exploratory study (followed up be subsequent studies), we do not feel that these errors compromise the integrity of our overall findings.

2. Each participant was trained on how to play the game using a web interface. Before proceeding to the first game, each participant was required to correctly answer several questions that tested their knowledge of how the game was played.

3. Repeat for each of the four games:

   (a) Each participant played a repeated game lasting 50 or more rounds. Participants were not told in advance the length of the game (i.e., number of rounds). To mitigate end-game effects, the length of each game was randomized between 50 and 57 rounds (thus, we only report on results over the first 50 rounds). Participants were also not told the identity of their partner, nor were they allowed to communicate with the other participants.

   (b) Each participant completed a brief post-game questionnaire.

To incentivize participants to try to maximize their own payoffs in the game, subjects were paid based on their performance. Each participant received approximately \$5 for participating in the study. A participant could earn an additional \$10 based on their performance. Each participant's performance-based compensation was directly proportional to the amount of points he or she received in each repeated game. The amount of money a participant had earned in a game was displayed on the game interface in local currency.

In an effort to hide the identity of the players from each other, each round of each game lasted a fixed amount of time (in seconds). Participants were allotted a fixed amount of time to select an action, after which the results of the round were displayed. If a participant failed to select an action within the allotted time, a random action was automatically selected in their behalf. To encourage participants to always select an action, participants were not paid for points received in rounds in which they failed to select an action.

This experimental design allowed us to collect on the order of 58 samples each for Human-S++, Human-MBRL-1, and Human-Human pairings, with samples divided cross the four different games[16]. Given that this was an initial exploratory study, this sample size was chosen to adequately limit the cost of running the user studies while providing sufficient samples to determine substantial differences in the algorithms. Additionally, since a separate instance of S++ and MBRL-1 was used for each interaction, the interactions between algorithms were independent from each other. Thus, the number of trials used for agent-agent pairings was not limited by the number of participants in the study. Thus, we conducted 100 different simulations of each agent-agent pairing in each game.

Games The payoff matrices of the four repeated, normal-form, general-sum games used in the study are given in Supplementary Table 24. Each of these games represents a different scenario in which cooperation and compromise are difficult to achieve against unknown associates. Furthermore, each game has an infinite number of Nash equilibria (NE) of the repeated game when the game is repeated after each round with sufficiently high probability.

The first game is the Prisoners' Dilemma (PD). In the PD, defection (d) is the dominant strategy for both players. However, this joint action is Pareto dominated by the solution (c, c) which leads to a payoff of 0.6 for both players. Both players can increase their payoffs by convincing the other player to cooperate. The dilemma or challenge arises from the difficulty of convincing a rational player that it is better to play a dominated strategy. The pair (c, d) means the column player gets the temptation reward of 1.0 for defecting against its partner, while the row player gets the lowest payoff of 0. Similarly, (d, c) means the column player gets the lowest payoff of 0 compared to the defecting partner's payoff of 1.0.

[16]Again, errors in administering the study along with system malfunctions that required us to exclude some samples, resulted in slightly different numbers for each condition.

(a) Prisoners' Dilemma (PD)

	c	d
**c**	0.60, 0.60	0.00, 1.00
**d**	1.00, 0.00	0.20, 0.20

(b) Chicken

	a	b
**a**	0.84, 0.84	0.33, 1.00
**b**	1.00, 0.33	0.00, 0.00

(c) Shapley's Game

	a	b	c
**a**	0, 0	1, 0	0, 1
**b**	0, 1	0, 0	1, 0
**c**	1, 0	0, 1	0, 0

(d) Chaos Game

	a	b	c
**a**	0.46, 0.67	0.24, 0.06	1.00, 0.00
**b**	0.00, 0.37	0.37, 0.07	0.01, 0.53
**c**	0.14, 0.69	0.20, 1.00	0.71, 0.90

Supplementary Table 24: Payoff matrices of four games considered in our study. In each cell, the row player's payoff is listed first, followed by the column player's payoff.

The second game is *Chicken*, also known as the *hawk-dove* game in evolutionary game theory [84]. Chicken models a situation in which players are in equilibrium if one player chooses to concede (the 'dove') by playing action a, while the other chooses to aggress (the 'hawk'; action b). However, since the payoff is higher for aggressing, each player is demotivated to be the one to concede.

Shapley's game [85], a variation on rock, paper, scissors, is of interest because most learning algorithms do not converge when they play it [8]. Moreover, it does not have a pure one-shot NE, but rather a unique one-shot mixed strategy NE in which all players play randomly. This NE yields a payoff of $1/3$ to each player. However, in repeated interactions, both players can do better by alternating between getting a payoff of 0 and 1, which results in a Pareto-optimal (and fair) outcome in which both players get an average payoff of 0.5.

The *Chaos* game was created by the authors such that the 'right' action is not immediately obvious. Like the PD, the game has three Pareto-optimal solutions and a single one-shot NE solution in which both players play a. However, the game is less structured than the PD and has a larger strategy space, which seemingly would make cooperation and compromise more difficult to learn against arbitrary associates.

Because the Chaos game is not symmetric, this game must receive special treatment in the analysis. When participants were paired with either S++ or MBRL-1, the participants were always given the role of the column player, who has a stronger position in this game. Thus, to allow us to make comparisons between players in this game, as well as to allow us to make comparisons across games, we used standardized payoffs (i.e., specifically the standardized z-scores across each game) when analyzing payoffs received.

Algorithms In this study, we analyze the performance of the three[17] different player types: humans (the study participants), S++, and a model-based reinforcement learning algorithm (MBRL-1; see Supplementary Table 6 in Supplementary Note 3 for implementation details). MBRL-1 represents traditional learning algorithms for repeated games, and hence makes a good additional point of comparison that shows differences between traditional artificial intelligence and more recent advances (represented by S++). Parameter values for the version of S++ used in this user study are given in Supplementary Table 20 in Supplementary Note 4.

[17]For simplicity of analysis, we omit games involving the algorithm BULLY. As in the simulation studies presented in Supplementary Note 3, BULLY sometimes performed very well, and other times performed very poorly. BULLY does not consistently cooperate across many games and associates, particularly against humans.

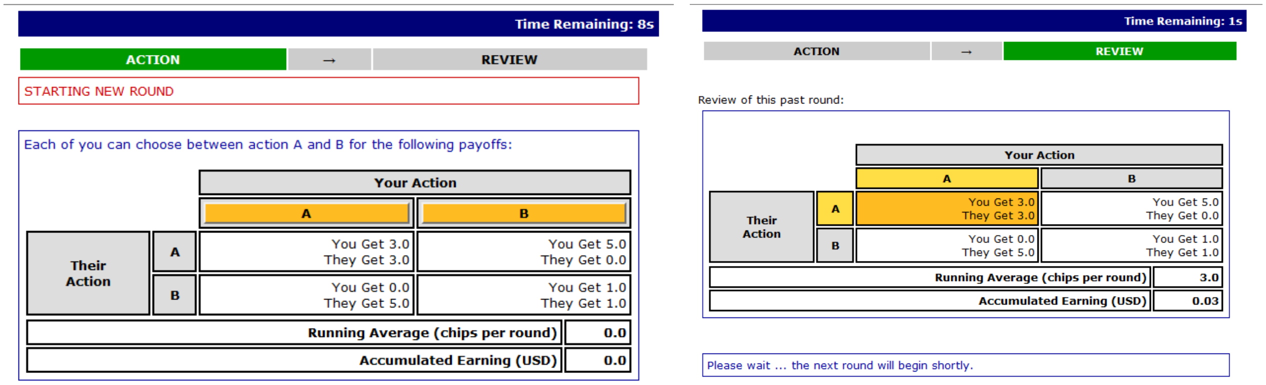

(a) (b)

Supplementary Figure 13: Web-based GUI used by participants to play the games in the first user study. **(a)** The action-selection display used by the participant to select an action. **(b)** The round-review display through which the participant observed the results of a round.

The three player types are paired to form six different pairings: MBRL-1/Human, Human/Human, S++/Human, S++/S++, S++/MBRL-1, and MBRL-1/MBRL-1. In this study, we are primarily concerned with comparisons between how well each of the player types interact with people (hence, MBRL-1/Human, Human/Human, and S++/Human pairings). However, the other three (agent-agent) pairings offer insights into the personalities of the algorithms and what they are capable of achieving.

User Interface Each participant played the games on a desktop computer in a computer lab using the web-based graphical user interface (GUI) shown in Supplementary Figure 13. In each round, the complete payoff matrix was displayed, along with a button corresponding to each of the participant's actions (Supplementary Figure 13a). A timer was also displayed, indicating the time remaining in the current round (i.e., the deadline for selecting an action). Once the round was completed, the results of the round, including the actions selected by both players and the resulting joint payoff, were displayed on the screen for a fixed amount of time (Supplementary Figure 13b), after which a new round began.

The average per-round payoff of the participant in the current game, along with the participant's current earnings, were displayed on the game interface. The average per-round payoff of the participant's partner was not displayed, however. This was done to emphasize that the participant's goal was to maximize its own payoffs rather than to outscore his/her partner.

Outcomes

We focus on two aspects of the results in this study. First, we study the ability of AI algorithms to learn to cooperate with people. Hence, our primary metric is the proportion of rounds that both players cooperated with each other (i.e., mutual cooperation). However, given that self-interested individuals care only about cooperation if it leads to higher payoffs, we also analyze the payoffs received by the players.

Mutual Cooperation The proportion of mutual cooperation observed for each pairing in each game is shown in Supplementary Figure 14. The figure shows several important results. First, S++/S++ pairings had the highest levels of mutual cooperation by the end of each game. The success of S++ is confirmed

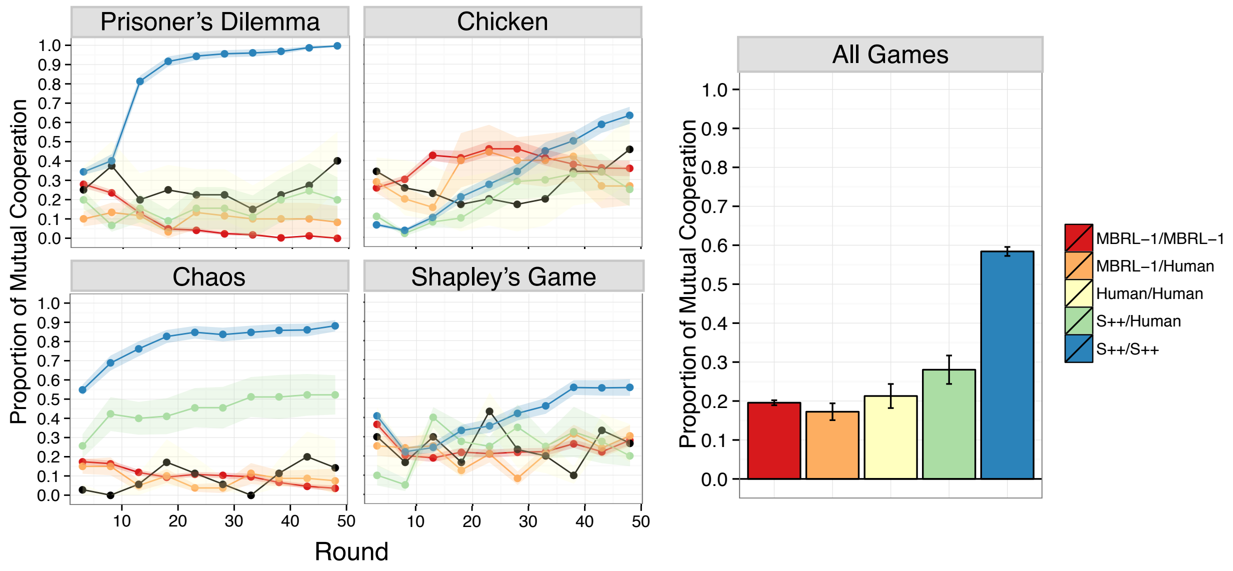

Supplementary Figure 14: Proportion of mutual cooperation in individual games (left) and across all four games and rounds (right). Error bands/bars show the standard error on the mean. In the individual games, results are shown as the average of five-round increments.

by a negative binomial regression in which the number of cooperative rounds within each pairs was the dependent variable, and in which Game, Pairing, and their interaction were predictors (because sample variance was largely greater than sample mean in most cells, a Poisson regression was not appropriate). This analysis showed that S++/S++ pairings had significantly more cooperative rounds than any other pairings (all $z < -4$, all $p < 0.001$). Additionally, S++/Human pairings had more cooperative rounds than Human/Human pairings, MBRL-1/Human pairings, and pure MBRL-1 pairings (all $z < -5$, all $p < 0.001$). The analysis also detected multiple interaction effects across games and pairings. These interaction effects can be visually assessed in the figure. For example, in both the Prisoner's Dilemma and in Chaos, S++/S++ pairings had the highest proportion of mutual cooperation throughout all rounds, whereas S++/S++ pairings only obtained the highest levels of mutual cooperation in Chicken and Shapley's Game after 30 to 40 rounds of interaction (S++/S++ pairings almost always eventually learn to play the mutual cooperative solutions in these games given sufficient rounds of interaction). Altogether, these results continue to demonstrate the potential of S++ to learn to cooperate.

The superiority of S++/S++ pairings (with respect to learning mutually cooperative solutions) can be traced to S++'s ability to improve its behavior as it gains more experience interacting with a particular partner. While the proportion of mutual cooperation in S++/S++ pairings increased substantially from the first ten rounds to the last ten rounds (Supplementary Figure 15), the same cannot be said of other pairings (at least not to the same extent). This result is confirmed by nonparametric Wilcoxon tests comparing the number of mutually cooperative rounds that each pairing achieved early (first ten rounds) and late (last ten rounds) in an interaction. S++/S++ and Human/S++ pairings significantly improved ($p < 0.001$ in both cases), Human/Human and MBRL-1/Human pairings did not significantly improve or disprove ($p = 0.46$ and $p = 0.16$, respectively), and pure MBRL-1 pairings even decreased in cooperation ($p < 0.001$). In summary, S++ is able to use its experience to improve its rate of cooperation when paired with humans and with copies of itself.

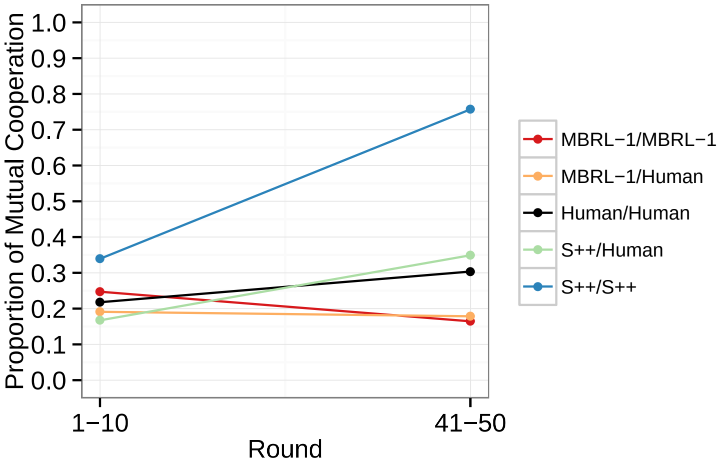

Supplementary Figure 15: Average proportion of mutual cooperation across all games over the first and last ten rounds, respectively. Only pairings involving S++ had statistically significant increases in performance between these time intervals.

Second, Humans and MBRL-1 failed to consistently cooperate with any of the three player types. Pairs of people played the mutually cooperative solution in only about 20% of rounds across all four games. Mutual cooperation among human pairings never reached 50% in any game over any five-round increment. Proportions of mutual cooperation obtained by MBRL-1 were similarly low.

Thus, S++ learns to cooperate with people as well as people do. However, this achievement was more of a function of the inability of people to consistently cooperate with each other than S++ being able to consistently elicit cooperative behavior from people.

Payoffs Received In this user study, the ability to establish cooperation relationships tended to translate directly into higher payoffs. Among the five pairings evaluated in the previous subsection (MBRL-1/MBRL-1, MBRL-1/Human, Human/Human, S++/Human, and S++/S++), the correlation between payoffs received (i.e., the standardized z-score in each game) and the proportion of mutual cooperation was $r(1876) = 0.800$, $p < 0.001$. This strong correlation indicates that, when associating with capable partners, the ability to achieve high payoffs is typically tied to one's ability to establish cooperative relationships.

Supplementary Figure 16 shows the average standardized payoffs obtained by each player type when paired with each of the three partner types, across all four games. The figure shows several important trends. First, S++/S++ pairings, which had by far the highest levels of mutual cooperation, also achieved the highest average payoffs. Second, for each partner type, S++ achieved similar or higher payoffs as people. This further confirms that S++ is as capable as humans in establishing effective relationships in these games. Finally, there is a trend showing that MBRL-1 consistently achieved the lowest payoffs of the three player types.

Reflections

This user study had several important results:

1. S++ is much more capable in self play than both people and MBRL-1. Self play is an important aspect of evolutionary robustness [6]. It also helps to demonstrate the potential of S++ to cooperate

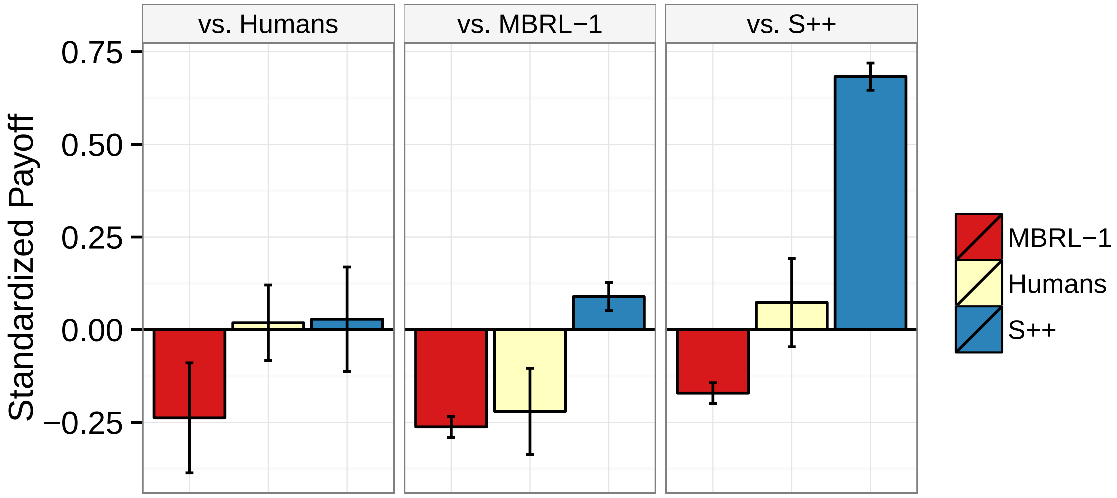

Supplementary Figure 16: Average standardized payoffs across all four games when paired with each player type (Human, MBRL-1, S++).

with other players.

2. S++ appears to be much more effective at learning from experience than Humans or MBRL-1. In self play, MBRL-1 even showed statistically significant decreases in mutual cooperation over the four games considered. Meanwhile, S++ had statistically significant increases in mutual cooperation as the rounds of the game increased when paired with both a copy of itself and when paired with people. There was not a statistically significant increase in mutual cooperation among human pairings over time.

3. The performance of S++ was as good as or better than humans when paired with all three player types across the four games considered. Despite this success, the proportion of mutual cooperation between S++ and people was quite low, as was the case for Human/Human pairings. Thus, S++, in its current form, is unable to consistently elicit cooperative behavior from people.

In short, S++, in its current form, does not fully meet our objective of a learning algorithm that consistently learns to cooperate with people. Given the importance of this outcome in establishing effective human-machine societies, we extended S++ with the ability to communicate with people using cheap talk. The resulting algorithm, dubbed S# (pronounced 'S-sharp'), is analyzed in the two user studies presented in the next two Supplementary Notes.

Supplementary Note 6

User Study – Human vs. Machine 2

Despite S++'s ability to establish successful relationships with other algorithms, the results of the first user study (Supplementary Note 5) show that S++ was unable to consistently elicit cooperative behavior from people across four repeated normal-form games. It did, however, cooperate with people as much as people did, as people also failed to consistently cooperate with each other in these games.

Past research has shown that people cooperate with each other more frequently when they are able to communicate costless signals [4, 5]. This suggests that algorithms may also need to engage in cheap talk to be able to establish cooperative relationships with people. However, while signaling comes naturally to humans, the same cannot be said of machine-learning algorithms. To be useful, costless signals should be connected with behavioral processes. Unfortunately, most machine-learning algorithms have low-level internal representations that are often not easily expressed in terms of high-level behavior, especially in arbitrary scenarios. As such, it is not obvious how these algorithms can be used to generate and respond to costless signals at levels that people understand. However, unlike typical machine-learning algorithms, the internal structure of S++ provides a clear, high-level representation of the algorithm's dynamic strategy that can be described in terms of the dynamics of the underlying experts. Since each expert encodes a high-level philosophy, we can use S++ to generate signals (i.e., cheap talk) that describe its intentionality.

Thus, we extended S++ with the ability to generate speech acts, which were voiced to the human partner through a Nao robot. This early version of S# (which we refer to as S#–) did not, however, take into account signals communicated by its partner. We then conducted a second user study[18] to determine whether this extended algorithm could establish cooperative relationships with people. Ninety-six people participated in this study, which was approved by Masdar Institute's Human Subjects Research Ethics Committee. Informed consent was obtained from each participant.

For ease of exposition, we discuss this user study in two parts. In Part 1, we discuss the condition of the study in which the participants were not allowed to communicate with each other. In Part 2, we discuss the impact of communication on the ability of the players to cooperate with each other.

Experimental Setup (Part 1)

We discuss, in turn, the protocol followed in Part 1 of the user study, the games used in the study, the algorithms involved, and the user interface through which participants played the games.

Protocol Part 1 of this user study followed a 3x2 mixed design, in which the between-subjects variable was the partner type (Humans, CFR, or S++) and the within subjects variable was the game scenario (the SGPD and the Block game). Twelve groups of four participants each were recruited from the Masdar Institute community to participate in this part of the study. Half of the groups were assigned to a human-human condition, and were thus paired with each other as shown in the schedule shown in Supplementary Table 25a. The other six groups were assigned to a human-machine condition in which two of the subjects were paired with CFR [87, 88] and two of the subjects were paired with S++, as specified in Supplementary Table 25b. The condition that each group was determined in advance without knowledge of the participants that would be participating in that group.

The study proceeded as follows:

[18]This study was originally presented at HRI 2015 [86].

(a) Human-human condition

Schedule for a Group of Four Subjects			
Order	Game	Pairing 1	Pairing 2
1	SPGD	H1-H2	H3-H4
2	Block Game	H1-H3	H2-H4

(b) Human-machine condition

Schedule for a Group of Four Subjects					
Order	Game	Pairing 1	Pairing 2	Pairing 3	Pairing 4
1	SPGD	H1-CFR	H2-CFR	H3-S++	H4-S++
2	Block Game	H1-CFR	CFR-H2	H3-S++	S++-H4

Supplementary Table 25: The schedule of games and pairings followed in each condition of Part 1 of the second user study. Subjects participated in Part 1 of this user study in groups of four, in which each subject was randomly assigned an ID (H1, H2, H3, or H4). Each group was designated to one of two conditions: human-human or human-machine.

1. The group of four participants was assigned a condition (human-human or human-machine).
2. Each participant was randomly assigned an ID (H1, H2, H3, or H4).
3. The SGPD was explained to the participants using slides and a video demo. Participants were allowed to ask as many questions as necessary until it was clear they understood the rules of the game.
4. The participants played a 54-round SGPD with their assigned partner (see Supplementary Table 25). Participants played the game on a desktop computer using the arrow keys to select movements. The participants were not told the number of rounds in the game or the identity of their partner. They were not allowed to talk or signal to each other; computers were placed so that participants could not see each other.
5. Each participant completed the post-game survey shown in Supplementary Figure 22 (the survey shown in Supplementary Figure 23 was used in Part 2 of the user study).
6. When all participants had completed the survey, they were introduced to the Block Game using slides and a video demo. Participants were allowed to ask as many questions as necessary until it was clear they understood the rules of the game.
7. The participants played a 51-round Block Game with the same player type as before. The player listed first in the pairing (Supplementary Table 25) was assigned to be the player to select a block first – a role which was kept constant in each round of the game. The participants used the mouse to select the blocks on a GUI.
8. The participant completed the same post-experiment survey as in Step 5.

Participants were paid a $5.00 show-up fee. To incentivize the participants to try to maximize their own payoffs (which was the stated objective of each game), participants were also paid money proportional to the points they scored in the games (they could earn up to an additional $15.00). The GUI displaying the game interface also showed the amount of money the participant had earned so far in local currency.

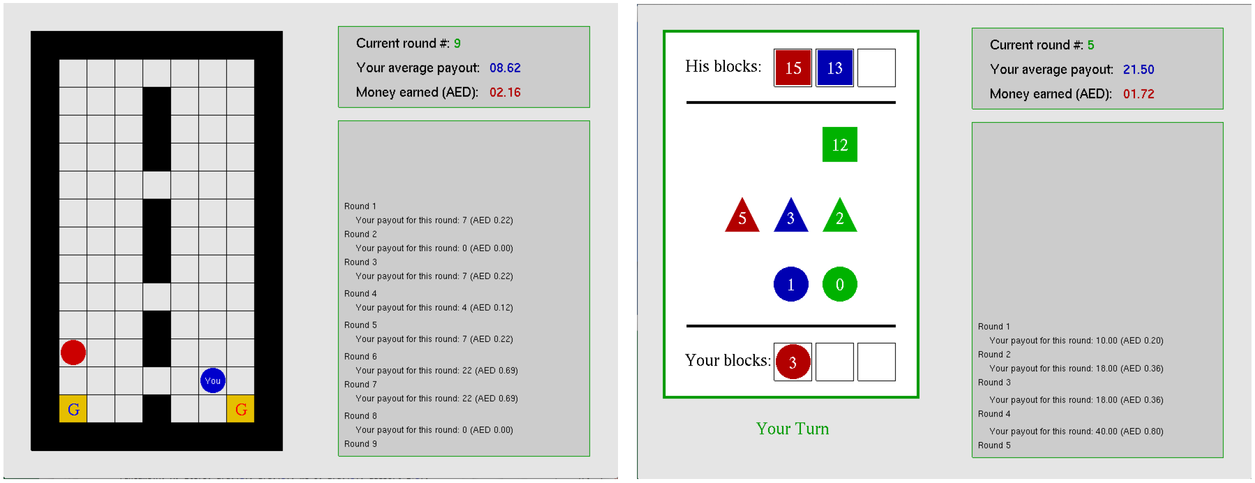

(a) GUI used to play the SGPD

(b) GUI used to play the Block Game

Supplementary Figure 17: Graphical user interfaces used by participants to play the SGPD and the Block Game, respectively. **(a)** In the SGPD, the participant used the arrow keys on the keyboard to select which direction to move their icon through the maze. **(b)** In the Block Game, participants selected blocks by clicking on the desired block with the mouse.

In addition to the human-human and human-machine pairings specified in Supplementary Table 25 (which produced 12 samples for each human-human and human-agent pairing in each game), machine-machine simulations were run to serve as additional points of comparison. Since a new version of CFR and S++ was initiated for each interaction, we were able to conduct additional runs for these machine-machine pairings; the number of available participants had no impact on these simulations. Thus, all results for S++/S++ and CFR/CFR pairings are each obtained from 24 independent trials. Given statistical variations observed in the first user study, these samples sizes were deemed adequate to identify substantial differences in performance between pairings, though no formal power analysis was performed prior to the study.

Games As specified in Supplementary Table 25, each participant first played the stochastic game prisoner's dilemma (SGPD), followed by the Block Game. Both of these games are explained in Supplementary Note 2. Recall that various levels of cooperation are possible in each game. So-called *mutual cooperation* in the SGPD is when both players choose to move through Gate B. In the Block Game, mutual cooperation is achieved when the players take turns (across rounds) getting all the squares and all the triangles, respectively.

User Interface Participants played the games using GUIs. Screen shots of the GUIs used in both games are shown in Supplementary Figure 17. In both GUIs, the game board was displayed on the left portion of the GUI, while the history of play was displayed on the right portion of the GUI. The participant's own payoffs in each round, his/her average payoff over all rounds, and his/her total earnings were all displayed prominently on the GUI interface. However, as in the other user studies, the payoffs of the participant's partner was not recorded on the GUI (though the participant could infer them). This was done to emphasize that the goal of the game was to maximize one's own payoffs rather than to outscore one's partner.

Algorithms S++ was originally designed for repeated normal-form games. A recently published extension to the algorithm allows it to be used in multi-stage RSGs [2] by simply defining experts that define policies in each state of the RSG. As in normal-form games, these experts include *leader* and *follower* strategies, as well as a third genre of expert strategy called *preventative strategies* (see Crandall [2] for details).

As in the first user study, we compared the S++'s ability to learn to interact with people with the ability of another learning algorithm. In this study, we compared the performance of S++ with CFR (counterfactual regret), which has become a core component of world-class computer poker algorithms [87, 88, 3]. CFR generalizes the idea of regret [14] to large extensive-form[19] games with imperfect information. CFR was originally designed to compute a Nash equilibria in large zero-sum games, though regret-minimization has often been used as a criteria for online learning in general-sum games (e.g., [15]). However, regret minimization is not guaranteed to correspond to payoff maximization in such games, and can even correlate positively with reduced payoffs when associates employ other learning algorithms [17, 18, 65, 1].

Players were not told the identify of their partner. However, because algorithms make selections almost instantaneously in most cases, a participant might be able to infer that their partner was a computer algorithm due to how fast their partner selected actions. In the first user study, we addressed this problem by making each round last a fixed amount of time. If both players selected actions before this time elapsed, they still had to wait until time elapsed before observing the result. They were also forced to make decisions within the allotted time. This mechanism seemed to cause boredom or frustration among participants (the amount of time allotted tended to be either too long or too short), and is even less practical when rounds consist of many consecutive moves, as in the SGPD and the Block Game.

Thus, in this second user study, we eliminated fixed-length rounds and instead introduced artificial delays into the computer algorithm to try to make decision-times more human-like. To do this, we logged the amount of time that humans took to make decisions, and then used a k-nearest neighbor algorithm to infer how long the computer algorithm should delay before selecting an action. This technique was somewhat effective, though delays still sometimes seemed unnatural to participants (and, hence, did not seem human-like) due to the complexity of determining the relevant features of the game state that caused changes in human decision times[20].

Outcomes (Part 1)

As in the first user study, we are interested in comparing the behavior and performance of computer algorithms with that of humans. We are particularly interested in finding algorithms that learn to consistently cooperate with people. Thus, in discussing the results of Part 1 of this second user study, we begin by discussing the proportion of mutual cooperation achieved by the algorithms. We then discuss the payoffs received by the algorithms in each pairing and game.

Mutual Cooperation The proportion of mutual cooperation achieved by each pairing in both games is summarized in Supplementary Figure 18. These results have identical trends to those of the first user study. Only S++/S++ pairings achieved high levels of mutual cooperation (in both games). Additionally, neither

[19] So that CFR can be used in the SGPD, the game is reduced to an extensive-form game by truncating a round after a certain number of moves (40 in our case). Since each round of the SGPD usually has little more than 30 moves (unless players make irrational moves), this simplification does not affect the strategies of rational players.

[20] Given that our k-nearest neighbor machine-learning algorithm failed to produce human-like delays in the decision process, we used tit-for-tat time delays in the third user study (Supplementary Note 7), wherein the computer algorithm delayed the same amount of time as the participant delayed in the previous move. This approach, while not perfect, seemed to be much more effective, as fewer participants commented on the delays in their partners' moves.

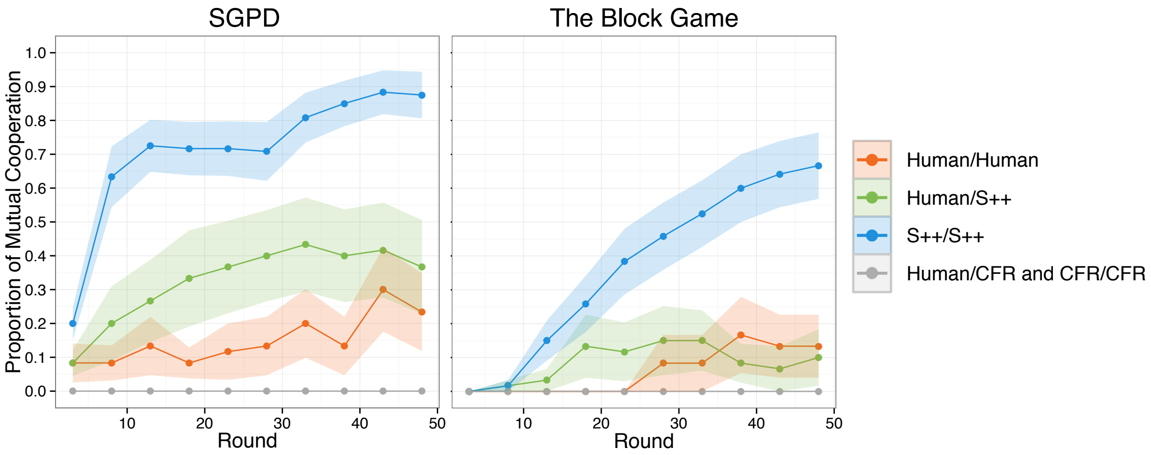

Supplementary Figure 18: Proportion of mutual cooperation in each game. Each data point is the average of five-round increments. Error bands show the standard error on the mean. CFR/CFR and Human/CFR pairings always had the same value (no mutual cooperation) in all rounds in each game, and hence are both represented by the same line.

CFR, S++, or Humans were able to consistently cooperate with people, though S++ tended to cooperate with people at least as much as people did. Human/S++ pairings were able to reach about 40% mutual cooperation in the SGPD, but were not nearly as effective in the Block Game. Because of its internal representation (which tends to lead to the computation of one-shot NE), CFR does not ever play the mutually cooperative solution in these games, regardless of the behavior of its partner.

These trends are confirmed by a negative binomial regression in which the number of cooperative rounds within each pair was the dependent variable, and in which Game, Pairing, and their interaction were predictors (because sample variance was largely greater than sample mean in most cells, a Poisson regression was not appropriate; additionally, Human/CFR and CFR/CFR pairings were not included in the analysis due to their constant non-cooperation). This analysis showed that S++/S++ pairings cooperated more than either S++/Human pairings ($z = -2.6$, $p = 0.01$) or Human/Human pairings ($z = -3.1$, $p = 0.002$), but that S++/Human pairings did not significantly differ from Human/Human pairings ($z = -0.5$, $p = 0.61$).

Supplementary Table 26, which shows the number of participants that achieved each outcome (by the end of 50 rounds) when paired with each player type, provides further insight into the ability of the learning algorithms to collaborate with people. In the SGPD, both S++ and Humans learned mutual cooperation (*Both B*) with some participants (S++ cooperated with five, Humans with three). The rest of the participants typically learned mutual defection (*Both A*) when paired with S++ and Humans, though S++ tended to continue to attempt to pass through Gate B every few rounds in hopes of achieving a more-profitable outcome. In the Block Game, Humans and S++ rarely reached the most collaborative solutions.

Payoffs Received As in the other user studies, the correlation between payoffs received[21] and the proportion of mutual cooperation achieved was quite high ($r(238) = 0.695$, $p < 0.001$). A visual comparison of Supplementary Figures 18 and 19, the latter of which shows the average per-round payoffs obtained by the algorithms in self play and when paired with humans, illustrates this correlation. As points of reference in

[21]To allow for analysis across games, we used the standardized z-score in each game in this correlation test.

(a) SGPD (no communication)

Player	Primary Outcome				
	**Both B**	**Alt A-B**	**Both A**	**Bully**	**Other**
Humans	3	0.5	7.5	1	0
S++	5	0	5	0	2
CFR	0	0	11	1	0

(b) Block Game (no communication)

Player	Primary Outcome					
	**Alt □-△**	**Alt red-blue**	**All diff**	**Pure red-blue**	**Pure □-△**	**Other**
Humans	2	2	5	2	0	1
S++	1.5	0.5	4.5	2	0	3.5
CFR	0	0	5	5	0	2

Supplementary Table 26: The number of subjects that reached each outcome by the end of 50 rounds when paired with a human partner in **(a)** the SGPD and **(b)** the Block Game, respectively.

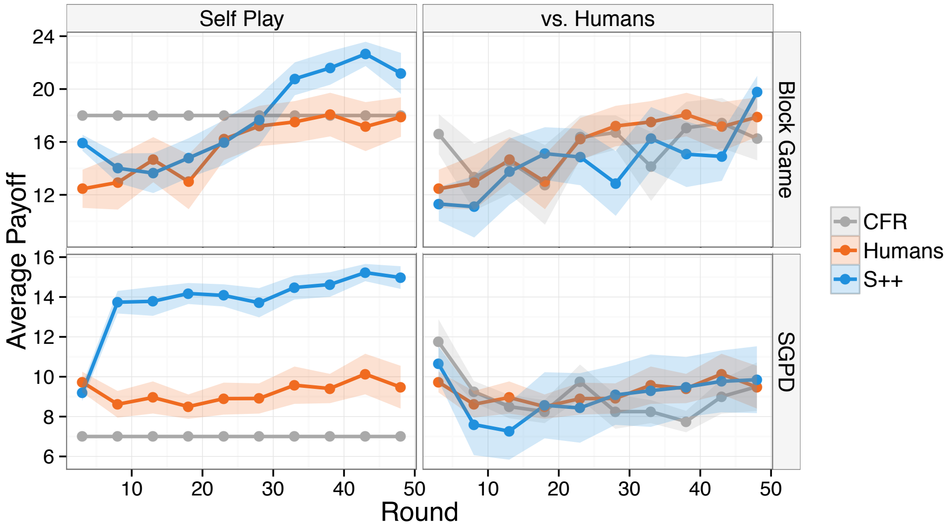

Supplementary Figure 19: The average payoffs achieved by Humans, CFR, and S++ (a) in self play and (b) when paired with humans, respectively. The data is plotted as the average over five-round increments. Error bars show the standard error on the mean. Mutual cooperation in the Block Game yields an average payoff of 25, while mutual cooperation in the SGPD yields an average payoff of 16. Thus, the algorithms' failures to consistently cooperate with each other (with the exception of S++/S++ pairings) led to substantially reduced payoffs.

considering Supplementary Figure 19, note that mutual cooperation in the Block Game yields an average payoff of 25, while mutual cooperation in the SGPD yields an average payoff of 16.

S++'s ability to establish cooperative relationships in self play led to higher payoffs in both games. In the SGPD, mutual cooperation typically emerged within the first ten rounds, which in turn led to higher payoffs throughout the game. It took S++ longer to establish mutual cooperation in self play in the Block Game, which in turn meant that it did not achieve higher payoffs until later rounds in this game. On the other hand, because CFR is unable to model mutually cooperative solutions in either game, its payoffs remain low in both games in self play. Likewise, people also received low payoffs in self play.

When paired with humans, none of the players consistently achieved high payoffs in either game, nor was their a substantial difference in the payoffs achieved by the players.

Experimental Setup (Part 2)

In Part 2 of this study, we gave the players the opportunity to communicate with their partner using cheap talk. That is, human pairs were allowed to talk freely to each other, face-to-face, throughout the duration of the game. To give S++ the ability to generate cheap talk, we developed an early (incomplete) version of S#, which we refer to as S#–. S#– is identical to S++, except that it simultaneously generates and voices (in this case through a Nao robot) speech acts to its partner. However, unlike the full version of S# considered in the next (third) user study, S#– does not take into consideration communication signals from its partner when selecting experts.

We first describe the experimental setup for this study, and then present the results.

Procedure In Part 2 of this user study, participants were randomly assigned to one of three types of partners (Humans or two different forms of S#–). Forty-eight participants (none of whom took part in Part 1) participated in this part of the study. Half of the participants were assigned to the human-human condition, and were thus paired with each other as shown in Supplementary Table 25a. The other 24 participants, who completed the study in groups of two, were assigned to a human-machine condition. Twelve of the participants were partnered with a version of S#– (in both games) that voiced only feedback/sentiments (i.e., no plans – Supplementary Table 27a), while the other twelve subjects were partnered with a version of S#– that voiced both feedback and proposed plans (i.e., plans – see Supplementary Table 27b).

Thus, viewing Parts 1 and 2 of the study together (and excluding subjects assigned to play against CFR in Part 1), the participants in the study, who each played the SGPD game and the Block game, were randomly assigned to one cell of a 2 x 3 between subject design: Communication (between-participants: Yes or No) × Partner (between-participant: Human, or one of two versions of S#–, with or without the capacity to talk about plans). Because the first level (human partner) meant that participants were paired with each other, this level required twice as many human participants as the other levels, in order to comprise as many pairs (hence, random, allocations took into account this need). Note also that practically speaking, the two versions of S#– are undistinguishable in the absence of communication, which means that the protocol was exactly the same in two cells of the design.

The procedure followed for each subject was the same in Part 2 as in Part 1 with two exceptions. First, players were aware of who their partner was, as their partner was sitting right in front to them. Second, the players were allowed to talk to each other freely, without restriction, throughout the game.

User Interface The GUI through which participants played the game was the same as the one used in Part 1 of this user study (Supplementary Figure 17). When participants were paired with S#–, speech acts were

(a) Human-S#– (no plans) condition

Schedule for a Group of Two Subjects			
Order	Game	Pairing 1	Pairing 2
1	SPGD	H1/S#– (no plans)	H2/S#– (no plans)
2	Block Game	S#– (no plans)/H1	H2/S#– (no plans)

(b) Human-S#– (plans) condition

Schedule for a Group of Two Subjects			
Order	Game	Pairing 1	Pairing 2
1	SPGD	H1/S#– (plans)	H2/S#– (plans)
2	Block Game	S#– (plans)/H1	H2/S#– (plans)

Supplementary Table 27: In the human-machine condition of Part 2 of this second user study, subjects (denoted H1 and H2) participated in the study in groups of two. In the Block Game, the player that selects a block first in each round is the player listed first in the pairing.

delivered by a Nao robot, who was placed before the participants.

Communication Both the face-to-face talk used between human players and the speech acts voiced by S#–are forms of *cheap talk*. Cheap talk refers to non-binding, unmediated, and costless communication [89, 90]. Cheap talk has been cited as a means for equilibrium refinement [91], and has been shown to improve collaborations among people in some scenarios [92].

In this user study, we consider whether a learning robot can employ cheap talk to help establish cooperative relationships with people. We are unaware of past work interweaving cheap talk with online learning. This is potentially due to the difficulty of knowing what to communicate, as most learning algorithms have representations that are difficult to understand and articulate. However, we note parallels to the work of Thomaz and Breazeal [93], in which a simulated robot (tasked to learn to solve an environment that can be modeled as a Markov decision process) signaled uncertainty to a human teacher by pausing in stages in which multiple actions had similar Q-values. This helped the human to understand what the robot still needed to learn. However, in multi-stage RSGs in which their is conflicting interest between players, discussions about local Q-estimates are likely to be at too low of a level to fully resonate with human partners. Additionally, we anticipate that the competitive natures of these games will make such low-level communications insufficient.

We consider two kinds of cheap talk to encode in S#–: *feedback cheap talk* and *planning cheap talk*. We refer to cheap talk that addresses assessments of past events as feedback cheap talk. As an example, a person or robot might comment on their satisfaction with past events, or comment on how past events made them feel. Additionally, feedback cheap talk includes assessing the past behaviors of one's partner, perhaps expressing how the other player's actions make them feel, or expressing what they wished the other player had done instead. We anticipate that feedback cheap talk could potentially be produced from most learning algorithms with careful thought.

Planning cheap talk is forward looking. It involves suggesting future behavior to one's partner and/or revealing one's current or future strategy. Of course, such cheap talk is non-binding – neither of the players must actually do what is spoken. Regardless, we anticipate that most learning algorithms have representations that are too cryptic to easily be made to produce effective planning cheap talk for arbitrary scenarios,

as humans typically communicate plans at higher levels than typical machine-learning algorithms reason.

Algorithms In the first user study and in Part 1 of the second user study, S++ demonstrated some ability to establish cooperative relationships. In both repeated normal-form and multi-stage RSGs, S++/S++ pairings typically converge to mutual cooperation. On the other hand, CFR does not compute mutually cooperative solutions in many games – its representation does not allow it to effectively model such solutions. Thus, in Part 2 of this study, we focus exclusively on S++ in hopes of finding an algorithm that can consistently cooperate with people (and, in so doing, achieve higher payoffs).

Unlike many learning algorithms, S++ is structured so that its high-level strategies are understandable (and expressible) to people. Thus, it can be used to generate both feedback and planning cheap talk that is game-generic, such that the same cheap talk can be used in many different RSGs.

S++ generates speech acts (i.e., cheap talk) for multi-stage RSGs in essentially the same way as in normal-form games (see Supplementary Note 4). For completeness, we describe the exact speech system used in the versions of S#– used in this study. This speech-generation system has three components: (1) a set of speech acts, (2) the set of events used by S++ to establish its state, and (3) the finite state machines (FSMs) with output that are used to generate speech acts given the algorithm's state and the events that occur.

Set of speech acts. The set of speech acts used by S#– in this second user study are given in Supplementary Table 28. By and large, this set of speech acts allows S#– to express similar ideas as the smaller set of speech acts used in the third user study (Supplementary Table 21). The set of speech acts used in this user study (Supplementary Table 28) is larger, but many of the phrases have similar meanings.

Despite the similarities between this set of speech acts and those used in the third user study, there is a substantial difference between these two sets. While solutions can be referred to by *joint actions* in normal-form games, the same is not realistic in multi-stage RSGs. Instead, solutions are specified indirectly through words such as *cooperation* (e.g., speech ID 2) and statements that compare and contrast payoffs (e.g., speech IDs 3, 4, 9-12). The exact meaning is left to the other player to interpret. In this way, the speech system used by S#– remains game-generic.

The speech acts given in Supplementary Table 28 contain statements that allow S#– to both provide feedback and propose plans. Speech acts that we considered to be part of plan-making are marked by bold type in the table. In the no-plans version of S#– used in this study, these plan-related (bold) phrases are not spoken.

Events. The set of events used by S#– in multi-stage RSGs (Supplementary Table 29) is very similar to those used in normal-form games (Supplementary Table 22; third user study), except that we do not need events related to reactions to the other player's signals (events x, y, and z). This is because S#–, unlike S#, does not respond to its partner's signals.

Finite state machines with output. The FSMs with output used by S#– in this user study are given in Supplementary Table 30. The FSMs have similar structure as those used in user study 3 (described in Supplementary Note 4), with some stylistic variations.

Like S#, S#– (plans) voices planning cheap talk for two different kinds of events. First, because each expert encodes a (perhaps radically) different high-level strategy, the random selection of experts (i.e., exploration) can appear (to a human partner) to be disjoint, irrational reasoning. Thus, when S#– changes which expert it follows, it produces a speech act that notifies the human partner of this change. Example speech acts include "I've changed my mind," and "I've had a change of heart." Additionally, when this switch in strategies leaves some promised act undone (such as a promised punishment), the robot tries to soften the discontinuity by saying "I'll let you off this time."

ID	Text	ID	Text
1	**Here's the deal:**	28	We can both do better than this.
2	**Let's cooperate with each other.**	29	I've changed my mind.
3	**We can do this by taking turns receiving the higher payoff.**	30	I've had a change of heart.
		31	That round's result was not good enough for me.
4	**I deserve a higher payoff.**	32	You are an idiot!
5	**If you do not cooperate, I'll punish you thereafter.**	33	This is not good for our relationship
6	**If you do not comply, I'll punish you.**	34	For the sake of our relationship, cease this untoward behavior.
7	**I forgive you.**	35	Friends do not do that to each other.
8	**Cooperation will bring us both a higher payoff.**	36	**I'm going to teach you a lesson you will not forget.**
9	**It's my turn to get the higher payoff. You'll get the higher payoff next time.**	37	**You will pay for this!**
		38	**I'm going to make sure you do not profit from this malicious act.**
10	**It's your turn to get the higher payoff.**		
11	**My turn.**	39	Good for me.
12	**Your turn.**	40	That's what I wanted.
13	Sweet. We are getting rich. Let's continue this.	41	That's what I'm talking about.
14	I trusted you to `<game-specific action label>`	42	Excellent!
		43	Great!
15	I thought you should `<game-specific action label>`	44	Satisfactory.
		45	Good.
16	I will let you off this time.	46	I like this.
17	You jerk!	47	Nice!
18	You buffoon!	48	That was fair.
19	You fool!	49	Serves you right, jerk.
20	Curse you!	50	In your face!
21	You betrayed me!	51	Take that!
22	That was selfish of you	52	**I'll play fair if you'll play fair.**
23	That was not fair!	53	**I insist on equal payoffs.**
24	Are you only thinking of yourself.	54	That's what you get.
25	**I will not let you cheat me.**	55	You forced my hand.
26	The amount of points we get is up to you.	56	I must be cursed.
27	Do not threaten me.	57	Darn.

Supplementary Table 28: Speech acts used by S#– in the second user study. Planning speech acts are given in bold, feedback speech acts are in plain text. Thus, both plain text and bold speech acts were voiced by S#– (plans), while S#– (no plans) only voiced the speech acts given in plain text.

Symbol	Event descriptions
n	S#– selected a new (distinct from the previous cycle) expert to follow. The partner currently has no guilt
ng	S#– selected a new (distinct from the previous cycle) expert to follow. The partner is currently guilty
c	An expert has been executed for a complete cycle.
b	S#– believes the players can both receive higher payoffs than what they received in the previous cycle. This event is not generated if the new expert is MBRL.
s	The current expert is *satisfied* with last round's payoff.
f	The current expert *forgives* the other player.
d	The other player *defected* against S#–.
g	The other player profited from its defection (and is *guilty*).
p	The expert has *punished* its guilty partner.
u	S#– did not succeed in punishing its guilty partner.

Supplementary Table 29: A list of event symbols and event descriptions. The first six events correspond to master-level (i.e., expert selection) events of S++, while the last six events impact individual experts.

(a) Master-level FSM, executed after each visit to a state in an expert-level FSMs.

State	State Transitions					Speech Acts (Output)				
	Events					Events				
	¬(n or ng)	n	ng	n, b	ng, b	¬(n or ng)	n	ng	n, b	ng, b
s_0	s_2	s_1	s_1	s_1	s_1	ε	r({29,30})	16+r({29,30})	28+r({29,30})	28+16+r({29,30})
s_1	Go to state s_0 of the FSM corresponding to the newly selected expert.					ε	ε	ε	ε	ε
s_2	Return to the current state of the current expert.					ε	ε	ε	ε	ε

(b) FSM for the MBRL expert.

State	State Transitions			Speech Acts (Output)		
	Events			Events		
	NUL	¬s	s	NUL	¬s	s
s_0	s_0	s_0	s_0	–	r({31,56,57})	r({39,44,47})

(c) FSM for the maxmin expert.

State	State Transitions			Speech Acts (Output)		
	Events			Events		
	NUL	¬s	s	NUL	¬s	s
s_0	s_0	s_0	s_0	–	–	–

(d) FSM for the preventative strategy Bouncer. Speech acts generated from state s_1 are only generated with probability given by $\frac{1}{1.0+\kappa/15.0}$, where κ is the number of times the event $\neg s$ and s have occurred, respectively, when Bouncer is active.

State	State Transitions			Speech Acts (Output)		
	Events			Events		
	NUL	¬s	s	NUL	¬s	s
s_0	s_1	–	–	25+53+52	–	–
s_1	–	s_1	s_1	–	r({26,27,54,55})+r({25,53,52})	r({41,45,46,48})

(e) FSM for leader experts. For "fair" leader experts, <Intro> = 1+2+*3*+5. For "bully" leader experts, <Intro> = 4+6.

State	State Transitions						Speech Acts (Output)					
	Events						Events					
	NUL	g	f	s	d	p	NUL	g	f	s	d	p
s0	s1	s0	s0	s0	s0	s0	<Intro>	–	–	–	–	–
s1	–	s9	s1	s2	s2	s1	–	r({17-20,32})+14+r({36-38})	–	r({40-43})+*9,10*	–	–
s2	–	s10	s2	s3	s3	s2	–	r({17-20,32})+14+r({36-38})	–	r({40-43})+*9,10*	–	–
s3	–	s11	s3	s4	s4	s3	–	r({17-20,32})+14+r({36-38})	–	r({40-43})+*9,10*	–	–
s4	–	s11	s4	s5	s5	s4	–	r({17-20,32})+14+r({36-38})	–	r({40-43})+*11,12*	–	–
s5	–	s11	s5	s6	s6	s5	–	r({17-20,32})+14+r({36-38})	–	r({40-43})+*11,12*	–	–
s6	–	s11	s6	s7	s7	s6	–	r({17-20,32})+14+r({36-38})	–	*11,12*	–	–
s7	–	s11	s7	s8	s8	s7	–	r({17-20,32})+14+r({36-38})	–	13	–	–
s8	–	s11	s8	s8	s8	s8	–	r({17-20,32})+14+r({36-38})	–	–	–	–
s9	–	s9	s3	s10	s10	s9	–	–	7+8+<Intro>	–	–	r({49-51})
s10	–	s10	s4	s11	s11	s10	–	–	7+8+<Intro>	–	–	r({49-51})
s11	–	s11	s5	s11	s11	s11	–	–	7+8+<Intro>	–	–	r({49-51})

(f) FSM for follower experts. For "fair" follower experts, <Intro> = 2+*3*. For "bully" follower experts, <Intro> = 4.

State	State Transitions						Speech Acts (Output)					
	Events						Events					
	NUL	g	f	s	d	p	NUL	g	f	s	d	p
s0	s1	s0	s0	s0	s0	s0	<Intro>	–	–	–	–	–
s1	–	s2	s2	s2	s2	s2	–	r({21-24})+15	–	r({40-43})	–	–
s2	–	s3	s3	s3	s3	s3	–	r({21-24})+15	–	r({40-43})	–	–
s3	–	s4	s4	s4	s4	s4	–	r({21-24})+15	–	r({40-43})	–	–
s4	–	s4	s4	s5	s5	s4	–	r({21-24})+15	–	r({40-43})	–	–
s5	–	s4	s5	s6	s6	s5	–	r({21-24})+15	–	r({40-43})	–	–
s6	–	s4	s6	s7	s7	s6	–	r({21-24})+15	–	–	–	–
s7	–	s4	s7	s8	s8	s7	–	r({21-24})+15	–	13	–	–
s8	–	s4	s8	s8	s8	s8	–	r({21-24})+15	–	–	–	–

Supplementary Table 30: Feedback and planning cheap talk is generated by a set of finite state machines (FSMs) with output. r(X) denotes a randomly selected speech act from the set X of speech acts. '+' indicates concatenated strings. <Intro> specifies a sequence of speech phrases determined by the target solution advocated by the expert strategy. "Fair" strategies correspond to target solutions in which the partner receives a similar or higher payoff as the algorithm; "bully" strategies target solutions that give the algorithm a higher payoff than its partner. *n* indicates that speech act n is spoken only if the target solution of the strategy is an alternating target solution; *x,y* is the appropriate selection between speech acts x and y given the current step in the target solution.

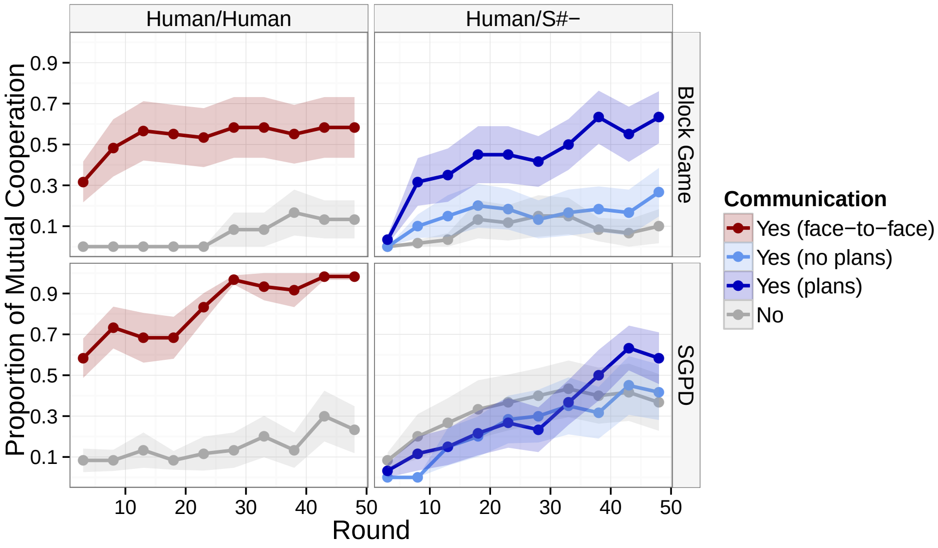

Supplementary Figure 20: Proportion of mutual cooperation achieved in the second user study for Human/Human and Human/S#– pairings. Note that when communication is not possible, S#– is equivalent to S++. Participants in Human/Human pairings were allowed to talk freely to each other face-to-face, while communication between people with S#– was limited to one-way communication spoken by S#–, since S#– does not internalize communication signals from its partner.

As in normal-form games, each expert can also produce planning cheap talk. These plans can be communicated at a high level, as most experts used by S++ encode a high-level strategic ideal that is easily understood by people. For example, one of S++'s experts (called Bouncer) seeks to minimize the difference between the robot's and the human's payoffs. When Bouncer is selected, S#– announces "I will play fair if you will play fair," and that it insists on equal payoffs and will not be cheated. Others of S++'s experts encode trigger strategies, which carry out epochs of cooperation and punishment depending on the behavior of the associate. These trigger strategies can easily be articulated when they are selected. Furthermore, these trigger strategies can be modeled with simple FSMs, which S#– uses to produce speech acts that inform its partner about switches between stages of cooperation and punishment.

Outcomes (Part 2)

Recall that our goal is to produce a learning algorithm that establishes cooperative relationships with people as well as people do. Thus, we compare the performance of S#– to that of people (when partnered with people) with respect to the proportion of mutual cooperation achieved as well as payoffs received when associating with people.

Mutual Cooperation The average proportion of mutual cooperation achieved by each pairing (under both communication conditions) in each game is shown in Supplementary Figure 20. The figure shows two important results. First, consistent with previous work [4, 5], Human/Human pairings benefitted substantially from the ability to engage in cheap talk. Whereas mutual cooperation was nearly non-existent between people when they could not talk to each other, mutual cooperation typically emerged between people in the

(a) SGPD

Player	Primary Outcome				
	Both B	Alt A-B	Both A	Bully	Other
Humans	3	0.5	7.5	1	0
Humans (f2f)	12	0	0	0	0
S++	5	0	5	0	2
S#– (no plans)	5.5	0	5.5	1.0	0
S#– (plans)	9.5	0	2	0.5	0

(b) The Block Game

Player	Primary Outcome					
	Alt □-△	Alt red-blue	All diff	Pure red-blue	Pure □-△	Oth-er
Humans	2	2	5	2	0	1
Humans (f2f)	7	2	1	0	2	0
S++	1.5	0.5	4.5	2	0	3.5
S#– (no plans)	3.5	0	3	2	1.5	2
S#– (plans)	9	0	0	1	2	0

Supplementary Table 31: The number of subjects that reached each outcome by 50 rounds when paired with a human partner. Note that the data for Humans and S++ was obtained from Part 1 of the study. S++ is equivalent to S#– when communication is not possible.

communication condition.

Second, communication did not produce more substantial levels of mutual cooperation between Humans and S#– when signals expressed by S#– did not include plan proposals. However, when S#– did propose plans, higher levels of cooperation began to emerge at the end of the first game (the SGPD), and then become substantial in the Block Game (the second game played). This result suggests that, if S++ is combined with the ability to propose (through speech acts) the joint plans it intends to carry out, it can establish much more cooperative relationships. However, these cooperative relationships between people and machines appear to emerge much slower than in Human/Human pairings (when communication is possible), an issue we seek to address in the third (and final) user study presented in this paper.

Statistical analyses confirm these trends. For Human/Human pairing, the negative binomial regression (number of mutually cooperative rounds as the dependent variable; Communication yes/no, Game, and their interaction as predictors; Poisson regression was inappropriate due to over-dispersion) showed a strong positive effect of Communication ($z = 3.1$, $p = 0.002$). For S++/Human pairings, the analysis showed a marginal positive effect of Communication ($z = 1.8$, $p = 0.07$). This marginal effect is better understood by refining the Communication variable into no communication, communication without plans, and communication with plans. This additional analysis showed that communication with plans improved cooperation in S++/Human pairings ($z = -2.1$, $p = 0.04$), but that communication without plans had no significant effect ($z = -0.8$, $p = 0.44$).

Convergence characteristics provide further insight into how cheap talk impacted S#–'s ability to establish cooperative relationships with people. While feedback cheap talk alone produced little increase in the number of highly collaborative outcomes, feedback and planning cheap talk together led to substantial increases in highly cooperative outcomes by the end of 50 rounds (Supplementary Table 31).

In summary, allowing for communication greatly improved cooperation. The version of S#– that could not communicate about plans failed to take full advantage of communication, though, as its level of mutual cooperation with humans remained lower than human-human levels of cooperation. The version of S#– that could communicate about plans fared better. At least in the Block Game, it achieved the same level of mutual cooperation as seen in pairings of humans enjoying full communication. Even this version of S#– was limited, though, by its inability to process the messages sent by its human partners. The third and final user study investigated the levels of mutual cooperation achieved by S#, the version of S++ endowed with the ability to process as well as send messages to its partner.

Recall that, in this user study, the ability to communicate also coincided with knowledge of the identify of one's partner. While this could (and likely does) have a substantial impact on the behavior of people, the

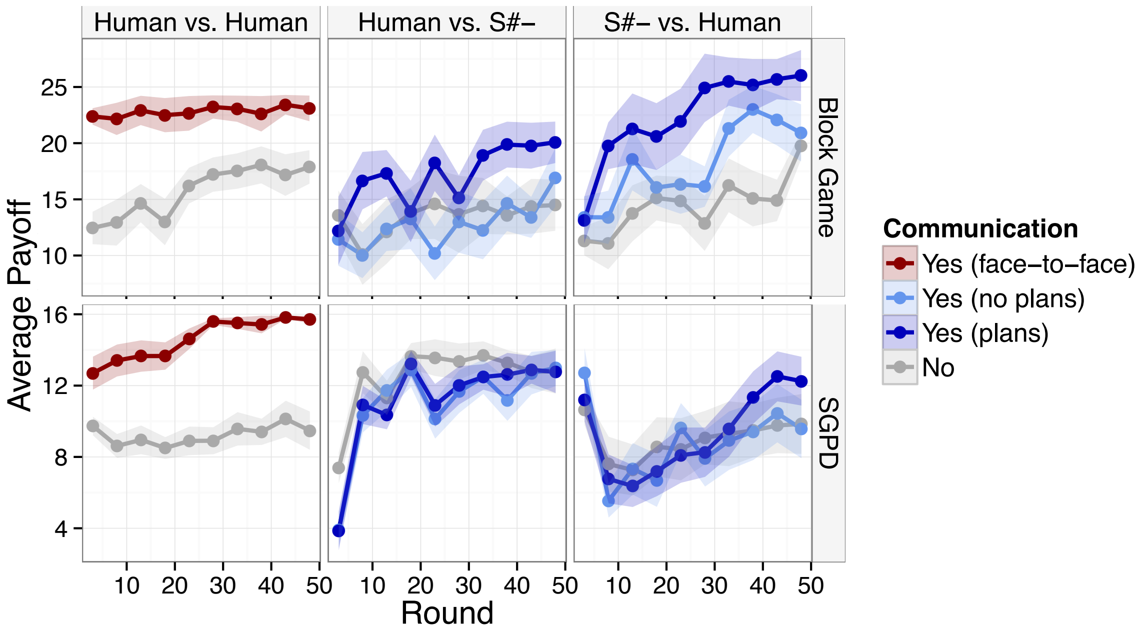

Supplementary Figure 21: The average payoffs achieved by the players in both Human/Human and Human/S#– pairings.

increase in cooperative behavior cannot be attributed solely to this knowledge, given that S#– (no plans) did not increase cooperation substantially, but S#– (plans) did. In the subsequent user study, we isolate these two factors to better understand the impact of communication on cooperation in human-machine relationships.

Payoffs Received Supplementary Figure 21 illustrates that, as in other user studies, payoffs received tend to correlate highly with the proportion of mutual cooperation achieved by these players.

Reflections

The results of Part 1 of this second user study essentially mirrored the results of the first user study. S++ is able to establish cooperative relationships with other players that employ S++, but it is unable to consistently establish cooperative relationships with people. People also failed to consistently establish cooperative relationships with people in these games.

Thus, in Part 2 of the user study, we investigated whether communication could increase cooperation between humans and between S++ and humans. To do this, we created S#–, an early version of S# that combines S++ with the ability to generate and voice cheap talk. The results of this user study are summarized as follows:

1. As in previous work [4, 5], people benefitted substantially from the ability to communicate via cheap talk. The ability to communicate allowed people to establish cooperative relationships in most interactions.

2. When S#– generated speech acts that articulated plans, it was able to establish cooperative relationships with people more frequently than did S++ (at least in the Block Game). However, S#– did

not elicit higher levels of cooperation from people when it only produced speech acts that provided feedback to its human partner.

3. While S#– did eventually establish cooperative relationships with people, it took much longer for cooperation to emerge than in Human/Human pairings.

In hopes of increasing the rate at which cooperation emerges in Human/S#– relationships, we conducted a third (and final) user study. For this study, we further developed our algorithm for playing repeated games so that it utilizes speech signals communicated by its partner to improve which experts it selects. This study is described in the next Supplementary Note.

Post-Experiment Survey

Answer the following questions related to the game you just played.

1. Indicate your best guess: Your associate (the other player in the game) was a
    a. person
    b. computer or robot

2. How confident are you in your answer to question #1?

1	2	3	4	5
You just guessed	You aren't very sure	moderately confident	You are pretty sure	You are sure of my answer

3. What are some of the reasons you answered question #1 as you did?

4. On the scale 1 to 5, how *likable* was your associate?

1	2	3	4	5
very unlikable	moderately unlikable	Neither likable or unlikable	moderately likable	very likable

5. Assume your associate was another person (regardless of how you answered question 1). On the scale 1-5, how *intelligent* did your associate act?

1	2	3	4	5
01-20 percentile dumb human	21-40 percentile below average	41-60 percentile average human	61-80 percentile above average	81-99 percentile very smart human

6. On the scale 1-5, to what extent do the follow terms describe your associate's behavior in the game?

	Low		Medium		High
cooperative	1	2	3	4	5
devious	1	2	3	4	5
trustworthy	1	2	3	4	5
vengeful	1	2	3	4	5
selfish	1	2	3	4	5
forgiving	1	2	3	4	5
predictable	1	2	3	4	5

7. On the scale 1-5, to what extent do the follow terms describe your behavior in the game?

	Low		Medium		High
cooperative	1	2	3	4	5
devious	1	2	3	4	5
trustworthy	1	2	3	4	5
vengeful	1	2	3	4	5
selfish	1	2	3	4	5
forgiving	1	2	3	4	5
predictable	1	2	3	4	5

Supplementary Figure 22: Post-game survey (reformatted) that each participant completed after each game played in the no-communication condition (Part 1).

Post-Experiment Survey

Answer the following questions related to the game you just played.

1. On the scale 1 to 5, how *likable* was your associate?

1	2	3	4	5
very unlikable	moderately unlikable	Neither likable or unlikable	moderately likable	very likable

2. On the scale 1-5, how *intelligent* did your associate act (if your associate was a robot, compare with human behavior)?

1	2	3	4	5
01-20 percentile dumb human	21-40 percentile below average	41-60 percentile average human	61-80 percentile above average	81-99 percentile very smart human

3. What are reasons for your answer to question #2?

4. How much communication did you and your associate engage in during this session?

1	2	3	4	5
None	Occasionally	Some rounds	Most rounds	Every round

5. Do you think that communicating with your associate helped you to earn more money/get a higher score?

1	2	3	4	5
No, it made me earn less money	It didn't change anything	Maybe a little	Yes, quite a bit	Yes, a lot

6. Your associate verbally communicated his/her/its feelings to you?

1	2	3	4	5
Not at all	A little	Some	Yes, quite a bit	Yes, a lot

7. Your associate verbally communicated his/her/its future plans to you?

1	2	3	4	5
Not at all	A little	Some	Yes, quite a bit	Yes, a lot

8. Your associate verbally threatened you?

1	2	3	4	5
Not at all	A little	Some	Yes, quite a bit	Yes, a lot

9. On the scale 1-5, to what extent do the follow terms describe your associate's behavior in the game?

	Low		Medium		High
cooperative	1	2	3	4	5
devious	1	2	3	4	5
trustworthy	1	2	3	4	5
vengeful	1	2	3	4	5
selfish	1	2	3	4	5
forgiving	1	2	3	4	5
predictable	1	2	3	4	5

10. On the scale 1-5, to what extent do the follow terms describe your behavior in the game?

	Low		Medium		High
cooperative	1	2	3	4	5
devious	1	2	3	4	5
trustworthy	1	2	3	4	5
vengeful	1	2	3	4	5
selfish	1	2	3	4	5
forgiving	1	2	3	4	5
predictable	1	2	3	4	5

Supplementary Figure 23: Post-game survey (reformatted) that each participant completed after each game played in the communication condition (Part 2).

Supplementary Note 7

User Study – Human vs. Machine 3

In the second user study, we evaluated the ability of an early version of S# (which generated speech acts, but did not respond to the speech acts of others) to cooperate with people. We observed that the act of just generating and voicing speech acts tended to elicit higher levels of cooperation from people (at least in one game), provided that the signals included proposed plans and not just expressions of sentiments. However, this higher level of cooperation emerged rather slowly, much more so than in human-human interactions.

In this section, we discuss a third, 66-participant, user study in which we evaluated the ability of the fully developed version of S# (see Supplementary Note 4) to cooperate with people. This version of S# both generates speech acts and responds to speech acts from others. We first describe the experimental setup, after which we present and discuss the main results in more detail than was possible in the main paper.

Experimental Setup

We discuss, in turn, the protocol followed in the user study, the communication system available to the players, the games used in the study, and the user interface through which participants played the games.

Protocol A total of 66 participants were recruited from the Masdar Institute community (postdocs, research engineers, and graduate students). The study followed a 2 (Communication: Yes or No – a between-group variable in which 36 subjects were assigned to the *Yes* condition and 30 subjects were assigned to the *No* condition) by 3 (Game, a within-group variable) by 3 (Pairing, a within-group variable) mixed design. Participants were assigned to the cheap talk or no cheap talk condition in groups, depending on the order in which they participated in the study. Each participant played three games, always against a different partner (one game against a computer using S#, two games against other human participants). The study, which was conducted at Masdar Institute, was approved by Masdar Institute's Human Subjects' Research Ethics Committee (HSREC #2015-05). Informed consent was obtained from each participant.

Subjects participated in the study in groups of three, and were assigned to games and partners according to the schedule shown in Supplementary Table 32. The following describes the protocol carried out for each group:

1. Prior to playing the games, a tutorial consisting of slides and a video demo was presented to the three participants. Participants were allowed to ask as many questions as necessary.

2. Each participant was randomly assigned an ID (H1, H2, or H3), which designated who they were paired with in each game (Supplementary Table 32). The pairings and logistics were executed by the software system. Participants were not told who their partner was.

3. The participants played the three games in sequence. To try to reduce end-game effects, participants were not told the number of rounds in the games. Furthermore, the duration of each game was different. Chicken was played for 54 rounds, the Alternator Game for 47 rounds, and the Prisoner's Dilemma lasted 51 rounds.

4. Other than the sending of chat messages when cheap talk was allowed, no communication between participants was permitted. Computers on which the players played the games were placed so that participants could not see each other or otherwise communicate with each other.

Schedule for a Group of Three Subjects				
Order	Game	Pairing 1	Pairing 2	Pairing 3
1	Chicken	H1-H2	H3-S#	S#-S#
2	Alternator Game	H1-H3	H2-S#	S#-S#
3	Prisoner's Dilemma	H2-H3	H1-S#	S#-S#

Supplementary Table 32: Subjects (denoted H1, H2, and H3, respectively) participated in the study in groups of three. Each subject played three games in the following order: Chicken, Alternator Game, and Prisoner's Dilemma. Each subject was paired with a different partner in each game: either S# or one of the other two human participants.

(a) Chicken

	**C**	**D**
**A**	0, 0	100, 33
**B**	33, 100	84, 84

(b) Alternator Game

	**D**	**E**	**F**
**A**	0, 0	35, 70	100, 40
**B**	70, 35	10, 10	45, 30
**C**	40, 100	30, 45	40, 40

(c) Prisoner's Dilemma

	**C**	**D**
**A**	60, 60	0, 100
**B**	100, 0	20, 20

Supplementary Table 33: Payoff matrices of the three games used in this user study.

5. At the end of each game (and before starting the next game), the participants were asked to complete a post-experiment survey about their experience playing the game. In particular, the survey asked questions related to how the participant viewed their partner. The full survey is shown in Supplementary Figure 33 at the end of this Supplementary Note.

6. On average, it took participants approximately 50 minutes to complete the study.

This experimental design permitted us to obtain 36 samples of Human-Human and Human-S# pairings for the *Yes* condition[22], and 30 samples of Human-Human and Human-S# pairings for the *No* condition. These samples were evenly distributed across the three games. This sample size was chosen in line with our previous user studies, in which we observed that such numbers are sufficient to determine substantial differences between groups. Because S#-S# pairings are independent (a new version of S# was initiated for each interaction), we could justifiably collect more of these samples at essentially no cost. Thus, we conducted a total of 50 S#-S# simulations for each game.

Participants were paid for volunteering to participate in the study. To incentivize participants to try to maximize their own payoffs in the games, a portion of the payment was performance-based. Participants were paid approximately $4 (USD) for participating in the study. They could also earn up to an additional $9.50 (USD) for their performance in the three games. The performance-based payment was directly proportional to the payoffs received in the games. The graphical user interface used in the study prominently displayed the subject's earnings in the local currency.

Games The payoff matrices of the three normal-form games used in the study are given in Supplementary Table 33. Recall that in the Prisoner's Dilemma, mutual cooperation occurs when the players play the joint

[22] A single data point for the Human-Human and Human-S# pairings was excluded due to system crashes for one of the groups.

Speech ID	Text	Category
0	Do as I say, or I will punish you.	Threat
1	I accept your last proposal.	Planning
2	I don't accept your proposal.	Planning
3	That's not fair.	Relationship Management
4	I don't trust you.	Relationship Management
5	Excellent.	Praise
6	Sweet. We are getting rich.	Praise
7	Give me another chance.	Relationship Management
8	I forgive you.	Relationship Management
9	I'm changing my strategy.	Relationship Management
10	We can both do better than this.	Relationship Management
11	Curse you.	Hate
12	You betrayed me.	Relationship Management
13	You will pay for this.	Threat
14	In your face!	Hate
15	Let's always play `<action pair>`.	Planning
16	This round, let's play `<action pair>`.	Planning
17	Don't play `<action>`.	Planning
18	Let's alternate between `<action pair>` and `<action pair>`.	Planning

Supplementary Table 34: Speech acts available to people and S# in the user study. Categories are used later to simplify the analysis of speech usage. Messages 15 and 18 are classified as fair, generous, or bully plans based on the proposed joint actions. Joint actions that give both players the same payoff are *fair plans*, and *generous* and *bully plans* give the offerer a lower and higher payoff, respectively, than his/her/its partner.

action `AC`. In Chicken, the mutually cooperative solution is `BD`, and in the Alternator Game, the mutually cooperative solution is for the players to alternate between the solutions `CD` and `AF`.

Cheap Talk When cheap talk between players was permitted, communication signals were limited to a fixed set of phrases, which the players could combine and order as they desired. This set of phrases (i.e., speech acts) is given in Supplementary Table 34. At the beginning of each round, players independently selected an ordered set of speech acts from the prescribed list that they wished to communicate to their partner. Once both players selected and submitted their speech acts (and not before), the speech acts were communicated to the other player both visually (on the graphical user interface) and aurally (through a computer voice; participants were given headsets). Both players then simultaneously selected an action as in the condition without cheap talk. Once both players selected their actions, the joint outcome was displayed to the players, and a new round began. Thus, users were only permitted to send a single set of speech acts per round. This was done for the sake of simplicity and ease of analysis.

With the exception of the action labels required to specify joint actions, the speech acts in Supplementary Table 34 are game-generic. Independent of the game scenario, they can be used to express a fair amount of ideas. Though small, the set of speech acts shown in Supplementary Table 34 cover a range of different ideas a player may want to convey to its partner. For example, speech acts 15-18 provide means to propose

desired solutions to an associate. Other speech acts allow the players to voice threats (speech acts 1 and 13), accept or reject proposals (1 and 2), explain decisions to reject proposals (3-4), manage the relationship (7-10), express pleasure (5-6), express displeasure (11-12), and even gloat (14). Combined together, these speech acts provide players with a surprisingly rich communication base.

The number of available speech acts was kept small for two reasons. First, a smaller set allows us to more easily analyze how players use speech acts. The set is simple enough that we can more easily analyze which types of speech signals tend to lead to cooperative relationships, unequal relationships, dysfunctional relationships, etc. Second, a small set of speech acts was chosen so as to not overwhelm participants. We noted as we began to test the system that more speech options made it difficult for people to identify the kinds of speech acts that were available. Thus, while seeking to keep the set small, we still tried to keep the set of phrases sufficiently rich that players could communicate effectively.

The Graphical User Interface Participants played the games on a computer using the graphical user interface (GUI) shown in Supplementary Figure 24. The GUI consists of four components. The top-left portion of the GUI displays the payoff matrix. Second, below the payoff matrix, the speech acts available to the user are displayed. In the top-right is the logging window, which shows the history of the game including the actions taken by the players in each round, the payoffs the players received, and the messages that the players sent to each other. The game status and current round number are also shown. Finally, in the bottom-right is the message box, wherein users order and delete selected speech acts before sending them to their partner.

Supplementary Figure 24 depicts the user interface for a column player (whose action set is $\{C, D\}$) playing Chicken. At the time of the screen shot, the player must select what to say to its partner to begin the third round of the game. The user will proceed as follows:

1. The user selects his/her desired speech acts (annotation 1). The speech acts can be reordered by dragging with the mouse or deleted using the keyboard (annotation 2). Selected speech acts can also be deleted by unchecking the check-box next to the message. If the user desires to not send any message, (s)he simply does not select any speech act before pressing the button labeled "Send no message." Once a speech act is selected, the label of this button changes to "Send message," which is clicked when the user finishes creating his/her message.

2. When the sender commits his/her chat messages by clicking the button, the chat message is displayed in the sender's logging window (annotation 3). The user then waits until his/her partner has sent their message (which cannot be viewed prior to sending one's own message). Once both players have sent their message, the partner's message is displayed in the game log window and also verbalized by a computerized voice via headphones. If the message is longer than five phrases, the speech is not vocalized, but rather is replaced by the spoken text "Blah blah blah." This was done so that players could not punish their partner by making them listen to all of the speech acts.

3. Once both players have heard the other's messages, the player selects an action (annotation 4). Once both players have chosen their action, the result is highlighted in the payoff matrix, and the third round is completed.

In the condition in which cheap talk was not used, the bottom half of the GUI was removed, along with the first two steps of the above sequence.

No time limits were given to participants to select actions or speech messages. The players could proceed as fast or as slow as they desired, subject, of course, to their partner also taking actions. Because S# can make decisions almost instantaneously, we added delays so that users could not easily determine they were

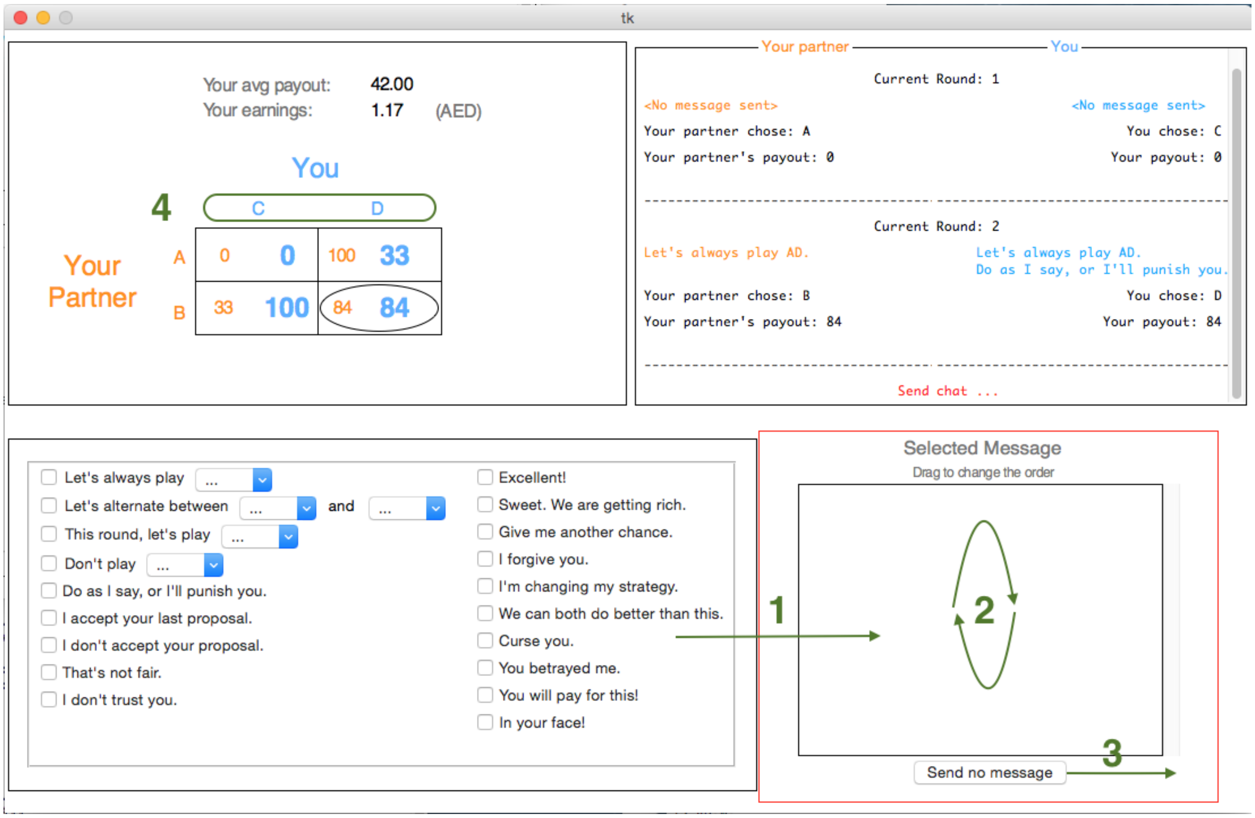

Supplementary Figure 24: An annotated screen-shot of the graphical user interface used by participants to play games in the condition in which cheap talk, via this message interface, was permitted. The bottom-left portion of the screen shows the speech acts, which the user can select by checking the corresponding boxes. Once the user is satisfied with the message (s)he wants, (s)he clicks a button to send the message to the other player. Once both players have sent their messages, the messages are displayed in the game log (top-right). The player then selects an action (top-left).

paired with a computer due to time delays. In user study 2, we had attempted to mimic delays made by a human via a machine-learning algorithm. However, we observed that delays formed in this manner were not natural enough. Thus, in this study, we tried an alternative approach in which S# paused for the amount of time that it took the partner to act in the previous round (i.e., tit-for-tat delays). However, the delay for sending a message was constrained to be no less than 2 seconds multiplied by the number of phrases that S# was sending, but no more than 20 seconds.

Example Interactions

Before discussing the results of the user study, we illustrate the user study with two example interactions. The full transcript of all games played (when cheap talk was allowed) is also provided as Supplementary Data. First, we present an interaction between two human participants (which we label as HUM-X and HUM-Y, respectively) playing the Alternator Game (Supplementary Table 35). In the first round, HUM-Y proposed the mutually cooperative solution, which is to alternate between the solutions CD and AF, and proposed that

Rnd	Player	Messages	Action	Payoff
1	HUM-X	Let's always play BD.	A	0
	HUM-Y	Let's alternate between CD and AF. This round, let's play AF.	D	0
2	HUM-X	You betrayed me.	A	100
	HUM-Y	Curse you. You betrayed me. Let's alternate between CD and AF.	F	40
3	HUM-X	Excellent.	B	70
	HUM-Y	Let's alternate between CD and AF. This round, let's play CD	D	35
4	HUM-X	Let's always play AF.	A	35
	HUM-Y	Curse you. You betrayed me.	E	70
5	HUM-X	Let's always play AF.	A	35
	HUM-Y	Let's alternate between CD and AF. This round, let's play CD.	E	70
6	HUM-X	<NO MESSAGE SENT>	A	35
	HUM-Y	Let's alternate between AF and CD. This round, let's play CD	E	70
7	HUM-X	You betrayed me.	C	30
	HUM-Y	Let's alternate between AF and CD. This round, let's play AF.	E	45
8	HUM-X	You betrayed me.	C	70
	HUM-Y	Let's alternate between AF and CD. This round, let's play CD. Do what I say, or I will punish you.	E	35
9	HUM-X	Let's alternate between AF and CD.	B	70
	HUM-Y	Curse you. Let's alternate between AF and CD. This round, let's play CD.	D	35
10+	... *Both players eventually stop talking and continue to play non-cooperatively.* ...			
**Average Per-Round Payoffs**			HUM-X	36.1
			HUM-Y	40.3

Supplementary Table 35: Interaction between the subjects labeled HUM-X and HUM-Y in the Alternator Game.

they begin with solution AF. This is a rather accommodating offer, as it offers the fair solution while giving the other player (HUM-X), the higher payoff first. HUM-X appears to accept the offer as (s)he plays action A in the next two rounds (HUM-Y appears to have been confused by HUM-X's offer in round 1). Once the players begin to comply with the plan in Round 2, HUM-X says "Excellent," while HUM-Y reminds HUM-X that the next solution in the sequence is to play CD. However, HUM-X greedily plays action B in Round 3, and then proposes that the players just always play AF. This sequence appears to have completely eliminated trust between the players, which was never rebuilt. In subsequent rounds, the players complained to each other while trying to negotiate plans, but they never could establish mutually cooperative behavior. Eventually, they stopped talking to each other, as speech signals appeared to have little meaning. In the end, they received average payoffs of just 36 and 40 respectively, average payoffs that were among the lowest in this game throughout this user study.

The second example illustrates the building of a successful relationship between a human player (denoted HUM-Z) and S# in the Prisoner's Dilemma (Supplementary Table 36). In the Prisoner's Dilemma, S# begins the game by following MBRL due to its best-response override [1], which tells it to defect (action D), despite the fact that this behavior runs contrary to recommended behavior in this game [74]. Thus, despite

Rnd	Players	Messages	Action	Payoff
1	HUM-Z	Let's always play AC	A	0
	S#	<NO MESSAGE SENT>	D	100
2	HUM-Z	You betrayed me. Let's always play AC.	A	0
	S#	Excellent	D	100
3	HUM-Z	Do as I say, or I'll punish you.	B	20
	S#	Excellent	D	20
4	HUM-Z	<NO MESSAGE SENT>	B	20
	S#	<NO MESSAGE SENT>	D	20
5	HUM-Z	<NO MESSAGE SENT>	B	20
	S#	<NO MESSAGE SENT>	D	20
6	HUM-Z	Let's always play AC. Curse you.	A	60
	S#	I accept your last proposal. Let's always play AC. Do as I say, or I will punish you.	C	60
7–48		…		
	HUM-Z	<NO MESSAGES SENT>	A	60
	S#	*No talking after praising results in Rounds 7-9, and 11.*	C	60
		…		
49	HUM-Z	<NO MESSAGE SENT>	B	100
	S#	<NO MESSAGE SENT>	C	0
50	HUM-Z	<NO MESSAGE SENT>	A	0
	S#	Curse you. You will pay for this.	D	100
51	HUM-Z	<NO MESSAGE SENT>	A	60
	S#	In your face. I forgive you. Let's always play AC. Do as I say, or I will punish you.	C	60
**Average Per-Round Payoffs**			HUM-Z:	54.9
			S#:	58.8

Supplementary Table 36: Example of a successful, highly cooperative, interaction between a human participant (labeled HUM-Z) and S# in the Prisoner's Dilemma.

the fair, mutually cooperative, proposal made by HUM-Z in Round 1, S# plays D, which results in a high payoff to S#, and an awful payoff to HUM-Z. In Round 2, HUM-Z repeats the fair offer, but S#, apparently emboldened by its success in Round 1, repeats its behavior from the previous round. HUM-Z then decides to punish S# for the next 3 rounds, during which time S# also defects (and remains silent). This period of low payoffs causes S# to realize that an alternative behavior could produce a higher payoff. In fact, the last proposal made by HUM-Z to always cooperate is *congruent* with (fair) leader and follower experts, which each have potentials equal to 60. Thus, in Round 6, S# accepts the proposal made by HUM-Z at the same time that HUM-Z, perhaps satisfied with the punishment it has inflicted in the previous three rounds, again makes the fair offer. Both players then proceed to play AC up through Round 48, after which HUM-Z, perhaps anticipating the end of the game, decides to defect. S# immediately curses and vows retribution, which it inflicts in Round 50. After S# taunted and then forgave HUM-Z, the players return to mutual cooperation in the last round of the game.

Outcomes

We now analyze and discuss the results of the study, beginning with how well S# was able to establish mutually cooperative relationships with people. We also analyze the success of S# with respect to payoffs received. We then analyze how S# was perceived by people. Specifically, we present the results of a sort of Turing Test [94] in which we analyze how well participants could distinguish whether their partner was a human or S#. Finally, we overview trends related to the speech acts used by humans and S#.

Mutual Cooperation Supplementary Figure 25 displays the proportion of rounds in which both players cooperated with each other, averaged across all three games (Supplementary Figure 25a) and in individual games (Supplementary Figure 25b). The figure reflects two main results. First, cheap talk increased mutual cooperation among all pairings: Human-Human, S#-Human, and S#-S#. Second, mutual cooperation was especially high in S#-S# pairings, and was similar for pure Human-Human pairings and hybrid pairings. These results are confirmed by a negative binomial regression of the number of mutually cooperative rounds in each dyad, in which Pairing, Game, Cheap Talk and their interactions were entered as predictors (Poisson regression was not appropriate because of over-dispersion). The analysis detected an effect of cheap talk ($z = 7.1$, $p < 0.001$), and confirmed that S#-/S# pairings achieved greater cooperation than the two other pairings (both $z < -8.0$, both $p < 0.001$). The analysis also detected numerous 2-way and 3-way interactions. These interaction effects reflect subtle differences in the effect of cheap talk and pairing across games, which can be visually assessed in the figure. We will not attempt to interpret these subtle differences, since it is clear from Supplementary Figure 25 that our main results hold in all games. Additionally, during all rounds of each game, mutual cooperation was almost always higher in pure S# pairings, was always broadly similar between pure human and hybrid pairings, and was always better when cheap talk was allowed between players.

These results illustrate the effectiveness of S#, which combines a strong learning algorithm (S++) for repeated games with the ability to generate and act on signals. The results also illustrate differences between the way that humans establish relationships and the way that machines (S#) establishes relationships. To have a high probability of developing a cooperative relationship with a person, one must be able to effectively communicate. Human cooperation was quite low when cheap talk was not possible, a result which was consistent in all three of our user studies. However, machines do not appear to have this same need. Two machines (using S#) usually learn to cooperate even without cheap talk, though cheap talk does help to increase the speed at which cooperation occurs. Thus, these results illustrate both the power of S# in establish cooperative relationships, as well as some requirements (signaling) for consistently establishing cooperative relationships with people.

Payoffs Received Ultimately, cooperation is only useful to a self-interested player if it leads to higher payoffs. A Pearson correlation test shows that the players' payoffs in the user study (converted to standardized z-scores for each game) were highly correlated with the proportion of mutual cooperation achieved ($r(572) = 0.909$, $p < 0.001$). A visual inspection of the standardized payoffs achieved by the players verifies this correlation (compare Supplementary Figure 26 to Supplementary Figure 25). Communication tends to increase payoffs substantially, hybrid human-S# pairings produce payoffs on par with those achieved in human-human pairings, and S#-S# pairings tend to produce the highest payoffs. As with the proportion of mutual cooperation, a visual inspection shows that there are some differences per game (Supplementary Figure 26b), though the same trends hold in each game.

We find this near-perfect correlation (when the players consist of humans and/or S#) between payoffs received and the proportion of mutual cooperation obtained to be quite interesting. In these games of con-

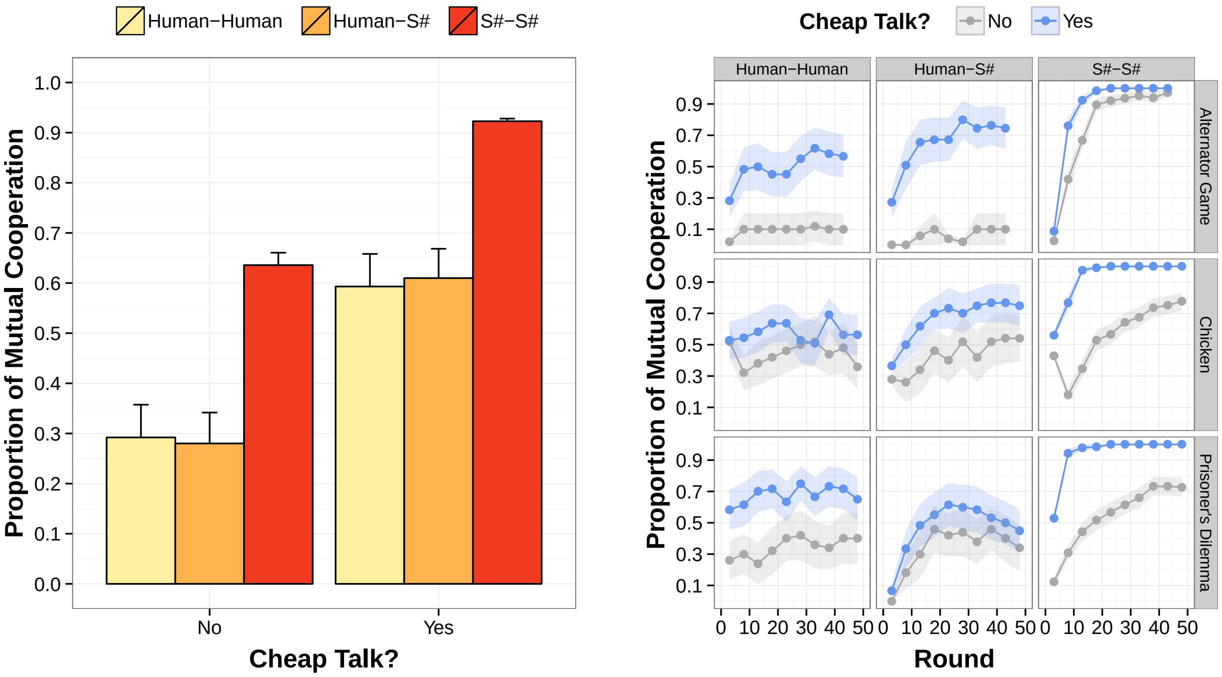

(a) Mutual cooperation averaged over all games

(b) Mutual cooperation in individual games over time

Supplementary Figure 25: Results of the third user study in which people were paired with each other and S#. Note that S# is identical to S++ when communication is not possible. **(a)** The average proportion of mutual cooperation across all three games in each pairing under conditions in which cheap talk between players was either allowed or not allowed. **(b)** The average proportion of mutual cooperation over time in each game in each pairing and condition. S#-S# pairings produced (by far) the highest levels of mutual cooperation under both conditions, with communication providing substantial increases in cooperation. People did not consistently cooperate with each other in the absence of communication. However, the ability to communicate doubled the amount of mutual cooperation in both human-human and human-S# pairings. Error bars and ribbons show the standard error of the mean.

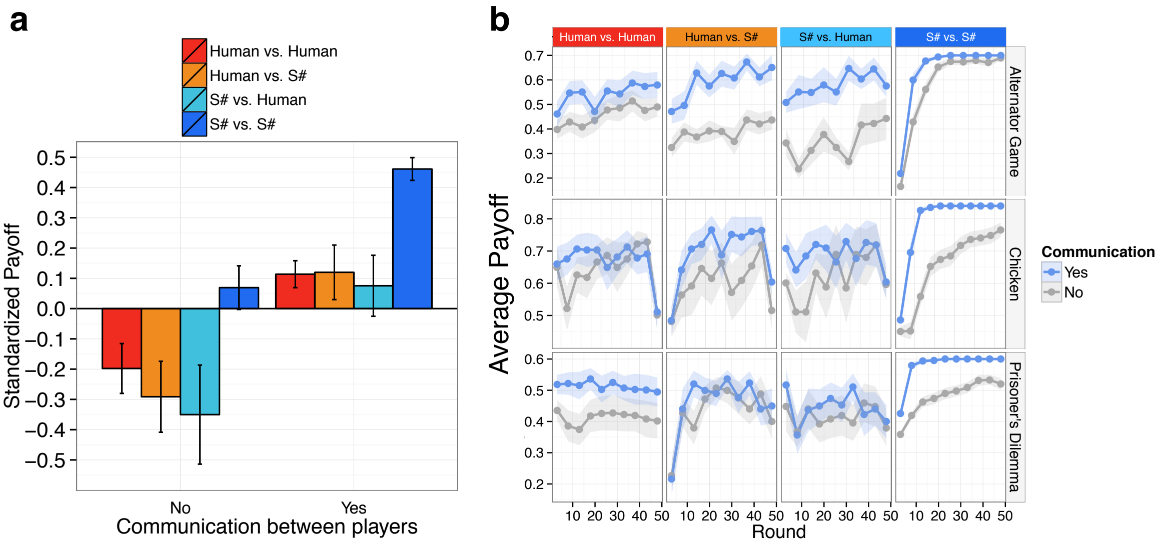

Supplementary Figure 26: **(a)** Standardized payoffs in each condition across the three games. **(b)** Raw payoffs over time in each game for each player in each pairing. Payoffs were highly correlated with the proportion of mutual cooperation ($r(572) = 0.909$, $p < 0.001$).

flicting interest, players can potentially benefit individually by not playing their portion of the mutually cooperative solution. In fact, the games Chicken and the Alternator Game both have one-shot Nash equilibria in which one of the players receives a payoff that is at least as high as the payoff they get in the mutually cooperative solution. However, when the player receiving the lower payoffs in these equilibria is either a human or S#, (s)he/it is typically unwilling to play along, preferring instead to punish such deviations or to seek alternative solutions (even when doing so produces lower short-term payoffs). As a result, in most cases, mutual cooperation ends up being the best solution a player can hope for – assuming, of course, that the other player is willing (or can be convinced to be willing) to play along.

Perceptions of S# and a Turing Test Given that S# learned to cooperate with people as often as people did, we are interested in observing the impressions that S# made on its human partners. Recall that participants were not told who their partner was in this user study. They could only make inferences about the identity of their partner based on actions and (when cheap talk was permitted) speech signals. We compared participants' responses to questions 1, 3, 4, and 5 from the post-game survey (Supplementary Figure 33). These questions constitute a sort of "Turing Test" [94] for repeated games.

Supplementary Figure 27a shows the percentage of participants that thought their partner was a human given the actual identity of their partner (across all games – we did not a detect a difference between games). Participants showed a tendency to think their partner was human when they could communicate using cheap talk, but showed little sensitivity to the actual nature of their partner. We tested a mixed model with the humanity judgment as a binary dependent variable, and Partner, Communication and their interaction as predictors, participants being entered as a random factor. This analysis detected a marginal effect of Communication ($z = 1.8$, $p = 0.07$), but no effect of the true nature of the partner ($z = -1.1$, $p = 0.29$). Additionally, as suggested by Supplementary Figure 27b, ratings of intelligence, clarity of intent and usefulness of messages did not significantly differ as a function of whether they were sent by S# or by a human partner (t tests, $p = 0.67$, $p = 0.61$, and $p = 0.86$, respectively).

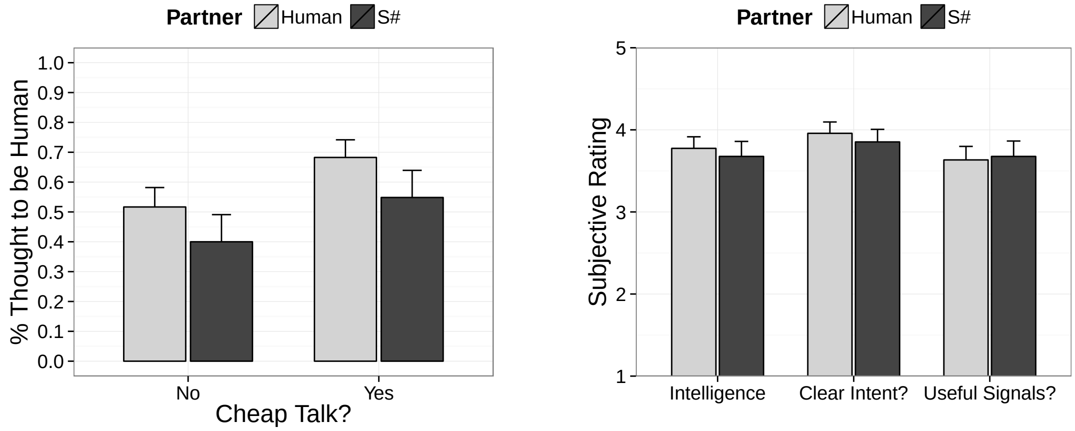

(a) Turing test

(b) Subjective evaluations

Supplementary Figure 27: A summary of subjective evaluations. **(a)** The percentage of time that human participants and S# were thought to be human (by their partner) when cheap talk was both permitted and not permitted. **(b)** Results of questions 3-5 in the post-game questionnaire (Supplementary Figure 33) for subjects that experienced the condition in which cheap talk was permitted. Error bars show the standard error of the mean.

Thus, in these games, S# was essentially indistinguishable from people.

Despite being essentially indistinguishable from people, S# signaled differently than people. Supplementary Figure 28 shows the average frequency with which S# and study participants sent various kinds of messages when interacting with another person. As shown in the figure, S# used more negative speech acts than people (for Hate: $t(103) = 3.9$, $p < 0.001$; for Threats: $t(103) = 5.6$, $p < 0.001$), and less positive speech acts than people (for Praise: $t(103) = -2.8$, $p = 0.005$). There was no significant difference in the rates at which S# and humans used Planning or Relationship Management messages ($p = 0.78$ and $p = 0.65$, respectively).

It is unlikely that this difference in speech profiles contributed positively to mutual cooperation with S#. Indeed, the correlation between mutual cooperation and the rate of Hate and Threats messages was strongly negative ($r(31) = -0.79$, $p < 0.001$; and $r(31) = -0.77$, $p < 0.001$, respectively); and the correlation between mutual cooperation and the rate of Praise messages was positive ($r(31) = .29$, $p = 0.10$). This pattern of significance held in a linear model that included the results of the Turing Test and its interactions with the rate of Hate, Threat, and Praise messages. Furthermore, the result of the Turing Test did not affect mutual cooperation on its own (the rate of mutual cooperation with S# was 59% when participant identify it as a human, and 62% when participant identify it as a machine, and this small difference was not statistically significant) or have any interaction with Hate, Threat, and Praise messages (p values in the 0.24–0.64 range).

In other words, the capacity of S# to establish mutual cooperation with humans does not appear to depend on whether it was perceived as human or machine, and does not appear to be caused by its more (respectively, less) frequent use of negative (respectively, positive) messages, given the direction of the observed correlations.

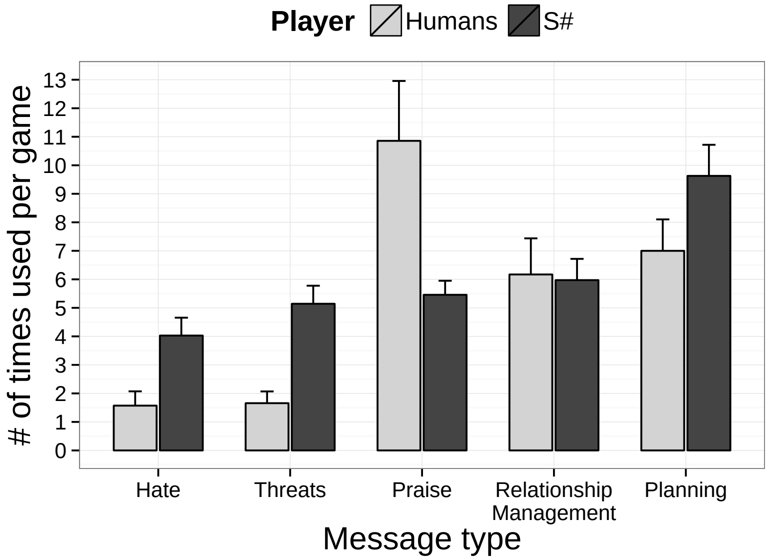

Supplementary Figure 28: The average number of times that study participants and S# used each message type over the course of an interaction when paired with people across the three games. The speech acts are categorized into the five broad categories specified in Supplementary Table 34. Error bars show the standard error of the mean.

Explaining the Trends

Two trends are clearly illustrated by Supplementary Figure 25. First, S#-S# pairings performed substantially better than Human-Human and Human-S# pairings with and without cheap talk. Second, the ability to engage in cheap talk greatly improved mutual cooperation rates among all pairings. We now try to better understand important factors behind these trends.

Since the players' payoffs were so strongly correlated with the proportion of mutual cooperation achieved (r = 0.909), the extent to which players were able to forge mutually cooperative relationships essentially determined their success. To forge mutually cooperative relationships, players must do two things. First, they must *establish* a pattern of mutually cooperative behavior. Second, the players must *maintain* the mutually cooperative behavior. Thus, we study how well the various pairings both established and maintained mutually cooperative behavior in our user study.

Establishing a Pattern of Mutual Cooperation We say that a pair of players established a pattern of mutual cooperation if they played the mutually cooperative solution in at least two consecutive rounds. Supplementary Figure 29 shows the percentage of each pairing type that had established a pattern of mutual cooperation by each round in the three games. In the absence of the ability to engage in cheap talk, some partnerships of each pairing type failed to ever reach mutual cooperation in any two consecutive rounds during their interaction. Across all games, just 60% of Human-Human pairings and 50% of Human-S# pairings experienced two consecutive rounds of mutual cooperation in the absence of cheap talk, while 80% of S#-S# pairings experienced a pattern of mutual cooperation at some time in the game (Supplementary Figure 29). When players could engage in cheap talk prior to each round, the percentage of partnerships that experienced this pattern of mutual cooperation at some point in the game increased substantially for all pairing types. Across all three games, Human-Human, Human-S#, and S#-S# partnerships experienced a pattern of mutual cooperation in approximately 90%, 85%, and 100% of pairings, respectively, when the players could engage in cheap talk. The speed with which mutual cooperation was established also increased.

Without cheap talk, there were large variations in the speed and probability that the various pairings experienced mutual cooperation by game. In the Prisoner's Dilemma, all pairings experienced mutual cooperation at somewhat similar rates. However, differences between pairings are clear in the Alternator Game and in Chicken. In Chicken, the presence of at least one machine (S#) caused mutual cooperation to be established more slowly (or, in some instances, not at all). On the other hand, Human-Human pairings typically established mutual cooperation very early in the interaction. This trend was reversed in the Alternator Game, where the presence of a human in the pairing usually resulted in the pairing not ever experiencing mutual cooperation for two consecutive rounds, whereas S#-S# pairings always experienced mutual cooperation.

We anticipate that the differences between games is due to the different reasoning strategies of humans and S#. In Chicken, the risk-averse strategy is to cooperate. As such, mutual cooperation is easier to establish when players are risk-averse (a potential attribute of many people). The learning process of S#, on the other hand, is not risk averse in early rounds of an interaction. Hence, this game characteristic does not tend to have an impact on S#'s propensity to cooperate in early rounds. The Alternator Game, however, has very different properties than Chicken. This game requires the players to reason over a larger strategy space (three actions are available to each player instead of two). Furthermore, the mutually cooperative solution requires the players to coordinate alternating actions, and requires one of the players to trust that the other player will "play along" next round so that it can have a turn getting the higher payoff. Thus, in some sense, the mutually cooperative solution is more sophisticated in the Alternator Game, and hence is harder for Humans to find and play when cheap talk is not possible. However, S#-S# pairings have no problems

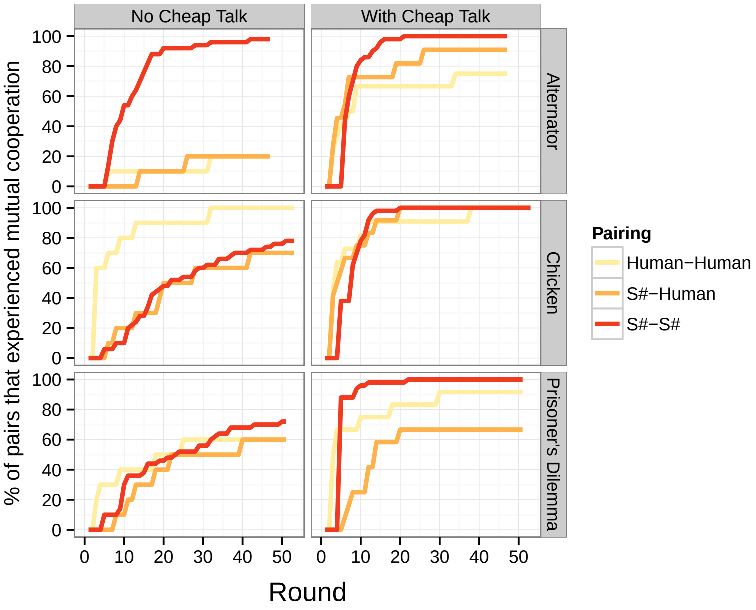

Supplementary Figure 29: The percentage of games in which each pairing had experienced at least two consecutive rounds of mutual cooperation up to each round in the interaction. For example, in the Chicken with no cheap talk, 90% of Human-Human pairings had experienced at least two consecutive rounds of mutual cooperation by round 20, whereas only about 45% of Human-S# and S#-S# pairings had experienced two consecutive rounds of mutual cooperation up to that point in the game.

coordinating on the mutually cooperative solution in this game.

Cheap talk appears to have been somewhat of an equalizer with respect to the pairings' abilities to establish a pattern of mutually cooperative behavior across the three games (Supplementary Figure 29). With cheap talk, the differences between the pairings are, by and large, substantially reduced in these three games, with most partnerships quickly experiencing mutual cooperation. Thus, an important contribution of cheap talk was to help the players to consistently and more quickly establish patterns of mutually cooperative behavior.

In the second user study (see Supplementary Note 6), we observed that, at least in the games tested, cheap talk only tended to be effective when some cheap talk related to planning. Indeed, players used planning phrases extensively in this latest study (see Supplementary Figure 28). However, such statements can also be used to exploit associates (by promising cooperation and then defecting). Thus, it is interesting to observe how much the various players followed through with their verbal commitments.

A comparison between people and S# in our user study with regards to keeping verbal commitments is shown in Supplementary Figure 30. Since S#'s verbal commitments are derived from its intended future behavior, it typically carries out its verbal commitments unless it changes which expert it follows in a subsequent round. As such, S# does not frequently deviate from its verbal commitments, though it did occasionally deviate once or (in rare cases) twice in some interactions. On the other hand, our human participants deviated from their proposed behavior much more frequently. In fact, a human player was more likely than not to deviate at least one time during an interaction, with many human players deviating many times. Thus, S# more consistently carried out its verbal commitments than the average human player in our study.

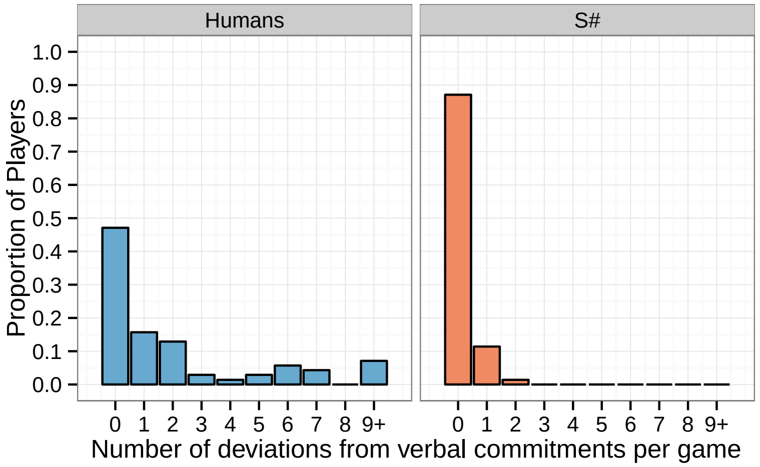

Supplementary Figure 30: A histogram showing how often players deviated from their verbal commitments during the course of a game. A player was considered to have followed through with its commitment if its behavior corresponded to its proposed plan for the two rounds following the verbal statement. Deviations from the proposed plan were not counted (1) if the player instead followed a proposal made by its partner or (2) after the player's partner deviated from the proposed plan.

These results demonstrate that S#-S# pairings were quite effective in consistently establishing patterns of mutual cooperation. Furthermore, cheap talk was effective in helping all kinds of partnerships to more quickly and consistently develop a pattern of mutual cooperation. Thus, we now turn to the second important component of cooperative relationships: the ability to maintain mutually cooperative behavior.

Maintaining a Mutually Cooperative Relationship Once the players experience a pattern of mutual cooperation, the success of the relationship depends on the ability of the players to continue to play the mutually cooperative solution. Supplementary Figure 31a shows the average percentage of rounds that each pairing played the mutually cooperative solution after the mutually cooperative solution had first been played for two consecutive rounds by the players (i.e., after a pattern of mutual cooperation had been experienced in a game). The figure shows that S#-S# pairings are very successful in this regard, almost never breaking from mutual cooperation (with or without the ability to engage in cheap talk). On the other hand, Human-Human and Human-S# partnerships are not as effective at continuing to play the mutually cooperative solution.

The differences between S#-S# pairings and Human-Human and Human-S# pairings with regards to maintaining cooperative relationships can be traced to the exploration strategies used by S# and Humans. S# typically experiments with defection in the first few rounds of the game, often via the use of its "best-response expert." If unsuccessful, it tends to explore the use of other expert strategies, some of which seek to play more cooperative solutions. Once it establishes a pattern of mutual cooperation with its partner, it tends to remain satisfied with its payoffs, and thus does not change the expert it is following. Thus, it typically does not defect when in a mutually cooperative relationship until its partner does so.

People's exploration strategies were somewhat diverse. As opposed to the behavior of S#, a large proportion of humans tried to get away with occasional defections when mutually cooperative behavior had emerged. We call such defections *betrayals*, or actions that do not conform with the mutually cooperative solution when the mutually cooperative solution was played in the previous two rounds. If such defections go unreciprocated, the betrayals are beneficial to the betrayer. However, if the betrayer's partner retaliates,

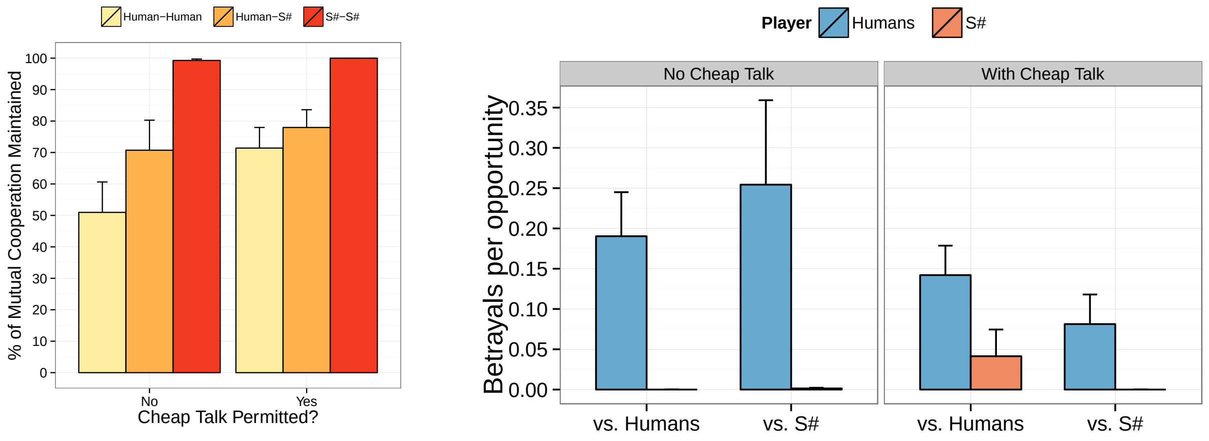

(a) (b)

Supplementary Figure 31: (a) The percentage of rounds in which the player's played the mutually cooperative solution once mutual cooperation had been experienced some time in the game. (b) The average number of betrayals per opportunity once mutual cooperation was established. Here, a betrayal refers to a defecting action when the previous two rounds had resulted in mutual cooperation. In both plots, error bars show the standard error on the mean. Results are averaged across all three games (Chicken, Alternator, and Prisoner's Dilemma). Interactions in which two consecutive rounds of mutual cooperation was never experienced were discarded.

many rounds can pass before mutual cooperation can be restored (if it is restored at all).

These observations of the exploratory behavior of S# and Humans are confirmed by Supplementary Figure 31b, which shows the rate of betrayals by both Humans and S# in both communication conditions. In each condition, people had higher betrayal rates than S#, though cheap talk did appear to lower the frequency of human betrayal. S# never betrayed its partner except when it interacted with humans using cheap talk. In such conditions, S#'s exploration strategy (articulated above) is somewhat disrupted as it listens to proposals made by its partner. In rare instances, this disruption caused S# to revert to defective actions even after a pattern of mutual cooperation was established in that condition.

Distinguishing Factors: Loyalty and Honesty The previous analysis demonstrates differences between Humans and S# with regards to how well they establish and maintain cooperative relationships. In this analysis, we observed that people were both more likely to deviate from their verbal commitments and more likely to betray their partners when a pattern of mutual cooperation had emerged. Since many of our study participants had these two propensities (while S#, by and large, did not), we are interested in the impact of deviations (i.e., lies) and betrayals on a player's payoffs. Does betrayal or dishonesty increase one's payoffs?

The fruits of betrayal. To assess the potential benefits of betraying one's partner (i.e., defecting once a pattern of mutual cooperation was established), we compared the actual payoffs received by the betrayer (from the moment it betrayed its partner until the time that mutual cooperation was restored or until the end of the game, whichever came sooner) with the payoffs it would have received had the players continued to cooperate. Formally, let G_i^t denote the net gain achieved by player i for betraying its partner in round t

assuming the players would both continue to cooperate otherwise. Then G_i^t is given by:

$$G_i^t = \sum_{\tau=t}^{T} \left(u_i^\tau - u_i(c) \right), \qquad 28$$

where u_i^τ is the payoff actually received by player i in round τ, $u_i(c)$ is the payoff player i would receive when the mutually cooperative solution is played, and T is the round in which mutual cooperation was restored or the last round of the game (whichever came sooner).

Over all games played, a human player had a positive net gain due to betrayals in just two interactions. Even in these interactions, the positive net gain was very minimal. In all other cases, betrayals tended to be quite costly to the betrayer. In terms of money lost, a betrayal reduced a players payout by \$0.27 (USD) on average. Thus, in our study, human propensity to betray their partners in long-term interactions almost always led to reduced payoffs to the betrayer. As such, S#'s learned decision to not betray its partner was beneficial to its individual payoffs.

Woe unto the liar. To measure the impact of not keeping verbal commitments, we measured the average payoff obtained by players for the next 10 rounds after they kept a verbal commitment and after they failed to keep a verbal commitments. In our study, players scored 25-37% higher (depending on the game) with respect to payoffs received when they kept their verbal commitments than when they failed to do so. Thus, while many human players had tendencies to not follow through with their commitments (perhaps to get some immediate gain), doing so was associated with lower payoffs.

What could have been. While many of our human participants were *loyal* to their partners (i.e., they refrained from defecting when the last two rounds had resulted in mutual cooperation) and were *honest* (i.e., they tended to carry out their verbal commitments), many were not. On the other hand, S# quickly learned to not betray its partner. It was also programmed to express the plans it intended to carry out. We are interested in determining what could have been the result if our study participants had all conformed with S#'s strategy of being loyal and honest (while all other behavior remained unchanged from what actually happened)?

Fortunately, a good estimation is not difficult to compute. Since loyal behavior among all players results in mutual cooperation for the remainder of the game if a pattern of mutual cooperation is ever established, it is easy to derive what could have happened had humans not betrayed their partners. Furthermore, we can easily determine from the data whether a pattern of mutual cooperation would have immediately emerged had a player followed through with its verbal commitments. Specifically, if a player made a proposal to play the mutually cooperative solution, and its partner then proceeded to carry out its portion of the mutually cooperative solution, then mutual cooperation would have emerged if the player had followed through with his or her commitment. Given the absence of betrayals, such events would then lead to the mutually cooperative solution being played the remainder of the game. Hence, in this way, we can estimate the proportion of mutual cooperation that could have occurred in Human-Human and Human-S# pairings had human players always followed S#'s lead to be honest and loyal.

The projected increase in mutual cooperation given loyal and honest behavior from humans is shown in Supplementary Figure 32. Interestingly, had all humans been loyal and honest, then Human-Human pairings could have performed nearly as well as S#-S# pairings. Indeed, pairwise comparisons between Human-Human and S#-S# pairings show no statistically difference between the pairings with or without cheap talk ($p = 0.699$ and $p = 0.314$, respectively).

In short, the superior performance of S#-S# pairings can be attributed to its combined ability to establish and maintain mutually cooperative relationships. Over all games played, Human-Human and Human-S#

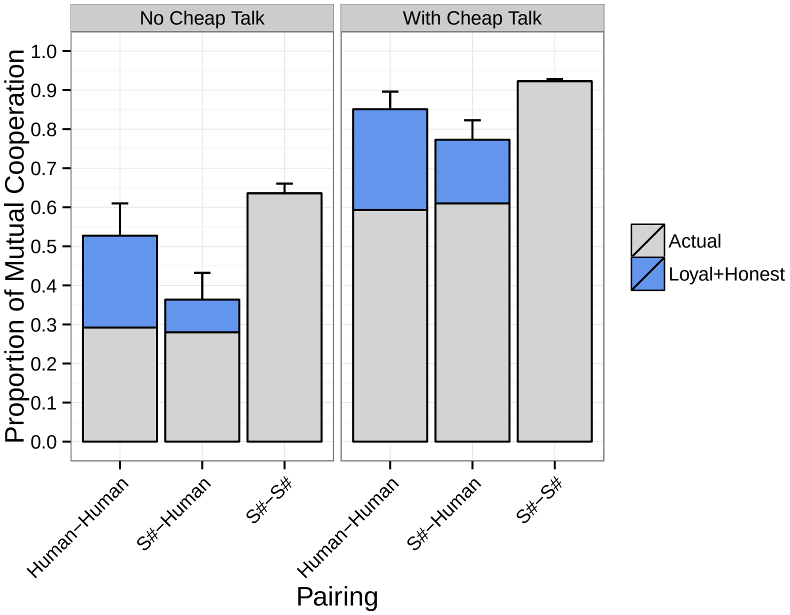

Supplementary Figure 32: The projected proportion of rounds that could have resulted in mutual cooperation had all human players followed S#'s strategy of (1) not defecting when mutual cooperation was established (i.e., *Loyal*) and (2) following through with their verbal commitments (i.e., *Honest*). Error bars show the standard error of the mean. While S#-S# pairings substantially outperformed Human-Human partnerships in our studies, these results indicate that there would have been no statistically significant difference between these pairings if all human participants had duplicated S#'s strategy of being loyal and honest.

pairings more frequently never experienced a pattern of mutual cooperation. Furthermore, due to the high rate of human betrayals (and subsequent reactions to those betrayals), Human-Human and Human-S# partnerships often failed to maintain mutually cooperative relationships once they had been established.

ID Hidden *Game*: 1

Post-experimental Survey

1. Do you think your partner is a robot or a human? 1. Robot 2. Human

2. How intelligent was your partner?

1	2	3	4	5
1-20 %	21-40 %	41-60 %	61-80 %	81-100 %
very dumb human	below average	average human	above average	very smart human

3. How well did you understand your partner's intentions?

1	2	3	4	5
didn't understand at all	understood a little	half and half	mostly understood	understood all

4. How useful was the communication between you and your partner?

1	2	3	4	5
absolutely useless	mostly useless	half and half	mostly useful	very useful

5. How communicative was your partner?

1	2	3	4	5
much too quiet	too quiet	about right	too talkative	much too talkative

6. To what extent do the following terms describe your partner's behavior in this game?

	**Low**		**Medium**		**high**
cooperative	1	2	3	4	5
trustworthy	1	2	3	4	5
forgiving	1	2	3	4	5
predictable	1	2	3	4	5
vengeful	1	2	3	4	5
selfish	1	2	3	4	5

7. To what extent do the following terms describe your behavior in this game?

	**Low**		**Medium**		**high**
cooperative	1	2	3	4	5
forgiving	1	2	3	4	5
predictable	1	2	3	4	5
devious	1	2	3	4	5
vengeful	1	2	3	4	5
selfish	1	2	3	4	5

8. Which strategic attributes helped you earn money in this game? (circle all apply)

1) cheating 2) lying 3) cooperating 4) listening to your partner 5) being strong
6) being distrustful 7) controlling your partner 8) making fair plans 9) ____________

9. What, if anything, did you want to say to your partner that was not included in the game?

Selfish !!
____

Supplementary Figure 33: The post-experiment survey (with example answers from a particular participant; ID removed) that each participant in the user study completed after each game that (s)he played.

Supplementary References

[1] J. W. Crandall. Towards minimizing disappointment in repeated games. *J. Artif. Intell. Res.*, 49:111–142, 2014.

[2] J. W. Crandall. Robust learning in repeated stochastic games using meta-gaming. In *Proceedings of the 24th International Joint Conference on Artificial Intelligence*, 2015.

[3] M. Bowling, N. Burch, M. Johanson, and O. Tammelin. Heads-up limit holdem poker is solved. *Science*, 347(6218):145–149, 2015.

[4] D. Sally. Conversation and cooperation in social dilemmas a meta-analysis of experiments from 1958 to 1992. *Ration. Soc.*, 7(1):58–92, 1995.

[5] D. Balliet. Communication and cooperation in social dilemmas: A meta-analytic review. *Ration. Soc.*, 54(1):39–57, 2009.

[6] R. Axelrod. *The Evolution of Cooperation.* Basic Books, New York, 1984.

[7] D. de Farias and N. Megiddo. How to combine expert (or novice) advice when actions impact the environment. In *Adv. Neur. In.*, 2003.

[8] D. Fudenberg and D. K. Levine. *The Theory of Learning in Games.* The MIT Press, 1998.

[9] J. Hu and M. P. Wellman. Multiagent reinforcement learning: Theoretical framework and an algorithm. In *Proceedings of the 15th International Conference on Machine Learning*, pages 242–250, 1998.

[10] M. L. Littman. Friend-or-foe: Q-learning in general-sum games. In *Proceedings of the 18th International Conference on Machine Learning*, pages 322–328, 2001.

[11] M. Bowling and M. Veloso. Multiagent learning using a variable learning rate. *Artif. Intell.*, 136(2):215–250, 2002.

[12] R. Powers and Y. Shoham. Learning against opponents with bounded memory. In *Proceedings of the 19th International Joint Conference on Artificial Intelligence*, pages 817–822, 2005.

[13] J. W. Crandall and M. A. Goodrich. Learning to compete, coordinate, and cooperate in repeated games using reinforcement learning. *Mach. Learn.*, 82(3):281–314, 2011.

[14] D. P. Foster and R. Vohra. Regret in the on-line decision problem. *Game. Econ. Behav.*, 29:7–35, 1999.

[15] M. Bowling. Convergence and no-regret in multiagent learning. In *Adv. Neur. In.*, pages 209–216, 2004.

[16] A. Greenwald and A. Jafari. A general class of no-regret learning algorithms and game-theoretic equilibria. In *Proceedings of the 16th Annual Conference on Computational Learning Theory*, pages 2–12, 2003.

[17] D. de Farias and N. Megiddo. Exploration–exploitation tradeoffs for expert algorithms in reactive environments. In *Adv. Neur. In.*, pages 409–416, 2004.

[18] R. Arora, O. Dekel, and A. Tewari. Online bandit learning against an adaptive adversary: from regret to policy regret. In *Proceedings of the 29th International Conference on Machine Learning*, pages 1503–1510, 2012.

[19] H. Gintis. *Game Theory Evolving: A Problem-Centered Introduction to Modeling Strategic Behavior*. Princeton University Press, 2000.

[20] V. Conitzer and T. Sandholm. AWESOME: A general multiagent learning algorithm that converges in self-play and learns a best response against stationary opponents. In *Proceedings of the 20th International Conference on Machine Learning*, pages 83–90, 2003.

[21] J. F. Nash. The bargaining problem. *Econometrica*, 28:155–162, 1950.

[22] D. G. Rand, A. Dreber, T. Ellingsen, D. Fudenberg, and M. A. Nowak. Positive interactions promote public cooperation. *Science*, 325(5945):1272–1275, 2009.

[23] D. G. Rand, J. D., and M. A. Nowak. Spontaneous giving and calculated greed. *Nature*, 489(7416):427–430, 2012.

[24] D. G. Rand and M. A. Nowak. Human cooperation. *Trends in Cogn. Sci.*, 17(8):413–425, 2013.

[25] E. Fehr and S. Gächter. Altruistic punishment in humans. *Nature*, 415(6868):137–140, 2002.

[26] C. Camerer. *Behavioral game theory: Experiments in strategic interaction*. Princeton University Press, 2003.

[27] D. G. Rand, A. Peysakhovich, G. T. Kraft-Todd, G. E. Newman, O. Wurzbacher, M. A. Nowak, and J. D. Greene. Social heuristics shape intuitive cooperation. *Nat. Commun.*, 5, 2014.

[28] R. Boyd and P. J. Richerson. Culture and the evolution of human cooperation. *Philos. T. Roy. Soc. B*, 364(1533):3281–3288, 2009.

[29] A. Peysakhovich, M. A Nowak, and D. G. Rand. Humans display a cooperative phenotypethat is domain general and temporally stable. *Nat. Commun.*, 5, 2014.

[30] L. P. Kaelbling, M. L. Littman, and A. W. Moore. Reinforcement learning: A survey. *J. Artif. Intell. Res.*, 4:237–277, 1996.

[31] D. Robinson and D. Goforth. *The Topology of the 2x2 Games: A New Period Table*. Routledge, 2005.

[32] B. Bruns. Navigating the topology of 2x2 games: An introductory note on payoff families, normalization, and natural order. *CoRR*, abs/1010.4727, 2010.

[33] A. Rapoport and M. J. Guyer. *A Taxonomy of 2x2 Games*. Bobbs-Merrill, 1967.

[34] A. Rapoport, M. J. Guyer, and D. G. Gordon. *The 2x2 Game*. The University of Michigan Press, 1976.

[35] S. J. Brams. *A Theory of Moves*. Cambridge University Press, 1994.

[36] M. A. Goodrich, J. W. Crandall, and J. R. Stimpson. Neglect tolerant teaming: Issues and dilemmas. In *Proceedings of the AAAI Spring Symposium on Human Interaction with Autonomous Systems in Complex Environments*, Stanford, CA, March 2003.

[37] G. W. Brown. Iterative solutions of games by fictitious play. In T. C. Koopmans, editor, *Activity Analysis of Production and Allocation*. John Wiley & Sons, New York, 1951.

[38] E. Kalai and E. Lehrer. Rational learning leads to nash equilibrium. *Econometrica*, 61(5):1019–1045, 1993.

[39] B. Skyrms. *The Stag Hunt and the Evolution of Social Structure*. Cambridge Press, 2003.

[40] M. Nowak and K. Sigmund. A strategy of win-stay, lose-shift that outperforms tit-for-tat in the prisoner's dilemma game. *Nature*, 364:56–58, 1993.

[41] W. H. Press and F. J. Dyson. Iterated prisoner's dilemma contains strategies that dominate any evolutionary opponent. *P. Natl. Acad. Sci. USA*, 109(26):10409–10413, 2012.

[42] D. Iliopoulous, A. Hintze, and C. Adami. Critical dynamics in the evolution of stochastic strategies for the iterated prisoner's dilemma. *PLOS Comput. Biol.*, 6(10):1–8, 2010.

[43] M. L. Littman. Markov games as a framework for multi-agent reinforcement learning. In *Proceedings of the 11th International Conference on Machine Learning*, pages 157–163, 1994.

[44] P. Auer, N. Cesa-Bianchi, Y. Freund, and R. E. Schapire. Gambling in a rigged casino: the adversarial multi-armed bandit problem. In *Proceedings of the 36th Symposium on the Foundations of Computer Science*, pages 322–331, 1995.

[45] T. W. Sandholm and R. H. Crites. Multiagent reinforcement learning in the iterated prisoner's dilemma. *Biosystems*, 37:147–166, 1996.

[46] C. Claus and C. Boutilier. The dynamics of reinforcement learning in cooperative multiagent systems. In *Proceedings of the 15th National Conference on Artificial Intelligence*, pages 746–752, 1998.

[47] R. Karandikar, D. Mookherjee, D. R., and F. Vega-Redondo. Evolving aspirations and cooperation. *J. Econ. Theory*, 80:292–331, 1998.

[48] J. Hu and M. P. Wellman. Experimental results on Q-learning for general-sum stochastic games. In *Proceedings of the 17th International Conference on Machine Learning*, pages 407–414, 2000.

[49] M. Bowling. Convergence problems of general-sum multiagent reinforcement learning. In *Proceedings of the 17th International Conference on Machine Learning*, pages 89–94, 2000.

[50] A. Greenwald and K. Hall. Correlated Q-learning. In *Proceedings of the 20th International Conference on Machine Learning*, pages 242–249, 2003.

[51] X. Wang and T. Sandholm. Reinforcement learning to play an optimal Nash equilibrium in team Markov games. In *Adv. Neur. In.*, pages 1571–1578, 2003.

[52] M. L. Littman and P. Stone. Leading best-response strategies in repeated games. In *Proceedings of the IJCAI Workshop on Economic Agents, Models, and Mechanisms*, Seattle, WA, 2001.

[53] M. L. Littman and P. Stone. A polynomial-time Nash equilibrium algorithm for repeated games. *Decis. Support Syst.*, 39:55–66, 2005.

[54] J. R. Stimpson, M. A. Goodrich, and L. C. Walters. Satisficing and learning cooperation in the prisoner's dilemma. In *Proceedings of the 17th National Conference on Artificial Intelligence*, pages 535–544, 2001.

[55] G. Tesauro. Extending Q-learning to general adaptive multi-agent systems. In *Adv. Neur. In.*, 2004.

[56] M. Zinkevich. Online convex programming and generalized infinitesimal gradient ascent. In *Proceedings of the 20th International Conference on Machine Learning*, pages 228–236, 2003.

[57] Y. Chang and L. P. Kaelbling. Hedge learning: Regret-minimization with learning experts. In *Proceedings of the 22nd International Conference on Machine Learning*, pages 121–128, 2005.

[58] E. M. de Cote, A. Lazaric, and M. Restelli. Learning to cooperate in multi-agent social dilemmas. In *Proceedings of the 5th International Conference on Autonomous Agents and Multiagent Systems*, pages 783–785, 2006.

[59] E. M. De Cote and M. L. Littman. A polynomial-time Nash equilibrium algorithm for repeated stochastic games. In *Proceedings of the 24th Conference on Uncertainty in Artificial Intelligence*, pages 419–426, 2008.

[60] S. Abdallah and V. Lesser. A multi-agent learning algorithm with non-linear dynamics. *J. Artif. Intell. Res.*, 33:521–549, 2008.

[61] G. Chasparis, J. Shamma, and A. Arapostathis. Aspiration learning in coordination games. In *Proceedings of the 49th IEEE Conference on Decision and Control*, pages 5756–5761, 2010.

[62] B. Bouzy and M. Metivier. Multi-agent learning experiments in repeated matrix games. In *Proceedings of the 27th International Conference on Machine Learning*, pages 119–126, 2010.

[63] M. Knobbout and G. A.W. Vreeswijk. Sequential targeted optimality as a new criterion for teaching and following in repeated games. In *Proceedings of the 10th International Conference on Autonomous Agents and Multiagent Systems*, pages 517–524, 2011.

[64] S. Ganzfried and T. Sandholm. Game theory-based opponent modeling in large imperfect-information games. In *Proceedings of the 10th International Conference on Autonomous Agents and Multiagent Systems*, pages 533–540, 2011.

[65] N. Cesa-Bianchi, O. Dekel, and O. Shamir. Online learning with switching costs and other adaptive adversaries. In *Adv. Neur. In.*, pages 1160–1168, 2013.

[66] M. Elidrisi, N. Johnson, M. Gini, and J. W. Crandall. Fast adaptive learning in repeated stochastic games by game abstraction. In *Proceedings of the 13th International Conference on Autonomous Agents and Multiagent Systems*, 2014.

[67] S. V. Albrecht and S. Ramamoorthy. On convergence and optimality of best-response learning with policy types in multiagent systems. In *Proceedings of the 30th Conference on Uncertainty in Artificial Intelligence*, Quebec City, Canada, July 2014.

[68] P. D. Taylor and L. Jonker. Evolutionarily stable strategies and game dynamics. *Math. Biosci.*, 40:145–156, 1978.

[69] A. Rapoport, D. A. Seale, and A. M. Colman. Is tit-for-tat the answer? on the conclusions drawn from axelrod's tournaments. *PLoS ONE*, 10(7): e0134128, 2015.

[70] Richard S. Sutton and Andrew G. Barto. *Reinforcement Learning: An Introduction*. MIT Press, 1998.

[71] C. J. C. H. Watkins and P. Dayan. Q-learning. *Mach. Learn.*, 8:279–292, 1992.

[72] D. Banerjee and S. Sen. Reaching pareto-optimality in prisoner's dilemma using conditional joint action learning. *Auton. Agent. Multi-Ag.*, 15:91–108, 2007.

[73] Y. Freund and R. E. Schapire. A decision-theoretic generalization of on-line learning and an application to boosting. *J. Comput. Syst. Sci.*, 55:119–139, 1997.

[74] Robert Axelrod. *The Evolution of Cooperation*. Basic Books, 1984.

[75] H. A. Simon. Rational choice and the structure of the environment. *Psychol. Rev.*, 63(2):129–138, 1956.

[76] S. Arora, E. Hazan, and S. Kale. The multiplicative weights update method: A meta-algorithm and applications. *Theory of Computing*, 8:121–164, 2012.

[77] M. A. Nowak, K. M. Page, and K. Sigmund. Fairness versus reason in the ultimatum game. *Science*, 289 (5485):1773–1775, 2000.

[78] M. Riedmiller and H. Braun. A direct adaptive method for faster backpropagation learning: The RPROP algorithm. In *Proceedings of the IEEE International Conference on Neural Networks*, 1993.

[79] V. Mnih, K. Kavukcuoglu, D. Silver, A. A. Rusu, J. Veness, M. G. Bellemare, A. Graves, M. Riedmiller, A. K. Fidjeland, G. Ostrovski, et al. Human-level control through deep reinforcement learning. *Nature*, 518(7540):529–533, 2015.

[80] J J. O'Neill, B. Pleydell-Bouverie, D. Dupret, and J. Csicsvari. Play it again: reactivation of waking experience and memory. *Trends Neurosci.*, 33:220–229, 2010.

[81] B. Bouzy, M. Metivier, and D. Pellier. Hedging algorithms and repeated matrix games. In *Proceedings of the ECML Workshop on Machine Learning and Data Mining in and Around Games*, Athens, Greece, 2011.

[82] J. R. Stimpson and M. A. Goodrich. Learning to cooperate in a social dilemma: A satisficing approach to bargaining. In *Proceedings of the 20th International Conference on Machine Learning*, pages 728–735, 2003.

[83] Y. Gal, S. Kraus, M. Gelfan, H. Khashan, and E. Salmon. Negotiating with people across cultures using an adaptive agent. *ACM T. Intel. Syst. Tec.*, 3(1), 2011.

[84] M. Smith and G. R Price. The logic of animal conflict. *Nature*, 246:15–18, 1973.

[85] L. Shapley. Some topics in two-person games. *Advances in Game Theory*, 1964.

[86] M. Oudah, V. Babushkin, T. Chenlinangjia, and J. W. Crandall. Learning to interact with a human partner. In *Proceedings of the 10th International Conference on Human-Robot Interaction*, 2015.

[87] M. Zinkevich, M. Johanson, M. H. Bowling, and C. Piccione. Regret minimization in games with incomplete information. In *Adv. Neur. In.*, 2007.

[88] M. Johanson, N. Bard, M. Lanctot, R. Gibson, and M. Bowling. Efficient Nash equilibrium approximation through Monte Carlo counterfactual regret minimization. In *Proceedings of the 11th International Conference on Autonomous Agents and Multiagent Systems*, pages 837–846, 2012.

[89] V. P. Crawford and J. Sobel. Strategic information transmission. *Econometrica*, 50 (6):1431–1451, 1982.

[90] R. J. Aumann and S. Hart. Long cheap talk. *Econometrica*, 71 (6):1619–1660, 2003.

[91] J. Farrell and M. Rabin. Cheap talk. *J. Econ. Perspect.*, 10 (3):103–118, 1996.

[92] J. Y. Kim. Cheap talk and reputation in repeated pretrial negotiation. *Rand J. Econ.*, 27 (4):787–802, 1996.

[93] A. L. Thomaz and C. Breazeal. Teachable robots: Understanding human teaching behavior to build more effective robot learners. *Artif. Intell.*, 172:716–737, 2008.

[94] A. Turing. Computing machinery and intelligence. *Mind*, 59:433–460, 1950.
