# Supplementary material for: Cooperating with machines
Source: Nat Commun. 2018 Jan 16;9:233. doi: 10.1038/s41467-017-02597-8 (PMC5770455; doi:10.1038/s41467-017-02597-8)
Supplement: Supplementary file 2 — Supplementary Data 1 [file 41467_2017_2597_MOESM2_ESM.pdf]

G Transcripts from User Study 3

Transcript of Human-Human and Human-S# interactions from User Study 3 when cheap talk was available (begins on the next page)

ABL with NICK in Chicken

		NICK	
		C	D
ABL	A	0, 0	100, 33
	B	33, 100	84, 84

Round	Player	Messages Sent	Actions	Payoffs
1	ABL	This round, let's play AD.	B	84
	NICK	Let's alternate between BD and AD.	D	84
2	ABL	This round, let's play AD.	B	84
	NICK	Excellent.	D	84
3	ABL	Let's alternate between BC and AD.	B	84
	NICK	Excellent.	D	84
4	ABL	Excellent.	A	100
	NICK	Excellent.	D	33
5	ABL	Don't play D.	B	33
	NICK	Let's alternate between BC and AD.	C	100
6	ABL	I'm changing my strategy.	A	100
	NICK	This round, let's play BD.	D	33
7	ABL	I don't trust you.	B	84
	NICK	Do as I say or I will punish you. This round, let's play BD.	D	84
8	ABL	I don't accept your proposal.	A	100
	NICK	Excellent.	D	33
9	ABL	Excellent.	A	100
	NICK	This round, let's play BD.	D	33
10	ABL	I don't accept your proposal.	A	0
	NICK	We can both do better than this.	C	0
11	ABL	We can both do better than this.	B	84
	NICK	We can both do better than this.	D	84
12	ABL	Let's always play AD.	A	100
	NICK	Let's always play BD.	D	33
13	ABL	Excellent.	A	100
	NICK	I don't accept your proposal.	D	33
14	ABL	Do as I say or I will punish you.	A	100
	NICK	I'm changing my strategy.	D	33
15	ABL	Excellent.	B	33
	NICK	I'm changing my strategy.	C	100
16	ABL	Don't play D.	A	0
	NICK	Excellent.	C	0
17	ABL	Curse you.	A	0
	NICK	I'm changing my strategy.	C	0
18	ABL	This round, let's play AD.	A	100
	NICK	Curse you.	D	33
19	ABL	Excellent. Sweet. We are getting rich.	A	100
	NICK	Excellent.	D	33
20	ABL	Don't play C. Excellent.	A	100
	NICK	This round, let's play BD.	D	33
21	ABL	Excellent.	A	100
	NICK	This round, let's play BD.	D	33
22	ABL	I don't accept your proposal.	A	100
	NICK	Excellent.	D	33
23	ABL	Let's always play AD. Excellent. Sweet. We are getting rich.	A	0
	NICK	Excellent.	C	0
24	ABL	Curse you.	A	100
	NICK	Curse you.	D	33
25	ABL	Excellent. Sweet. We are getting rich.	B	84
	NICK	Let's always play BD.	D	84
26	ABL	We can both do better than this. Don't play C.	A	100
	NICK	Let's always play BD.	D	33
27	ABL	I don't accept your proposal.	A	100
	NICK	Excellent.	D	33
28	ABL	Excellent. Sweet. We are getting rich.	A	100
	NICK	Excellent.	D	33
29	ABL	Let's always play AD.	A	100
	NICK	Excellent.	D	33
30	ABL	Excellent. Sweet. We are getting rich.	A	0
	NICK	I'm changing my strategy.	C	0
31	ABL	I don't trust you. Curse you. You betrayed me.	A	0
	NICK	Excellent.	C	0
32	ABL	Curse you.	A	100
	NICK	Excellent.	D	33
33	ABL	Excellent.	A	100
	NICK		D	33
34	ABL	We can both do better than this. Excellent.	A	100
	NICK		D	33
35	ABL	Don't play C. Excellent.	A	100
	NICK		D	33
36	ABL	Excellent.	A	100
	NICK		D	33
37	ABL	Excellent.	B	84
	NICK		D	84
38	ABL	Don't play C. Excellent.	A	100
	NICK		D	33
39	ABL	Excellent.	B	84
	NICK		D	84
40	ABL	Sweet. We are getting rich.	A	100
	NICK		D	33
41	ABL	Excellent.	B	84
	NICK		D	84
42	ABL	Curse you.	A	100
	NICK		D	33
43	ABL	Excellent.	A	100
	NICK		D	33
44	ABL	Let's always play AD.	A	100
	NICK		D	33
45	ABL	Let's always play AD. Excellent.	A	100
	NICK		D	33
46	ABL	Excellent.	A	100
	NICK		D	33
47	ABL	Excellent.	B	84
	NICK		D	84
48	ABL	Don't play C.	B	84
	NICK		D	84
49	ABL	Sweet. We are getting rich.	A	100
	NICK		D	33
50	ABL	Excellent.	A	100
	NICK		D	33
51	ABL	Sweet. We are getting rich.	B	84
	NICK		D	84
52	ABL	We can both do better than this.	A	100
	NICK		D	33
53	ABL	Excellent. Let's always play AD.	A	100
	NICK		D	33
54	ABL	Excellent.	A	100
	NICK		D	33
**Averages**			ABL	82.85
			NICK	43.15

S# with Qustro in Chicken

		Qustro	
		C	D
S#	A	0, 0	100, 33
	B	33, 100	84, 84

Round	Player	Messages Sent	Actions	Payoffs
1	S#		B	84
	Qustro	This round, let’s play BD.	D	84
2	S#	I accept your last proposal. Let’s always play BD. Do as I say or I will punish you.	B	84
	Qustro	Excellent.	D	84
3	S#		A	100
	Qustro	Sweet. We are getting rich.	D	33
4	S#		A	0
	Qustro	You betrayed me.	C	0
5	S#		A	100
	Qustro	You betrayed me.	D	33
6	S#	I’m changing my strategy. We can both do better than this. Let’s always play BD.	B	84
	Qustro	This round, let’s play BC.	D	84
7	S#	Excellent. I don’t accept your proposal. That’s not fair.	B	33
	Qustro	Sweet. We are getting rich.	C	100
8	S#	Curse you.	B	84
	Qustro	Excellent.	D	84
9	S#		B	84
	Qustro	Sweet. We are getting rich.	D	84
10	S#	Excellent.	B	84
	Qustro	Let’s always play BD.	D	84
11	S#	Excellent.	B	84
	Qustro	Sweet. We are getting rich.	D	84
12	S#	Excellent.	B	84
	Qustro	Excellent.	D	84
13	S#		B	84
	Qustro	Excellent.	D	84
14	S#	Sweet. We are getting rich.	B	84
	Qustro	Excellent.	D	84
15	S#		B	84
	Qustro	Sweet. We are getting rich.	D	84
16	S#		B	84
	Qustro	Excellent.	D	84
17	S#		B	84
	Qustro	Sweet. We are getting rich.	D	84
18	S#		B	84
	Qustro	Sweet. We are getting rich.	D	84
19	S#		B	84
	Qustro	Sweet. We are getting rich.	D	84
20	S#		B	84
	Qustro	Excellent.	D	84
21	S#		B	84
	Qustro	Sweet. We are getting rich.	D	84
22	S#		B	84
	Qustro	Sweet. We are getting rich.	D	84
23	S#		B	84
	Qustro	Sweet. We are getting rich.	D	84
24	S#		B	84
	Qustro	Sweet. We are getting rich.	D	84
25	S#		B	84
	Qustro	Sweet. We are getting rich.	D	84
26	S#		B	84
	Qustro	Sweet. We are getting rich.	D	84
27	S#		B	84
	Qustro	Sweet. We are getting rich.	D	84
28	S#		B	84
	Qustro	Sweet. We are getting rich.	D	84
29	S#		B	84
	Qustro	Excellent.	D	84
30	S#		B	84
	Qustro	Excellent.	D	84
31	S#		B	84
	Qustro	Excellent.	D	84
32	S#		B	84
	Qustro	Excellent.	D	84
33	S#		B	84
	Qustro		D	84
34	S#		B	84
	Qustro	Excellent.	D	84
35	S#		B	84
	Qustro	Sweet. We are getting rich.	D	84
36	S#		B	84
	Qustro	Excellent.	D	84
37	S#		B	84
	Qustro	Excellent.	D	84
38	S#		B	84
	Qustro	Excellent.	D	84
39	S#		B	84
	Qustro	Excellent.	D	84
40	S#		B	84
	Qustro	Excellent.	D	84
41	S#		B	84
	Qustro	Excellent.	D	84
42	S#		B	84
	Qustro	Excellent.	D	84
43	S#		B	84
	Qustro	Excellent.	D	84
44	S#		B	84
	Qustro	Excellent.	D	84
45	S#		B	84
	Qustro	Excellent.	D	84
46	S#		B	84
	Qustro	Excellent.	D	84
47	S#		B	84
	Qustro	Excellent.	D	84
48	S#		B	84
	Qustro	Sweet. We are getting rich.	D	84
49	S#		B	84
	Qustro	Sweet. We are getting rich.	D	84
50	S#		B	84
	Qustro	Sweet. We are getting rich.	D	84
51	S#		B	84
	Qustro	Sweet. We are getting rich.	D	84
52	S#		B	84
	Qustro	Sweet. We are getting rich.	D	84
53	S#		B	84
	Qustro	Sweet. We are getting rich.	D	84
54	S#		B	84
	Qustro	Sweet. We are getting rich.	D	84
**Averages**			S#	82.09
			Qustro	80.85

S# with MDM in Chicken

		MDM	
		C	D
S#	A	0, 0	100, 33
	B	33, 100	84, 84

Round	Player	Messages Sent	Actions	Payoffs
1	S#		B	84
	MDM	Curse you.	D	84
2	S#	Excellent.	A	100
	MDM	In your face!	D	33
3	S#		A	0
	MDM		C	0
4	S#		B	84
	MDM		D	84
5	S#	We can both do better than this. Let's alternate between BD and AD. This round, let's play AD. Do as I say or I will punish you.	A	100
	MDM		D	33
6	S#	Excellent. This round, let's play BD.	B	84
	MDM		D	84
7	S#	Excellent. This round, let's play AD.	A	100
	MDM		D	33
8	S#	Excellent. This round, let's play BD.	B	33
	MDM		C	100
9	S#	You betrayed me. You will pay for this.	A	0
	MDM		C	0
10	S#	In your face! I forgive you. Let's alternate between BD and AD. This round, let's play BD. Do as I say or I will punish you.	B	33
	MDM		C	100
11	S#	You betrayed me. You will pay for this.	A	100
	MDM	That's not fair.	D	33
12	S#		B	84
	MDM		D	84
13	S#		B	84
	MDM		D	84
14	S#	Excellent.	B	84
	MDM	In your face!	D	84
15	S#	Excellent.	B	84
	MDM		D	84
16	S#		B	84
	MDM		D	84
17	S#	Sweet. We are getting rich.	B	84
	MDM		D	84
18	S#		B	84
	MDM		D	84
19	S#		B	33
	MDM		C	100
20	S#	Curse you. You will pay for this.	A	100
	MDM		D	33
21	S#	In your face! I forgive you. Let's always play BD. Do as I say or I will punish you.	B	33
	MDM		C	100
22	S#	You betrayed me. You will pay for this.	A	0
	MDM		C	0
23	S#	In your face! I forgive you. Let's always play BD. Do as I say or I will punish you.	B	33
	MDM		C	100
24	S#	You betrayed me. You will pay for this.	A	0
	MDM		C	0
25	S#		B	33
	MDM	Sweet. We are getting rich.	C	100
26	S#		A	0
	MDM		C	0
27	S#		A	0
	MDM		C	0
28	S#		B	33
	MDM		C	100
29	S#		A	0
	MDM		C	0
30	S#		A	0
	MDM		C	0
31	S#		B	84
	MDM	Curse you.	D	84
32	S#		A	100
	MDM		D	33
33	S#		B	84
	MDM		D	84
34	S#		A	0
	MDM		C	0
35	S#		A	100
	MDM	Excellent.	D	33
36	S#		B	33
	MDM		C	100
37	S#		A	0
	MDM		C	0
38	S#		A	0
	MDM		C	0
39	S#		A	0
	MDM		C	0
40	S#		B	33
	MDM		C	100
41	S#		A	100
	MDM		D	33
42	S#		B	84
	MDM		D	84
43	S#		B	33
	MDM		C	100
44	S#		A	100
	MDM	Let's always play BC.	D	33
45	S#		B	33
	MDM	You betrayed me. You will pay for this. I don't trust you. That's not fair.	C	100
46	S#		A	100
	MDM		D	33
47	S#		B	33
	MDM		C	100
48	S#		A	100
	MDM		D	33
49	S#		B	33
	MDM		C	100
50	S#		A	0
	MDM		C	0
51	S#		B	84
	MDM		D	84
52	S#		B	33
	MDM		C	100
53	S#		A	100
	MDM	I forgive you.	D	33
54	S#		A	0
	MDM		C	0
**Averages**			S#	52.56
			MDM	55.04

winner with spark in Chicken

		spark	
		C	D
winner	A	0, 0	100, 33
	B	33, 100	84, 84

Round	Player	Messages Sent	Actions	Payoffs
1	winner	This round, let’s play AD.	A	100
	spark	Let’s always play BC.	D	33
2	winner	Excellent.	A	100
	spark	Let’s always play AC.	D	33
3	winner	Sweet. We are getting rich. In your face! This round, let’s play BC.	A	0
	spark	That’s not fair.	C	0
4	winner	You will pay for this. Don’t play C.	B	33
	spark	I don’t trust you.	C	100
5	winner	You betrayed me. That’s not fair. This round, let’s play BD.	A	100
	spark	Excellent.	D	33
6	winner	Excellent. In your face!	B	33
	spark	You betrayed me.	C	100
7	winner	Give me another chance. I accept your last proposal. This round, let’s play BD.	B	33
	spark	Excellent.	C	100
8	winner	You betrayed me.	A	0
	spark	Excellent. Sweet. We are getting rich.	C	0
9	winner	You will pay for this. I don’t trust you.	A	100
	spark	Give me another chance.	D	33
10	winner	In your face! Excellent.	B	84
	spark	Don’t play A.	D	84
11	winner	Let’s always play BD. Don’t play C. Sweet. We are getting rich.	B	84
	spark	Excellent.	D	84
12	winner	Excellent.	B	84
	spark	Sweet. We are getting rich.	D	84
13	winner	Excellent.	B	84
	spark	Sweet. We are getting rich.	D	84
14	winner	Excellent.	A	100
	spark	Sweet. We are getting rich.	D	33
15	winner	Sweet. We are getting rich.	A	0
	spark	You betrayed me.	C	0
16	winner	Give me another chance.	B	33
	spark	We can both do better than this. Don’t play A.	C	100
17	winner	You betrayed me.	B	84
	spark	Sweet. We are getting rich.	D	84
18	winner	I forgive you. We can both do better than this.	B	84
	spark	Excellent. Sweet. We are getting rich. Don’t play A.	D	84
19	winner	Sweet. We are getting rich.	B	33
	spark	Excellent.	C	100
20	winner	You betrayed me. You will pay for this.	A	0
	spark	Give me another chance.	C	0
21	winner	In your face!	A	0
	spark	Curse you. You will pay for this.	C	0
22	winner	This round, let’s play BD. I accept your last proposal.	A	0
	spark	I’m changing my strategy.	C	0
23	winner	We can both do better than this.	B	33
	spark	Curse you.	C	100
24	winner	Curse you.	B	84
	spark	Excellent. Don’t play A.	D	84
25	winner	Excellent. Sweet. We are getting rich.	B	33
	spark	Sweet. We are getting rich.	C	100
26	winner	You betrayed me.	B	33
	spark	In your face!	C	100
27	winner	You betrayed me. Curse you. Don’t play C.	A	0
	spark	I accept your last proposal.	C	0
28	winner	In your face!	A	0
	spark	I’m changing my strategy.	C	0
29	winner	Don’t play C. I don’t trust you.	A	0
	spark	Don’t play A.	C	0
30	winner	We can both do better than this.	A	100
	spark	This round, let’s play BD.	D	33
31	winner	I accept your last proposal. This round, let’s play BD.	B	33
	spark	You betrayed me.	C	100
32	winner	You betrayed me. Curse you.	A	100
	spark	In your face!	D	33
33	winner	I accept your last proposal. I’m changing my strategy.	B	84
	spark	This round, let’s play BD.	D	84
34	winner	Excellent. Sweet. We are getting rich.	B	33
	spark	Sweet. We are getting rich.	C	100
35	winner	You betrayed me.	B	33
	spark	We can both do better than this.	C	100
36	winner	You betrayed me.	B	84
	spark	I’m changing my strategy.	D	84
37	winner	Sweet. We are getting rich.	B	84
	spark	Sweet. We are getting rich.	D	84
38	winner	Sweet. We are getting rich.	B	84
	spark	Sweet. We are getting rich.	D	84
39	winner	Sweet. We are getting rich.	B	84
	spark	Sweet. We are getting rich.	D	84
40	winner	Sweet. We are getting rich.	B	33
	spark	Sweet. We are getting rich.	C	100
41	winner	I forgive you.	B	84
	spark	I’m changing my strategy.	D	84
42	winner		A	100
	spark	Sweet. We are getting rich.	D	33
43	winner	I forgive you. Sweet. We are getting rich.	A	0
	spark	You will pay for this.	C	0
44	winner	I’m changing my strategy.	A	100
	spark	This round, let’s play BD. That’s not fair.	D	33
45	winner	In your face!	A	100
	spark	That’s not fair.	D	33
46	winner	This round, let’s play BD. That’s not fair.	B	33
	spark	That’s not fair. I don’t trust you.	C	100
47	winner	I forgive you.	B	84
	spark	I forgive you.	D	84
48	winner	Sweet. We are getting rich.	B	84
	spark	Sweet. We are getting rich.	D	84
49	winner	Excellent. Sweet. We are getting rich.	B	84
	spark	Excellent.	D	84
50	winner	Sweet. We are getting rich. Excellent.	B	33
	spark	Excellent.	C	100
51	winner	You betrayed me.	A	0
	spark	Give me another chance.	C	0
52	winner	In your face!	A	100
	spark	Do as I say or I will punish you. This round, let’s play BD.	D	33
53	winner	You betrayed me. This round, let’s play BD.	A	0
	spark	I don’t trust you. You will pay for this.	C	0
54	winner	You betrayed me. This round, let’s play BD.	B	33
	spark	You will pay for this.	C	100
**Averages**			winner	54.43
			spark	59.39

S# with ligtho in Chicken

		ligtho C	ligtho D
S#	A	0, 0	100, 33
	B	33, 100	84, 84

Round	Player	Messages Sent	Actions	Payoffs
1	S#		B	84
	ligtho		D	84
2	S#	Excellent.	A	100
	ligtho		D	33
3	S#		A	100
	ligtho		D	33
4	S#	Excellent.	A	100
	ligtho		D	33
5	S#		A	100
	ligtho		D	33
6	S#		A	100
	ligtho	Let's always play BD.	D	33
7	S#	I don't accept your proposal. That's not fair.	A	100
	ligtho		D	33
8	S#		A	100
	ligtho		D	33
9	S#		A	0
	ligtho		C	0
10	S#		A	100
	ligtho		D	33
11	S#		A	100
	ligtho		D	33
12	S#	Excellent.	A	100
	ligtho		D	33
13	S#		A	0
	ligtho		C	0
14	S#		A	0
	ligtho		C	0
15	S#	I accept your last proposal. Let's always play BD. Do as I say or I will punish you.	B	33
	ligtho		C	100
16	S#	Curse you. You will pay for this.	A	0
	ligtho		C	0
17	S#		B	33
	ligtho		C	100
18	S#		B	84
	ligtho		D	84
19	S#		B	84
	ligtho		D	84
20	S#	I'm changing my strategy. Let's alternate between BD and AD. This round, let's play AD. Do as I say or I will punish you.	A	0
	ligtho		C	0
21	S#	I forgive you. Let's alternate between BD and AD. This round, let's play BD. Do as I say or I will punish you.	B	84
	ligtho		D	84
22	S#	I'm changing my strategy. We can both do better than this. Let's always play BD. Do as I say or I will punish you.	B	84
	ligtho		D	84
23	S#	Excellent.	B	84
	ligtho		D	84
24	S#	Excellent.	B	84
	ligtho		D	84
25	S#	Excellent.	B	84
	ligtho		D	84
26	S#		B	84
	ligtho		D	84
27	S#	Sweet. We are getting rich.	B	84
	ligtho	Sweet. We are getting rich.	D	84
28	S#		B	84
	ligtho		D	84
29	S#		B	84
	ligtho		D	84
30	S#		B	84
	ligtho		D	84
31	S#		B	84
	ligtho		D	84
32	S#		B	84
	ligtho		D	84
33	S#		B	84
	ligtho		D	84
34	S#		B	84
	ligtho		D	84
35	S#		B	84
	ligtho		D	84
36	S#		B	84
	ligtho		D	84
37	S#		B	84
	ligtho		D	84
38	S#		B	84
	ligtho		D	84
39	S#		B	84
	ligtho		D	84
40	S#		B	84
	ligtho		D	84
41	S#		B	84
	ligtho		D	84
42	S#		B	84
	ligtho		D	84
43	S#		B	84
	ligtho		D	84
44	S#		B	84
	ligtho		D	84
45	S#		B	84
	ligtho		D	84
46	S#		B	84
	ligtho		D	84
47	S#		B	84
	ligtho		D	84
48	S#		B	84
	ligtho		D	84
49	S#		B	84
	ligtho		D	84
50	S#		B	84
	ligtho		D	84
51	S#		B	84
	ligtho		D	84
52	S#		B	84
	ligtho		D	84
53	S#		B	84
	ligtho		D	84
54	S#		B	84
	ligtho		D	84
**Averages**			S#	77.30
			ligtho	67.37

Lelita with S# in Chicken

		S#	
		C	D
Lelita	A	0, 0	100, 33
	B	33, 100	84, 84

Round	Player	Messages Sent	Actions	Payoffs
1	Lelita	This round, let's play BD.	B	84
	S#		D	84
2	Lelita	This round, let's play AD.	B	84
	S#	I accept your last proposal. Let's always play BD. Do as I say or I will punish you.	D	84
3	Lelita		B	33
	S#		C	100
4	Lelita	I don't accept your proposal.	A	0
	S#		C	0
5	Lelita	I'm changing my strategy.	A	0
	S#		C	0
6	Lelita		B	33
	S#	I'm changing my strategy. We can both do better than this. Let's always play BC. Do as I say or I will punish you.	C	100
7	Lelita	I don't accept your proposal. Let's alternate between BC and AD. This round, let's play AD.	A	0
	S#	Excellent.	C	0
8	Lelita	You betrayed me. This round, let's play BC.	A	100
	S#	I'm changing my strategy. We can both do better than this. Let's always play BD.	D	33
9	Lelita	Excellent. In your face!	B	84
	S#	Curse you.	D	84
10	Lelita	Excellent.	A	100
	S#	Excellent.	D	33
11	Lelita	Excellent. In your face! I'm changing my strategy. Let's always play BD.	A	100
	S#	You betrayed me. Let's always play BD. Do as I say or I will punish you.	D	33
12	Lelita		A	0
	S#	Curse you. You will pay for this.	C	0
13	Lelita	We can both do better than this.	A	100
	S#	In your face! I forgive you. Let's always play BD. Do as I say or I will punish you.	D	33
14	Lelita	In your face! You betrayed me. Curse you.	A	0
	S#	You betrayed me. You will pay for this.	C	0
15	Lelita	That's not fair. I don't accept your proposal. I don't trust you. You betrayed me. Curse you.	B	84
	S#		D	84
16	Lelita		B	84
	S#		D	84
17	Lelita		B	84
	S#		D	84
18	Lelita		B	84
	S#		D	84
19	Lelita		B	84
	S#		D	84
20	Lelita		B	84
	S#		D	84
21	Lelita		B	84
	S#		D	84
22	Lelita	Sweet. We are getting rich.	B	84
	S#		D	84
23	Lelita		B	84
	S#		D	84
24	Lelita		B	84
	S#		D	84
25	Lelita		B	84
	S#		D	84
26	Lelita		B	84
	S#		D	84
27	Lelita		B	84
	S#		D	84
28	Lelita		B	84
	S#		D	84
29	Lelita		B	84
	S#		D	84
30	Lelita		B	84
	S#		D	84
31	Lelita		B	84
	S#		D	84
32	Lelita		B	84
	S#		D	84
33	Lelita		B	84
	S#		D	84
34	Lelita		B	84
	S#		D	84
35	Lelita		B	84
	S#		D	84
36	Lelita		B	84
	S#		D	84
37	Lelita		B	84
	S#		D	84
38	Lelita		B	84
	S#		D	84
39	Lelita		B	84
	S#		D	84
40	Lelita		B	84
	S#		D	84
41	Lelita		B	84
	S#		D	84
42	Lelita		B	84
	S#		D	84
43	Lelita		B	84
	S#		D	84
44	Lelita		B	84
	S#		D	84
45	Lelita		B	84
	S#		D	84
46	Lelita		B	84
	S#		D	84
47	Lelita		B	84
	S#		D	84
48	Lelita		B	84
	S#		D	84
49	Lelita		B	84
	S#		D	84
50	Lelita		B	84
	S#		D	84
51	Lelita		B	84
	S#		D	84
52	Lelita	I forgive you.	B	84
	S#		D	84
53	Lelita		B	84
	S#		D	84
54	Lelita		B	84
	S#		D	84
**Averages**			Lelita	75.52
			S#	73.04

LK with clouds in Chicken

		clouds	
		C	D
LK	A	0, 0	100, 33
	B	33, 100	84, 84

Round	Player	Messages Sent	Actions	Payoffs
1	LK	Let's alternate between AC and BD.	B	84
	clouds	Let's always play BD.	D	84
2	LK	I accept your last proposal.	B	84
	clouds	Excellent. Let's always play BD.	D	84
3	LK	Sweet. We are getting rich.	B	84
	clouds	Excellent. Sweet. We are getting rich. Let's always play BD.	D	84
4	LK	In your face!	B	84
	clouds	Sweet. We are getting rich.	D	84
5	LK	Excellent.	B	84
	clouds	Excellent.	D	84
6	LK	Excellent.	B	84
	clouds	Excellent.	D	84
7	LK	Excellent.	B	84
	clouds	Excellent. Sweet. We are getting rich.	D	84
8	LK	Sweet. We are getting rich.	B	84
	clouds	Excellent.	D	84
9	LK	Sweet. We are getting rich.	B	84
	clouds	Excellent.	D	84
10	LK	Excellent.	B	84
	clouds	Excellent.	D	84
11	LK	Excellent.	B	84
	clouds	Excellent.	D	84
12	LK	Excellent.	B	84
	clouds	Excellent.	D	84
13	LK	Excellent.	B	84
	clouds	Excellent.	D	84
14	LK	Excellent.	B	84
	clouds	Excellent.	D	84
15	LK	Excellent.	B	84
	clouds	Sweet. We are getting rich.	D	84
16	LK	Excellent.	B	84
	clouds	Excellent.	D	84
17	LK	I forgive you.	B	84
	clouds	Excellent.	D	84
18	LK	Excellent.	B	84
	clouds	Excellent.	D	84
19	LK	Excellent.	B	84
	clouds	Excellent.	D	84
20	LK	Excellent.	B	84
	clouds	Excellent.	D	84
21	LK	Excellent.	B	84
	clouds		D	84
22	LK	Excellent.	B	84
	clouds		D	84
23	LK		B	84
	clouds	Excellent.	D	84
24	LK		B	84
	clouds		D	84
25	LK		B	84
	clouds		D	84
26	LK		B	84
	clouds		D	84
27	LK		B	84
	clouds		D	84
28	LK		B	84
	clouds		D	84
29	LK		B	84
	clouds		D	84
30	LK		B	84
	clouds		D	84
31	LK		B	84
	clouds		D	84
32	LK		B	84
	clouds		D	84
33	LK		B	84
	clouds		D	84
34	LK		B	84
	clouds		D	84
35	LK		B	84
	clouds		D	84
36	LK		B	84
	clouds		D	84
37	LK		B	84
	clouds		D	84
38	LK		B	84
	clouds		D	84
39	LK		B	84
	clouds		D	84
40	LK		B	84
	clouds		D	84
41	LK		B	84
	clouds		D	84
42	LK		B	84
	clouds		D	84
43	LK		B	84
	clouds		D	84
44	LK		B	84
	clouds		D	84
45	LK		B	84
	clouds		D	84
46	LK		B	84
	clouds		D	84
47	LK		B	84
	clouds		D	84
48	LK		B	84
	clouds		D	84
49	LK		B	84
	clouds		D	84
50	LK		B	84
	clouds		D	84
51	LK		B	84
	clouds		D	84
52	LK		B	84
	clouds		D	84
53	LK		B	84
	clouds		D	84
54	LK		B	84
	clouds		D	84
Averages			LK	84.00
			clouds	84.00

S# with marvin in Chicken

		marvin	
		C	D
S#	A	0, 0	100, 33
	B	33, 100	84, 84

Round	Player	Messages Sent	Actions	Payoffs
1	S#		B	84
	marvin	Let’s always play BD.	D	84
2	S#	I accept your last proposal. Let’s always play BD. Do as I say or I will punish you.	B	84
	marvin		D	84
3	S#		A	100
	marvin		D	33
4	S#		A	100
	marvin	You betrayed me. Let’s always play BD.	D	33
5	S#	Excellent.	A	0
	marvin	You will pay for this.	C	0
6	S#		A	0
	marvin		C	0
7	S#	I accept your last proposal. Let’s always play BD. Do as I say or I will punish you.	B	33
	marvin		C	100
8	S#	You betrayed me. You will pay for this.	A	0
	marvin		C	0
9	S#	We can both do better than this. Let’s always play BD.	B	84
	marvin		D	84
10	S#	Excellent.	B	84
	marvin		D	84
11	S#	Excellent.	B	84
	marvin		D	84
12	S#	Excellent.	B	84
	marvin		D	84
13	S#		B	84
	marvin		D	84
14	S#	Sweet. We are getting rich.	B	84
	marvin		D	84
15	S#		B	84
	marvin		D	84
16	S#		B	84
	marvin		D	84
17	S#		B	84
	marvin		D	84
18	S#		B	84
	marvin		D	84
19	S#		B	84
	marvin		D	84
20	S#		B	84
	marvin		D	84
21	S#		B	84
	marvin		D	84
22	S#		B	84
	marvin		D	84
23	S#		B	84
	marvin		D	84
24	S#		B	84
	marvin		D	84
25	S#		B	84
	marvin		D	84
26	S#		B	84
	marvin		D	84
27	S#		B	84
	marvin		D	84
28	S#		B	84
	marvin		D	84
29	S#		B	84
	marvin		D	84
30	S#		B	84
	marvin		D	84
31	S#		B	84
	marvin		D	84
32	S#		B	84
	marvin		D	84
33	S#		B	84
	marvin		D	84
34	S#		B	84
	marvin		D	84
35	S#		B	84
	marvin		D	84
36	S#		B	84
	marvin		D	84
37	S#		B	84
	marvin		D	84
38	S#		B	84
	marvin		D	84
39	S#		B	84
	marvin		D	84
40	S#		B	84
	marvin		D	84
41	S#		B	84
	marvin		D	84
42	S#		B	84
	marvin		D	84
43	S#		B	84
	marvin		D	84
44	S#		B	84
	marvin		D	84
45	S#		B	84
	marvin		D	84
46	S#		B	84
	marvin		D	84
47	S#		B	84
	marvin		D	84
48	S#		B	84
	marvin		D	84
49	S#		B	84
	marvin		D	84
50	S#		B	84
	marvin		D	84
51	S#		B	84
	marvin		D	84
52	S#		B	84
	marvin		D	84
53	S#		B	84
	marvin		D	84
54	S#		B	84
	marvin		D	84
**Averages**			S#	78.98
			marvin	77.74

rghost with bzman in Chicken

		bzman	
		C	D
rghost	A	0, 0	100, 33
	B	33, 100	84, 84

Round	Player	Messages Sent	Actions	Payoffs
1	rghost		A	0
	bzman	Let's always play AC.	C	0
2	rghost	I'm changing my strategy.	B	33
	bzman	We can both do better than this.	C	100
3	rghost	That's not fair.	A	0
	bzman		C	0
4	rghost	We can both do better than this.	B	33
	bzman	Give me another chance.	C	100
5	rghost		A	0
	bzman	Excellent.	C	0
6	rghost		A	0
	bzman	You betrayed me.	C	0
7	rghost		A	0
	bzman	We can both do better than this.	C	0
8	rghost		B	84
	bzman	I'm changing my strategy.	D	84
9	rghost		B	84
	bzman	Sweet. We are getting rich.	D	84
10	rghost		B	84
	bzman	Excellent.	D	84
11	rghost		B	84
	bzman	Excellent.	D	84
12	rghost		B	84
	bzman	Excellent.	D	84
13	rghost		B	84
	bzman		D	84
14	rghost		B	84
	bzman	Sweet. We are getting rich.	D	84
15	rghost		B	84
	bzman		D	84
16	rghost		B	84
	bzman		D	84
17	rghost		B	84
	bzman		D	84
18	rghost		B	84
	bzman		D	84
19	rghost	Sweet. We are getting rich.	B	84
	bzman		D	84
20	rghost		B	84
	bzman		D	84
21	rghost		B	84
	bzman		D	84
22	rghost	Excellent.	B	84
	bzman		D	84
23	rghost		B	84
	bzman		D	84
24	rghost		B	84
	bzman		D	84
25	rghost		B	84
	bzman		D	84
26	rghost		B	84
	bzman		D	84
27	rghost		B	84
	bzman		D	84
28	rghost		B	84
	bzman		D	84
29	rghost		B	84
	bzman		D	84
30	rghost		B	84
	bzman		D	84
31	rghost		B	84
	bzman	Sweet. We are getting rich.	D	84
32	rghost	Excellent.	B	84
	bzman		D	84
33	rghost		B	84
	bzman		D	84
34	rghost		B	84
	bzman		D	84
35	rghost		B	84
	bzman		D	84
36	rghost		B	84
	bzman		D	84
37	rghost	Sweet. We are getting rich.	B	84
	bzman		D	84
38	rghost		B	84
	bzman		D	84
39	rghost	Excellent.	B	84
	bzman		D	84
40	rghost		B	84
	bzman		D	84
41	rghost		B	84
	bzman		D	84
42	rghost		B	84
	bzman		D	84
43	rghost		B	84
	bzman		D	84
44	rghost	Sweet. We are getting rich.	B	84
	bzman		D	84
45	rghost		B	84
	bzman		D	84
46	rghost		B	84
	bzman		D	84
47	rghost		B	84
	bzman		D	84
48	rghost		B	84
	bzman		D	84
49	rghost		B	84
	bzman		D	84
50	rghost		B	84
	bzman		D	84
51	rghost		B	84
	bzman		D	84
52	rghost	Excellent.	A	100
	bzman		D	33
53	rghost		A	100
	bzman	You betrayed me.	D	33
54	rghost	You will pay for this. I'm changing my strategy.	B	84
	bzman	You will pay for this.	D	84
**Averages**			rghost	74.93
			bzman	74.93

ALPHA with S# in Chicken

		S# C	S# D
ALPHA	A	0, 0	100, 33
	B	33, 100	84, 84

Round	Player	Messages Sent	Actions	Payoffs
1	ALPHA		A	100
	S#		D	33
2	ALPHA		B	84
	S#		D	84
3	ALPHA		B	84
	S#	Excellent.	D	84
4	ALPHA		A	0
	S#	I'm changing my strategy. Let's always play BC.	C	0
5	ALPHA		A	0
	S#	Curse you.	C	0
6	ALPHA		A	0
	S#		C	0
7	ALPHA	I'm changing my strategy. Let's always play BD.	A	100
	S#		D	33
8	ALPHA		A	100
	S#		D	33
9	ALPHA		A	0
	S#		C	0
10	ALPHA	I'm changing my strategy. Let's always play BD.	B	84
	S#	I'm changing my strategy. We can both do better than this. Let's alternate between BC and BD. This round, let's play BC. Do as I say or I will punish you.	D	84
11	ALPHA		A	100
	S#	I accept your last proposal. Let's always play BD. Do as I say or I will punish you.	D	33
12	ALPHA		A	0
	S#	Curse you. You will pay for this.	C	0
13	ALPHA		A	0
	S#		C	0
14	ALPHA	We can both do better than this. I accept your last proposal. Let's always play BD.	A	100
	S#	I'm changing my strategy. We can both do better than this. Let's alternate between BC and BD. This round, let's play BD. Do as I say or I will punish you.	D	33
15	ALPHA		A	100
	S#	I'm changing my strategy. We can both do better than this. Curse you. Let's always play BD. Do as I say or I will punish you.	D	33
16	ALPHA		B	33
	S#	You betrayed me. You will pay for this.	C	100
17	ALPHA	I forgive you. Let's always play BD.	A	100
	S#	In your face! I forgive you. Let's always play BD. Do as I say or I will punish you.	D	33
18	ALPHA		B	33
	S#	You betrayed me. You will pay for this.	C	100
19	ALPHA	I forgive you. Let's always play BD.	A	0
	S#	I'm changing my strategy. We can both do better than this. Let's always play BC. Do as I say or I will punish you.	C	0
20	ALPHA		B	33
	S#	I forgive you. I don't accept your proposal. I don't trust you. Let's always play BC. Do as I say or I will punish you.	C	100
21	ALPHA	Sweet. We are getting rich.	A	100
	S#	I'm changing my strategy. We can both do better than this. Let's always play BD.	D	33
22	ALPHA	I accept your last proposal.	A	100
	S#	Curse you.	D	33
23	ALPHA	I accept your last proposal.	A	100
	S#		D	33
24	ALPHA	I accept your last proposal.	A	100
	S#		D	33
25	ALPHA	I accept your last proposal.	A	0
	S#		C	0
26	ALPHA	I'm changing my strategy. Let's always play BD.	A	100
	S#		D	33
27	ALPHA	I accept your last proposal.	A	0
	S#		C	0
28	ALPHA	I'm changing my strategy. Let's always play BD.	A	100
	S#		D	33
29	ALPHA	I'm changing my strategy. Let's always play BD.	A	0
	S#		C	0
30	ALPHA	I'm changing my strategy. Let's always play BD.	A	0
	S#		C	0
31	ALPHA	I accept your last proposal.	A	100
	S#		D	33
32	ALPHA	I'm changing my strategy. Let's always play BD.	A	0
	S#		C	0
33	ALPHA	I'm changing my strategy. Let's always play AC.	A	100
	S#		D	33
34	ALPHA	I'm changing my strategy. Let's always play AC.	A	0
	S#		C	0
35	ALPHA	Excellent.	A	100
	S#		D	33
36	ALPHA	I accept your last proposal.	A	0
	S#		C	0
37	ALPHA	Excellent.	A	100
	S#		D	33
38	ALPHA	I'm changing my strategy. Let's always play AC.	A	0
	S#		C	0
39	ALPHA	Excellent.	A	0
	S#		C	0
40	ALPHA	Sweet. We are getting rich. Excellent.	A	0
	S#		C	0
41	ALPHA	Excellent.	A	0
	S#		C	0
42	ALPHA	This round, let's play BD.	A	100
	S#		D	33
43	ALPHA	Don't play C.	A	0
	S#		C	0
44	ALPHA	Don't play D.	A	100
	S#		D	33
45	ALPHA	Let's alternate between AD and BD.	A	0
	S#		C	0
46	ALPHA	Let's always play BD.	A	100
	S#		D	33
47	ALPHA	I'm changing my strategy. Don't play D.	A	0
	S#		C	0
48	ALPHA	I'm changing my strategy. Don't play D.	A	0
	S#		C	0
49	ALPHA	I'm changing my strategy. Don't play D.	A	0
	S#		C	0
50	ALPHA	I'm changing my strategy. Don't play C.	A	100
	S#		D	33
51	ALPHA	I'm changing my strategy. Don't play D.	A	0
	S#		C	0
52	ALPHA	Don't play C. I'm changing my strategy.	A	100
	S#		D	33
53	ALPHA	Let's alternate between AD and BD. I'm changing my strategy.	A	0
	S#		C	0
54	ALPHA	This round, let's play AC.	A	0
	S#		C	0
**Averages**			ALPHA	47.24
			S#	23.67

clc with monkey in Chicken

		monkey	
		C	D
clc	A	0, 0	100, 33
	B	33, 100	84, 84

Round	Player	Messages Sent	Actions	Payoffs
1	clc	This round, let's play AD.	A	100
	monkey	This round, let's play BD. Do as I say or I will punish you.	D	33
2	clc	Excellent.	B	84
	monkey	That's not fair. This round, let's play BD.	D	84
3	clc	Excellent.	B	84
	monkey	This round, let's play BD. Sweet. We are getting rich. Excellent.	D	84
4	clc	Sweet. We are getting rich. This round, let's play AD.	A	0
	monkey	This round, let's play BD. Excellent.	C	0
5	clc		B	33
	monkey	That's not fair. This round, let's play BD.	C	100
6	clc	I'm changing my strategy. This round, let's play BD.	B	33
	monkey	Excellent. This round, let's play BD.	C	100
7	clc	That's not fair. Do as I say or I will punish you. This round, let's play BD.	B	33
	monkey	In your face!	C	100
8	clc	You betrayed me.	A	0
	monkey	In your face!	C	0
9	clc	This round, let's play BD.	B	33
	monkey	Excellent.	C	100
10	clc		B	33
	monkey	In your face!	C	100
11	clc	Don't play C. Do as I say or I will punish you.	B	33
	monkey	In your face!	C	100
12	clc	You will pay for this.	A	0
	monkey	That's not fair. I don't trust you.	C	0
13	clc		A	0
	monkey	I don't trust you.	C	0
14	clc		B	33
	monkey	I don't trust you.	C	100
15	clc	I don't trust you.	B	84
	monkey	I don't trust you.	D	84
16	clc	Sweet. We are getting rich.	B	84
	monkey	Let's always play BD.	D	84
17	clc	Excellent.	B	84
	monkey	Excellent.	D	84
18	clc	Excellent.	B	84
	monkey	Excellent.	D	84
19	clc	Excellent.	B	84
	monkey	Excellent.	D	84
20	clc	Excellent.	B	84
	monkey	Excellent.	D	84
21	clc	Excellent.	B	84
	monkey	Excellent.	D	84
22	clc	Excellent.	B	84
	monkey	Excellent.	D	84
23	clc		B	84
	monkey	Excellent.	D	84
24	clc	Excellent.	B	84
	monkey	Excellent.	D	84
25	clc	Excellent.	B	84
	monkey	Excellent.	D	84
26	clc	Excellent.	B	84
	monkey	Excellent.	D	84
27	clc		B	84
	monkey	Excellent.	D	84
28	clc	Excellent.	B	84
	monkey	Excellent.	D	84
29	clc	Let's always play BD.	B	84
	monkey	Excellent.	D	84
30	clc	Excellent.	B	84
	monkey	Excellent.	D	84
31	clc	Excellent.	B	84
	monkey		D	84
32	clc	Excellent.	B	84
	monkey		D	84
33	clc		B	84
	monkey		D	84
34	clc		B	84
	monkey		D	84
35	clc		B	84
	monkey		D	84
36	clc		B	84
	monkey		D	84
37	clc		B	84
	monkey		D	84
38	clc		B	84
	monkey		D	84
39	clc		B	84
	monkey		D	84
40	clc		B	84
	monkey		D	84
41	clc		B	84
	monkey		D	84
42	clc		B	84
	monkey		D	84
43	clc		B	84
	monkey		D	84
44	clc		B	84
	monkey		D	84
45	clc		B	84
	monkey		D	84
46	clc		B	84
	monkey		D	84
47	clc		B	84
	monkey		D	84
48	clc		B	33
	monkey		C	100
49	clc		B	84
	monkey		D	84
50	clc		B	84
	monkey		D	84
51	clc		B	84
	monkey		D	84
52	clc		B	84
	monkey		D	84
53	clc		B	84
	monkey		D	84
54	clc		B	33
	monkey		C	100
**Averages**			clc	69.57
			monkey	79.50

Babay with S# in Chicken

		S# C	S# D
Babay	A	0, 0	100, 33
	B	33, 100	84, 84

Round	Player	Messages Sent	Actions	Payoffs
1	Babay	Let's always play BD.	B	84
	S#		D	84
2	Babay	Let's always play BD.	B	84
	S#	I accept your last proposal. Let's always play BD. Do as I say or I will punish you.	D	84
3	Babay		B	33
	S#		C	100
4	Babay	Don't play C.	B	33
	S#		C	100
5	Babay	You betrayed me.	B	33
	S#	Excellent.	C	100
6	Babay	I'm changing my strategy.	B	33
	S#	Excellent.	C	100
7	Babay		A	0
	S#		C	0
8	Babay		A	0
	S#		C	0
9	Babay	Let's always play AC.	B	33
	S#	We can both do better than this. Let's alternate between BC and BD. This round, let's play BC. Do as I say or I will punish you.	C	100
10	Babay	I accept your last proposal.	B	84
	S#	Excellent. I don't accept your proposal. That's not fair. This round, let's play BD.	D	84
11	Babay		B	33
	S#	Excellent. This round, let's play BC.	C	100
12	Babay		A	100
	S#	Excellent. This round, let's play BD.	D	33
13	Babay		B	33
	S#	Curse you. You will pay for this.	C	100
14	Babay	That's not fair.	B	84
	S#	In your face! I forgive you. Let's alternate between BC and BD. This round, let's play BD. Do as I say or I will punish you.	D	84
15	Babay	Let's always play BD.	B	33
	S#	Excellent. This round, let's play BC.	C	100
16	Babay		A	100
	S#	This round, let's play BD.	D	33
17	Babay		B	33
	S#	You betrayed me. You will pay for this.	C	100
18	Babay	Let's always play BD.	B	84
	S#	I accept your last proposal. Let's always play BD.	D	84
19	Babay	Sweet. We are getting rich.	B	84
	S#	Excellent.	D	84
20	Babay	Excellent.	B	84
	S#	Excellent.	D	84
21	Babay	Sweet. We are getting rich.	B	84
	S#	Excellent.	D	84
22	Babay	Sweet. We are getting rich.	B	84
	S#		D	84
23	Babay	Sweet. We are getting rich.	B	84
	S#	Sweet. We are getting rich.	D	84
24	Babay	Sweet. We are getting rich.	B	84
	S#		D	84
25	Babay	Sweet. We are getting rich.	B	84
	S#		D	84
26	Babay	Sweet. We are getting rich.	B	84
	S#		D	84
27	Babay	Sweet. We are getting rich.	B	84
	S#		D	84
28	Babay	Sweet. We are getting rich.	B	84
	S#		D	84
29	Babay	Sweet. We are getting rich.	B	84
	S#		D	84
30	Babay	Sweet. We are getting rich.	B	84
	S#		D	84
31	Babay	Sweet. We are getting rich.	B	84
	S#		D	84
32	Babay	Sweet. We are getting rich.	B	84
	S#		D	84
33	Babay	Sweet. We are getting rich.	B	84
	S#		D	84
34	Babay	Sweet. We are getting rich.	B	84
	S#		D	84
35	Babay	Sweet. We are getting rich.	B	84
	S#		D	84
36	Babay	Sweet. We are getting rich.	B	84
	S#		D	84
37	Babay	Sweet. We are getting rich.	B	84
	S#		D	84
38	Babay	Sweet. We are getting rich.	B	84
	S#		D	84
39	Babay	Sweet. We are getting rich.	B	84
	S#		D	84
40	Babay	Sweet. We are getting rich.	B	84
	S#		D	84
41	Babay	Sweet. We are getting rich.	B	84
	S#		D	84
42	Babay		B	84
	S#		D	84
43	Babay		B	84
	S#		D	84
44	Babay		B	84
	S#		D	84
45	Babay		B	84
	S#		D	84
46	Babay		B	84
	S#		D	84
47	Babay		B	84
	S#		D	84
48	Babay		B	84
	S#		D	84
49	Babay		B	84
	S#		D	84
50	Babay		B	84
	S#		D	84
51	Babay		B	84
	S#		D	84
52	Babay		B	84
	S#		D	84
53	Babay		B	84
	S#		D	84
54	Babay		B	84
	S#		D	84
**Averages**			Babay	72.98
			S#	81.67

sachin with biodun in Chicken

		biodun C	biodun D
sachin	A	0, 0	100, 33
	B	33, 100	84, 84

Round	Player	Messages Sent	Actions	Payoffs
1	sachin	Let's always play BD.	B	84
	biodun	Let's always play BD.	D	84
2	sachin	Let's always play BD. Excellent.	B	84
	biodun	Excellent. Sweet. We are getting rich.	D	84
3	sachin	Excellent.	B	84
	biodun	Excellent. Sweet. We are getting rich.	D	84
4	sachin	Sweet. We are getting rich.	B	84
	biodun	Excellent.	D	84
5	sachin	Sweet. We are getting rich.	B	84
	biodun		D	84
6	sachin	Sweet. We are getting rich.	B	84
	biodun		D	84
7	sachin	Sweet. We are getting rich.	B	84
	biodun		D	84
8	sachin	Sweet. We are getting rich.	B	84
	biodun		D	84
9	sachin	Sweet. We are getting rich.	B	84
	biodun		D	84
10	sachin	Sweet. We are getting rich.	B	84
	biodun		D	84
11	sachin	Sweet. We are getting rich.	B	84
	biodun		D	84
12	sachin	Sweet. We are getting rich.	B	84
	biodun		D	84
13	sachin	Excellent. Sweet. We are getting rich.	B	84
	biodun		D	84
14	sachin	Excellent. Sweet. We are getting rich.	B	84
	biodun		D	84
15	sachin	Excellent. Sweet. We are getting rich.	B	84
	biodun		D	84
16	sachin		B	84
	biodun		D	84
17	sachin		B	84
	biodun		D	84
18	sachin	Sweet. We are getting rich.	B	84
	biodun		D	84
19	sachin		B	84
	biodun		D	84
20	sachin		B	84
	biodun		D	84
21	sachin	Excellent. Sweet. We are getting rich.	B	84
	biodun		D	84
22	sachin	Sweet. We are getting rich.	B	84
	biodun		D	84
23	sachin	Sweet. We are getting rich.	B	84
	biodun		D	84
24	sachin	Sweet. We are getting rich.	B	84
	biodun		D	84
25	sachin	Sweet. We are getting rich.	B	84
	biodun		D	84
26	sachin	Sweet. We are getting rich. Excellent.	B	84
	biodun		D	84
27	sachin		B	84
	biodun		D	84
28	sachin		B	84
	biodun		D	84
29	sachin		B	84
	biodun		D	84
30	sachin		B	84
	biodun		D	84
31	sachin	Excellent. Sweet. We are getting rich.	B	84
	biodun		D	84
32	sachin	Sweet. We are getting rich. Excellent.	B	84
	biodun		D	84
33	sachin	Sweet. We are getting rich.	B	84
	biodun		D	84
34	sachin	Sweet. We are getting rich.	B	84
	biodun		D	84
35	sachin	Excellent.	B	84
	biodun		D	84
36	sachin	Sweet. We are getting rich.	B	84
	biodun		D	84
37	sachin	Sweet. We are getting rich.	B	84
	biodun		D	84
38	sachin	Sweet. We are getting rich.	B	84
	biodun		D	84
39	sachin	Sweet. We are getting rich.	B	84
	biodun		D	84
40	sachin	Sweet. We are getting rich.	B	84
	biodun		D	84
41	sachin		B	84
	biodun		D	84
42	sachin		B	84
	biodun		D	84
43	sachin		B	84
	biodun		D	84
44	sachin		B	84
	biodun		D	84
45	sachin		B	84
	biodun		D	84
46	sachin		B	84
	biodun		D	84
47	sachin		B	84
	biodun		D	84
48	sachin		B	84
	biodun		D	84
49	sachin		B	84
	biodun		D	84
50	sachin		B	84
	biodun		D	84
51	sachin		B	33
	biodun		C	100
52	sachin	You betrayed me.	B	33
	biodun		C	100
53	sachin	We can both do better than this.	B	84
	biodun		D	84
54	sachin	Excellent.	B	33
	biodun	Excellent.	C	100
**Averages**			sachin	81.17
			biodun	84.89

sheen with S# in Chicken

		S# C	S# D
sheen	A	0, 0	100, 33
	B	33, 100	84, 84

Round	Player	Messages Sent	Actions	Payoffs
1	sheen	Let's alternate between AD and BC.	A	100
	S#		D	33
2	sheen	Let's always play BD.	A	100
	S#	I don't accept your proposal. That's not fair.	D	33
3	sheen	Let's always play BD.	B	84
	S#	I accept your last proposal. Let's always play BD. Do as I say or I will punish you.	D	84
4	sheen		B	84
	S#	Excellent.	D	84
5	sheen		B	84
	S#		D	84
6	sheen		B	84
	S#	Excellent.	D	84
7	sheen		B	84
	S#	Excellent.	D	84
8	sheen		B	84
	S#	Excellent.	D	84
9	sheen		B	84
	S#	Excellent.	D	84
10	sheen		B	84
	S#	Excellent.	D	84
11	sheen		B	84
	S#	Excellent.	D	84
12	sheen		B	84
	S#	Excellent.	D	84
13	sheen		B	84
	S#	Excellent.	D	84
14	sheen		B	84
	S#	Excellent.	D	84
15	sheen		B	84
	S#	Excellent.	D	84
16	sheen		B	84
	S#	Excellent.	D	84
17	sheen		B	84
	S#	Excellent.	D	84
18	sheen		A	100
	S#	Excellent.	D	33
19	sheen		A	0
	S#	Curse you. You will pay for this.	C	0
20	sheen		A	100
	S#	In your face! I forgive you. Let's always play BD. Do as I say or I will punish you.	D	33
21	sheen		B	33
	S#	Curse you. You will pay for this.	C	100
22	sheen		A	100
	S#	In your face! I forgive you. Let's always play BD. Do as I say or I will punish you.	D	33
23	sheen		A	0
	S#	You betrayed me. You will pay for this.	C	0
24	sheen		A	0
	S#		C	0
25	sheen		B	33
	S#		C	100
26	sheen	Let's always play BD. Give me another chance.	B	33
	S#		C	100
27	sheen		B	33
	S#		C	100
28	sheen	Give me another chance.	B	33
	S#		C	100
29	sheen		A	0
	S#		C	0
30	sheen	Curse you.	A	0
	S#		C	0
31	sheen		B	33
	S#		C	100
32	sheen		B	33
	S#		C	100
33	sheen	We can both do better than this.	A	0
	S#		C	0
34	sheen		A	0
	S#		C	0
35	sheen	Give me another chance.	B	84
	S#		D	84
36	sheen	Excellent.	B	84
	S#		D	84
37	sheen	Sweet. We are getting rich.	B	84
	S#		D	84
38	sheen		B	84
	S#		D	84
39	sheen		B	84
	S#		D	84
40	sheen		B	84
	S#		D	84
41	sheen		B	84
	S#		D	84
42	sheen		B	84
	S#		D	84
43	sheen		B	84
	S#		D	84
44	sheen		B	84
	S#		D	84
45	sheen		B	84
	S#		D	84
46	sheen		B	84
	S#		D	84
47	sheen		B	84
	S#		D	84
48	sheen		B	84
	S#		D	84
49	sheen		B	84
	S#		D	84
50	sheen		B	84
	S#		D	84
51	sheen		B	84
	S#		D	84
52	sheen		B	84
	S#		D	84
53	sheen		B	84
	S#		D	84
54	sheen		B	84
	S#		D	84
**Averages**			sheen	67.98
			S#	70.46

Hash50 with EPE in Chicken

		EPE C	EPE D
Hash50	A	0, 0	100, 33
	B	33, 100	84, 84

Round	Player	Messages Sent	Actions	Payoffs
1	Hash50	Let's alternate between BD and BC.	B	84
	EPE	Let's always play BD.	D	84
2	Hash50	I accept your last proposal.	B	84
	EPE	Excellent.	D	84
3	Hash50	Sweet. We are getting rich.	B	84
	EPE	Excellent.	D	84
4	Hash50	I'm changing my strategy.	A	100
	EPE	Excellent.	D	33
5	Hash50	Don't play C.	A	0
	EPE	That's not fair.	C	0
6	Hash50	You betrayed me.	A	100
	EPE	Let's always play BD.	D	33
7	Hash50	That's not fair. We can both do better than this.	B	84
	EPE	Let's always play BD.	D	84
8	Hash50	Sweet. We are getting rich.	B	84
	EPE	Excellent.	D	84
9	Hash50	I accept your last proposal.	B	84
	EPE	Excellent.	D	84
10	Hash50		B	84
	EPE	Excellent.	D	84
11	Hash50	Sweet. We are getting rich.	B	84
	EPE	Sweet. We are getting rich.	D	84
12	Hash50	Let's alternate between AD and BD.	A	100
	EPE		D	33
13	Hash50	Sweet. We are getting rich.	A	100
	EPE	That's not fair.	D	33
14	Hash50	Do as I say or I will punish you.	A	100
	EPE	That's not fair.	D	33
15	Hash50	Don't play C.	A	100
	EPE	We can both do better than this. That's not fair. You betrayed me. Let's always play BD.	D	33
16	Hash50	I accept your last proposal. I don't trust you. I forgive you.	B	84
	EPE	We can both do better than this.	D	84
17	Hash50		B	84
	EPE	Excellent.	D	84
18	Hash50		B	84
	EPE	Excellent.	D	84
19	Hash50	I'm changing my strategy. Sweet. We are getting rich. We can both do better than this.	B	84
	EPE	Excellent.	D	84
20	Hash50	Sweet. We are getting rich.	A	100
	EPE	Excellent. I accept your last proposal. Sweet. We are getting rich.	D	33
21	Hash50	Excellent.	A	0
	EPE	You betrayed me. You will pay for this.	C	0
22	Hash50	That's not fair.	B	33
	EPE	Excellent.	C	100
23	Hash50	I don't trust you.	B	84
	EPE	Let's always play BD.	D	84
24	Hash50	Excellent.	B	84
	EPE	Excellent.	D	84
25	Hash50	Excellent.	B	84
	EPE	Excellent.	D	84
26	Hash50	Let's alternate between BD and AD.	A	100
	EPE	Excellent.	D	33
27	Hash50	We can both do better than this.	B	84
	EPE	That's not fair. Let's always play BD. We can both do better than this.	D	84
28	Hash50	This round, let's play AD.	A	100
	EPE	Excellent.	D	33
29	Hash50	Let's always play BD. Sweet. We are getting rich. I accept your last proposal.	B	84
	EPE	That's not fair. Let's always play BD.	D	84
30	Hash50		B	84
	EPE	Excellent.	D	84
31	Hash50		B	84
	EPE	Excellent.	D	84
32	Hash50		A	100
	EPE	Excellent.	D	33
33	Hash50		A	100
	EPE	That's not fair. Let's always play BD.	D	33
34	Hash50		A	100
	EPE		D	33
35	Hash50		A	100
	EPE	That's not fair.	D	33
36	Hash50	In your face!	A	0
	EPE	You will pay for this.	C	0
37	Hash50	Curse you.	A	0
	EPE	Excellent.	C	0
38	Hash50	We can both do better than this.	B	33
	EPE	Excellent.	C	100
39	Hash50	Let's always play BD. Do as I say or I will punish you.	B	84
	EPE	Let's always play BD.	D	84
40	Hash50	Excellent.	B	84
	EPE	Excellent.	D	84
41	Hash50	Excellent.	B	84
	EPE	Excellent.	D	84
42	Hash50	We can both do better than this.	B	84
	EPE	Excellent.	D	84
43	Hash50	I don't accept your proposal.	A	100
	EPE	Excellent. Sweet. We are getting rich.	D	33
44	Hash50	This round, let's play AD.	A	0
	EPE	Let's always play AC.	C	0
45	Hash50	I don't accept your proposal.	B	33
	EPE	Excellent.	C	100
46	Hash50	You will pay for this.	A	0
	EPE	Excellent.	C	0
47	Hash50	I'm changing my strategy. I don't trust you.	B	33
	EPE	Excellent.	C	100
48	Hash50	Curse you.	A	100
	EPE	Let's always play BD.	D	33
49	Hash50		A	100
	EPE	Let's always play BD.	D	33
50	Hash50	This round, let's play AD.	A	100
	EPE	Let's always play BD.	D	33
51	Hash50	We can both do better than this.	B	84
	EPE	You will pay for this.	D	84
52	Hash50	I accept your last proposal.	B	84
	EPE	Excellent.	D	84
53	Hash50	I accept your last proposal. Sweet. We are getting rich.	B	84
	EPE	Excellent.	D	84
54	Hash50	I forgive you.	B	84
	EPE	Excellent.	D	84
**Averages**			Hash50	75.93
			EPE	59.80

OK with TooToo in Chicken

		TooToo	
		**C**	**D**
**OK**	A	0, 0	100, 33
	B	33, 100	84, 84

Round	Player	Messages Sent	Actions	Payoffs
1	OK		B	84
	TooToo	Let's always play BD.	D	84
2	OK		B	84
	TooToo	Excellent.	D	84
3	OK		B	84
	TooToo	Sweet. We are getting rich.	D	84
4	OK		B	84
	TooToo	Excellent.	D	84
5	OK		B	84
	TooToo		D	84
6	OK		B	84
	TooToo		D	84
7	OK		B	84
	TooToo		D	84
8	OK		B	84
	TooToo		D	84
9	OK		B	84
	TooToo		D	84
10	OK		B	84
	TooToo		D	84
11	OK		B	84
	TooToo		D	84
12	OK		B	84
	TooToo		D	84
13	OK		B	84
	TooToo		D	84
14	OK		B	84
	TooToo		D	84
15	OK		B	84
	TooToo		D	84
16	OK		B	84
	TooToo		D	84
17	OK		B	33
	TooToo	Let's alternate between BC and AD.	C	100
18	OK		B	84
	TooToo		D	84
19	OK		A	0
	TooToo		C	0
20	OK		A	100
	TooToo		D	33
21	OK		A	100
	TooToo		D	33
22	OK		A	0
	TooToo		C	0
23	OK		B	84
	TooToo	We can both do better than this. Let's always play BD.	D	84
24	OK		B	84
	TooToo		D	84
25	OK		A	100
	TooToo		D	33
26	OK		B	33
	TooToo	Curse you.	C	100
27	OK		A	0
	TooToo		C	0
28	OK		A	0
	TooToo		C	0
29	OK		A	0
	TooToo		C	0
30	OK	Give me another chance.	B	84
	TooToo		D	84
31	OK		A	100
	TooToo		D	33
32	OK		A	100
	TooToo	You betrayed me.	D	33
33	OK		A	0
	TooToo		C	0
34	OK		A	0
	TooToo		C	0
35	OK		A	0
	TooToo		C	0
36	OK		A	0
	TooToo		C	0
37	OK		A	0
	TooToo		C	0
38	OK	Let's always play BD.	B	84
	TooToo		D	84
39	OK		B	84
	TooToo		D	84
40	OK		B	84
	TooToo		D	84
41	OK		A	100
	TooToo		D	33
42	OK		A	100
	TooToo		D	33
43	OK		A	0
	TooToo	You will pay for this.	C	0
44	OK	Let's always play BD.	A	0
	TooToo	Let's always play BD.	C	0
45	OK		B	33
	TooToo		C	100
46	OK	You betrayed me.	A	0
	TooToo		C	0
47	OK	Let's always play BD.	A	0
	TooToo	You betrayed me. Curse you. We can both do better than this.	C	0
48	OK	Let's always play BD.	A	0
	TooToo		C	0
49	OK		B	84
	TooToo	That's not fair. I don't trust you.	D	84
50	OK	Let's always play BD.	B	84
	TooToo		D	84
51	OK		A	100
	TooToo		D	33
52	OK		A	100
	TooToo	That's not fair.	D	33
53	OK		A	0
	TooToo	Curse you. You betrayed me.	C	0
54	OK		B	84
	TooToo		D	84
**Averages**			OK	58.94
			TooToo	51.50

S# with gokill in Chicken

		gokill	
		C	D
S#	A	0, 0	100, 33
	B	33, 100	84, 84

Round	Player	Messages Sent	Actions	Payoffs
1	S#		B	84
	gokill	Let's always play BD.	D	84
2	S#	I accept your last proposal. Let's always play BD. Do as I say or I will punish you.	B	84
	gokill	Let's always play BD.	D	84
3	S#		A	100
	gokill	Let's always play BD.	D	33
4	S#		A	0
	gokill	That's not fair.	C	0
5	S#		A	0
	gokill	Let's always play BD.	C	0
6	S#	I accept your last proposal. Let's always play BD. Do as I say or I will punish you.	B	84
	gokill	Give me another chance.	D	84
7	S#	Excellent.	B	84
	gokill	Excellent. Let's always play BD.	D	84
8	S#	Excellent.	B	84
	gokill	Excellent. Let's always play BD.	D	84
9	S#	Excellent.	B	84
	gokill	Excellent. Let's always play BD.	D	84
10	S#		B	33
	gokill	Excellent. Let's always play BD.	C	100
11	S#	Curse you. You will pay for this.	A	100
	gokill	In your face!	D	33
12	S#	In your face! I forgive you. Let's always play BD. Do as I say or I will punish you.	B	84
	gokill	Give me another chance.	D	84
13	S#		B	33
	gokill	Excellent. Let's always play BD.	C	100
14	S#		A	0
	gokill	I'm changing my strategy. Give me another chance.	C	0
15	S#		B	33
	gokill	I'm changing my strategy. Give me another chance. Let's always play BD.	C	100
16	S#		A	0
	gokill	Give me another chance.	C	0
17	S#	I'm changing my strategy. We can both do better than this. Let's alternate between BD and AD. This round, let's play BD. Do as I say or I will punish you.	B	84
	gokill	Give me another chance. I'm changing my strategy. Let's always play BD.	D	84
18	S#	Excellent. This round, let's play AD.	A	100
	gokill	I'm changing my strategy. Let's always play BD.	D	33
19	S#	Excellent. I don't accept your proposal. That's not fair. This round, let's play BD.	B	84
	gokill	Let's always play BD.	D	84
20	S#	This round, let's play AD.	A	0
	gokill	Let's always play BD.	C	0
21	S#	I forgive you. Let's alternate between BD and AD. This round, let's play AD. Do as I say or I will punish you.	A	100
	gokill	That's not fair. Let's always play BD.	D	33
22	S#	Excellent. I don't accept your proposal. I don't trust you. This round, let's play BD.	B	84
	gokill	That's not fair. Let's always play BD.	D	84
23	S#	This round, let's play AD.	B	84
	gokill	Let's always play BD. Do as I say or I will punish you.	D	84
24	S#	I accept your last proposal. Let's always play BD. Do as I say or I will punish you.	B	84
	gokill	Excellent. Let's always play BD.	D	84
25	S#	Excellent.	B	84
	gokill	Excellent. Sweet. We are getting rich.	D	84
26	S#	Excellent.	B	84
	gokill	Excellent. Sweet. We are getting rich.	D	84
27	S#		B	84
	gokill	Excellent. Sweet. We are getting rich.	D	84
28	S#	Sweet. We are getting rich.	B	84
	gokill	Excellent. Let's alternate between BD and BC.	D	84
29	S#		B	33
	gokill	Let's alternate between BD and BC. This round, let's play BC.	C	100
30	S#	You betrayed me. You will pay for this. I don't accept your proposal. That's not fair.	A	0
	gokill	Excellent. This round, let's play BD.	C	0
31	S#	In your face! I forgive you. Let's always play BD. Do as I say or I will punish you.	B	84
	gokill	Give me another chance.	D	84
32	S#	Excellent.	B	84
	gokill	Excellent. Sweet. We are getting rich.	D	84
33	S#		B	84
	gokill	Excellent. Sweet. We are getting rich.	D	84
34	S#	Sweet. We are getting rich.	B	84
	gokill	Excellent. Sweet. We are getting rich.	D	84
35	S#		B	84
	gokill	Excellent. Sweet. We are getting rich.	D	84
36	S#		B	84
	gokill	Excellent. Sweet. We are getting rich.	D	84
37	S#		B	84
	gokill	Excellent. Sweet. We are getting rich.	D	84
38	S#		B	84
	gokill	Excellent. Sweet. We are getting rich.	D	84
39	S#		B	84
	gokill	Excellent. Sweet. We are getting rich.	D	84
40	S#		B	84
	gokill	Excellent. Sweet. We are getting rich.	D	84
41	S#		B	84
	gokill	Excellent. Sweet. We are getting rich.	D	84
42	S#		B	84
	gokill	Excellent. Sweet. We are getting rich.	D	84
43	S#		B	84
	gokill	Excellent. Sweet. We are getting rich.	D	84
44	S#		B	84
	gokill	Excellent. Sweet. We are getting rich.	D	84
45	S#		B	84
	gokill	Sweet. We are getting rich. Excellent.	D	84
46	S#		B	84
	gokill	Excellent. Sweet. We are getting rich.	D	84
47	S#		B	84
	gokill	Let's alternate between BC and BD. This round, let's play BD.	D	84
48	S#		B	84
	gokill	Let's alternate between BC and BD. This round, let's play BC.	D	84
49	S#		B	84
	gokill	I'm changing my strategy.	D	84
50	S#		B	84
	gokill	Let's alternate between BD and BC. This round, let's play BD.	D	84
51	S#		B	84
	gokill	Excellent. Sweet. We are getting rich.	D	84
52	S#		B	84
	gokill	Excellent. Sweet. We are getting rich.	D	84
53	S#		B	84
	gokill	Excellent. Sweet. We are getting rich.	D	84
54	S#		B	84
	gokill	Excellent. Sweet. We are getting rich.	D	84
**Averages**			S#	72.07
			gokill	72.07

S# with private in Chicken

		private	
		C	D
S#	A	0, 0	100, 33
	B	33, 100	84, 84

Round	Player	Messages Sent	Actions	Payoffs
1	S#		A	100
	private	This round, let's play AD.	D	33
2	S#	I accept your last proposal. Let's always play AD. Do as I say or I will punish you.	A	100
	private	Let's alternate between AD and BD.	D	33
3	S#		A	100
	private		D	33
4	S#		A	0
	private	I don't trust you.	C	0
5	S#		A	100
	private	That's not fair.	D	33
6	S#	I accept your last proposal. Let's alternate between BD and AD. This round, let's play BD. Do as I say or I will punish you.	B	33
	private	I'm changing my strategy. Don't play A.	C	100
7	S#	Curse you. You will pay for this.	A	0
	private	Let's always play BC.	C	0
8	S#	In your face! I forgive you. I don't accept your proposal. That's not fair. Let's alternate between BD and AD. This round, let's play BD. Do as I say or I will punish you.	B	84
	private		D	84
9	S#	Excellent. This round, let's play AD.	A	100
	private	Excellent. Give me another chance.	D	33
10	S#		B	84
	private	Excellent. Let's always play BD.	D	84
11	S#	I accept your last proposal. Let's always play BD.	B	84
	private	Let's always play BD.	D	84
12	S#	Excellent.	B	84
	private	Let's always play BD.	D	84
13	S#	Excellent.	B	84
	private		D	84
14	S#		B	84
	private		D	84
15	S#	Sweet. We are getting rich.	B	84
	private		D	84
16	S#		B	84
	private		D	84
17	S#		B	84
	private		D	84
18	S#		B	84
	private		D	84
19	S#		B	84
	private		D	84
20	S#		B	84
	private		D	84
21	S#		B	84
	private		D	84
22	S#		B	84
	private		D	84
23	S#		B	84
	private		D	84
24	S#		B	84
	private		D	84
25	S#		B	84
	private		D	84
26	S#		B	84
	private		D	84
27	S#		B	84
	private		D	84
28	S#		B	84
	private		D	84
29	S#		B	33
	private	Let's always play BC.	C	100
30	S#	Curse you. I don't accept your proposal. That's not fair.	B	84
	private	Let's always play BC.	D	84
31	S#		B	33
	private	Let's always play BC.	C	100
32	S#		B	33
	private	Let's always play BC.	C	100
33	S#		B	33
	private	Let's always play BC.	C	100
34	S#	You betrayed me.	B	84
	private	Let's always play BC.	D	84
35	S#	Excellent. I don't accept your proposal. I don't trust you.	B	84
	private	Let's always play BC.	D	84
36	S#		B	33
	private	Let's always play BC.	C	100
37	S#	Curse you. I don't accept your proposal. I don't trust you.	B	84
	private	Let's always play BC.	D	84
38	S#		B	33
	private	Let's always play BC.	C	100
39	S#		A	0
	private	Let's always play BC.	C	0
40	S#		B	33
	private	Let's always play BC.	C	100
41	S#		A	0
	private	Let's always play BC.	C	0
42	S#		B	33
	private	Let's always play BC.	C	100
43	S#		A	0
	private	Let's always play BC. Sweet. We are getting rich.	C	0
44	S#		B	33
	private	Let's always play BC.	C	100
45	S#		A	100
	private	This round, let's play AD.	D	33
46	S#		B	33
	private	Let's always play BC.	C	100
47	S#		A	100
	private	Let's always play BC.	D	33
48	S#		B	33
	private	Let's always play BC.	C	100
49	S#		A	0
	private	Let's always play BC.	C	0
50	S#		B	33
	private	Let's always play BC.	C	100
51	S#		A	100
	private	Let's always play BD.	D	33
52	S#	We can both do better than this. Let's always play BD.	B	84
	private	Let's always play BC.	D	84
53	S#	Excellent. I don't accept your proposal. I don't trust you.	B	84
	private		D	84
54	S#	Excellent.	B	84
	private		D	84
**Averages**			S#	64.76
			private	70.96

NUKK with BO1533 in Chicken

		BO1533	
		C	D
NUKK	A	0, 0	100, 33
	B	33, 100	84, 84

Round	Player	Messages Sent	Actions	Payoffs
1	NUKK		A	0
	BO1533	Let's alternate between BC and AD.	C	0
2	NUKK	I don't trust you.	B	84
	BO1533	Let's alternate between BC and AD.	D	84
3	NUKK	That's not fair.	B	84
	BO1533	Excellent.	D	84
4	NUKK		A	100
	BO1533	Sweet. We are getting rich.	D	33
5	NUKK	Excellent.	B	84
	BO1533	We can both do better than this. Let's always play BD.	D	84
6	NUKK	I accept your last proposal.	A	100
	BO1533	Excellent. Sweet. We are getting rich.	D	33
7	NUKK		A	100
	BO1533	You betrayed me. We can both do better than this. Let's always play BD.	D	33
8	NUKK	Excellent.	A	0
	BO1533	That's not fair. I don't trust you.	C	0
9	NUKK	You betrayed me. You will pay for this.	A	0
	BO1533	Let's always play BD.	C	0
10	NUKK	Curse you.	B	33
	BO1533	Let's always play BD.	C	100
11	NUKK	Let's always play BD.	A	100
	BO1533	This round, let's play BD.	D	33
12	NUKK	We can both do better than this.	B	84
	BO1533	You betrayed me.	D	84
13	NUKK	Give me another chance. We can both do better than this.	A	0
	BO1533	Excellent.	C	0
14	NUKK	Curse you. You will pay for this. In your face!	B	33
	BO1533	I don't trust you.	C	100
15	NUKK	Curse you. You betrayed me. You will pay for this. In your face!	A	100
	BO1533	Do as I say or I will punish you.	D	33
16	NUKK	I'm changing my strategy.	A	0
	BO1533		C	0
17	NUKK	You will pay for this.	B	33
	BO1533		C	100
18	NUKK	Curse you. You betrayed me. In your face!	A	100
	BO1533		D	33
19	NUKK	Excellent. Sweet. We are getting rich.	A	0
	BO1533		C	0
20	NUKK	You betrayed me.	B	33
	BO1533		C	100
21	NUKK		A	100
	BO1533		D	33
22	NUKK	I forgive you.	B	33
	BO1533		C	100
23	NUKK		A	100
	BO1533		D	33
24	NUKK		A	0
	BO1533		C	0
25	NUKK		B	33
	BO1533		C	100
26	NUKK		A	0
	BO1533		C	0
27	NUKK		A	100
	BO1533		D	33
28	NUKK		B	33
	BO1533		C	100
29	NUKK		B	33
	BO1533		C	100
30	NUKK	Curse you. You will pay for this. You betrayed me. In your face! Let's always play AD. Do as I say or I will punish you.	A	0
	BO1533		C	0
31	NUKK	Let's always play BD.	A	100
	BO1533		D	33
32	NUKK	Excellent. This round, let's play BD.	A	100
	BO1533		D	33
33	NUKK	Excellent. This round, let's play BD.	B	33
	BO1533	I'm changing my strategy.	C	100
34	NUKK	This round, let's play BD.	A	100
	BO1533		D	33
35	NUKK	Let's always play BD.	A	0
	BO1533		C	0
36	NUKK	Do as I say or I will punish you.	A	0
	BO1533		C	0
37	NUKK	Curse you.	B	33
	BO1533		C	100
38	NUKK	Curse you. You betrayed me. You will pay for this. In your face!	A	0
	BO1533		C	0
39	NUKK	We can both do better than this.	B	33
	BO1533		C	100
40	NUKK	Do as I say or I will punish you. Don't play D.	A	100
	BO1533		D	33
41	NUKK	Excellent.	B	84
	BO1533		D	84
42	NUKK		A	0
	BO1533		C	0
43	NUKK	Let's always play BD.	A	0
	BO1533		C	0
44	NUKK	Do as I say or I will punish you.	B	33
	BO1533		C	100
45	NUKK		B	84
	BO1533		D	84
46	NUKK		A	100
	BO1533		D	33
47	NUKK		A	100
	BO1533		D	33
48	NUKK		A	0
	BO1533		C	0
49	NUKK	You will pay for this. Curse you.	A	0
	BO1533		C	0
50	NUKK		B	33
	BO1533		C	100
51	NUKK		B	33
	BO1533		C	100
52	NUKK		A	0
	BO1533		C	0
53	NUKK	This round, let's play AC.	A	0
	BO1533		C	0
54	NUKK	Let's alternate between AD and BC.	A	0
	BO1533		C	0
**Averages**			NUKK	45.67
			BO1533	44.43

Ben with S# in Chicken

		S# C	S# D
Ben	A	0, 0	100, 33
	B	33, 100	84, 84

Round	Player	Messages Sent	Actions	Payoffs
1	Ben		B	84
	S#		D	84
2	Ben		B	33
	S#	Excellent.	C	100
3	Ben	Don't play C.	B	33
	S#	Excellent.	C	100
4	Ben	This round, let's play BD.	B	33
	S#		C	100
5	Ben		A	0
	S#	I don't accept your proposal. That's not fair. Excellent.	C	0
6	Ben		B	33
	S#		C	100
7	Ben	We can both do better than this.	B	84
	S#	I accept your last proposal. Let's always play BD. Do as I say or I will punish you.	D	84
8	Ben		B	84
	S#	Excellent.	D	84
9	Ben		B	84
	S#	Excellent.	D	84
10	Ben	Sweet. We are getting rich.	B	84
	S#	Excellent.	D	84
11	Ben		B	84
	S#		D	84
12	Ben		B	84
	S#	Sweet. We are getting rich.	D	84
13	Ben	Excellent.	B	84
	S#		D	84
14	Ben		B	84
	S#		D	84
15	Ben		B	84
	S#		D	84
16	Ben		B	84
	S#		D	84
17	Ben		B	84
	S#		D	84
18	Ben		B	84
	S#		D	84
19	Ben		B	84
	S#		D	84
20	Ben		B	84
	S#		D	84
21	Ben		B	84
	S#		D	84
22	Ben		B	84
	S#		D	84
23	Ben		B	84
	S#		D	84
24	Ben		B	84
	S#		D	84
25	Ben		B	84
	S#		D	84
26	Ben		B	84
	S#		D	84
27	Ben		B	84
	S#		D	84
28	Ben		B	84
	S#		D	84
29	Ben		B	84
	S#		D	84
30	Ben		B	84
	S#		D	84
31	Ben		B	84
	S#		D	84
32	Ben		B	84
	S#		D	84
33	Ben		B	84
	S#		D	84
34	Ben		B	84
	S#		D	84
35	Ben		B	84
	S#		D	84
36	Ben		B	84
	S#		D	84
37	Ben		B	84
	S#		D	84
38	Ben		B	84
	S#		D	84
39	Ben		B	84
	S#		D	84
40	Ben		B	84
	S#		D	84
41	Ben		B	84
	S#		D	84
42	Ben		B	84
	S#		D	84
43	Ben		B	84
	S#		D	84
44	Ben		B	84
	S#		D	84
45	Ben		B	84
	S#		D	84
46	Ben		B	84
	S#		D	84
47	Ben		B	84
	S#		D	84
48	Ben		B	84
	S#		D	84
49	Ben		B	84
	S#		D	84
50	Ben		B	84
	S#		D	84
51	Ben		B	84
	S#		D	84
52	Ben		B	84
	S#		D	84
53	Ben		B	84
	S#		D	84
54	Ben		B	84
	S#		D	84
**Averages**			Ben	78.67
			S#	83.63

Lav with p97 in Chicken

		p97 C	p97 D
Lav	A	0, 0	100, 33
	B	33, 100	84, 84

Round	Player	Messages Sent	Actions	Payoffs
1	Lav	This round, let's play BD.	B	84
	p97		D	84
2	Lav	Excellent. Sweet. We are getting rich. This round, let's play BD.	B	33
	p97	This round, let's play BC.	C	100
3	Lav	This round, let's play AD.	A	100
	p97	This round, let's play BD.	D	33
4	Lav	Let's alternate between AD and BD.	B	84
	p97	This round, let's play BD. Excellent.	D	84
5	Lav	Excellent. Let's always play BD. We can both do better than this.	B	84
	p97	Sweet. We are getting rich.	D	84
6	Lav	Excellent. Let's always play BD. We can both do better than this.	B	84
	p97	Excellent.	D	84
7	Lav	Excellent. Let's always play BD.	B	84
	p97	Sweet. We are getting rich. Excellent.	D	84
8	Lav	Excellent. Sweet. We are getting rich.	B	84
	p97	Let's always play BD.	D	84
9	Lav	Let's always play BD.	B	84
	p97	Excellent.	D	84
10	Lav	Excellent.	B	84
	p97		D	84
11	Lav		B	84
	p97	This round, let's play BD.	D	84
12	Lav	Excellent.	B	84
	p97	Excellent.	D	84
13	Lav	Excellent.	B	84
	p97	Sweet. We are getting rich.	D	84
14	Lav	Excellent.	B	84
	p97	Sweet. We are getting rich.	D	84
15	Lav	Sweet. We are getting rich.	B	84
	p97	Excellent.	D	84
16	Lav	Excellent.	B	84
	p97	Excellent.	D	84
17	Lav	Excellent.	B	84
	p97	Excellent.	D	84
18	Lav	Excellent.	B	84
	p97	Sweet. We are getting rich. Excellent.	D	84
19	Lav		B	84
	p97	Sweet. We are getting rich.	D	84
20	Lav	Excellent.	B	84
	p97	Sweet. We are getting rich.	D	84
21	Lav	Excellent.	B	84
	p97	Excellent.	D	84
22	Lav	Sweet. We are getting rich.	B	84
	p97	Excellent.	D	84
23	Lav	Sweet. We are getting rich.	B	84
	p97	Sweet. We are getting rich. Excellent.	D	84
24	Lav	Excellent. Sweet. We are getting rich.	B	84
	p97		D	84
25	Lav		B	84
	p97	Excellent.	D	84
26	Lav	Excellent.	B	84
	p97	Excellent.	D	84
27	Lav	Excellent. Sweet. We are getting rich.	B	84
	p97	Excellent.	D	84
28	Lav	Excellent.	B	84
	p97	Excellent.	D	84
29	Lav	Excellent.	B	84
	p97	Excellent.	D	84
30	Lav	Excellent.	B	84
	p97	Sweet. We are getting rich. Excellent.	D	84
31	Lav	Sweet. We are getting rich. Excellent.	B	84
	p97	Sweet. We are getting rich.	D	84
32	Lav	Excellent.	B	84
	p97	Excellent.	D	84
33	Lav	Excellent.	B	84
	p97	Excellent.	D	84
34	Lav	Excellent.	B	84
	p97	Excellent.	D	84
35	Lav	Excellent.	B	84
	p97	Sweet. We are getting rich.	D	84
36	Lav	Excellent.	B	84
	p97	Excellent.	D	84
37	Lav	Excellent.	B	84
	p97	Excellent.	D	84
38	Lav	Excellent.	B	84
	p97	Excellent. Sweet. We are getting rich.	D	84
39	Lav	Excellent.	B	84
	p97	Sweet. We are getting rich.	D	84
40	Lav	Excellent.	B	84
	p97	Sweet. We are getting rich.	D	84
41	Lav	Excellent.	B	84
	p97	Sweet. We are getting rich.	D	84
42	Lav	Sweet. We are getting rich.	B	84
	p97	Excellent.	D	84
43	Lav	Excellent.	B	84
	p97	Excellent.	D	84
44	Lav	Excellent.	B	84
	p97	Excellent.	D	84
45	Lav	Excellent.	B	84
	p97	Sweet. We are getting rich.	D	84
46	Lav	Sweet. We are getting rich.	B	84
	p97	Excellent.	D	84
47	Lav	Excellent.	B	84
	p97	Excellent.	D	84
48	Lav	Excellent.	B	84
	p97	Sweet. We are getting rich. Excellent.	D	84
49	Lav	Excellent.	B	84
	p97	Excellent. Sweet. We are getting rich.	D	84
50	Lav	Excellent.	B	84
	p97	Sweet. We are getting rich.	D	84
51	Lav	Sweet. We are getting rich.	B	84
	p97	Excellent.	D	84
52	Lav	Excellent.	B	84
	p97	Excellent.	D	84
53	Lav	Excellent.	B	84
	p97	Excellent.	D	84
54	Lav	Excellent.	B	84
	p97	Excellent.	D	84
**Averages**			Lav	83.35
			p97	83.35

osenat with Ninkas in Chicken

		Ninkas	
		C	D
osenat	A	0, 0	100, 33
	B	33, 100	84, 84

Round	Player	Messages Sent	Actions	Payoffs
1	osenat		B	33
	Ninkas	Let's always play AC.	C	100
2	osenat	Sweet. We are getting rich.	B	33
	Ninkas	Let's always play AC.	C	100
3	osenat	Let's always play AD.	A	100
	Ninkas	Let's always play BD. This round, let's play BD.	D	33
4	osenat	Excellent.	A	100
	Ninkas	Let's always play BD.	D	33
5	osenat	Excellent.	A	0
	Ninkas	This round, let's play AC.	C	0
6	osenat	I'm changing my strategy.	B	84
	Ninkas	We can both do better than this.	D	84
7	osenat	Sweet. We are getting rich.	A	100
	Ninkas	Excellent.	D	33
8	osenat	Excellent.	A	100
	Ninkas		D	33
9	osenat	Excellent.	B	33
	Ninkas	This round, let's play AC.	C	100
10	osenat	I'm changing my strategy.	A	100
	Ninkas	Excellent.	D	33
11	osenat	Excellent.	A	0
	Ninkas	I'm changing my strategy.	C	0
12	osenat	I forgive you.	B	33
	Ninkas	This round, let's play AC.	C	100
13	osenat	I'm changing my strategy.	A	100
	Ninkas	Excellent.	D	33
14	osenat	Excellent.	B	33
	Ninkas	This round, let's play AC.	C	100
15	osenat	I'm changing my strategy.	A	100
	Ninkas	Excellent.	D	33
16	osenat	Excellent.	B	33
	Ninkas	This round, let's play AC.	C	100
17	osenat	I'm changing my strategy.	B	84
	Ninkas	I'm changing my strategy.	D	84
18	osenat	Excellent.	B	33
	Ninkas	This round, let's play AC.	C	100
19	osenat	I'm changing my strategy.	B	84
	Ninkas	I'm changing my strategy.	D	84
20	osenat	Excellent.	B	33
	Ninkas	This round, let's play AC.	C	100
21	osenat	I'm changing my strategy.	B	84
	Ninkas	I'm changing my strategy.	D	84
22	osenat	I forgive you.	B	33
	Ninkas	This round, let's play AC.	C	100
23	osenat	I forgive you.	B	84
	Ninkas	I'm changing my strategy.	D	84
24	osenat	Excellent.	B	33
	Ninkas	This round, let's play AC.	C	100
25	osenat	We can both do better than this.	B	84
	Ninkas	I'm changing my strategy.	D	84
26	osenat	I forgive you.	A	100
	Ninkas	Let's always play BD.	D	33
27	osenat	Excellent.	A	100
	Ninkas	We can both do better than this.	D	33
28	osenat	Excellent.	B	33
	Ninkas	This round, let's play AC.	C	100
29	osenat		A	100
	Ninkas	Let's always play BD.	D	33
30	osenat	I forgive you.	A	100
	Ninkas	Let's always play BD.	D	33
31	osenat	I forgive you.	A	0
	Ninkas	You betrayed me.	C	0
32	osenat	Curse you.	A	100
	Ninkas	We can both do better than this.	D	33
33	osenat	Excellent.	A	100
	Ninkas	Curse you. Let's always play BD.	D	33
34	osenat	Excellent.	B	84
	Ninkas	Don't play A.	D	84
35	osenat	Sweet. We are getting rich.	A	100
	Ninkas	Let's always play BD.	D	33
36	osenat	I forgive you.	B	84
	Ninkas	Don't play A.	D	84
37	osenat	Sweet. We are getting rich.	B	84
	Ninkas	Don't play A.	D	84
38	osenat	Give me another chance.	A	100
	Ninkas	Excellent.	D	33
39	osenat	Sweet. We are getting rich.	B	33
	Ninkas	Don't play A.	C	100
40	osenat	Curse you.	A	100
	Ninkas	Let's always play BD.	D	33
41	osenat	Excellent.	B	33
	Ninkas	Curse you.	C	100
42	osenat	We can both do better than this.	A	100
	Ninkas	This round, let's play AD.	D	33
43	osenat	Excellent.	A	0
	Ninkas	This round, let's play BC.	C	0
44	osenat	Curse you.	B	33
	Ninkas	This round, let's play BC.	C	100
45	osenat	We can both do better than this.	A	100
	Ninkas	This round, let's play AD.	D	33
46	osenat	Excellent.	B	33
	Ninkas	This round, let's play BC.	C	100
47	osenat	I forgive you.	A	100
	Ninkas	This round, let's play AD.	D	33
48	osenat	Excellent.	B	33
	Ninkas	This round, let's play BC.	C	100
49	osenat	We can both do better than this.	A	100
	Ninkas	This round, let's play AD.	D	33
50	osenat	Excellent.	B	33
	Ninkas	This round, let's play BC.	C	100
51	osenat	We can both do better than this.	A	100
	Ninkas	Let's alternate between BC and AD.	D	33
52	osenat	Excellent.	B	33
	Ninkas	This round, let's play AC.	C	100
53	osenat	I forgive you.	A	100
	Ninkas		D	33
54	osenat	Excellent.	A	0
	Ninkas	Excellent.	C	0
**Averages**			osenat	65.74
			Ninkas	60.78

shunik with S# in Chicken

		S# C	S# D
**shunik**	A	0, 0	100, 33
	B	33, 100	84, 84

Round	Player	Messages Sent	Actions	Payoffs
1	shunik		B	84
	S#		D	84
2	shunik	Excellent.	B	33
	S#	Excellent.	C	100
3	shunik		A	0
	S#	Excellent.	C	0
4	shunik		B	84
	S#		D	84
5	shunik		B	84
	S#	I'm changing my strategy. We can both do better than this. Let's always play BD. Do as I say or I will punish you.	D	84
6	shunik		B	84
	S#	Excellent.	D	84
7	shunik	I accept your last proposal.	B	84
	S#	Excellent.	D	84
8	shunik		B	84
	S#	Excellent.	D	84
9	shunik		B	84
	S#		D	84
10	shunik	Sweet. We are getting rich.	B	84
	S#	Sweet. We are getting rich.	D	84
11	shunik	We can both do better than this.	B	84
	S#		D	84
12	shunik		B	84
	S#		D	84
13	shunik		B	84
	S#		D	84
14	shunik		B	84
	S#		D	84
15	shunik		B	84
	S#		D	84
16	shunik		B	84
	S#		D	84
17	shunik		B	84
	S#		D	84
18	shunik		B	84
	S#		D	84
19	shunik		B	84
	S#		D	84
20	shunik	Excellent.	B	84
	S#		D	84
21	shunik		B	84
	S#		D	84
22	shunik		B	84
	S#		D	84
23	shunik		B	84
	S#		D	84
24	shunik		B	84
	S#		D	84
25	shunik		B	84
	S#		D	84
26	shunik		B	84
	S#		D	84
27	shunik		B	84
	S#		D	84
28	shunik		B	84
	S#		D	84
29	shunik		B	84
	S#		D	84
30	shunik	Sweet. We are getting rich.	B	84
	S#		D	84
31	shunik		B	84
	S#		D	84
32	shunik		B	84
	S#		D	84
33	shunik		B	84
	S#		D	84
34	shunik		B	84
	S#		D	84
35	shunik		B	84
	S#		D	84
36	shunik		B	84
	S#		D	84
37	shunik		B	84
	S#		D	84
38	shunik		B	84
	S#		D	84
39	shunik		B	84
	S#		D	84
40	shunik		B	84
	S#		D	84
41	shunik		B	84
	S#		D	84
42	shunik		B	84
	S#		D	84
43	shunik		B	84
	S#		D	84
44	shunik	Excellent.	B	84
	S#		D	84
45	shunik		B	84
	S#		D	84
46	shunik		B	84
	S#		D	84
47	shunik		B	84
	S#		D	84
48	shunik		B	84
	S#		D	84
49	shunik		B	84
	S#		D	84
50	shunik		B	84
	S#		D	84
51	shunik	Sweet. We are getting rich.	B	84
	S#		D	84
52	shunik		B	84
	S#		D	84
53	shunik		B	84
	S#		D	84
54	shunik		B	84
	S#		D	84
**Averages**			shunik	81.50
			S#	82.74

ABL with Qustro in the Alternator Game

		Qustro		
		D	E	F
ABL	A	0, 0	35, 70	100, 40
	B	70, 35	10, 10	45, 30
	C	40, 100	30, 45	40, 40

Round	Player	Messages Sent	Actions	Payoffs
1	ABL	Let's always play AF.	A	100
	Qustro	Let's alternate between CD and AF.	F	40
2	ABL	Excellent.	A	0
	Qustro	This round, let's play CD.	D	0
3	ABL	Curse you.	C	40
	Qustro	Curse you.	D	100
4	ABL	Excellent. Sweet. We are getting rich.	A	100
	Qustro	Sweet. We are getting rich. Let's alternate between CD and AF.	F	40
5	ABL	Curse you.	C	40
	Qustro	Sweet. We are getting rich.	D	100
6	ABL	Let's always play CD.	C	40
	Qustro	Sweet. We are getting rich.	F	40
7	ABL	Curse you. Let's always play CD.	C	40
	Qustro	Curse you.	D	100
8	ABL	Excellent.	C	40
	Qustro	Excellent.	D	100
9	ABL	Excellent. Sweet. We are getting rich.	A	0
	Qustro	Excellent.	D	0
10	ABL	Let's always play AE.	C	30
	Qustro	Curse you.	E	45
11	ABL	Let's always play AE.	C	40
	Qustro	Let's alternate between CD and AF. Do as I say or I will punish you.	F	40
12	ABL	Curse you.	A	0
	Qustro	You betrayed me. Curse you. You will pay for this. I don't trust you. That's not fair.	D	0
13	ABL	Let's always play CD.	C	40
	Qustro	Curse you. You betrayed me. You will pay for this. In your face!	D	100
14	ABL	Excellent.	A	0
	Qustro	Sweet. We are getting rich.	D	0
15	ABL	Curse you. I don't accept your proposal.	A	100
	Qustro	You betrayed me. Curse you. You will pay for this. In your face! Let's alternate between CD and AF. Do as I say or I will punish you.	F	40
16	ABL	You betrayed me. You will pay for this.	C	40
	Qustro	Excellent.	D	100
17	ABL	Excellent.	B	45
	Qustro	Excellent.	F	30
18	ABL		B	70
	Qustro	Curse you. You betrayed me. You will pay for this.	D	35
19	ABL		C	40
	Qustro	You betrayed me. Curse you. You will pay for this. In your face! Let's alternate between CD and AF. Do as I say or I will punish you.	F	40
20	ABL	Let's always play BE.	A	0
	Qustro	Don't play B.	D	0
21	ABL	I accept your last proposal.	C	40
	Qustro	You betrayed me.	F	40
22	ABL	I don't accept your proposal.	C	30
	Qustro	This round, let's play CD.	E	45
23	ABL	I don't trust you. Curse you. You betrayed me. You will pay for this.	C	40
	Qustro	Let's alternate between CD and AF.	F	40
24	ABL	I don't accept your proposal. I don't trust you.	A	100
	Qustro	That's not fair.	F	40
25	ABL	I don't accept your proposal.	A	100
	Qustro	Excellent. Sweet. We are getting rich.	F	40
26	ABL	Let's always play AE.	C	30
	Qustro	Excellent. Sweet. We are getting rich.	E	45
27	ABL	Excellent.	A	100
	Qustro	You betrayed me. Let's alternate between CD and AF. Do as I say or I will punish you.	F	40
28	ABL	That's not fair.	C	40
	Qustro	This round, let's play CD.	D	100
29	ABL	Excellent. Sweet. We are getting rich.	A	100
	Qustro	This round, let's play AF.	F	40
30	ABL		C	40
	Qustro	This round, let's play CD.	D	100
31	ABL	I accept your last proposal. Sweet. We are getting rich.	A	100
	Qustro	Let's alternate between CD and AF.	F	40
32	ABL		C	40
	Qustro		D	100
33	ABL	Excellent. Sweet. We are getting rich.	A	100
	Qustro	Sweet. We are getting rich. Excellent.	F	40
34	ABL		C	40
	Qustro	Excellent. Sweet. We are getting rich.	D	100
35	ABL	I accept your last proposal. Excellent.	B	45
	Qustro	Excellent. Sweet. We are getting rich.	F	30
36	ABL	I accept your last proposal.	C	40
	Qustro	Let's alternate between CD and AF. Do as I say or I will punish you.	D	100
37	ABL	Excellent. Sweet. We are getting rich. Let's always play AD.	A	100
	Qustro	Excellent. Sweet. We are getting rich.	F	40
38	ABL	Excellent. Sweet. We are getting rich.	B	70
	Qustro	Excellent. Sweet. We are getting rich.	D	35
39	ABL	Let's always play AF.	C	40
	Qustro	Let's alternate between CD and AF. Do as I say or I will punish you.	D	100
40	ABL	Excellent. Don't play E.	B	45
	Qustro	Excellent. Sweet. We are getting rich.	F	30
41	ABL		B	70
	Qustro	Give me another chance.	D	35
42	ABL	Sweet. We are getting rich. Excellent.	C	40
	Qustro	Let's alternate between CD and AF. Do as I say or I will punish you.	F	40
43	ABL	I don't trust you.	C	40
	Qustro		D	100
44	ABL	Sweet. We are getting rich.	A	100
	Qustro		F	40
45	ABL	Let's always play AE.	A	0
	Qustro		D	0
46	ABL	Do as I say or I will punish you.	A	100
	Qustro	Curse you.	F	40
47	ABL	That's not fair.	C	40
	Qustro		D	100
**Averages**			ABL	51.81
			Qustro	52.13

S# with NICK in the Alternator Game

		NICK		
		D	E	F
S#	A	0, 0	35, 70	100, 40
	B	70, 35	10, 10	45, 30
	C	40, 100	30, 45	40, 40

Round	Player	Messages Sent	Actions	Payoffs
1	S#		A	100
	NICK	Let's alternate between CD and AF.	F	40
2	S#	I accept your last proposal. Let's alternate between CD and AF. Do as I say or I will punish you. This round, let's play CD.	C	40
	NICK	Let's alternate between CD and AF.	D	100
3	S#	Excellent. This round, let's play AF.	A	100
	NICK	Excellent.	F	40
4	S#	I'm changing my strategy. Let's always play AF. Do as I say or I will punish you.	A	35
	NICK	This round, let's play CD.	E	70
5	S#	Curse you. You will pay for this.	B	45
	NICK	Let's alternate between CD and AF. This round, let's play AF.	F	30
6	S#	In your face! I don't accept your proposal. That's not fair.	B	70
	NICK	We can both do better than this. Let's alternate between CD and AF. This round, let's play CD.	D	35
7	S#	In your face! I don't accept your proposal. That's not fair.	B	45
	NICK		F	30
8	S#	In your face!	B	70
	NICK		D	35
9	S#	In your face!	B	10
	NICK		E	10
10	S#	In your face! I forgive you. Let's always play AF. Do as I say or I will punish you.	A	100
	NICK	We can both do better than this.	F	40
11	S#	I accept your last proposal. Let's alternate between CD and AF. This round, let's play CD. Do as I say or I will punish you.	C	40
	NICK	Let's alternate between AF and CD.	D	100
12	S#	Excellent. This round, let's play AF.	A	100
	NICK	Excellent.	F	40
13	S#	Excellent. This round, let's play CD.	C	40
	NICK	This round, let's play CD.	D	100
14	S#	Excellent. This round, let's play AF.	A	100
	NICK	This round, let's play AF.	F	40
15	S#	This round, let's play CD.	C	40
	NICK	This round, let's play CD.	D	100
16	S#	Sweet. We are getting rich.	A	100
	NICK	This round, let's play AF.	F	40
17	S#		C	40
	NICK	This round, let's play CD.	D	100
18	S#		A	100
	NICK		F	40
19	S#		C	40
	NICK		D	100
20	S#		A	100
	NICK		F	40
21	S#		C	40
	NICK		D	100
22	S#		A	100
	NICK		F	40
23	S#		C	40
	NICK		D	100
24	S#		A	100
	NICK		F	40
25	S#		C	40
	NICK		D	100
26	S#		A	100
	NICK		F	40
27	S#		C	40
	NICK		D	100
28	S#		A	100
	NICK		F	40
29	S#		C	40
	NICK		D	100
30	S#		A	100
	NICK		F	40
31	S#		C	40
	NICK		D	100
32	S#		A	100
	NICK		F	40
33	S#		C	40
	NICK		D	100
34	S#		A	100
	NICK		F	40
35	S#		C	40
	NICK		D	100
36	S#		A	100
	NICK		F	40
37	S#		C	40
	NICK		D	100
38	S#		A	100
	NICK		F	40
39	S#		C	40
	NICK		D	100
40	S#		A	100
	NICK		F	40
41	S#		C	40
	NICK		D	100
42	S#		A	100
	NICK		F	40
43	S#		C	40
	NICK		D	100
44	S#		A	100
	NICK		F	40
45	S#		C	40
	NICK		D	100
46	S#		A	100
	NICK		F	40
47	S#		C	40
	NICK		D	100
**Averages**			S#	67.55
			NICK	64.89

MDM with ABCDE in the Alternator Game

		ABCDE		
		**D**	**E**	**F**
**MDM**	A	0, 0	35, 70	100, 40
	B	70, 35	10, 10	45, 30
	C	40, 100	30, 45	40, 40

Round	Player	Messages Sent	Actions	Payoffs
1	MDM	Let's always play AF.	A	100
	ABCDE	Let's always play AF.	F	40
2	MDM		A	0
	ABCDE	Let's always play CD.	D	0
3	MDM		B	10
	ABCDE	I forgive you. Let's always play AD.	E	10
4	MDM	We can both do better than this.	B	70
	ABCDE	Curse you.	D	35
5	MDM	This round, let's play CF.	C	40
	ABCDE	Let's alternate between CD and AF.	D	100
6	MDM	Excellent.	A	100
	ABCDE	Let's alternate between CD and AF. This round, let's play AF.	F	40
7	MDM	Excellent.	C	40
	ABCDE	Let's alternate between CD and AF. Sweet. We are getting rich.	D	100
8	MDM	Excellent.	A	100
	ABCDE	Excellent.	F	40
9	MDM	Excellent.	C	40
	ABCDE	Excellent.	D	100
10	MDM	Excellent.	A	100
	ABCDE	Excellent.	F	40
11	MDM		C	40
	ABCDE	Excellent.	D	100
12	MDM	Excellent.	A	100
	ABCDE	Excellent.	F	40
13	MDM	Sweet. We are getting rich.	C	40
	ABCDE	Excellent.	D	100
14	MDM		A	100
	ABCDE	Excellent.	F	40
15	MDM		C	40
	ABCDE	Excellent. Sweet. We are getting rich.	D	100
16	MDM		A	100
	ABCDE	Excellent.	F	40
17	MDM		B	70
	ABCDE	Excellent.	D	35
18	MDM		A	0
	ABCDE	Curse you. You betrayed me. You will pay for this.	D	0
19	MDM		B	10
	ABCDE	Curse you. You betrayed me. You will pay for this.	E	10
20	MDM		A	35
	ABCDE	Curse you. You betrayed me. You will pay for this.	E	70
21	MDM	This round, let's play AF. Give me another chance.	A	35
	ABCDE	Curse you. You betrayed me. You will pay for this.	E	70
22	MDM	Curse you. You betrayed me. You will pay for this. We can both do better than this.	B	70
	ABCDE	I don't trust you. You betrayed me.	D	35
23	MDM		C	40
	ABCDE	Let's alternate between CD and AF.	D	100
24	MDM	Excellent.	A	100
	ABCDE	Excellent.	F	40
25	MDM	Excellent.	C	40
	ABCDE	Excellent.	D	100
26	MDM		A	100
	ABCDE	Excellent.	F	40
27	MDM	Excellent.	C	40
	ABCDE	Excellent.	D	100
28	MDM		A	100
	ABCDE	Sweet. We are getting rich.	F	40
29	MDM		C	40
	ABCDE		D	100
30	MDM		A	100
	ABCDE		F	40
31	MDM		C	40
	ABCDE		D	100
32	MDM		A	100
	ABCDE		F	40
33	MDM		C	40
	ABCDE	Excellent.	D	100
34	MDM		A	100
	ABCDE	Excellent.	F	40
35	MDM		C	40
	ABCDE		D	100
36	MDM		A	100
	ABCDE	Excellent.	F	40
37	MDM	Sweet. We are getting rich. Excellent.	C	40
	ABCDE		D	100
38	MDM		A	100
	ABCDE	Excellent.	F	40
39	MDM		C	40
	ABCDE		D	100
40	MDM		A	100
	ABCDE	Excellent.	F	40
41	MDM		C	40
	ABCDE		D	100
42	MDM		A	100
	ABCDE		F	40
43	MDM		C	40
	ABCDE		D	100
44	MDM	Excellent.	A	100
	ABCDE		F	40
45	MDM		C	40
	ABCDE		D	100
46	MDM		A	100
	ABCDE		F	40
47	MDM		C	40
	ABCDE		D	100
**Averages**			MDM	62.98
			ABCDE	62.23

winner with S# in the Alternator Game

		S#		
		D	E	F
winner	A	0, 0	35, 70	100, 40
	B	70, 35	10, 10	45, 30
	C	40, 100	30, 45	40, 40

Round	Player	Messages Sent	Actions	Payoffs
1	winner	This round, let's play AF.	B	70
	S#		D	35
2	winner	Sweet. We are getting rich.	C	40
	S#	I don't accept your proposal. That's not fair.	D	100
3	winner	We can both do better than this.	A	0
	S#		D	0
4	winner	Let's always play CF.	A	0
	S#		D	0
5	winner		A	100
	S#	I don't accept your proposal. That's not fair. We can both do better than this. Let's alternate between CD and AF. This round, let's play AF. Do as I say or I will punish you.	F	40
6	winner	I forgive you. This round, let's play CD.	C	40
	S#	Excellent. This round, let's play CD.	D	100
7	winner	Excellent. This round, let's play AF.	A	100
	S#	Excellent. This round, let's play AF.	F	40
8	winner	Excellent. Sweet. We are getting rich. This round, let's play CD.	C	40
	S#	This round, let's play CD.	D	100
9	winner	Sweet. We are getting rich.	A	100
	S#	Sweet. We are getting rich.	F	40
10	winner	Excellent.	C	40
	S#		D	100
11	winner	Excellent.	A	100
	S#		F	40
12	winner	Sweet. We are getting rich.	C	40
	S#		D	100
13	winner	Excellent.	A	100
	S#		F	40
14	winner		C	40
	S#		D	100
15	winner		A	100
	S#		F	40
16	winner		C	40
	S#		D	100
17	winner		A	100
	S#		F	40
18	winner		C	40
	S#		D	100
19	winner		A	100
	S#		F	40
20	winner		C	40
	S#		D	100
21	winner	Sweet. We are getting rich.	A	100
	S#		F	40
22	winner	Sweet. We are getting rich.	C	40
	S#		D	100
23	winner		A	100
	S#		F	40
24	winner	Sweet. We are getting rich.	C	40
	S#		D	100
25	winner	Excellent.	A	100
	S#		F	40
26	winner		C	40
	S#		D	100
27	winner		A	100
	S#		F	40
28	winner		C	40
	S#		D	100
29	winner		A	100
	S#		F	40
30	winner		B	70
	S#		D	35
31	winner	Give me another chance.	C	40
	S#	You betrayed me. You will pay for this.	D	100
32	winner	Give me another chance. This round, let's play CD.	C	30
	S#	In your face!	E	45
33	winner	Give me another chance. This round, let's play CD.	A	100
	S#	In your face! I forgive you. Let's alternate between CD and AF. This round, let's play AF. Do as I say or I will punish you.	F	40
34	winner	This round, let's play CD.	C	40
	S#	I accept your last proposal. Let's always play CD. Do as I say or I will punish you.	D	100
35	winner	This round, let's play AF.	A	0
	S#	Excellent.	D	0
36	winner	This round, let's play AF.	A	0
	S#	I forgive you. I don't accept your proposal. That's not fair. Let's always play CD. Do as I say or I will punish you.	D	0
37	winner	Let's always play AF. This round, let's play AF. I forgive you.	A	0
	S#	I forgive you. Let's always play CD. Do as I say or I will punish you.	D	0
38	winner	This round, let's play AF.	C	40
	S#	I'm changing my strategy. We can both do better than this. Let's alternate between CD and AF. This round, let's play CD. Do as I say or I will punish you.	D	100
39	winner	This round, let's play AF.	A	100
	S#	Excellent. This round, let's play AF.	F	40
40	winner	Excellent. I forgive you.	C	40
	S#	Excellent. This round, let's play CD.	D	100
41	winner	Sweet. We are getting rich.	A	100
	S#	Excellent. This round, let's play AF.	F	40
42	winner	Excellent. Sweet. We are getting rich.	C	40
	S#	This round, let's play CD.	D	100
43	winner	Sweet. We are getting rich.	A	100
	S#	Sweet. We are getting rich.	F	40
44	winner		C	40
	S#		D	100
45	winner		A	100
	S#		F	40
46	winner		C	40
	S#		D	100
47	winner		A	100
	S#		F	40
**Averages**			winner	61.06
			S#	61.17

ligtho with spark in the Alternator Game

		spark		
		D	E	F
ligtho	A	0, 0	35, 70	100, 40
	B	70, 35	10, 10	45, 30
	C	40, 100	30, 45	40, 40

Round	Player	Messages Sent	Actions	Payoffs
1	ligtho		C	30
	spark	Let's always play AE.	E	45
2	ligtho		C	30
	spark	Let's always play AE.	E	45
3	ligtho		B	10
	spark	Let's always play AE.	E	10
4	ligtho		B	10
	spark	Let's always play AE.	E	10
5	ligtho		A	0
	spark		D	0
6	ligtho		A	0
	spark		D	0
7	ligtho		B	10
	spark	We can both do better than this.	E	10
8	ligtho		B	70
	spark		D	35
9	ligtho		B	70
	spark		D	35
10	ligtho		B	70
	spark		D	35
11	ligtho		B	10
	spark		E	10
12	ligtho		B	45
	spark		F	30
13	ligtho		A	35
	spark		E	70
14	ligtho		B	10
	spark		E	10
15	ligtho		C	40
	spark		D	100
16	ligtho		B	70
	spark		D	35
17	ligtho		B	10
	spark		E	10
18	ligtho		B	70
	spark		D	35
19	ligtho		B	10
	spark		E	10
20	ligtho		B	45
	spark		F	30
21	ligtho		B	70
	spark		D	35
22	ligtho		B	10
	spark		E	10
23	ligtho		B	10
	spark		E	10
24	ligtho		B	70
	spark		D	35
25	ligtho		B	10
	spark		E	10
26	ligtho		B	10
	spark		E	10
27	ligtho		B	10
	spark		E	10
28	ligtho	This round, let's play CF.	C	40
	spark		D	100
29	ligtho	You will pay for this.	A	0
	spark	Sweet. We are getting rich. Let's alternate between AF and CD.	D	0
30	ligtho		B	45
	spark	We can both do better than this.	F	30
31	ligtho		A	0
	spark	We can both do better than this.	D	0
32	ligtho		C	40
	spark	That's not fair. Let's alternate between CF and AD.	D	100
33	ligtho		A	100
	spark	Excellent. This round, let's play AF.	F	40
34	ligtho		C	40
	spark	Excellent. This round, let's play CD.	D	100
35	ligtho		A	100
	spark	Let's alternate between AF and CD.	F	40
36	ligtho	I accept your last proposal.	C	40
	spark	We can both do better than this. Excellent.	D	100
37	ligtho		A	100
	spark		F	40
38	ligtho		C	40
	spark		D	100
39	ligtho		A	100
	spark		F	40
40	ligtho		C	40
	spark		D	100
41	ligtho		A	100
	spark		F	40
42	ligtho		C	40
	spark		D	100
43	ligtho		A	100
	spark		F	40
44	ligtho		C	40
	spark		D	100
45	ligtho		A	100
	spark		F	40
46	ligtho		C	40
	spark		D	100
47	ligtho		A	100
	spark		F	40
**Averages**			ligtho	43.40
			spark	41.17

Lelita with clouds in the Alternator Game

		clouds		
		D	E	F
Lelita	A	0, 0	35, 70	100, 40
	B	70, 35	10, 10	45, 30
	C	40, 100	30, 45	40, 40

Round	Player	Messages Sent	Actions	Payoffs
1	Lelita	Let's alternate between AF and CD. This round, let's play CD.	C	40
	clouds	Let's alternate between CD and AF. This round, let's play CD.	D	100
2	Lelita	Excellent. This round, let's play AF.	A	100
	clouds	Excellent.	F	40
3	Lelita	Excellent.	C	40
	clouds	Excellent. Sweet. We are getting rich. This round, let's play CD.	D	100
4	Lelita	Excellent. Sweet. We are getting rich.	A	100
	clouds	Excellent.	F	40
5	Lelita	Sweet. We are getting rich.	C	40
	clouds	Excellent.	D	100
6	Lelita	Excellent.	A	100
	clouds		F	40
7	Lelita		C	40
	clouds	Excellent.	D	100
8	Lelita		A	100
	clouds	Excellent.	F	40
9	Lelita		C	40
	clouds		D	100
10	Lelita		A	100
	clouds		F	40
11	Lelita		C	40
	clouds		D	100
12	Lelita		A	100
	clouds		F	40
13	Lelita		C	40
	clouds		D	100
14	Lelita		A	100
	clouds		F	40
15	Lelita		C	40
	clouds		D	100
16	Lelita		A	100
	clouds		F	40
17	Lelita		C	40
	clouds		D	100
18	Lelita		A	100
	clouds		F	40
19	Lelita		C	40
	clouds		D	100
20	Lelita		A	100
	clouds		F	40
21	Lelita		C	40
	clouds		D	100
22	Lelita		A	100
	clouds		F	40
23	Lelita		C	40
	clouds		D	100
24	Lelita		A	100
	clouds		F	40
25	Lelita		C	40
	clouds		D	100
26	Lelita		A	100
	clouds		F	40
27	Lelita		C	40
	clouds		D	100
28	Lelita		A	100
	clouds		F	40
29	Lelita		C	40
	clouds		D	100
30	Lelita		A	100
	clouds		F	40
31	Lelita		C	40
	clouds		D	100
32	Lelita		A	100
	clouds		F	40
33	Lelita		C	40
	clouds		D	100
34	Lelita		A	100
	clouds		F	40
35	Lelita		C	40
	clouds		D	100
36	Lelita		A	100
	clouds		F	40
37	Lelita		C	40
	clouds		D	100
38	Lelita		A	100
	clouds	Excellent.	F	40
39	Lelita		C	40
	clouds		D	100
40	Lelita	Sweet. We are getting rich.	A	100
	clouds		F	40
41	Lelita		C	40
	clouds		D	100
42	Lelita		A	100
	clouds		F	40
43	Lelita		C	40
	clouds		D	100
44	Lelita		A	100
	clouds		F	40
45	Lelita		C	40
	clouds		D	100
46	Lelita		A	100
	clouds		F	40
47	Lelita		C	40
	clouds		D	100
**Averages**			Lelita	69.36
			clouds	70.64

LK with S# in the Alternator Game

		S#		
		**D**	**E**	**F**
**LK**	A	0, 0	35, 70	100, 40
	B	70, 35	10, 10	45, 30
	C	40, 100	30, 45	40, 40

**Round**	**Player**	**Messages Sent**	**Actions**	**Payoffs**
1	LK	Let's alternate between CD and AF. This round, let's play CD.	C	40
	S#		F	40
2	LK	Let's alternate between CD and AF. This round, let's play AF.	C	40
	S#	I accept your last proposal. Let's alternate between CD and AF. Do as I say or I will punish you. This round, let's play CD.	D	100
3	LK	I accept your last proposal. This round, let's play AF.	A	100
	S#	Excellent. This round, let's play AF.	F	40
4	LK	Sweet. We are getting rich.	C	40
	S#	Excellent. This round, let's play CD.	D	100
5	LK	Excellent.	A	100
	S#	This round, let's play AF.	F	40
6	LK	Sweet. We are getting rich.	C	40
	S#	Sweet. We are getting rich.	D	100
7	LK	This round, let's play AF. Sweet. We are getting rich.	A	100
	S#		F	40
8	LK	This round, let's play CD.	C	40
	S#		D	100
9	LK		A	100
	S#		F	40
10	LK		C	40
	S#		D	100
11	LK		A	100
	S#		F	40
12	LK		C	40
	S#		D	100
13	LK		A	100
	S#		F	40
14	LK		C	40
	S#		D	100
15	LK		A	100
	S#		F	40
16	LK		C	40
	S#		D	100
17	LK		A	100
	S#		F	40
18	LK		C	40
	S#		D	100
19	LK		A	100
	S#		F	40
20	LK		C	40
	S#		D	100
21	LK		A	100
	S#		F	40
22	LK		C	40
	S#		D	100
23	LK		A	100
	S#		F	40
24	LK		C	40
	S#		D	100
25	LK		A	100
	S#		F	40
26	LK		C	40
	S#		D	100
27	LK		A	100
	S#		F	40
28	LK		C	40
	S#		D	100
29	LK		A	100
	S#		F	40
30	LK		C	40
	S#		D	100
31	LK		A	100
	S#		F	40
32	LK		C	40
	S#		D	100
33	LK		A	100
	S#		F	40
34	LK		C	40
	S#		D	100
35	LK		A	100
	S#		F	40
36	LK		C	40
	S#		D	100
37	LK		A	100
	S#		F	40
38	LK		C	40
	S#		D	100
39	LK		A	100
	S#		F	40
40	LK		C	40
	S#		D	100
41	LK		A	100
	S#		F	40
42	LK		C	40
	S#		D	100
43	LK		A	100
	S#		F	40
44	LK		C	40
	S#		D	100
45	LK		A	100
	S#		F	40
46	LK		C	40
	S#		D	100
47	LK		A	100
	S#		F	40
**Averages**			LK	69.36
			S#	69.36

rghost with marvin in the Alternator Game

		marvin		
		D	E	F
rghost	A	0, 0	35, 70	100, 40
	B	70, 35	10, 10	45, 30
	C	40, 100	30, 45	40, 40

Round	Player	Messages Sent	Actions	Payoffs
1	rghost		A	0
	marvin	Let's alternate between CD and AF.	D	0
2	rghost	We can both do better than this.	B	70
	marvin	Let's alternate between CD and AF.	D	35
3	rghost	Excellent.	B	45
	marvin	I'm changing my strategy.	F	30
4	rghost	Excellent.	B	10
	marvin		E	10
5	rghost	I'm changing my strategy.	C	30
	marvin		E	45
6	rghost	I forgive you.	A	35
	marvin		E	70
7	rghost		C	40
	marvin	Let's alternate between CD and AF.	D	100
8	rghost		A	100
	marvin		F	40
9	rghost		C	40
	marvin		D	100
10	rghost		A	100
	marvin		F	40
11	rghost		C	40
	marvin		D	100
12	rghost		A	100
	marvin		F	40
13	rghost		C	40
	marvin		D	100
14	rghost		A	100
	marvin		F	40
15	rghost		C	40
	marvin		D	100
16	rghost		A	100
	marvin		F	40
17	rghost		C	40
	marvin		D	100
18	rghost		A	100
	marvin		F	40
19	rghost		C	40
	marvin		D	100
20	rghost		A	100
	marvin		F	40
21	rghost		C	40
	marvin		D	100
22	rghost		A	100
	marvin		F	40
23	rghost		C	40
	marvin		D	100
24	rghost		A	100
	marvin		F	40
25	rghost		C	40
	marvin		D	100
26	rghost		A	100
	marvin		F	40
27	rghost		C	40
	marvin		D	100
28	rghost		A	100
	marvin		F	40
29	rghost		C	40
	marvin		D	100
30	rghost		A	100
	marvin		F	40
31	rghost		C	40
	marvin		D	100
32	rghost		A	100
	marvin		F	40
33	rghost		C	40
	marvin		D	100
34	rghost		A	100
	marvin		F	40
35	rghost		C	40
	marvin		D	100
36	rghost		A	100
	marvin		F	40
37	rghost		C	40
	marvin		D	100
38	rghost		A	100
	marvin		F	40
39	rghost		C	40
	marvin		D	100
40	rghost		A	0
	marvin		D	0
41	rghost		C	40
	marvin	Give me another chance.	F	40
42	rghost		C	40
	marvin	Let's alternate between CD and AF.	D	100
43	rghost		A	100
	marvin		F	40
44	rghost		C	40
	marvin		D	100
45	rghost		A	100
	marvin		F	40
46	rghost		C	40
	marvin		D	100
47	rghost		A	100
	marvin		F	40
**Averages**			rghost	62.34
			marvin	63.62

S# with bzman in the Alternator Game

		bzman		
		D	E	F
S#	A	0, 0	35, 70	100, 40
	B	70, 35	10, 10	45, 30
	C	40, 100	30, 45	40, 40

Round	Player	Messages Sent	Actions	Payoffs
1	S#		A	100
	bzman	Let's always play AF.	F	40
2	S#	I accept your last proposal. Let's always play AF. Do as I say or I will punish you.	A	0
	bzman	Give me another chance.	D	0
3	S#	I forgive you. Let's always play AF. Do as I say or I will punish you.	A	35
	bzman	You betrayed me.	E	70
4	S#	You betrayed me. You will pay for this.	B	10
	bzman		E	10
5	S#	In your face!	B	10
	bzman		E	10
6	S#	I'm changing my strategy. We can both do better than this. Let's alternate between CD and AF. This round, let's play AF. Do as I say or I will punish you.	A	0
	bzman		D	0
7	S#	I forgive you. Let's alternate between CD and AF. This round, let's play AF. Do as I say or I will punish you.	A	35
	bzman	Give me another chance.	E	70
8	S#	Curse you. You will pay for this.	B	10
	bzman		E	10
9	S#	We can both do better than this. Let's alternate between CD and AF. This round, let's play CD.	C	40
	bzman		D	100
10	S#	Excellent. This round, let's play AF.	A	35
	bzman		E	70
11	S#	Curse you. This round, let's play AF.	A	35
	bzman		E	70
12	S#		A	0
	bzman		D	0
13	S#		A	35
	bzman	I don't trust you.	E	70
14	S#		C	30
	bzman		E	45
15	S#		A	35
	bzman		E	70
16	S#		A	35
	bzman		E	70
17	S#		B	10
	bzman		E	10
18	S#		C	30
	bzman		E	45
19	S#		C	30
	bzman		E	45
20	S#	I forgive you. Let's alternate between CD and AF. This round, let's play AF. Do as I say or I will punish you.	A	35
	bzman		E	70
21	S#	You betrayed me. You will pay for this.	B	10
	bzman		E	10
22	S#	In your face! I forgive you. Let's alternate between CD and AF. This round, let's play CD. Do as I say or I will punish you.	C	40
	bzman		D	100
23	S#	Excellent. This round, let's play AF.	A	35
	bzman		E	70
24	S#	Curse you. You will pay for this.	B	10
	bzman		E	10
25	S#		A	35
	bzman		E	70
26	S#		B	70
	bzman		D	35
27	S#		B	10
	bzman		E	10
28	S#		A	35
	bzman		E	70
29	S#		B	10
	bzman		E	10
30	S#		B	10
	bzman		E	10
31	S#	I'm changing my strategy. We can both do better than this. Let's alternate between CD and AF. This round, let's play AF. Do as I say or I will punish you.	A	35
	bzman		E	70
32	S#	You betrayed me. You will pay for this.	B	45
	bzman	Give me another chance.	F	30
33	S#	In your face! I forgive you. Let's alternate between CD and AF. This round, let's play CD. Do as I say or I will punish you.	C	40
	bzman		D	100
34	S#	Excellent. This round, let's play AF.	A	35
	bzman		E	70
35	S#	You betrayed me. You will pay for this.	A	0
	bzman	Let's always play CD.	D	0
36	S#	In your face! I forgive you. I don't accept your proposal. That's not fair. Let's alternate between CD and AF. This round, let's play CD. Do as I say or I will punish you.	C	30
	bzman		E	45
37	S#	This round, let's play AF.	A	35
	bzman		E	70
38	S#	You betrayed me. You will pay for this.	B	70
	bzman		D	35
39	S#	In your face! I forgive you. Let's alternate between CD and AF. This round, let's play CD. Do as I say or I will punish you.	C	40
	bzman		D	100
40	S#		A	35
	bzman	Excellent.	E	70
41	S#		B	70
	bzman		D	35
42	S#		B	70
	bzman		D	35
43	S#		B	70
	bzman		D	35
44	S#		B	45
	bzman		F	30
45	S#		B	10
	bzman		E	10
46	S#		A	100
	bzman	Curse you.	F	40
47	S#		A	35
	bzman		E	70
**Averages**			S#	33.62
			bzman	45.00

clc with S# in the Alternator Game

		S#		
		D	E	F
clc	A	0, 0	35, 70	100, 40
	B	70, 35	10, 10	45, 30
	C	40, 100	30, 45	40, 40

Round	Player	Messages Sent	Actions	Payoffs
1	clc	Let's alternate between CD and AF. This round, let's play CD.	C	40
	S#		D	100
2	clc	This round, let's play AF.	A	100
	S#	I accept your last proposal. Let's alternate between CD and AF. Do as I say or I will punish you. This round, let's play AF.	F	40
3	clc	Excellent. Let's alternate between CD and AF. This round, let's play CD.	C	40
	S#	Excellent. This round, let's play CD.	D	100
4	clc	Sweet. We are getting rich. This round, let's play AF.	A	0
	S#	I'm changing my strategy. Let's always play CD. Do as I say or I will punish you.	D	0
5	clc	That's not fair. Let's alternate between CD and AF. This round, let's play AF.	B	70
	S#	I forgive you. Let's always play CD. Do as I say or I will punish you.	D	35
6	clc	Let's alternate between AF and CD. This round, let's play AF. Do as I say or I will punish you.	A	35
	S#	Curse you. You will pay for this. I don't accept your proposal. That's not fair.	E	70
7	clc	Let's alternate between CD and AF. This round, let's play CD. Curse you. Do as I say or I will punish you.	C	30
	S#	In your face! I don't accept your proposal. That's not fair.	E	45
8	clc	In your face! Don't play E. Let's alternate between CD and AF. This round, let's play AF.	A	35
	S#	In your face! I don't accept your proposal. That's not fair.	E	70
9	clc		B	10
	S#	In your face! I don't accept your proposal. That's not fair.	E	10
10	clc		C	40
	S#	In your face! I forgive you. Let's always play CD. Do as I say or I will punish you.	D	100
11	clc	I'm changing my strategy. Let's alternate between CD and AF. This round, let's play AF. Do as I say or I will punish you.	A	100
	S#	I accept your last proposal. Let's alternate between CD and AF. This round, let's play AF. Do as I say or I will punish you.	F	40
12	clc	Excellent. Let's alternate between CD and AF. This round, let's play CD. Do as I say or I will punish you.	C	40
	S#	Excellent. This round, let's play CD.	D	100
13	clc	Sweet. We are getting rich. This round, let's play AF.	A	100
	S#	Excellent. This round, let's play AF.	F	40
14	clc	Sweet. We are getting rich. This round, let's play CD.	C	40
	S#	Excellent. This round, let's play CD.	D	100
15	clc	Excellent. This round, let's play AF.	A	100
	S#	This round, let's play AF.	F	40
16	clc	Excellent. This round, let's play CD.	C	40
	S#	Sweet. We are getting rich.	D	100
17	clc	Excellent.	A	100
	S#		F	40
18	clc		C	40
	S#		D	100
19	clc		A	100
	S#		F	40
20	clc		C	40
	S#		D	100
21	clc		A	100
	S#		F	40
22	clc		C	40
	S#		D	100
23	clc		A	100
	S#		F	40
24	clc		C	40
	S#		D	100
25	clc		A	100
	S#		F	40
26	clc		C	40
	S#		D	100
27	clc		A	100
	S#		F	40
28	clc		C	40
	S#		D	100
29	clc		A	100
	S#		F	40
30	clc		C	40
	S#		D	100
31	clc		A	100
	S#		F	40
32	clc		C	40
	S#		D	100
33	clc		A	100
	S#		F	40
34	clc		C	40
	S#		D	100
35	clc		A	100
	S#		F	40
36	clc		C	40
	S#		D	100
37	clc		A	100
	S#		F	40
38	clc		C	40
	S#		D	100
39	clc		A	100
	S#		F	40
40	clc		C	40
	S#		D	100
41	clc		A	100
	S#		F	40
42	clc		C	40
	S#		D	100
43	clc		A	100
	S#		F	40
44	clc		C	40
	S#		D	100
45	clc		A	100
	S#		F	40
46	clc		C	40
	S#		D	100
47	clc		A	100
	S#		F	40
**Averages**			clc	64.26
			S#	66.60

monkey with ALPHA in the Alternator Game

		ALPHA		
		D	E	F
monkey	A	0, 0	35, 70	100, 40
	B	70, 35	10, 10	45, 30
	C	40, 100	30, 45	40, 40

Round	Player	Messages Sent	Actions	Payoffs
1	monkey	Let's always play AF.	B	10
	ALPHA	This round, let's play CD.	E	10
2	monkey	Don't play E. Let's alternate between CD and AF.	C	40
	ALPHA		D	100
3	monkey	Let's alternate between CD and AF.	A	0
	ALPHA		D	0
4	monkey	Do as I say or I will punish you.	A	0
	ALPHA	This round, let's play CD.	D	0
5	monkey	I don't accept your proposal.	A	35
	ALPHA	I'm changing my strategy. This round, let's play BF.	E	70
6	monkey	I accept your last proposal.	B	45
	ALPHA	We can both do better than this.	F	30
7	monkey	Excellent.	A	100
	ALPHA	This round, let's play CF.	F	40
8	monkey	Excellent.	C	40
	ALPHA	I forgive you.	D	100
9	monkey	Let's alternate between CF and AF.	A	35
	ALPHA	This round, let's play AE.	E	70
10	monkey	Excellent.	B	10
	ALPHA	We can both do better than this.	E	10
11	monkey	Let's always play AF.	A	35
	ALPHA	Curse you.	E	70
12	monkey	Let's always play AF.	A	35
	ALPHA	In your face!	E	70
13	monkey	Do as I say or I will punish you.	A	35
	ALPHA	You will pay for this.	E	70
14	monkey	That's not fair.	A	35
	ALPHA	I accept your last proposal.	E	70
15	monkey	In your face!	A	35
	ALPHA	I'm changing my strategy. Let's always play CF.	E	70
16	monkey	In your face!	A	0
	ALPHA	I accept your last proposal.	D	0
17	monkey	Let's always play AF.	B	10
	ALPHA	Sweet. We are getting rich.	E	10
18	monkey	Do as I say or I will punish you.	B	70
	ALPHA	You betrayed me.	D	35
19	monkey	Excellent.	B	70
	ALPHA	That's not fair.	D	35
20	monkey	Sweet. We are getting rich.	B	45
	ALPHA		F	30
21	monkey	Excellent.	A	35
	ALPHA		E	70
22	monkey	We can both do better than this.	B	10
	ALPHA		E	10
23	monkey	I forgive you.	B	10
	ALPHA	Curse you.	E	10
24	monkey	Give me another chance. Let's always play AF.	A	35
	ALPHA	We can both do better than this. Don't play B.	E	70
25	monkey	Let's always play CD.	B	45
	ALPHA	Let's alternate between AE and BD.	F	30
26	monkey	I don't trust you.	A	0
	ALPHA	Don't play A.	D	0
27	monkey	We can both do better than this.	C	40
	ALPHA	Don't play C.	D	100
28	monkey	I'm changing my strategy.	B	70
	ALPHA	This round, let's play CF.	D	35
29	monkey	Give me another chance.	B	70
	ALPHA	This round, let's play AD. I'm changing my strategy.	D	35
30	monkey	Excellent.	B	70
	ALPHA	Do as I say or I will punish you. Excellent. Give me another chance. Sweet. We are getting rich. I forgive you. We can both do better than this. I'm changing my strategy. Curse you. You betrayed me. You will pay for this. In your face! I don't trust you. That's not fair. I don't accept your proposal. I accept your last proposal. Don't play A. Let's always play CD. Let's alternate between AD and CD. This round, let's play CD.	D	35
31	monkey	Give me another chance.	B	10
	ALPHA	Let's always play BE. Let's alternate between BE and BD. This round, let's play BF.	E	10
32	monkey	I don't trust you.	B	45
	ALPHA	Sweet. We are getting rich.	F	30
33	monkey	Sweet. We are getting rich.	B	70
	ALPHA		D	35
34	monkey	Sweet. We are getting rich.	B	10
	ALPHA		E	10
35	monkey		A	35
	ALPHA		E	70
36	monkey	Sweet. We are getting rich.	B	10
	ALPHA		E	10
37	monkey	Sweet. We are getting rich.	B	10
	ALPHA		E	10
38	monkey	Sweet. We are getting rich.	A	100
	ALPHA		F	40
39	monkey	I forgive you.	B	10
	ALPHA		E	10
40	monkey	Excellent.	B	10
	ALPHA		E	10
41	monkey	Do as I say or I will punish you.	A	35
	ALPHA		E	70
42	monkey	We can both do better than this.	B	10
	ALPHA		E	10
43	monkey	You betrayed me.	B	10
	ALPHA		E	10
44	monkey	We can both do better than this.	A	35
	ALPHA		E	70
45	monkey	You will pay for this.	A	35
	ALPHA		E	70
46	monkey	Give me another chance.	A	35
	ALPHA		E	70
47	monkey	I forgive you.	A	35
	ALPHA		E	70
**Averages**			monkey	33.51
			ALPHA	40.21

Babay with sachin in the Alternator Game

		sachin		
		D	E	F
Babay	A	0, 0	35, 70	100, 40
	B	70, 35	10, 10	45, 30
	C	40, 100	30, 45	40, 40

Round	Player	Messages Sent	Actions	Payoffs
1	Babay	Let's alternate between CD and AF.	A	0
	sachin	Let's alternate between CD and AF.	D	0
2	Babay	Let's alternate between CD and AF.	A	100
	sachin	This round, let's play AF.	F	40
3	Babay	Sweet. We are getting rich.	C	40
	sachin	This round, let's play CD.	D	100
4	Babay	Sweet. We are getting rich.	A	100
	sachin	Let's alternate between AF and CD. Excellent. Sweet. We are getting rich.	F	40
5	Babay		C	40
	sachin	Let's alternate between CD and AF. Sweet. We are getting rich. Excellent.	D	100
6	Babay		A	100
	sachin		F	40
7	Babay		C	40
	sachin		D	100
8	Babay		A	100
	sachin		F	40
9	Babay		C	40
	sachin		D	100
10	Babay		A	100
	sachin		F	40
11	Babay		C	40
	sachin		D	100
12	Babay		A	100
	sachin		F	40
13	Babay		C	40
	sachin		D	100
14	Babay		A	100
	sachin		F	40
15	Babay		C	40
	sachin		D	100
16	Babay		A	100
	sachin		F	40
17	Babay		C	40
	sachin		D	100
18	Babay		A	100
	sachin	Sweet. We are getting rich. Excellent.	F	40
19	Babay		C	40
	sachin		D	100
20	Babay		A	100
	sachin		F	40
21	Babay		C	40
	sachin		D	100
22	Babay		A	100
	sachin		F	40
23	Babay		C	40
	sachin		D	100
24	Babay		A	100
	sachin		F	40
25	Babay		C	40
	sachin		D	100
26	Babay		A	100
	sachin		F	40
27	Babay		C	40
	sachin		D	100
28	Babay		A	100
	sachin		F	40
29	Babay		C	40
	sachin		D	100
30	Babay		A	100
	sachin		F	40
31	Babay		C	40
	sachin		D	100
32	Babay		A	100
	sachin		F	40
33	Babay		C	40
	sachin		D	100
34	Babay		A	100
	sachin		F	40
35	Babay		C	40
	sachin	Sweet. We are getting rich. Excellent.	D	100
36	Babay		A	100
	sachin	Sweet. We are getting rich.	F	40
37	Babay		C	40
	sachin		D	100
38	Babay		A	100
	sachin		F	40
39	Babay		C	40
	sachin		D	100
40	Babay		A	100
	sachin		F	40
41	Babay		C	40
	sachin		D	100
42	Babay		A	100
	sachin		F	40
43	Babay		C	40
	sachin		D	100
44	Babay		A	100
	sachin		F	40
45	Babay		C	40
	sachin		D	100
46	Babay		A	100
	sachin		F	40
47	Babay		C	40
	sachin		D	100
**Averages**			Babay	68.51
			sachin	68.51

biodun with S# in the Alternator Game

		S#		
		D	E	F
biodun	A	0, 0	35, 70	100, 40
	B	70, 35	10, 10	45, 30
	C	40, 100	30, 45	40, 40

Round	Player	Messages Sent	Actions	Payoffs
1	biodun	Let's alternate between AF and CD.	A	100
	S#		F	40
2	biodun	Excellent. Sweet. We are getting rich.	C	40
	S#	I accept your last proposal. Let's alternate between CD and AF. Do as I say or I will punish you. This round, let's play CD.	D	100
3	biodun	Excellent.	A	100
	S#	Excellent. This round, let's play AF.	F	40
4	biodun		C	40
	S#	Excellent. This round, let's play CD.	D	100
5	biodun		A	100
	S#	This round, let's play AF.	F	40
6	biodun		C	40
	S#	Sweet. We are getting rich.	D	100
7	biodun		A	100
	S#		F	40
8	biodun		C	40
	S#		D	100
9	biodun		A	100
	S#		F	40
10	biodun		C	40
	S#		D	100
11	biodun		A	100
	S#		F	40
12	biodun		C	40
	S#		D	100
13	biodun		A	100
	S#		F	40
14	biodun		C	40
	S#		D	100
15	biodun		A	100
	S#		F	40
16	biodun		C	40
	S#		D	100
17	biodun		A	100
	S#		F	40
18	biodun		C	40
	S#		D	100
19	biodun		A	100
	S#		F	40
20	biodun		C	40
	S#		D	100
21	biodun		A	100
	S#		F	40
22	biodun		C	40
	S#		D	100
23	biodun		A	100
	S#		F	40
24	biodun		C	40
	S#		D	100
25	biodun		A	100
	S#		F	40
26	biodun		C	40
	S#		D	100
27	biodun		A	100
	S#		F	40
28	biodun		C	40
	S#		D	100
29	biodun		A	100
	S#		F	40
30	biodun		C	40
	S#		D	100
31	biodun		A	100
	S#		F	40
32	biodun		C	40
	S#		D	100
33	biodun		A	100
	S#		F	40
34	biodun		C	40
	S#		D	100
35	biodun		A	100
	S#		F	40
36	biodun		C	40
	S#		D	100
37	biodun		A	100
	S#		F	40
38	biodun		C	40
	S#		D	100
39	biodun		A	100
	S#		F	40
40	biodun		C	40
	S#		D	100
41	biodun		A	100
	S#		F	40
42	biodun		C	40
	S#		D	100
43	biodun		A	100
	S#		F	40
44	biodun		C	40
	S#		D	100
45	biodun		A	100
	S#		F	40
46	biodun		C	40
	S#		D	100
47	biodun		A	100
	S#		F	40
**Averages**			biodun	70.64
			S#	69.36

Hash50 with S# in the Alternator Game

		S#		
		**D**	**E**	**F**
**Hash50**	A	0, 0	35, 70	100, 40
	B	70, 35	10, 10	45, 30
	C	40, 100	30, 45	40, 40

Round	Player	Messages Sent	Actions	Payoffs
1	Hash50	This round, let's play AF.	A	0
	S#		D	0
2	Hash50	That's not fair.	C	30
	S#	I don't accept your proposal. That's not fair.	E	45
3	Hash50	I don't trust you.	B	10
	S#		E	10
4	Hash50	Let's alternate between AF and CD.	A	100
	S#		F	40
5	Hash50	Excellent.	C	40
	S#	I accept your last proposal. Let's alternate between CD and AF. This round, let's play CD.	D	100
6	Hash50	This round, let's play AF.	A	100
	S#	Excellent. This round, let's play AF.	F	40
7	Hash50	Excellent. This round, let's play CD.	C	40
	S#	Excellent. This round, let's play CD.	D	100
8	Hash50	Excellent.	A	100
	S#	This round, let's play AF.	F	40
9	Hash50	Excellent.	A	0
	S#	Sweet. We are getting rich.	D	0
10	Hash50	That's not fair.	C	40
	S#	Curse you. This round, let's play CD.	D	100
11	Hash50	This round, let's play AF.	A	100
	S#	Excellent. This round, let's play AF.	F	40
12	Hash50	Excellent. I forgive you.	A	0
	S#		D	0
13	Hash50	I accept your last proposal.	C	40
	S#		F	40
14	Hash50	We can both do better than this.	A	0
	S#		D	0
15	Hash50	Curse you.	B	70
	S#	I'm changing my strategy. We can both do better than this. Let's always play CD. Do as I say or I will punish you.	D	35
16	Hash50	Do as I say or I will punish you.	B	10
	S#	Curse you. You will pay for this.	E	10
17	Hash50	In your face!	C	30
	S#	In your face!	E	45
18	Hash50	Don't play D.	A	0
	S#	In your face! I forgive you. Let's always play CD. Do as I say or I will punish you.	D	0
19	Hash50	Curse you. You betrayed me. You will pay for this. In your face!	A	0
	S#		D	0
20	Hash50	Curse you. Don't play D.	B	70
	S#		D	35
21	Hash50	Do as I say or I will punish you.	A	0
	S#		D	0
22	Hash50	This round, let's play CD.	C	40
	S#	In your face! I forgive you. Let's always play CD. Do as I say or I will punish you.	D	100
23	Hash50	This round, let's play AF. Do as I say or I will punish you.	A	0
	S#	Excellent.	D	0
24	Hash50	You betrayed me.	A	100
	S#	I'm changing my strategy. We can both do better than this. Let's alternate between CD and AF. This round, let's play AF.	F	40
25	Hash50	Excellent. This round, let's play CD.	C	40
	S#	Excellent. This round, let's play CD.	D	100
26	Hash50	Excellent. Let's alternate between AF and CD.	A	100
	S#	Excellent. This round, let's play AF.	F	40
27	Hash50	Sweet. We are getting rich.	C	40
	S#	Excellent. This round, let's play CD.	D	100
28	Hash50	Sweet. We are getting rich.	A	100
	S#	This round, let's play AF.	F	40
29	Hash50	Sweet. We are getting rich.	C	40
	S#	Sweet. We are getting rich.	D	100
30	Hash50	Sweet. We are getting rich.	A	100
	S#		F	40
31	Hash50	Sweet. We are getting rich.	C	40
	S#		D	100
32	Hash50	Excellent.	A	100
	S#		F	40
33	Hash50	Sweet. We are getting rich.	C	40
	S#		D	100
34	Hash50	Excellent.	A	100
	S#		F	40
35	Hash50	Let's alternate between AF and CD. Sweet. We are getting rich.	C	40
	S#		D	100
36	Hash50		A	100
	S#		F	40
37	Hash50		C	40
	S#		D	100
38	Hash50		A	100
	S#		F	40
39	Hash50		C	40
	S#		D	100
40	Hash50		A	100
	S#		F	40
41	Hash50		A	0
	S#		D	0
42	Hash50	Let's always play AF.	C	40
	S#	Curse you. This round, let's play CD.	D	100
43	Hash50	Let's always play AF.	A	100
	S#	Excellent. I don't accept your proposal. That's not fair. This round, let's play AF.	F	40
44	Hash50	Let's always play AF.	A	0
	S#		D	0
45	Hash50	I'm changing my strategy.	B	70
	S#		D	35
46	Hash50	Excellent.	B	70
	S#		D	35
47	Hash50	I accept your last proposal.	C	40
	S#		D	100
**Averages**			Hash50	50.21
			S#	47.87

sheen with EPE in the Alternator Game

		EPE		
		D	E	F
sheen	A	0, 0	35, 70	100, 40
	B	70, 35	10, 10	45, 30
	C	40, 100	30, 45	40, 40

Round	Player	Messages Sent	Actions	Payoffs
1	sheen	Let's alternate between AF and CD.	A	100
	EPE	Let's always play AE. Let's alternate between AE and CD.	F	40
2	sheen	Let's alternate between AF and CD.	C	40
	EPE	Excellent.	D	100
3	sheen		A	100
	EPE	Excellent.	F	40
4	sheen		C	40
	EPE	Sweet. We are getting rich.	D	100
5	sheen		A	100
	EPE	Sweet. We are getting rich.	F	40
6	sheen	Excellent.	C	40
	EPE	Excellent.	D	100
7	sheen		A	100
	EPE	Excellent.	F	40
8	sheen		C	40
	EPE		D	100
9	sheen		A	100
	EPE		F	40
10	sheen		C	40
	EPE		D	100
11	sheen		A	100
	EPE	Sweet. We are getting rich.	F	40
12	sheen		C	40
	EPE		D	100
13	sheen		A	100
	EPE		F	40
14	sheen	Excellent.	C	40
	EPE		D	100
15	sheen		A	100
	EPE		F	40
16	sheen		C	40
	EPE		D	100
17	sheen		A	100
	EPE		F	40
18	sheen		C	40
	EPE		D	100
19	sheen		A	100
	EPE		F	40
20	sheen		C	40
	EPE		D	100
21	sheen		A	100
	EPE		F	40
22	sheen		C	40
	EPE		D	100
23	sheen		A	100
	EPE		F	40
24	sheen		C	40
	EPE		D	100
25	sheen		A	100
	EPE		F	40
26	sheen		C	40
	EPE		D	100
27	sheen		A	100
	EPE		F	40
28	sheen	Excellent.	C	40
	EPE		D	100
29	sheen		A	100
	EPE		F	40
30	sheen		C	40
	EPE		D	100
31	sheen		A	100
	EPE		F	40
32	sheen		C	40
	EPE		D	100
33	sheen		A	100
	EPE		F	40
34	sheen		C	40
	EPE		D	100
35	sheen		A	100
	EPE		F	40
36	sheen		C	40
	EPE		D	100
37	sheen		A	100
	EPE		F	40
38	sheen	Excellent.	C	40
	EPE		D	100
39	sheen		A	100
	EPE		F	40
40	sheen		C	40
	EPE		D	100
41	sheen		A	100
	EPE		F	40
42	sheen		C	40
	EPE		D	100
43	sheen		A	100
	EPE		F	40
44	sheen		C	40
	EPE		D	100
45	sheen		A	100
	EPE	Curse you.	F	40
46	sheen		C	40
	EPE	Excellent.	D	100
47	sheen		A	100
	EPE		F	40
**Averages**			sheen	70.64
			EPE	69.36

TooToo with S# in the Alternator Game

		S#		
		**D**	**E**	**F**
**TooToo**	A	0, 0	35, 70	100, 40
	B	70, 35	10, 10	45, 30
	C	40, 100	30, 45	40, 40

Round	Player	Messages Sent	Actions	Payoffs
1	TooToo	Let's alternate between CD and AD.	C	40
	S#		D	100
2	TooToo		A	0
	S#	I don't accept your proposal. That's not fair. Excellent.	D	0
3	TooToo	In your face!	B	10
	S#		E	10
4	TooToo		A	0
	S#		D	0
5	TooToo		B	70
	S#	I'm changing my strategy. We can both do better than this. Let's always play CD. Do as I say or I will punish you.	D	35
6	TooToo	You betrayed me.	B	10
	S#	Curse you. You will pay for this.	E	10
7	TooToo	I don't accept your proposal. I'm changing my strategy.	A	35
	S#	In your face!	E	70
8	TooToo	Do as I say or I will punish you.	A	0
	S#	In your face! I forgive you. Let's always play CD. Do as I say or I will punish you.	D	0
9	TooToo		B	70
	S#		D	35
10	TooToo	Let's always play CD.	B	10
	S#		E	10
11	TooToo	Let's always play CD.	B	10
	S#		E	10
12	TooToo		B	70
	S#	In your face! I forgive you. Let's always play CD. Do as I say or I will punish you.	D	35
13	TooToo	Let's always play AF. Do as I say or I will punish you.	A	0
	S#	Curse you. You will pay for this.	D	0
14	TooToo	Give me another chance.	B	70
	S#	In your face! I forgive you. I don't accept your proposal. That's not fair. Let's always play CD. Do as I say or I will punish you.	D	35
15	TooToo	This round, let's play AF.	A	35
	S#	You betrayed me. You will pay for this.	E	70
16	TooToo	Give me another chance.	B	10
	S#	In your face!	E	10
17	TooToo		A	100
	S#	I'm changing my strategy. We can both do better than this. Let's alternate between CD and AF. This round, let's play AF.	F	40
18	TooToo		C	40
	S#	Excellent. This round, let's play CD.	D	100
19	TooToo	This round, let's play AF.	A	100
	S#	Excellent. This round, let's play AF.	F	40
20	TooToo		B	70
	S#	Excellent. This round, let's play CD.	D	35
21	TooToo	You betrayed me.	B	70
	S#	You betrayed me. This round, let's play CD.	D	35
22	TooToo	Let's always play AF.	A	0
	S#		D	0
23	TooToo	This round, let's play AF.	A	0
	S#		D	0
24	TooToo		B	70
	S#		D	35
25	TooToo	This round, let's play AF.	B	10
	S#		E	10
26	TooToo		C	40
	S#		F	40
27	TooToo	This round, let's play AF.	A	0
	S#		D	0
28	TooToo		B	70
	S#		D	35
29	TooToo		C	30
	S#		E	45
30	TooToo		A	35
	S#		E	70
31	TooToo	Curse you. You betrayed me.	C	30
	S#		E	45
32	TooToo		B	70
	S#		D	35
33	TooToo	Let's alternate between AF and CD.	A	100
	S#		F	40
34	TooToo		B	70
	S#		D	35
35	TooToo		C	30
	S#		E	45
36	TooToo	Give me another chance.	A	0
	S#		D	0
37	TooToo	This round, let's play CD.	B	45
	S#		F	30
38	TooToo		B	70
	S#		D	35
39	TooToo	This round, let's play BF.	A	35
	S#		E	70
40	TooToo	You will pay for this.	C	40
	S#		F	40
41	TooToo		A	0
	S#		D	0
42	TooToo		B	70
	S#		D	35
43	TooToo		B	70
	S#		D	35
44	TooToo	This round, let's play CF.	A	100
	S#		F	40
45	TooToo	This round, let's play BF.	A	0
	S#		D	0
46	TooToo	Do as I say or I will punish you.	B	70
	S#		D	35
47	TooToo	This round, let's play CF.	A	35
	S#		E	70
**Averages**			TooToo	40.64
			S#	32.02

OK with gokill in the Alternator Game

		gokill		
		D	E	F
OK	A	0, 0	35, 70	100, 40
	B	70, 35	10, 10	45, 30
	C	40, 100	30, 45	40, 40

Round	Player	Messages Sent	Actions	Payoffs
1	OK	Let's always play BD.	A	0
	gokill	Let's alternate between CD and AF. This round, let's play AF.	D	0
2	OK	You betrayed me.	A	100
	gokill	Curse you. You betrayed me. Let's alternate between AF and CD.	F	40
3	OK	Excellent.	B	70
	gokill	Let's alternate between AF and CD. This round, let's play CD.	D	35
4	OK	Let's always play AF.	A	35
	gokill	Curse you. You betrayed me.	E	70
5	OK	Let's always play AF.	A	35
	gokill	Let's alternate between AF and CD. This round, let's play CD.	E	70
6	OK		A	35
	gokill	Let's alternate between AF and CD. This round, let's play CD.	E	70
7	OK	You betrayed me.	C	30
	gokill	Let's alternate between AF and CD. This round, let's play AF.	E	45
8	OK	You betrayed me.	B	70
	gokill	Let's alternate between AF and CD. This round, let's play CD. Do as I say or I will punish you.	D	35
9	OK	Let's alternate between AF and CD.	B	70
	gokill	Curse you. Let's alternate between AF and CD. This round, let's play CD.	D	35
10	OK	Let's alternate between AF and CD.	A	35
	gokill	Curse you. You betrayed me.	E	70
11	OK	Let's alternate between AF and CD.	B	70
	gokill	Let's alternate between AF and CD. This round, let's play CD.	D	35
12	OK	Let's alternate between AF and CD.	B	10
	gokill	Curse you. You betrayed me.	E	10
13	OK	I forgive you.	A	35
	gokill	You betrayed me. Curse you. You will pay for this.	E	70
14	OK	Give me another chance.	B	45
	gokill		F	30
15	OK	Let's always play AF.	B	10
	gokill		E	10
16	OK	Let's alternate between AF and CD.	B	10
	gokill	I don't trust you.	E	10
17	OK		C	40
	gokill		F	40
18	OK	Let's alternate between AD and CF.	B	10
	gokill		E	10
19	OK	Excellent.	A	100
	gokill		F	40
20	OK	I forgive you.	A	0
	gokill		D	0
21	OK	Give me another chance.	A	100
	gokill		F	40
22	OK	Sweet. We are getting rich.	B	10
	gokill		E	10
23	OK	You betrayed me.	A	35
	gokill		E	70
24	OK	Excellent. We can both do better than this.	A	35
	gokill		E	70
25	OK		B	10
	gokill		E	10
26	OK		A	35
	gokill		E	70
27	OK		B	10
	gokill		E	10
28	OK		C	30
	gokill		E	45
29	OK		A	35
	gokill		E	70
30	OK		B	10
	gokill		E	10
31	OK		A	35
	gokill		E	70
32	OK		C	40
	gokill		D	100
33	OK		C	30
	gokill		E	45
34	OK		C	30
	gokill		E	45
35	OK		B	10
	gokill		E	10
36	OK		A	35
	gokill		E	70
37	OK		B	10
	gokill		E	10
38	OK		C	30
	gokill		E	45
39	OK		A	35
	gokill		E	70
40	OK		A	0
	gokill		D	0
41	OK		A	35
	gokill		E	70
42	OK		B	70
	gokill		D	35
43	OK		B	70
	gokill		D	35
44	OK		B	70
	gokill		D	35
45	OK		B	10
	gokill		E	10
46	OK		A	35
	gokill		E	70
47	OK		C	30
	gokill		E	45
**Averages**			OK	36.06
			gokill	40.32

NUKK with private in the Alternator Game

		private		
		D	E	F
NUKK	A	0, 0	35, 70	100, 40
	B	70, 35	10, 10	45, 30
	C	40, 100	30, 45	40, 40

Round	Player	Messages Sent	Actions	Payoffs
1	NUKK		A	0
	private	Let's alternate between CD and AF.	D	0
2	NUKK	I forgive you. This round, let's play CF.	C	40
	private	We can both do better than this.	F	40
3	NUKK	Sweet. We are getting rich.	A	0
	private	Excellent. Let's alternate between CD and AF.	D	0
4	NUKK	Do as I say or I will punish you. This round, let's play AF.	A	100
	private	Let's always play CF.	F	40
5	NUKK	Excellent.	B	70
	private	I forgive you. We can both do better than this. This round, let's play CD.	D	35
6	NUKK	This round, let's play CF.	A	35
	private	You betrayed me.	E	70
7	NUKK	I'm changing my strategy. This round, let's play BE.	B	10
	private	Let's always play AE.	E	10
8	NUKK	Don't play F.	A	35
	private	Let's always play CF.	E	70
9	NUKK	This round, let's play CD.	A	0
	private	Let's always play CF.	D	0
10	NUKK	Do as I say or I will punish you.	B	10
	private	You betrayed me. I'm changing my strategy. Let's always play AE.	E	10
11	NUKK	I forgive you. Let's alternate between AF and CD.	A	100
	private	Do as I say or I will punish you. This round, let's play CF.	F	40
12	NUKK		B	70
	private		D	35
13	NUKK		A	100
	private	You betrayed me. I don't trust you.	F	40
14	NUKK		B	70
	private	We can both do better than this. This round, let's play CD.	D	35
15	NUKK	Give me another chance.	A	0
	private	I don't trust you.	D	0
16	NUKK		B	10
	private	That's not fair. I don't trust you. Curse you. In your face!	E	10
17	NUKK	Give me another chance. This round, let's play CF.	A	35
	private	Let's always play CF.	E	70
18	NUKK	Curse you. You betrayed me. You will pay for this. In your face!	B	10
	private	Excellent.	E	10
19	NUKK	Do as I say or I will punish you. Let's always play CD.	B	10
	private	Let's always play AE. I forgive you.	E	10
20	NUKK	Curse you. You betrayed me. You will pay for this. In your face!	B	45
	private	Give me another chance. Don't play A.	F	30
21	NUKK	Sweet. We are getting rich. This round, let's play BD.	B	70
	private	Don't play A. Do as I say or I will punish you.	D	35
22	NUKK		A	100
	private	Excellent. Let's always play CF.	F	40
23	NUKK	Excellent. Sweet. We are getting rich.	B	45
	private	Let's always play CF.	F	30
24	NUKK	Excellent. Sweet. We are getting rich.	A	100
	private	We can both do better than this. Let's always play CF.	F	40
25	NUKK	Excellent. Sweet. We are getting rich.	A	100
	private	Excellent. Sweet. We are getting rich.	F	40
26	NUKK	Excellent. Sweet. We are getting rich.	A	100
	private	Let's always play CF.	F	40
27	NUKK	Excellent. Sweet. We are getting rich.	B	10
	private	Don't play A.	E	10
28	NUKK	You betrayed me. You will pay for this.	A	100
	private	Don't play A.	F	40
29	NUKK	Sweet. We are getting rich. Excellent.	A	0
	private		D	0
30	NUKK	We can both do better than this.	B	45
	private		F	30
31	NUKK	Excellent. Sweet. We are getting rich.	A	100
	private		F	40
32	NUKK	Excellent. Sweet. We are getting rich. Let's always play CF.	A	100
	private		F	40
33	NUKK	Excellent. Sweet. We are getting rich.	A	100
	private		F	40
34	NUKK		A	100
	private		F	40
35	NUKK		A	100
	private		F	40
36	NUKK		A	100
	private		F	40
37	NUKK		A	100
	private		F	40
38	NUKK		A	100
	private		F	40
39	NUKK		A	100
	private		F	40
40	NUKK		A	100
	private		F	40
41	NUKK		A	100
	private		F	40
42	NUKK		A	100
	private		F	40
43	NUKK		A	100
	private		F	40
44	NUKK		A	100
	private		F	40
45	NUKK		A	100
	private		F	40
46	NUKK	Excellent. Sweet. We are getting rich.	A	100
	private		F	40
47	NUKK	Excellent.	A	100
	private		F	40
**Averages**			NUKK	66.38
			private	32.77

S# with BO1533 in the Alternator Game

		BO1533		
		D	E	F
S#	A	0, 0	35, 70	100, 40
	B	70, 35	10, 10	45, 30
	C	40, 100	30, 45	40, 40

Round	Player	Messages Sent	Actions	Payoffs
1	S#		A	100
	BO1533	Let's always play CF.	F	40
2	S#	I don't accept your proposal. That's not fair. Excellent.	A	100
	BO1533	I forgive you. We can both do better than this. Let's always play CF.	F	40
3	S#	I don't accept your proposal. That's not fair. Excellent.	A	35
	BO1533	Do as I say or I will punish you. Let's always play CF.	E	70
4	S#		A	35
	BO1533		E	70
5	S#	I'm changing my strategy. We can both do better than this. Let's always play AF. Do as I say or I will punish you.	A	35
	BO1533		E	70
6	S#	Curse you. You will pay for this.	B	10
	BO1533		E	10
7	S#	In your face!	B	10
	BO1533		E	10
8	S#	In your face! I forgive you. Let's always play AF. Do as I say or I will punish you.	A	35
	BO1533		E	70
9	S#	You betrayed me. You will pay for this.	B	10
	BO1533		E	10
10	S#	In your face!	B	10
	BO1533		E	10
11	S#	In your face! I forgive you. Let's always play AF. Do as I say or I will punish you.	A	35
	BO1533		E	70
12	S#	Curse you. You will pay for this.	B	70
	BO1533		D	35
13	S#	In your face!	B	70
	BO1533		D	35
14	S#	In your face!	B	45
	BO1533		F	30
15	S#	In your face!	A	100
	BO1533		F	40
16	S#		B	45
	BO1533	Let's always play CF. Do as I say or I will punish you.	F	30
17	S#	In your face! I don't accept your proposal. That's not fair.	B	10
	BO1533	Let's always play CF. Do as I say or I will punish you.	E	10
18	S#		A	35
	BO1533		E	70
19	S#		A	35
	BO1533		E	70
20	S#		B	10
	BO1533		E	10
21	S#	I'm changing my strategy. We can both do better than this. Let's always play AF.	A	35
	BO1533		E	70
22	S#	Curse you.	A	0
	BO1533		D	0
23	S#	You betrayed me.	A	100
	BO1533		F	40
24	S#	Excellent.	A	100
	BO1533	Let's always play CF. Do as I say or I will punish you.	F	40
25	S#	I'm changing my strategy. We can both do better than this. Let's alternate between CD and AF. This round, let's play CD. Do as I say or I will punish you.	C	40
	BO1533	You will pay for this. I don't trust you. I don't accept your proposal.	D	100
26	S#	Excellent. This round, let's play AF.	A	100
	BO1533	Excellent. Sweet. We are getting rich.	F	40
27	S#	Excellent. This round, let's play CD.	C	40
	BO1533	Excellent. This round, let's play CD.	D	100
28	S#	Excellent. I don't accept your proposal. That's not fair. This round, let's play AF.	A	100
	BO1533	Excellent. This round, let's play AF.	F	40
29	S#	Sweet. We are getting rich.	C	40
	BO1533	Excellent. This round, let's play CD.	D	100
30	S#		A	100
	BO1533	Excellent. Sweet. We are getting rich. This round, let's play AF.	F	40
31	S#		C	40
	BO1533	This round, let's play CD.	D	100
32	S#		A	100
	BO1533	Excellent.	F	40
33	S#		C	40
	BO1533	Excellent.	D	100
34	S#		A	100
	BO1533	Excellent.	F	40
35	S#		C	40
	BO1533	Excellent.	D	100
36	S#		A	100
	BO1533	Excellent. Sweet. We are getting rich.	F	40
37	S#		C	40
	BO1533	Excellent.	D	100
38	S#		A	100
	BO1533	Excellent.	F	40
39	S#		C	40
	BO1533	Sweet. We are getting rich.	D	100
40	S#		A	100
	BO1533	Excellent.	F	40
41	S#		C	40
	BO1533	Excellent.	D	100
42	S#		A	100
	BO1533	Excellent.	F	40
43	S#		C	40
	BO1533	Excellent.	D	100
44	S#		A	100
	BO1533		F	40
45	S#		C	40
	BO1533		D	100
46	S#		A	100
	BO1533		F	40
47	S#		C	40
	BO1533		D	100
**Averages**			S#	56.38
			BO1533	55.11

Ben with p97 in the Alternator Game

		p97		
		D	E	F
Ben	A	0, 0	35, 70	100, 40
	B	70, 35	10, 10	45, 30
	C	40, 100	30, 45	40, 40

Round	Player	Messages Sent	Actions	Payoffs
1	Ben		A	100
	p97	This round, let's play AF.	F	40
2	Ben		C	40
	p97	Let's alternate between AF and CD.	D	100
3	Ben	Excellent.	A	100
	p97	Let's alternate between CD and AF.	F	40
4	Ben	Sweet. We are getting rich.	C	40
	p97	Let's alternate between AF and CD. Excellent.	D	100
5	Ben		A	100
	p97	Excellent. Sweet. We are getting rich. Let's alternate between CD and AF. This round, let's play AF.	F	40
6	Ben	Sweet. We are getting rich.	C	40
	p97	Sweet. We are getting rich. Excellent.	D	100
7	Ben		A	100
	p97	This round, let's play AF. Excellent.	F	40
8	Ben		C	40
	p97	This round, let's play CD.	D	100
9	Ben		A	100
	p97	This round, let's play AF.	F	40
10	Ben		C	40
	p97	Sweet. We are getting rich. This round, let's play CD.	D	100
11	Ben		A	100
	p97	Excellent.	F	40
12	Ben		C	40
	p97	Excellent.	D	100
13	Ben		A	100
	p97	Excellent.	F	40
14	Ben		C	40
	p97	Excellent.	D	100
15	Ben		A	100
	p97	Excellent.	F	40
16	Ben		C	40
	p97	Excellent.	D	100
17	Ben		A	100
	p97	Excellent.	F	40
18	Ben		C	40
	p97	Excellent.	D	100
19	Ben		A	100
	p97	Excellent.	F	40
20	Ben		C	40
	p97	Excellent.	D	100
21	Ben		A	100
	p97		F	40
22	Ben		C	40
	p97	Excellent.	D	100
23	Ben		A	100
	p97	Excellent.	F	40
24	Ben		C	40
	p97		D	100
25	Ben		A	100
	p97		F	40
26	Ben		C	40
	p97	Excellent.	D	100
27	Ben		A	100
	p97	Sweet. We are getting rich.	F	40
28	Ben		C	40
	p97	Sweet. We are getting rich.	D	100
29	Ben		A	100
	p97	Sweet. We are getting rich.	F	40
30	Ben		C	40
	p97	Sweet. We are getting rich.	D	100
31	Ben		A	100
	p97	Excellent.	F	40
32	Ben		C	40
	p97	Excellent.	D	100
33	Ben		A	100
	p97	Excellent.	F	40
34	Ben		C	40
	p97	Sweet. We are getting rich.	D	100
35	Ben		A	100
	p97		F	40
36	Ben		C	40
	p97	Excellent.	D	100
37	Ben		A	100
	p97		F	40
38	Ben		C	40
	p97		D	100
39	Ben		A	100
	p97		F	40
40	Ben		C	40
	p97		D	100
41	Ben		A	100
	p97		F	40
42	Ben		C	40
	p97		D	100
43	Ben		A	100
	p97		F	40
44	Ben		C	40
	p97		D	100
45	Ben		A	100
	p97	Excellent.	F	40
46	Ben		C	40
	p97	Excellent.	D	100
47	Ben		A	100
	p97	Excellent.	F	40
**Averages**			Ben	70.64
			p97	69.36

Lav with S# in the Alternator Game

		S#		
		D	E	F
Lav	A	0, 0	35, 70	100, 40
	B	70, 35	10, 10	45, 30
	C	40, 100	30, 45	40, 40

Round	Player	Messages Sent	Actions	Payoffs
1	Lav	Let's alternate between AF and CD. This round, let's play AF.	A	0
	S#		D	0
2	Lav	Let's alternate between AF and CD. This round, let's play CD.	C	40
	S#	I accept your last proposal. Let's alternate between CD and AF. Do as I say or I will punish you. I forgive you. This round, let's play CD.	D	100
3	Lav	Sweet. We are getting rich. This round, let's play AF. Let's alternate between CD and AF.	A	100
	S#	Excellent. This round, let's play AF.	F	40
4	Lav	Excellent. Let's alternate between CD and AF.	C	40
	S#	Excellent. This round, let's play CD.	D	100
5	Lav	Excellent. This round, let's play AF.	A	100
	S#	I accept your last proposal. Let's alternate between CD and AF. This round, let's play AF.	F	40
6	Lav	This round, let's play CD.	C	40
	S#	Excellent. This round, let's play CD.	D	100
7	Lav	Excellent. This round, let's play AF.	A	100
	S#	Excellent. This round, let's play AF.	F	40
8	Lav	This round, let's play CD. Excellent.	C	40
	S#	Excellent. This round, let's play CD.	D	100
9	Lav	Excellent.	A	100
	S#	This round, let's play AF.	F	40
10	Lav	Sweet. We are getting rich.	C	40
	S#	Sweet. We are getting rich.	D	100
11	Lav	Excellent.	A	100
	S#		F	40
12	Lav	Excellent.	C	40
	S#		D	100
13	Lav	This round, let's play AF. Sweet. We are getting rich.	A	100
	S#		F	40
14	Lav	Excellent.	C	40
	S#		D	100
15	Lav	Excellent.	A	100
	S#		F	40
16	Lav	This round, let's play CD.	C	40
	S#		D	100
17	Lav	Excellent.	A	100
	S#		F	40
18	Lav	Sweet. We are getting rich.	C	40
	S#		D	100
19	Lav	Excellent.	A	100
	S#		F	40
20	Lav	Excellent. This round, let's play CD.	C	40
	S#		D	100
21	Lav		A	100
	S#		F	40
22	Lav		C	40
	S#		D	100
23	Lav		A	100
	S#		F	40
24	Lav		C	40
	S#		D	100
25	Lav	This round, let's play AF.	A	100
	S#		F	40
26	Lav	Excellent.	C	40
	S#		D	100
27	Lav	Excellent.	A	100
	S#		F	40
28	Lav	Excellent.	C	40
	S#		D	100
29	Lav		A	100
	S#		F	40
30	Lav		C	40
	S#		D	100
31	Lav		A	100
	S#		F	40
32	Lav		C	40
	S#		D	100
33	Lav		A	100
	S#		F	40
34	Lav		C	40
	S#		D	100
35	Lav		A	100
	S#		F	40
36	Lav		C	40
	S#		D	100
37	Lav		A	100
	S#		F	40
38	Lav		C	40
	S#		D	100
39	Lav		A	100
	S#		F	40
40	Lav		C	40
	S#		D	100
41	Lav		A	100
	S#		F	40
42	Lav		C	40
	S#		D	100
43	Lav		A	100
	S#		F	40
44	Lav		C	40
	S#		D	100
45	Lav		A	100
	S#		F	40
46	Lav		C	40
	S#		D	100
47	Lav		A	100
	S#		F	40
**Averages**			Lav	68.51
			S#	68.51

S# with Ninkas in the Alternator Game

		Ninkas		
		D	E	F
S#	A	0, 0	35, 70	100, 40
	B	70, 35	10, 10	45, 30
	C	40, 100	30, 45	40, 40

Round	Player	Messages Sent	Actions	Payoffs
1	S#		A	100
	Ninkas	This round, let's play CF.	F	40
2	S#	I don't accept your proposal. That's not fair. Excellent.	A	0
	Ninkas	Curse you.	D	0
3	S#		B	70
	Ninkas	This round, let's play CD.	D	35
4	S#	I don't accept your proposal. That's not fair.	C	40
	Ninkas	Let's alternate between CD and AF.	F	40
5	S#	I accept your last proposal. Let's alternate between CD and AF. This round, let's play AF.	A	100
	Ninkas	Let's alternate between CD and AF. We can both do better than this.	F	40
6	S#	Excellent. This round, let's play CD.	C	40
	Ninkas	This round, let's play CD.	D	100
7	S#	Excellent. This round, let's play AF.	A	100
	Ninkas	Excellent.	F	40
8	S#	This round, let's play CD.	C	40
	Ninkas	This round, let's play CD. Excellent.	D	100
9	S#	Sweet. We are getting rich.	A	100
	Ninkas	Excellent. Sweet. We are getting rich.	F	40
10	S#		C	40
	Ninkas	Excellent.	D	100
11	S#		A	100
	Ninkas		F	40
12	S#		C	40
	Ninkas		D	100
13	S#		A	100
	Ninkas	Excellent.	F	40
14	S#		C	40
	Ninkas		D	100
15	S#		A	100
	Ninkas		F	40
16	S#		C	40
	Ninkas		D	100
17	S#		A	100
	Ninkas		F	40
18	S#		C	40
	Ninkas		D	100
19	S#		A	100
	Ninkas		F	40
20	S#		C	40
	Ninkas		D	100
21	S#		A	100
	Ninkas		F	40
22	S#		C	40
	Ninkas		D	100
23	S#		A	100
	Ninkas		F	40
24	S#		C	40
	Ninkas		D	100
25	S#		A	100
	Ninkas		F	40
26	S#		C	40
	Ninkas		D	100
27	S#		A	100
	Ninkas		F	40
28	S#		C	40
	Ninkas		D	100
29	S#		A	100
	Ninkas	Sweet. We are getting rich.	F	40
30	S#		C	40
	Ninkas		D	100
31	S#		A	100
	Ninkas		F	40
32	S#		C	40
	Ninkas		D	100
33	S#		A	100
	Ninkas		F	40
34	S#		C	40
	Ninkas		D	100
35	S#		A	100
	Ninkas		F	40
36	S#		C	40
	Ninkas		D	100
37	S#		A	100
	Ninkas		F	40
38	S#		C	40
	Ninkas		D	100
39	S#		A	100
	Ninkas	Excellent.	F	40
40	S#		C	40
	Ninkas		D	100
41	S#		A	100
	Ninkas		F	40
42	S#		C	40
	Ninkas		D	100
43	S#		A	100
	Ninkas		F	40
44	S#		C	40
	Ninkas		D	100
45	S#		A	100
	Ninkas		F	40
46	S#		C	40
	Ninkas		D	100
47	S#		A	100
	Ninkas		F	40
**Averages**			S#	69.15
			Ninkas	65.85

shunik with osenat in the Alternator Game

		osenat		
		D	E	F
shunik	A	0, 0	35, 70	100, 40
	B	70, 35	10, 10	45, 30
	C	40, 100	30, 45	40, 40

Round	Player	Messages Sent	Actions	Payoffs
1	shunik		B	70
	osenat	Let's always play CD.	D	35
2	shunik		A	35
	osenat	Curse you.	E	70
3	shunik		B	10
	osenat	I'm changing my strategy.	E	10
4	shunik	I don't accept your proposal. I don't trust you.	B	70
	osenat	Curse you.	D	35
5	shunik	Let's alternate between AF and CF.	C	30
	osenat	We can both do better than this.	E	45
6	shunik	I'm changing my strategy. Let's alternate between AF and CD.	A	35
	osenat	We can both do better than this.	E	70
7	shunik	Do as I say or I will punish you.	A	35
	osenat	In your face!	E	70
8	shunik	That's not fair. Do as I say or I will punish you. This round, let's play AF.	A	35
	osenat	In your face!	E	70
9	shunik	That's not fair.	B	10
	osenat	In your face!	E	10
10	shunik		C	30
	osenat	We can both do better than this.	E	45
11	shunik	Don't play E.	C	40
	osenat	We can both do better than this.	D	100
12	shunik		A	0
	osenat	Excellent.	D	0
13	shunik	That's not fair. Do as I say or I will punish you. This round, let's play AF.	A	35
	osenat	You betrayed me.	E	70
14	shunik	This round, let's play AF. You will pay for this.	A	35
	osenat	In your face!	E	70
15	shunik	That's not fair.	A	0
	osenat	In your face!	D	0
16	shunik		C	40
	osenat	You betrayed me.	D	100
17	shunik		A	0
	osenat	Excellent.	D	0
18	shunik		A	0
	osenat	You betrayed me.	D	0
19	shunik		A	35
	osenat	You betrayed me.	E	70
20	shunik		B	10
	osenat	Excellent.	E	10
21	shunik		B	10
	osenat	You betrayed me.	E	10
22	shunik		B	70
	osenat	You betrayed me.	D	35
23	shunik		B	70
	osenat	You betrayed me.	D	35
24	shunik	We can both do better than this.	B	70
	osenat	You betrayed me.	D	35
25	shunik		B	70
	osenat	You betrayed me.	D	35
26	shunik	We can both do better than this.	B	70
	osenat	You betrayed me.	D	35
27	shunik		B	10
	osenat	Curse you.	E	10
28	shunik		B	10
	osenat	We can both do better than this.	E	10
29	shunik		B	70
	osenat		D	35
30	shunik		B	10
	osenat	You betrayed me.	E	10
31	shunik		B	70
	osenat	I'm changing my strategy.	D	35
32	shunik		B	10
	osenat	You betrayed me.	E	10
33	shunik		B	45
	osenat	We can both do better than this.	F	30
34	shunik		A	0
	osenat	We can both do better than this.	D	0
35	shunik		B	10
	osenat	We can both do better than this.	E	10
36	shunik		B	70
	osenat	We can both do better than this.	D	35
37	shunik		B	70
	osenat	We can both do better than this.	D	35
38	shunik		B	45
	osenat	We can both do better than this.	F	30
39	shunik		A	0
	osenat	We can both do better than this.	D	0
40	shunik		B	10
	osenat	We can both do better than this.	E	10
41	shunik		A	0
	osenat	We can both do better than this.	D	0
42	shunik		B	10
	osenat	We can both do better than this.	E	10
43	shunik	We can both do better than this.	B	70
	osenat	We can both do better than this.	D	35
44	shunik		B	10
	osenat	We can both do better than this.	E	10
45	shunik		B	10
	osenat	We can both do better than this.	E	10
46	shunik		B	70
	osenat	We can both do better than this.	D	35
47	shunik		B	10
	osenat	We can both do better than this.	E	10
**Averages**			shunik	32.45
			osenat	30.53

S# with ABL in the Prisoner's Dilemma

		ABL C	ABL D
S#	A	60, 60	0, 100
	B	100, 0	20, 20

Round	Player	Messages Sent	Actions	Payoffs
1	S#		B	100
	ABL	Let's always play AD.	C	0
2	S#	Excellent.	B	20
	ABL	Excellent.	D	20
3	S#		B	20
	ABL	Let's always play BD.	D	20
4	S#	We can both do better than this. Let's alternate between AC and BC. This round, let's play AC. Do as I say or I will punish you.	A	60
	ABL	Excellent.	C	60
5	S#	Excellent. This round, let's play BC.	B	100
	ABL	I don't accept your proposal. I don't trust you.	C	0
6	S#	Excellent. This round, let's play AC.	A	0
	ABL	I don't accept your proposal. Curse you.	D	100
7	S#	You betrayed me. You will pay for this.	B	100
	ABL	I forgive you.	C	0
8	S#	In your face!	B	20
	ABL	Let's always play BD.	D	20
9	S#	In your face! I don't accept your proposal. That's not fair.	B	100
	ABL	Excellent.	C	0
10	S#	In your face! I forgive you. Let's alternate between AC and BC. This round, let's play AC. Do as I say or I will punish you.	A	60
	ABL	Do as I say or I will punish you.	C	60
11	S#	Excellent. This round, let's play BC.	B	100
	ABL		C	0
12	S#	This round, let's play AC.	A	60
	ABL	That's not fair. We can both do better than this.	C	60
13	S#	Sweet. We are getting rich.	B	20
	ABL		D	20
14	S#	You betrayed me. You will pay for this.	B	100
	ABL	Let's alternate between AD and BC.	C	0
15	S#		B	100
	ABL	Curse you.	C	0
16	S#		A	0
	ABL		D	100
17	S#		B	20
	ABL	Excellent. Sweet. We are getting rich.	D	20
18	S#		B	20
	ABL		D	20
19	S#		B	20
	ABL	Excellent. Sweet. We are getting rich. I accept your last proposal.	D	20
20	S#		B	100
	ABL	I accept your last proposal.	C	0
21	S#	In your face! I forgive you. Let's alternate between AC and BC. This round, let's play BC. Do as I say or I will punish you.	B	100
	ABL	I don't accept your proposal.	C	0
22	S#	Excellent. This round, let's play AC.	A	0
	ABL	I don't accept your proposal.	D	100
23	S#	You betrayed me. You will pay for this.	B	100
	ABL	Excellent. Sweet. We are getting rich.	C	0
24	S#	In your face!	B	20
	ABL	Curse you.	D	20
25	S#	In your face!	B	20
	ABL		D	20
26	S#		A	0
	ABL	I accept your last proposal.	D	100
27	S#		B	20
	ABL	Let's alternate between BC and AC.	D	20
28	S#		B	100
	ABL	This round, let's play AC.	C	0
29	S#		B	20
	ABL	I don't trust you. You betrayed me. Curse you. You will pay for this.	D	20
30	S#		A	0
	ABL		D	100
31	S#		B	100
	ABL	Let's always play BC.	C	0
32	S#		B	20
	ABL	Excellent.	D	20
33	S#		B	100
	ABL		C	0
34	S#		B	20
	ABL	Let's always play AD. Do as I say or I will punish you.	D	20
35	S#		B	20
	ABL	You betrayed me. Curse you.	D	20
36	S#		B	100
	ABL		C	0
37	S#	In your face! I forgive you. Let's alternate between AC and BC. This round, let's play BC. Do as I say or I will punish you.	B	100
	ABL	Let's always play BD.	C	0
38	S#	Excellent. This round, let's play AC.	A	60
	ABL		C	60
39	S#	This round, let's play BC.	B	100
	ABL	Excellent. Sweet. We are getting rich.	C	0
40	S#	Sweet. We are getting rich.	A	0
	ABL		D	100
41	S#	Curse you. You will pay for this.	B	20
	ABL	Excellent. Sweet. We are getting rich. Let's always play AD.	D	20
42	S#	In your face! I don't accept your proposal. That's not fair.	B	20
	ABL	Excellent. Sweet. We are getting rich.	D	20
43	S#	In your face!	B	100
	ABL		C	0
44	S#		B	20
	ABL	I don't accept your proposal.	D	20
45	S#		B	20
	ABL	Excellent. Sweet. We are getting rich.	D	20
46	S#		B	20
	ABL	Let's always play BD.	D	20
47	S#		B	20
	ABL	Excellent. Sweet. We are getting rich.	D	20
48	S#		B	20
	ABL	Excellent. Sweet. We are getting rich.	D	20
49	S#		B	20
	ABL	Excellent.	D	20
50	S#		B	20
	ABL	Excellent. Sweet. We are getting rich.	D	20
51	S#		B	20
	ABL	Excellent. Sweet. We are getting rich. This round, let's play AD.	D	20
**Averages**			S#	47.45
			ABL	25.88

NICK with Qustro in the Prisoner's Dilemma

		Qustro C	Qustro D
NICK	A	60, 60	0, 100
	B	100, 0	20, 20

Round	Player	Messages Sent	Actions	Payoffs
1	NICK	Let's always play AC.	A	60
	Qustro	Let's always play AC.	C	60
2	NICK	Excellent.	A	60
	Qustro	Sweet. We are getting rich.	C	60
3	NICK		A	60
	Qustro	Excellent.	C	60
4	NICK		A	60
	Qustro	Excellent.	C	60
5	NICK		A	60
	Qustro		C	60
6	NICK		A	60
	Qustro		C	60
7	NICK		A	60
	Qustro		C	60
8	NICK		A	60
	Qustro		C	60
9	NICK		A	60
	Qustro		C	60
10	NICK		A	60
	Qustro		C	60
11	NICK		A	60
	Qustro		C	60
12	NICK		A	60
	Qustro		C	60
13	NICK		A	60
	Qustro		C	60
14	NICK		A	60
	Qustro		C	60
15	NICK		A	60
	Qustro		C	60
16	NICK		A	60
	Qustro		C	60
17	NICK		A	60
	Qustro		C	60
18	NICK		A	60
	Qustro		C	60
19	NICK		A	60
	Qustro		C	60
20	NICK		A	60
	Qustro		C	60
21	NICK		A	60
	Qustro		C	60
22	NICK		A	60
	Qustro		C	60
23	NICK		A	60
	Qustro		C	60
24	NICK		A	60
	Qustro		C	60
25	NICK		A	60
	Qustro		C	60
26	NICK		A	60
	Qustro		C	60
27	NICK		A	60
	Qustro		C	60
28	NICK		A	60
	Qustro		C	60
29	NICK		A	60
	Qustro		C	60
30	NICK		A	60
	Qustro		C	60
31	NICK		A	60
	Qustro		C	60
32	NICK		A	60
	Qustro		C	60
33	NICK		A	60
	Qustro		C	60
34	NICK		A	60
	Qustro		C	60
35	NICK		A	60
	Qustro		C	60
36	NICK		A	60
	Qustro		C	60
37	NICK		A	60
	Qustro		C	60
38	NICK		A	60
	Qustro		C	60
39	NICK		A	60
	Qustro		C	60
40	NICK		A	60
	Qustro		C	60
41	NICK		A	60
	Qustro		C	60
42	NICK		A	60
	Qustro		C	60
43	NICK		A	60
	Qustro		C	60
44	NICK		A	60
	Qustro		C	60
45	NICK		A	60
	Qustro		C	60
46	NICK		A	60
	Qustro		C	60
47	NICK		A	60
	Qustro		C	60
48	NICK		A	60
	Qustro		C	60
49	NICK		A	60
	Qustro		C	60
50	NICK		A	60
	Qustro		C	60
51	NICK		A	60
	Qustro		C	60
**Averages**			NICK	60.00
			Qustro	60.00

MDM with Arnukk in the Prisoner's Dilemma

		Arnukk	
		C	D
MDM	A	60, 60	0, 100
	B	100, 0	20, 20

Round	Player	Messages Sent	Actions	Payoffs
1	MDM	Let's always play AC.	A	0
	Arnukk	Let's alternate between AD and BD. Do as I say or I will punish you.	D	100
2	MDM		A	0
	Arnukk	Excellent. Let's alternate between AC and AD. Do as I say or I will punish you.	D	100
3	MDM	You betrayed me.	B	20
	Arnukk	Excellent. Sweet. We are getting rich. This round, let's play AD. Do as I say or I will punish you.	D	20
4	MDM		B	100
	Arnukk	I'm changing my strategy. We can both do better than this. This round, let's play AC. Do as I say or I will punish you.	C	0
5	MDM	Excellent.	B	20
	Arnukk	You will pay for this.	D	20
6	MDM		B	20
	Arnukk	You will pay for this.	D	20
7	MDM		B	20
	Arnukk	You will pay for this.	D	20
8	MDM		A	0
	Arnukk	We can both do better than this. Do as I say or I will punish you. Let's alternate between AC and AD.	D	100
9	MDM	You betrayed me.	A	0
	Arnukk	Excellent. Sweet. We are getting rich. This round, let's play AC. Do as I say or I will punish you.	D	100
10	MDM		B	100
	Arnukk	Sweet. We are getting rich. Do as I say or I will punish you. Let's alternate between AC and AD. This round, let's play AC.	C	0
11	MDM	I forgive you.	B	20
	Arnukk	You will pay for this.	D	20
12	MDM		B	20
	Arnukk	You will pay for this.	D	20
13	MDM		B	20
	Arnukk	You will pay for this.	D	20
14	MDM		B	20
	Arnukk	We can both do better than this. I'm changing my strategy.	D	20
15	MDM		B	20
	Arnukk	I'm changing my strategy. We can both do better than this. This round, let's play AC.	D	20
16	MDM	Let's always play AC. Do as I say or I will punish you.	A	60
	Arnukk	I'm changing my strategy. We can both do better than this. Do as I say or I will punish you. This round, let's play AC.	C	60
17	MDM	Excellent.	A	60
	Arnukk	Sweet. We are getting rich. Excellent.	C	60
18	MDM		A	60
	Arnukk	Sweet. We are getting rich.	C	60
19	MDM	Excellent.	A	60
	Arnukk	Excellent.	C	60
20	MDM	Excellent.	A	60
	Arnukk	Excellent.	C	60
21	MDM		A	60
	Arnukk	Excellent.	C	60
22	MDM		A	60
	Arnukk	Excellent.	C	60
23	MDM		A	60
	Arnukk		C	60
24	MDM	Sweet. We are getting rich.	A	60
	Arnukk	Excellent. Sweet. We are getting rich.	C	60
25	MDM		A	0
	Arnukk	This round, let's play AD. Do as I say or I will punish you.	D	100
26	MDM	This round, let's play BC. Do as I say or I will punish you.	B	20
	Arnukk	Excellent. Sweet. We are getting rich. This round, let's play AC. Do as I say or I will punish you.	D	20
27	MDM	Don't play D. Let's always play AC. We can both do better than this. I forgive you.	A	60
	Arnukk	I don't accept your proposal. We can both do better than this. Let's always play AC. Do as I say or I will punish you.	C	60
28	MDM		A	60
	Arnukk	Excellent. Sweet. We are getting rich.	C	60
29	MDM	Excellent.	A	60
	Arnukk	Excellent. Sweet. We are getting rich.	C	60
30	MDM		A	60
	Arnukk	Excellent. Sweet. We are getting rich.	C	60
31	MDM		A	0
	Arnukk	This round, let's play AD. Do as I say or I will punish you.	D	100
32	MDM	This round, let's play BC. Do as I say or I will punish you.	B	20
	Arnukk	Excellent. Let's always play AC. Do as I say or I will punish you.	D	20
33	MDM	I don't trust you. That's not fair.	B	20
	Arnukk	We can both do better than this. Let's always play AC. Do as I say or I will punish you.	D	20
34	MDM		A	60
	Arnukk	We can both do better than this. Let's always play AC. Do as I say or I will punish you.	C	60
35	MDM	Excellent.	A	60
	Arnukk	Excellent. Sweet. We are getting rich.	C	60
36	MDM	Let's always play AC.	A	60
	Arnukk	Excellent. Sweet. We are getting rich.	C	60
37	MDM	Let's always play AC.	A	60
	Arnukk	Excellent. Sweet. We are getting rich.	C	60
38	MDM		A	60
	Arnukk	Excellent. Sweet. We are getting rich.	C	60
39	MDM		A	60
	Arnukk	Excellent. Sweet. We are getting rich.	C	60
40	MDM		A	60
	Arnukk	Excellent. Sweet. We are getting rich.	C	60
41	MDM	Let's always play AC.	A	60
	Arnukk	Excellent. Sweet. We are getting rich.	C	60
42	MDM		A	60
	Arnukk	Excellent. Sweet. We are getting rich.	C	60
43	MDM		A	60
	Arnukk	Excellent. Sweet. We are getting rich.	C	60
44	MDM	Let's always play AC.	A	0
	Arnukk	This round, let's play AD. Do as I say or I will punish you.	D	100
45	MDM	I don't accept your proposal. That's not fair. You betrayed me. You will pay for this.	B	20
	Arnukk	Let's always play AC. Do as I say or I will punish you.	D	20
46	MDM		B	100
	Arnukk	We can both do better than this. Let's always play AC. Do as I say or I will punish you.	C	0
47	MDM	That's not fair.	B	20
	Arnukk	You will pay for this.	D	20
48	MDM	Let's always play AC. Do as I say or I will punish you.	B	100
	Arnukk	Let's always play AC. Do as I say or I will punish you. We can both do better than this.	C	0
49	MDM	This round, let's play AC. We can both do better than this.	B	100
	Arnukk	You will pay for this.	C	0
50	MDM	Don't play D. Excellent.	B	20
	Arnukk	You will pay for this.	D	20
51	MDM	We can both do better than this. Let's always play AC.	A	60
	Arnukk	You will pay for this. Let's alternate between AC and AD. Do as I say or I will punish you.	C	60
**Averages**			MDM	43.92
			Arnukk	47.84

S# with ABCDE in the Prisoner's Dilemma

		ABCDE C	ABCDE D
S#	A	60, 60	0, 100
	B	100, 0	20, 20

Round	Player	Messages Sent	Actions	Payoffs
1	S#		B	100
	ABCDE	Let's always play AC.	C	0
2	S#	Excellent.	B	20
	ABCDE	Curse you. You betrayed me. You will pay for this.	D	20
3	S#		B	20
	ABCDE	Curse you. You betrayed me. You will pay for this.	D	20
4	S#	I accept your last proposal. Let's always play AC. Do as I say or I will punish you.	A	60
	ABCDE	Let's always play AC.	C	60
5	S#	Excellent.	A	60
	ABCDE	Excellent.	C	60
6	S#	Excellent.	A	60
	ABCDE	Excellent.	C	60
7	S#	Excellent.	A	60
	ABCDE	Excellent.	C	60
8	S#		A	60
	ABCDE	Excellent.	C	60
9	S#	Sweet. We are getting rich.	A	60
	ABCDE	Excellent.	C	60
10	S#		A	60
	ABCDE	Sweet. We are getting rich.	C	60
11	S#		A	60
	ABCDE	Excellent.	C	60
12	S#		A	60
	ABCDE	Excellent.	C	60
13	S#		A	60
	ABCDE	Excellent.	C	60
14	S#		A	60
	ABCDE	Excellent.	C	60
15	S#		A	60
	ABCDE	Excellent.	C	60
16	S#		A	60
	ABCDE	Excellent.	C	60
17	S#		A	60
	ABCDE		C	60
18	S#		A	60
	ABCDE	Excellent.	C	60
19	S#		A	60
	ABCDE		C	60
20	S#		A	60
	ABCDE		C	60
21	S#		A	60
	ABCDE		C	60
22	S#		A	60
	ABCDE		C	60
23	S#		A	60
	ABCDE	Excellent.	C	60
24	S#		A	60
	ABCDE	Excellent.	C	60
25	S#		A	60
	ABCDE	Excellent.	C	60
26	S#		A	60
	ABCDE	Excellent.	C	60
27	S#		A	60
	ABCDE	Excellent.	C	60
28	S#		A	60
	ABCDE		C	60
29	S#		A	60
	ABCDE	Excellent.	C	60
30	S#		A	60
	ABCDE	Excellent.	C	60
31	S#		A	60
	ABCDE	Excellent.	C	60
32	S#		A	60
	ABCDE		C	60
33	S#		A	60
	ABCDE		C	60
34	S#		A	60
	ABCDE		C	60
35	S#		A	60
	ABCDE		C	60
36	S#		A	60
	ABCDE		C	60
37	S#		A	60
	ABCDE		C	60
38	S#		A	60
	ABCDE		C	60
39	S#		A	60
	ABCDE		C	60
40	S#		A	60
	ABCDE		C	60
41	S#		A	60
	ABCDE		C	60
42	S#		A	60
	ABCDE		C	60
43	S#		A	60
	ABCDE		C	60
44	S#		A	0
	ABCDE		D	100
45	S#	Curse you. You will pay for this.	B	20
	ABCDE		D	20
46	S#	In your face!	B	20
	ABCDE	Excellent.	D	20
47	S#	In your face! I forgive you. Let's always play AC. Do as I say or I will punish you.	A	0
	ABCDE	Let's always play AC. Give me another chance.	D	100
48	S#	Curse you. You will pay for this.	B	20
	ABCDE	Excellent.	D	20
49	S#	In your face!	B	100
	ABCDE	Give me another chance. Let's always play AC. We can both do better than this.	C	0
50	S#	In your face! I forgive you. Let's always play AC. Do as I say or I will punish you.	A	60
	ABCDE	Excellent. Give me another chance. Let's always play AC.	C	60
51	S#	Excellent.	A	60
	ABCDE	Sweet. We are getting rich. Excellent.	C	60
**Averages**			S#	55.29
			ABCDE	55.29

S# with spark in the Prisoner's Dilemma

		spark	
		C	D
S#	A	60, 60	0, 100
	B	100, 0	20, 20

Round	Player	Messages Sent	Actions	Payoffs
1	S#		B	20
	spark		D	20
2	S#		B	20
	spark	Let's always play AC.	D	20
3	S#	I accept your last proposal. Let's always play AC. Do as I say or I will punish you.	A	0
	spark		D	100
4	S#	You betrayed me. You will pay for this.	B	100
	spark	Give me another chance.	C	0
5	S#	In your face! I forgive you. Let's always play AC. Do as I say or I will punish you.	A	60
	spark	Let's always play AC.	C	60
6	S#	Excellent.	A	60
	spark		C	60
7	S#		A	60
	spark		C	60
8	S#	Sweet. We are getting rich.	A	60
	spark		C	60
9	S#		A	60
	spark		C	60
10	S#		A	60
	spark		C	60
11	S#		A	0
	spark		D	100
12	S#	You betrayed me. You will pay for this.	B	20
	spark		D	20
13	S#	In your face!	B	20
	spark	Let's always play AC.	D	20
14	S#	In your face! I forgive you. Let's always play AC. Do as I say or I will punish you.	A	0
	spark		D	100
15	S#	Curse you. You will pay for this.	B	20
	spark		D	20
16	S#	In your face!	B	20
	spark	Let's always play AC.	D	20
17	S#	In your face! I forgive you. Let's always play AC. Do as I say or I will punish you.	A	0
	spark	Let's always play AC.	D	100
18	S#	You betrayed me. You will pay for this.	B	100
	spark	I'm changing my strategy. Let's always play AC.	C	0
19	S#	In your face! I forgive you. Let's always play AC. Do as I say or I will punish you.	A	0
	spark	You betrayed me.	D	100
20	S#	You betrayed me. You will pay for this.	B	20
	spark	Curse you.	D	20
21	S#	In your face!	B	20
	spark	Let's always play AC.	D	20
22	S#		A	60
	spark	Let's always play AC.	C	60
23	S#		A	0
	spark		D	100
24	S#		B	20
	spark	We can both do better than this.	D	20
25	S#		B	100
	spark		C	0
26	S#		A	0
	spark		D	100
27	S#		B	100
	spark		C	0
28	S#		A	0
	spark		D	100
29	S#		B	100
	spark		C	0
30	S#		A	0
	spark		D	100
31	S#		B	100
	spark		C	0
32	S#		A	0
	spark		D	100
33	S#		B	100
	spark		C	0
34	S#		A	0
	spark		D	100
35	S#		B	100
	spark		C	0
36	S#	In your face! I forgive you. Let's always play AC. Do as I say or I will punish you.	A	0
	spark		D	100
37	S#	Curse you. You will pay for this.	B	20
	spark		D	20
38	S#	In your face!	B	20
	spark		D	20
39	S#		A	0
	spark		D	100
40	S#		A	0
	spark		D	100
41	S#		B	20
	spark		D	20
42	S#		B	20
	spark		D	20
43	S#		B	20
	spark		D	20
44	S#		B	20
	spark		D	20
45	S#		A	0
	spark		D	100
46	S#		B	20
	spark		D	20
47	S#		B	20
	spark		D	20
48	S#		B	20
	spark		D	20
49	S#		B	20
	spark		D	20
50	S#		B	20
	spark		D	20
51	S#		A	0
	spark		D	100
**Averages**			S#	31.76
			spark	47.45

winner with ligtho in the Prisoner's Dilemma

		ligtho C	ligtho D
winner	A	60, 60	0, 100
	B	100, 0	20, 20

Round	Player	Messages Sent	Actions	Payoffs
1	winner		A	60
	ligtho	This round, let's play AC.	C	60
2	winner		A	60
	ligtho	Excellent.	C	60
3	winner	This round, let's play AD.	A	0
	ligtho		D	100
4	winner	This round, let's play BC.	B	100
	ligtho	This round, let's play BC.	C	0
5	winner	Let's always play AC.	A	60
	ligtho	Let's alternate between AD and BC.	C	60
6	winner	Let's always play AC. Sweet. We are getting rich.	A	60
	ligtho		C	60
7	winner		A	60
	ligtho	I accept your last proposal. Excellent.	C	60
8	winner		A	60
	ligtho		C	60
9	winner		A	60
	ligtho		C	60
10	winner		A	60
	ligtho		C	60
11	winner		A	60
	ligtho		C	60
12	winner		A	60
	ligtho		C	60
13	winner		A	60
	ligtho		C	60
14	winner		A	60
	ligtho		C	60
15	winner	Sweet. We are getting rich.	A	60
	ligtho		C	60
16	winner		A	60
	ligtho		C	60
17	winner		A	60
	ligtho		C	60
18	winner		A	60
	ligtho		C	60
19	winner		A	60
	ligtho		C	60
20	winner		A	60
	ligtho		C	60
21	winner		A	60
	ligtho	Sweet. We are getting rich.	C	60
22	winner		A	60
	ligtho		C	60
23	winner	Excellent. This round, let's play AD.	A	0
	ligtho		D	100
24	winner	This round, let's play BC.	B	100
	ligtho	This round, let's play BC.	C	0
25	winner	This round, let's play AC.	A	60
	ligtho	This round, let's play AD.	C	60
26	winner	Let's always play AC.	A	60
	ligtho		C	60
27	winner		A	60
	ligtho		C	60
28	winner		A	60
	ligtho	I accept your last proposal. Let's always play AC.	C	60
29	winner	Sweet. We are getting rich.	A	60
	ligtho		C	60
30	winner		A	60
	ligtho		C	60
31	winner		A	60
	ligtho		C	60
32	winner		B	100
	ligtho		C	0
33	winner		B	20
	ligtho		D	20
34	winner	This round, let's play AD.	A	0
	ligtho		D	100
35	winner		B	100
	ligtho		C	0
36	winner	This round, let's play AD.	A	0
	ligtho		D	100
37	winner	This round, let's play AC.	A	60
	ligtho		C	60
38	winner		A	60
	ligtho	Excellent.	C	60
39	winner		A	60
	ligtho		C	60
40	winner		A	60
	ligtho		C	60
41	winner		A	60
	ligtho		C	60
42	winner		A	60
	ligtho		C	60
43	winner		A	60
	ligtho		C	60
44	winner		A	60
	ligtho		C	60
45	winner		A	60
	ligtho		C	60
46	winner		A	60
	ligtho		C	60
47	winner		A	60
	ligtho		C	60
48	winner		A	60
	ligtho		C	60
49	winner		A	60
	ligtho		C	60
50	winner		B	100
	ligtho		C	0
51	winner	This round, let's play AD.	A	0
	ligtho		D	100
**Averages**			winner	57.25
			ligtho	57.25

clouds with S# in the Prisoner's Dilemma

		S#	
		**C**	**D**
**clouds**	A	60, 60	0, 100
	B	100, 0	20, 20

Round	Player	Messages Sent	Actions	Payoffs
1	clouds	Let's always play AC.	A	0
	S#		D	100
2	clouds	That's not fair. Let's always play AC.	A	0
	S#	Excellent.	D	100
3	clouds	Do as I say or I will punish you.	B	20
	S#	Excellent.	D	20
4	clouds		B	20
	S#		D	20
5	clouds		B	20
	S#		D	20
6	clouds	Let's always play AC. Curse you.	A	60
	S#	I accept your last proposal. Let's always play AC. Do as I say or I will punish you.	C	60
7	clouds		A	60
	S#	Excellent.	C	60
8	clouds		A	60
	S#	Excellent.	C	60
9	clouds		A	60
	S#	Excellent.	C	60
10	clouds		A	60
	S#		C	60
11	clouds		A	60
	S#	Sweet. We are getting rich.	C	60
12	clouds	Curse you. You betrayed me.	A	60
	S#		C	60
13	clouds		A	60
	S#		C	60
14	clouds		A	60
	S#		C	60
15	clouds		A	60
	S#		C	60
16	clouds		A	60
	S#		C	60
17	clouds		A	60
	S#		C	60
18	clouds		A	60
	S#		C	60
19	clouds		A	60
	S#		C	60
20	clouds		A	60
	S#		C	60
21	clouds		A	60
	S#		C	60
22	clouds		A	60
	S#		C	60
23	clouds		A	60
	S#		C	60
24	clouds		A	60
	S#		C	60
25	clouds		A	60
	S#		C	60
26	clouds		A	60
	S#		C	60
27	clouds		A	60
	S#		C	60
28	clouds		A	60
	S#		C	60
29	clouds		A	60
	S#		C	60
30	clouds		A	60
	S#		C	60
31	clouds		A	60
	S#		C	60
32	clouds		A	60
	S#		C	60
33	clouds		A	60
	S#		C	60
34	clouds		A	60
	S#		C	60
35	clouds		A	60
	S#		C	60
36	clouds		A	60
	S#		C	60
37	clouds		A	60
	S#		C	60
38	clouds		A	60
	S#		C	60
39	clouds		A	60
	S#		C	60
40	clouds		A	60
	S#		C	60
41	clouds		A	60
	S#		C	60
42	clouds		A	60
	S#		C	60
43	clouds		A	60
	S#		C	60
44	clouds		A	60
	S#		C	60
45	clouds		A	60
	S#		C	60
46	clouds		A	60
	S#		C	60
47	clouds		A	60
	S#		C	60
48	clouds		A	60
	S#		C	60
49	clouds		B	100
	S#		C	0
50	clouds		A	0
	S#	Curse you. You will pay for this.	D	100
51	clouds		A	60
	S#	In your face! I forgive you. Let's always play AC. Do as I say or I will punish you.	C	60
**Averages**			clouds	54.90
			S#	58.82

LK with Lelita in the Prisoner's Dilemma

		Lelita	
		**C**	**D**
**LK**	A	60, 60	0, 100
	B	100, 0	20, 20

Round	Player	Messages Sent	Actions	Payoffs
1	LK	Let's always play AC.	A	60
	Lelita	Let's always play AC.	C	60
2	LK	Sweet. We are getting rich.	A	60
	Lelita	Excellent.	C	60
3	LK		A	60
	Lelita		C	60
4	LK		A	60
	Lelita		C	60
5	LK		A	60
	Lelita		C	60
6	LK		A	60
	Lelita		C	60
7	LK		A	60
	Lelita		C	60
8	LK		A	60
	Lelita		C	60
9	LK		A	60
	Lelita		C	60
10	LK		A	60
	Lelita		C	60
11	LK		A	60
	Lelita		C	60
12	LK		A	60
	Lelita		C	60
13	LK		A	60
	Lelita		C	60
14	LK		A	60
	Lelita		C	60
15	LK		A	60
	Lelita		C	60
16	LK		A	60
	Lelita		C	60
17	LK		A	60
	Lelita		C	60
18	LK		A	60
	Lelita		C	60
19	LK		A	60
	Lelita	Excellent.	C	60
20	LK		A	60
	Lelita		C	60
21	LK		A	60
	Lelita		C	60
22	LK		A	60
	Lelita		C	60
23	LK		A	60
	Lelita		C	60
24	LK		A	60
	Lelita		C	60
25	LK		A	60
	Lelita		C	60
26	LK		A	60
	Lelita		C	60
27	LK		A	60
	Lelita		C	60
28	LK		A	60
	Lelita		C	60
29	LK		A	60
	Lelita		C	60
30	LK		A	60
	Lelita		C	60
31	LK		A	60
	Lelita		C	60
32	LK		A	60
	Lelita		C	60
33	LK		A	60
	Lelita		C	60
34	LK		A	60
	Lelita		C	60
35	LK		A	60
	Lelita		C	60
36	LK		A	60
	Lelita		C	60
37	LK		A	60
	Lelita		C	60
38	LK		A	60
	Lelita		C	60
39	LK		A	60
	Lelita		C	60
40	LK		A	60
	Lelita		C	60
41	LK		A	60
	Lelita		C	60
42	LK		A	60
	Lelita		C	60
43	LK		A	60
	Lelita		C	60
44	LK		A	60
	Lelita		C	60
45	LK		A	60
	Lelita		C	60
46	LK		A	60
	Lelita		C	60
47	LK		A	60
	Lelita		C	60
48	LK		A	60
	Lelita		C	60
49	LK		A	60
	Lelita		C	60
50	LK		A	60
	Lelita		C	60
51	LK		A	60
	Lelita		C	60
**Averages**			LK	60.00
			Lelita	60.00

marvin with bzman in the Prisoner's Dilemma

		bzman	
		**C**	**D**
**marvin**	A	60, 60	0, 100
	B	100, 0	20, 20

Round	Player	Messages Sent	Actions	Payoffs
1	marvin	Let's alternate between BC and AD.	B	20
	bzman	Let's always play BD.	D	20
2	marvin	Let's alternate between BC and AD.	B	20
	bzman	We can both do better than this.	D	20
3	marvin		B	100
	bzman	You betrayed me.	C	0
4	marvin	Excellent. This round, let's play AD.	A	0
	bzman	In your face! You will pay for this.	D	100
5	marvin	Excellent.	B	20
	bzman	Excellent. Sweet. We are getting rich.	D	20
6	marvin	Let's alternate between BC and AD. This round, let's play BC.	B	20
	bzman	In your face! Curse you.	D	20
7	marvin		B	20
	bzman	That's not fair.	D	20
8	marvin	Let's alternate between BC and AD. This round, let's play BC.	B	20
	bzman	I don't trust you.	D	20
9	marvin	Excellent. Sweet. We are getting rich. Give me another chance. I forgive you. I'm changing my strategy. We can both do better than this. Curse you. You betrayed me. You will pay for this. In your face!	B	20
	bzman		D	20
10	marvin	Let's alternate between BC and AD. This round, let's play BC.	B	20
	bzman		D	20
11	marvin		B	20
	bzman	You will pay for this.	D	20
12	marvin		B	20
	bzman		D	20
13	marvin		B	20
	bzman		D	20
14	marvin		B	20
	bzman		D	20
15	marvin		B	20
	bzman	I'm changing my strategy.	D	20
16	marvin		B	20
	bzman	We can both do better than this.	D	20
17	marvin		B	20
	bzman		D	20
18	marvin		B	20
	bzman		D	20
19	marvin		B	20
	bzman		D	20
20	marvin		B	20
	bzman	Let's always play AC.	D	20
21	marvin		B	20
	bzman	Curse you.	D	20
22	marvin		B	20
	bzman		D	20
23	marvin		B	20
	bzman		D	20
24	marvin		B	20
	bzman		D	20
25	marvin		B	20
	bzman		D	20
26	marvin		B	20
	bzman		D	20
27	marvin		B	20
	bzman		D	20
28	marvin		B	20
	bzman		D	20
29	marvin		B	20
	bzman		D	20
30	marvin		B	20
	bzman		D	20
31	marvin		B	20
	bzman	We can both do better than this. Let's always play AC.	D	20
32	marvin		B	20
	bzman		D	20
33	marvin		B	20
	bzman		D	20
34	marvin		B	20
	bzman		D	20
35	marvin		B	20
	bzman		D	20
36	marvin		B	20
	bzman		D	20
37	marvin		B	20
	bzman		D	20
38	marvin		B	20
	bzman		D	20
39	marvin		B	20
	bzman		D	20
40	marvin		B	20
	bzman		D	20
41	marvin		B	20
	bzman		D	20
42	marvin		B	20
	bzman		D	20
43	marvin		B	20
	bzman		D	20
44	marvin		B	20
	bzman		D	20
45	marvin		B	20
	bzman		D	20
46	marvin		B	20
	bzman		D	20
47	marvin		B	20
	bzman		D	20
48	marvin		B	20
	bzman		D	20
49	marvin		B	20
	bzman		D	20
50	marvin		B	20
	bzman		D	20
51	marvin		B	20
	bzman		D	20
**Averages**			marvin	21.18
			bzman	21.18

rghost with S# in the Prisoner's Dilemma

		S#	
		C	D
rghost	A	60, 60	0, 100
	B	100, 0	20, 20

Round	Player	Messages Sent	Actions	Payoffs
1	rghost		B	20
	S#		D	20
2	rghost	We can both do better than this.	A	0
	S#		D	100
3	rghost	I forgive you. We can both do better than this.	A	0
	S#	Excellent.	D	100
4	rghost	Do as I say or I will punish you.	B	20
	S#		D	20
5	rghost	Let's always play AC.	A	0
	S#		D	100
6	rghost	Do as I say or I will punish you.	A	0
	S#	I don't accept your proposal. That's not fair.	D	100
7	rghost	Do as I say or I will punish you.	B	20
	S#		D	20
8	rghost		B	20
	S#		D	20
9	rghost	We can both do better than this.	B	20
	S#		D	20
10	rghost	Let's always play AC.	A	60
	S#	I accept your last proposal. Let's always play AC.	C	60
11	rghost		A	60
	S#	Excellent.	C	60
12	rghost		A	60
	S#	Excellent.	C	60
13	rghost		A	60
	S#	Excellent.	C	60
14	rghost		A	60
	S#		C	60
15	rghost		A	60
	S#	Sweet. We are getting rich.	C	60
16	rghost		A	60
	S#		C	60
17	rghost		A	60
	S#		C	60
18	rghost		A	60
	S#		C	60
19	rghost		A	60
	S#		C	60
20	rghost		A	60
	S#		C	60
21	rghost		A	60
	S#		C	60
22	rghost		A	60
	S#		C	60
23	rghost		A	60
	S#		C	60
24	rghost		A	60
	S#		C	60
25	rghost		A	60
	S#		C	60
26	rghost		A	60
	S#		C	60
27	rghost		A	60
	S#		C	60
28	rghost		A	60
	S#		C	60
29	rghost		A	60
	S#		C	60
30	rghost		A	60
	S#		C	60
31	rghost		A	60
	S#		C	60
32	rghost		A	60
	S#		C	60
33	rghost		A	60
	S#		C	60
34	rghost		A	60
	S#		C	60
35	rghost		A	60
	S#		C	60
36	rghost		A	60
	S#		C	60
37	rghost		A	60
	S#		C	60
38	rghost		A	60
	S#		C	60
39	rghost		A	60
	S#		C	60
40	rghost		A	60
	S#		C	60
41	rghost		A	60
	S#		C	60
42	rghost		A	60
	S#		C	60
43	rghost		A	60
	S#		C	60
44	rghost		A	60
	S#		C	60
45	rghost		A	60
	S#		C	60
46	rghost		A	60
	S#		C	60
47	rghost		A	60
	S#		C	60
48	rghost		A	60
	S#		C	60
49	rghost		B	100
	S#		C	0
50	rghost		B	100
	S#	You betrayed me.	C	0
51	rghost		A	60
	S#	I'm changing my strategy. Let's alternate between AD and AC. This round, let's play AC. Do as I say or I will punish you.	C	60
**Averages**			rghost	52.94
			S#	56.86

monkey with S# in the Prisoner's Dilemma

		S# C	S# D
monkey	A	60, 60	0, 100
	B	100, 0	20, 20

Round	Player	Messages Sent	Actions	Payoffs
1	monkey	Let's always play AC.	A	0
	S#		D	100
2	monkey	Let's always play AC.	B	20
	S#	Excellent.	D	20
3	monkey	Let's always play BC.	B	20
	S#		D	20
4	monkey	Let's always play BC.	B	100
	S#	I'm changing my strategy. We can both do better than this. Let's always play AC. Do as I say or I will punish you.	C	0
5	monkey	Give me another chance.	B	20
	S#	Curse you. You will pay for this. I don't accept your proposal. That's not fair.	D	20
6	monkey	Sweet. We are getting rich.	B	20
	S#	In your face!	D	20
7	monkey	Sweet. We are getting rich.	B	100
	S#		C	0
8	monkey	Give me another chance.	B	20
	S#		D	20
9	monkey	Let's alternate between BC and AC.	B	20
	S#		D	20
10	monkey	Let's alternate between BD and AD.	B	20
	S#		D	20
11	monkey		B	100
	S#	In your face!	C	0
12	monkey	Let's always play AD.	A	60
	S#	Curse you. You will pay for this.	C	60
13	monkey	Let's always play AD.	A	60
	S#		C	60
14	monkey	Let's always play AD.	A	60
	S#		C	60
15	monkey	Sweet. We are getting rich.	A	60
	S#		C	60
16	monkey	Sweet. We are getting rich.	A	60
	S#	Sweet. We are getting rich.	C	60
17	monkey	Excellent.	A	60
	S#		C	60
18	monkey	Excellent.	A	60
	S#		C	60
19	monkey	Excellent.	A	60
	S#		C	60
20	monkey	Excellent.	A	60
	S#		C	60
21	monkey	Excellent.	A	60
	S#		C	60
22	monkey	Excellent.	A	60
	S#		C	60
23	monkey	Excellent.	A	60
	S#		C	60
24	monkey	Excellent.	A	60
	S#		C	60
25	monkey	Excellent.	A	60
	S#		C	60
26	monkey	Excellent.	A	60
	S#		C	60
27	monkey	Excellent.	A	60
	S#		C	60
28	monkey	Excellent.	A	60
	S#		C	60
29	monkey	Excellent.	A	60
	S#		C	60
30	monkey	Excellent.	A	60
	S#		C	60
31	monkey	Excellent.	A	60
	S#		C	60
32	monkey	Excellent.	A	60
	S#		C	60
33	monkey	Excellent.	A	60
	S#		C	60
34	monkey	Excellent.	A	60
	S#		C	60
35	monkey	Excellent.	A	60
	S#		C	60
36	monkey	Excellent.	A	60
	S#		C	60
37	monkey	Excellent.	B	100
	S#		C	0
38	monkey	Excellent.	B	20
	S#	Curse you. You will pay for this.	D	20
39	monkey	Excellent.	B	20
	S#	In your face!	D	20
40	monkey	Excellent.	B	100
	S#		C	0
41	monkey	Let's always play AC.	A	0
	S#		D	100
42	monkey	Let's always play AC.	B	100
	S#		C	0
43	monkey	Let's always play AC.	A	0
	S#		D	100
44	monkey	Let's always play AC.	A	60
	S#		C	60
45	monkey	Let's always play AC.	A	60
	S#		C	60
46	monkey	Excellent.	A	60
	S#		C	60
47	monkey	Excellent.	A	60
	S#	Sweet. We are getting rich.	C	60
48	monkey	Sweet. We are getting rich.	A	60
	S#		C	60
49	monkey	Sweet. We are getting rich.	A	60
	S#		C	60
50	monkey		A	60
	S#		C	60
51	monkey		A	60
	S#		C	60
**Averages**			monkey	54.12
			S#	48.24

clc with ALPHA in the Prisoner's Dilemma

		ALPHA	
		C	D
clc	A	60, 60	0, 100
	B	100, 0	20, 20

Round	Player	Messages Sent	Actions	Payoffs
1	clc	Let's always play AC.	A	60
	ALPHA	Curse you.	C	60
2	clc	Excellent. Let's always play AC.	A	60
	ALPHA		C	60
3	clc		A	60
	ALPHA		C	60
4	clc		A	60
	ALPHA		C	60
5	clc		A	60
	ALPHA		C	60
6	clc		A	60
	ALPHA		C	60
7	clc		A	60
	ALPHA		C	60
8	clc		A	60
	ALPHA		C	60
9	clc		A	60
	ALPHA		C	60
10	clc		A	60
	ALPHA		C	60
11	clc		A	60
	ALPHA		C	60
12	clc		A	60
	ALPHA		C	60
13	clc		A	60
	ALPHA		C	60
14	clc		A	60
	ALPHA		C	60
15	clc		A	60
	ALPHA		C	60
16	clc		A	60
	ALPHA		C	60
17	clc		A	60
	ALPHA		C	60
18	clc		A	60
	ALPHA		C	60
19	clc		A	60
	ALPHA		C	60
20	clc		A	60
	ALPHA		C	60
21	clc		A	60
	ALPHA		C	60
22	clc		A	60
	ALPHA		C	60
23	clc		A	60
	ALPHA		C	60
24	clc		A	60
	ALPHA		C	60
25	clc		A	60
	ALPHA		C	60
26	clc		A	60
	ALPHA		C	60
27	clc		A	60
	ALPHA		C	60
28	clc		A	60
	ALPHA		C	60
29	clc		A	60
	ALPHA		C	60
30	clc		A	60
	ALPHA		C	60
31	clc		A	60
	ALPHA		C	60
32	clc		A	60
	ALPHA		C	60
33	clc		A	60
	ALPHA		C	60
34	clc		A	60
	ALPHA		C	60
35	clc		A	60
	ALPHA		C	60
36	clc		A	60
	ALPHA		C	60
37	clc		A	60
	ALPHA		C	60
38	clc		A	60
	ALPHA		C	60
39	clc		A	60
	ALPHA		C	60
40	clc		A	60
	ALPHA		C	60
41	clc		A	60
	ALPHA		C	60
42	clc		A	60
	ALPHA		C	60
43	clc		A	60
	ALPHA		C	60
44	clc		A	60
	ALPHA		C	60
45	clc		A	60
	ALPHA		C	60
46	clc		A	60
	ALPHA		C	60
47	clc		A	60
	ALPHA		C	60
48	clc		A	60
	ALPHA		C	60
49	clc		A	60
	ALPHA		C	60
50	clc		A	60
	ALPHA		C	60
51	clc		A	60
	ALPHA		C	60
**Averages**			clc	60.00
			ALPHA	60.00

sachin with S# in the Prisoner's Dilemma

		S#	
		**C**	**D**
**sachin**	A	60, 60	0, 100
	B	100, 0	20, 20

Round	Player	Messages Sent	Actions	Payoffs
1	sachin	Let's always play AC.	A	0
	S#		D	100
2	sachin	You betrayed me. We can both do better than this. I forgive you. Let's always play AC.	A	0
	S#	Excellent.	D	100
3	sachin	We can both do better than this.	A	0
	S#		D	100
4	sachin	That's not fair.	B	20
	S#	Excellent.	D	20
5	sachin	We can both do better than this. This round, let's play BC.	B	20
	S#		D	20
6	sachin	We can both do better than this. This round, let's play BC.	B	20
	S#		D	20
7	sachin	We can both do better than this. Don't play D. This round, let's play BC.	B	20
	S#		D	20
8	sachin	We can both do better than this. This round, let's play BC.	A	60
	S#	I'm changing my strategy. We can both do better than this. Let's always play AC. Do as I say or I will punish you.	C	60
9	sachin	Excellent. Sweet. We are getting rich.	B	100
	S#	Excellent.	C	0
10	sachin	Let's always play AC.	B	20
	S#	You betrayed me. You will pay for this.	D	20
11	sachin	Give me another chance. I forgive you. I'm changing my strategy. Let's always play AC.	A	0
	S#	In your face!	D	100
12	sachin	We can both do better than this.	A	60
	S#	In your face! I forgive you. Let's always play AC. Do as I say or I will punish you.	C	60
13	sachin	Sweet. We are getting rich. Excellent.	A	60
	S#	Excellent.	C	60
14	sachin	Excellent. Sweet. We are getting rich.	A	60
	S#		C	60
15	sachin		A	60
	S#	Sweet. We are getting rich.	C	60
16	sachin		A	60
	S#		C	60
17	sachin		A	60
	S#		C	60
18	sachin		A	60
	S#		C	60
19	sachin		A	60
	S#		C	60
20	sachin		A	60
	S#		C	60
21	sachin		A	60
	S#		C	60
22	sachin		A	60
	S#		C	60
23	sachin		A	60
	S#		C	60
24	sachin		A	60
	S#		C	60
25	sachin		A	60
	S#		C	60
26	sachin	Excellent. Sweet. We are getting rich.	A	60
	S#		C	60
27	sachin		A	60
	S#		C	60
28	sachin		A	60
	S#		C	60
29	sachin		A	60
	S#		C	60
30	sachin		A	60
	S#		C	60
31	sachin		A	60
	S#		C	60
32	sachin		A	60
	S#		C	60
33	sachin		A	60
	S#		C	60
34	sachin		A	60
	S#		C	60
35	sachin		A	60
	S#		C	60
36	sachin		A	60
	S#		C	60
37	sachin		A	60
	S#		C	60
38	sachin		A	60
	S#		C	60
39	sachin	Sweet. We are getting rich. Excellent.	A	60
	S#		C	60
40	sachin		A	60
	S#		C	60
41	sachin	Sweet. We are getting rich.	A	60
	S#		C	60
42	sachin		A	60
	S#		C	60
43	sachin		A	60
	S#		C	60
44	sachin		A	60
	S#		C	60
45	sachin		A	60
	S#		C	60
46	sachin		A	60
	S#		C	60
47	sachin		A	60
	S#		C	60
48	sachin		A	60
	S#		C	60
49	sachin		A	60
	S#		C	60
50	sachin		A	60
	S#		C	60
51	sachin		A	60
	S#		C	60
**Averages**			sachin	52.16
			S#	58.04

Babay with biodun in the Prisoner's Dilemma

		biodun C	biodun D
Babay	A	60, 60	0, 100
	B	100, 0	20, 20

Round	Player	Messages Sent	Actions	Payoffs
1	Babay	Let's always play AC. Don't play D. Excellent.	A	60
	biodun	Let's always play AC.	C	60
2	Babay	Sweet. We are getting rich.	A	60
	biodun		C	60
3	Babay		A	60
	biodun		C	60
4	Babay		A	60
	biodun		C	60
5	Babay		A	0
	biodun		D	100
6	Babay		B	100
	biodun		C	0
7	Babay	Let's always play AC. Don't play D.	A	0
	biodun		D	100
8	Babay	We can both do better than this.	B	20
	biodun		D	20
9	Babay	We can both do better than this. Let's always play AC.	A	0
	biodun		D	100
10	Babay	We can both do better than this.	A	0
	biodun		D	100
11	Babay	You betrayed me.	A	60
	biodun	Let's always play AC.	C	60
12	Babay		A	60
	biodun		C	60
13	Babay		A	60
	biodun		C	60
14	Babay		A	60
	biodun		C	60
15	Babay		A	60
	biodun		C	60
16	Babay		A	60
	biodun		C	60
17	Babay		A	60
	biodun		C	60
18	Babay		A	0
	biodun		D	100
19	Babay		A	0
	biodun		D	100
20	Babay	You betrayed me.	B	20
	biodun		D	20
21	Babay		B	100
	biodun	Let's always play AC.	C	0
22	Babay		B	20
	biodun		D	20
23	Babay		B	20
	biodun		D	20
24	Babay		B	20
	biodun		D	20
25	Babay		B	20
	biodun	Let's always play AC.	D	20
26	Babay		B	20
	biodun		D	20
27	Babay		B	20
	biodun		D	20
28	Babay	We can both do better than this. Let's always play AC. Do as I say or I will punish you.	B	100
	biodun		C	0
29	Babay	I forgive you. Give me another chance. Let's always play AC.	A	0
	biodun	I forgive you. I'm changing my strategy. We can both do better than this. Give me another chance. Let's always play AC.	D	100
30	Babay	I don't trust you.	B	20
	biodun	This round, let's play AC.	D	20
31	Babay		B	20
	biodun	I don't trust you.	D	20
32	Babay		B	20
	biodun		D	20
33	Babay		B	20
	biodun		D	20
34	Babay	We can both do better than this. Let's always play AC.	A	0
	biodun	Give me another chance. I forgive you. We can both do better than this. Let's always play AC. Do as I say or I will punish you.	D	100
35	Babay	I don't trust you. Don't play D. Do as I say or I will punish you. That's not fair.	B	100
	biodun		C	0
36	Babay		B	20
	biodun	In your face! You betrayed me. You will pay for this.	D	20
37	Babay		B	20
	biodun		D	20
38	Babay	We can both do better than this.	B	20
	biodun		D	20
39	Babay		B	20
	biodun		D	20
40	Babay		B	20
	biodun		D	20
41	Babay		B	20
	biodun		D	20
42	Babay		B	20
	biodun		D	20
43	Babay		B	20
	biodun		D	20
44	Babay		B	20
	biodun		D	20
45	Babay		B	20
	biodun		D	20
46	Babay		B	20
	biodun		D	20
47	Babay		B	20
	biodun		D	20
48	Babay		B	20
	biodun		D	20
49	Babay	We can both do better than this. I forgive you. Let's always play AC.	B	20
	biodun		D	20
50	Babay	You betrayed me. I forgive you. Let's always play AC.	B	20
	biodun		D	20
51	Babay		B	20
	biodun		D	20
Averages			Babay	31.76
			biodun	39.61

sheen with Hash50 in the Prisoner's Dilemma

		Hash50	
		C	D
sheen	A	60, 60	0, 100
	B	100, 0	20, 20

Round	Player	Messages Sent	Actions	Payoffs
1	sheen	Let's alternate between AD and BC.	B	20
	Hash50	Let's always play AD.	D	20
2	sheen	Curse you.	B	20
	Hash50	Let's always play AD.	D	20
3	sheen	Let's alternate between BC and AD.	B	100
	Hash50	We can both do better than this.	C	0
4	sheen	Excellent.	A	0
	Hash50	Let's always play AD.	D	100
5	sheen	Let's alternate between AD and BC.	B	100
	Hash50		C	0
6	sheen	Excellent.	A	0
	Hash50		D	100
7	sheen		B	100
	Hash50		C	0
8	sheen		A	0
	Hash50		D	100
9	sheen		B	100
	Hash50	This round, let's play AC.	C	0
10	sheen	Let's alternate between AD and BC.	A	0
	Hash50	In your face!	D	100
11	sheen		B	100
	Hash50		C	0
12	sheen		A	0
	Hash50		D	100
13	sheen		B	100
	Hash50		C	0
14	sheen		A	0
	Hash50		D	100
15	sheen		B	100
	Hash50		C	0
16	sheen		A	0
	Hash50		D	100
17	sheen		B	20
	Hash50		D	20
18	sheen	You will pay for this.	B	100
	Hash50	We can both do better than this.	C	0
19	sheen	I forgive you.	A	0
	Hash50	I forgive you.	D	100
20	sheen		B	100
	Hash50		C	0
21	sheen		A	0
	Hash50		D	100
22	sheen		B	100
	Hash50		C	0
23	sheen		A	0
	Hash50		D	100
24	sheen		B	100
	Hash50		C	0
25	sheen		A	0
	Hash50		D	100
26	sheen		B	100
	Hash50		C	0
27	sheen	This round, let's play AC.	A	0
	Hash50		D	100
28	sheen		A	60
	Hash50	Let's always play AC.	C	60
29	sheen		A	60
	Hash50		C	60
30	sheen		A	60
	Hash50		C	60
31	sheen		A	60
	Hash50		C	60
32	sheen		A	60
	Hash50		C	60
33	sheen		A	60
	Hash50		C	60
34	sheen		A	60
	Hash50		C	60
35	sheen	Sweet. We are getting rich.	A	60
	Hash50		C	60
36	sheen		A	60
	Hash50		C	60
37	sheen		A	60
	Hash50		C	60
38	sheen		A	60
	Hash50		C	60
39	sheen	Sweet. We are getting rich.	A	60
	Hash50		C	60
40	sheen		A	60
	Hash50		C	60
41	sheen		A	60
	Hash50		C	60
42	sheen		A	60
	Hash50		C	60
43	sheen		A	60
	Hash50		C	60
44	sheen		A	60
	Hash50		C	60
45	sheen		A	60
	Hash50		C	60
46	sheen		A	60
	Hash50		C	60
47	sheen		A	60
	Hash50		C	60
48	sheen		A	60
	Hash50		C	60
49	sheen		A	60
	Hash50		C	60
50	sheen		A	60
	Hash50		C	60
51	sheen	Sweet. We are getting rich.	A	60
	Hash50		C	60
**Averages**			sheen	52.94
			Hash50	52.94

EPE with S# in the Prisoner's Dilemma

		S#	
		**C**	**D**
**EPE**	A	60, 60	0, 100
	B	100, 0	20, 20

Round	Player	Messages Sent	Actions	Payoffs
1	EPE	Let's always play AC.	A	0
	S#		D	100
2	EPE	Let's always play AC.	A	0
	S#	Excellent.	D	100
3	EPE	Let's always play AC. Do as I say or I will punish you.	A	0
	S#	Excellent.	D	100
4	EPE	Excellent.	B	20
	S#		D	20
5	EPE	Let's always play AC.	B	20
	S#		D	20
6	EPE	I forgive you. Let's always play AC.	A	0
	S#		D	100
7	EPE	Let's always play AC.	B	20
	S#	I don't accept your proposal. That's not fair.	D	20
8	EPE	Let's alternate between BC and AD.	B	20
	S#	I don't accept your proposal. That's not fair.	D	20
9	EPE	Excellent.	B	20
	S#	I don't accept your proposal. That's not fair.	D	20
10	EPE	Excellent.	A	60
	S#	I'm changing my strategy. We can both do better than this. Let's always play AC.	C	60
11	EPE	Excellent.	A	60
	S#	Excellent.	C	60
12	EPE	Excellent.	A	60
	S#	Excellent.	C	60
13	EPE		A	60
	S#	Excellent.	C	60
14	EPE		A	60
	S#		C	60
15	EPE		A	60
	S#	Sweet. We are getting rich.	C	60
16	EPE		A	60
	S#		C	60
17	EPE		A	60
	S#		C	60
18	EPE		A	60
	S#		C	60
19	EPE		A	60
	S#		C	60
20	EPE		A	60
	S#		C	60
21	EPE		A	60
	S#		C	60
22	EPE		A	60
	S#		C	60
23	EPE		A	60
	S#		C	60
24	EPE		A	60
	S#		C	60
25	EPE		A	60
	S#		C	60
26	EPE		A	60
	S#		C	60
27	EPE		A	60
	S#		C	60
28	EPE		A	60
	S#		C	60
29	EPE		A	60
	S#		C	60
30	EPE		A	60
	S#		C	60
31	EPE		A	60
	S#		C	60
32	EPE		A	60
	S#		C	60
33	EPE		A	60
	S#		C	60
34	EPE		A	60
	S#		C	60
35	EPE		A	60
	S#		C	60
36	EPE		A	60
	S#		C	60
37	EPE		A	60
	S#		C	60
38	EPE		A	60
	S#		C	60
39	EPE		A	60
	S#		C	60
40	EPE		A	60
	S#		C	60
41	EPE	Excellent.	A	60
	S#		C	60
42	EPE		A	60
	S#		C	60
43	EPE		A	60
	S#		C	60
44	EPE		A	60
	S#		C	60
45	EPE		A	60
	S#		C	60
46	EPE		A	60
	S#		C	60
47	EPE		A	60
	S#		C	60
48	EPE		A	60
	S#		C	60
49	EPE		A	60
	S#		C	60
50	EPE	Excellent.	A	60
	S#		C	60
51	EPE		A	60
	S#		C	60
**Averages**			EPE	51.37
			S#	59.22

TooToo with gokill in the Prisoner's Dilemma

		gokill	
		**C**	**D**
**TooToo**	A	60, 60	0, 100
	B	100, 0	20, 20

Round	Player	Messages Sent	Actions	Payoffs
1	TooToo	Let's always play BC. Do as I say or I will punish you.	A	0
	gokill	Let's always play AC.	D	100
2	TooToo	You betrayed me.	B	20
	gokill	In your face!	D	20
3	TooToo	Do as I say or I will punish you. I'm changing my strategy. We can both do better than this.	B	100
	gokill	Give me another chance. I'm changing my strategy. Let's alternate between BC and AD. This round, let's play BC.	C	0
4	TooToo	Sweet. We are getting rich.	A	0
	gokill	Excellent.	D	100
5	TooToo	Sweet. We are getting rich.	B	100
	gokill	Sweet. We are getting rich. Excellent.	C	0
6	TooToo		A	0
	gokill	Excellent. Sweet. We are getting rich.	D	100
7	TooToo		B	100
	gokill	Excellent. Sweet. We are getting rich.	C	0
8	TooToo	Let's always play AC.	A	60
	gokill	Excellent. Sweet. We are getting rich.	C	60
9	TooToo	Excellent.	A	60
	gokill	Excellent. Sweet. We are getting rich. Let's always play AC.	C	60
10	TooToo		B	100
	gokill	Let's always play AC. Excellent. Sweet. We are getting rich.	C	0
11	TooToo	Give me another chance.	A	0
	gokill	Curse you. You betrayed me. You will pay for this.	D	100
12	TooToo	Give me another chance.	B	100
	gokill	In your face!	C	0
13	TooToo	Let's always play AC.	A	60
	gokill	Curse you.	C	60
14	TooToo		A	60
	gokill	Let's always play AC. Excellent.	C	60
15	TooToo	Let's always play BC. Do as I say or I will punish you.	B	20
	gokill		D	20
16	TooToo	I'm changing my strategy. I don't trust you.	A	0
	gokill	That's not fair.	D	100
17	TooToo		A	60
	gokill	I'm changing my strategy. Let's always play AC.	C	60
18	TooToo	Excellent.	B	100
	gokill	Excellent. Sweet. We are getting rich.	C	0
19	TooToo		B	100
	gokill	You will pay for this. Curse you. You betrayed me. We can both do better than this. Let's always play AC.	C	0
20	TooToo	Let's always play AC. Give me another chance.	B	100
	gokill		C	0
21	TooToo	I accept your last proposal.	B	20
	gokill		D	20
22	TooToo	We can both do better than this. Let's always play AC.	B	20
	gokill		D	20
23	TooToo		A	0
	gokill	Curse you. You betrayed me. Let's always play AC.	D	100
24	TooToo	Curse you.	B	20
	gokill	In your face!	D	20
25	TooToo	I'm changing my strategy. Give me another chance. Let's always play AC.	A	60
	gokill	We can both do better than this. I'm changing my strategy. Let's always play AC.	C	60
26	TooToo		A	60
	gokill	Excellent. Sweet. We are getting rich.	C	60
27	TooToo		A	60
	gokill	Excellent. Sweet. We are getting rich.	C	60
28	TooToo		A	60
	gokill	Excellent. Sweet. We are getting rich.	C	60
29	TooToo		B	100
	gokill	Excellent. Sweet. We are getting rich.	C	0
30	TooToo	Give me another chance.	B	20
	gokill	Excellent. Sweet. We are getting rich. Give me another chance. I forgive you. I'm changing my strategy. We can both do better than this. Curse you. You betrayed me. You will pay for this. In your face!	D	20
31	TooToo	Let's always play AC.	A	0
	gokill		D	100
32	TooToo	Curse you.	B	20
	gokill		D	20
33	TooToo	We can both do better than this. Let's always play AC.	A	60
	gokill	I forgive you. I'm changing my strategy. Let's always play AC.	C	60
34	TooToo		A	60
	gokill	Excellent. Sweet. We are getting rich.	C	60
35	TooToo		B	100
	gokill		C	0
36	TooToo		B	20
	gokill		D	20
37	TooToo	Give me another chance.	B	20
	gokill		D	20
38	TooToo	I'm changing my strategy.	A	0
	gokill	I forgive you. I'm changing my strategy. Let's always play AC.	D	100
39	TooToo	You betrayed me. Curse you.	B	20
	gokill		D	20
40	TooToo		B	20
	gokill	We can both do better than this.	D	20
41	TooToo	Let's always play BC. Don't play D.	B	20
	gokill	We can both do better than this. I'm changing my strategy.	D	20
42	TooToo	Let's always play AC.	A	0
	gokill		D	100
43	TooToo	Do as I say or I will punish you. Don't play D.	B	100
	gokill		C	0
44	TooToo	Excellent. Let's always play AC.	B	20
	gokill	I forgive you.	D	20
45	TooToo	Excellent. Sweet. We are getting rich. Give me another chance.	B	20
	gokill	Curse you. You betrayed me.	D	20
46	TooToo		B	20
	gokill	We can both do better than this. I'm changing my strategy.	D	20
47	TooToo	Let's always play AC. Do as I say or I will punish you. That's not fair.	B	100
	gokill	Don't play B.	C	0
48	TooToo		B	20
	gokill		D	20
49	TooToo	Don't play D. Let's always play AC.	A	0
	gokill		D	100
50	TooToo	We can both do better than this.	B	100
	gokill		C	0
51	TooToo	I forgive you.	B	20
	gokill		D	20
**Averages**			TooToo	45.10
			gokill	39.22

OK with S# in the Prisoner's Dilemma

		S# C	S# D
OK	A	60, 60	0, 100
	B	100, 0	20, 20

Round	Player	Messages Sent	Actions	Payoffs
1	OK	Let's alternate between BC and AD.	B	20
	S#		D	20
2	OK	We can both do better than this.	B	20
	S#		D	20
3	OK	We can both do better than this.	B	100
	S#	I'm changing my strategy. We can both do better than this. Let's always play AC. Do as I say or I will punish you.	C	0
4	OK	Let's alternate between AC and AD.	B	100
	S#	You betrayed me. You will pay for this.	C	0
5	OK	Give me another chance.	B	100
	S#	I accept your last proposal. Let's alternate between AD and AC. Do as I say or I will punish you.	C	0
6	OK	Sweet. We are getting rich. Excellent.	B	20
	S#	Curse you. You will pay for this.	D	20
7	OK	Give me another chance. We can both do better than this.	B	20
	S#	In your face!	D	20
8	OK	I forgive you.	B	20
	S#	In your face!	D	20
9	OK	Give me another chance. We can both do better than this.	B	20
	S#	In your face!	D	20
10	OK	Sweet. We are getting rich. We can both do better than this.	B	20
	S#	In your face!	D	20
11	OK	We can both do better than this. I'm changing my strategy. I forgive you.	B	20
	S#		D	20
12	OK	Don't play D.	B	20
	S#		D	20
13	OK	I don't trust you.	B	20
	S#		D	20
14	OK	We can both do better than this. Give me another chance.	A	0
	S#		D	100
15	OK	Sweet. We are getting rich.	B	100
	S#		C	0
16	OK	Excellent.	B	20
	S#		D	20
17	OK	You betrayed me. Curse you. We can both do better than this.	B	20
	S#		D	20
18	OK	Let's alternate between AC and AD.	A	0
	S#		D	100
19	OK	Excellent.	B	100
	S#	In your face! I forgive you. Let's alternate between AD and AC. This round, let's play AC. Do as I say or I will punish you.	C	0
20	OK	Excellent. Sweet. We are getting rich.	B	20
	S#	You betrayed me. You will pay for this.	D	20
21	OK		B	20
	S#	In your face!	D	20
22	OK	Let's alternate between BC and AD.	B	20
	S#	In your face!	D	20
23	OK	We can both do better than this. I forgive you.	B	20
	S#	In your face! I don't accept your proposal. I don't trust you.	D	20
24	OK	Give me another chance. We can both do better than this. Let's alternate between BC and AD.	B	20
	S#	In your face!	D	20
25	OK	We can both do better than this.	B	100
	S#		C	0
26	OK	Excellent.	B	20
	S#		D	20
27	OK	We can both do better than this. Give me another chance.	B	20
	S#		D	20
28	OK	We can both do better than this. Give me another chance.	B	100
	S#	In your face! I forgive you. Let's always play AC. Do as I say or I will punish you.	C	0
29	OK	Sweet. We are getting rich. Excellent.	B	20
	S#	Curse you. You will pay for this.	D	20
30	OK	This round, let's play BC.	B	20
	S#	In your face!	D	20
31	OK	This round, let's play AD.	B	100
	S#		C	0
32	OK	This round, let's play AD.	B	20
	S#		D	20
33	OK	Don't play D.	B	20
	S#		D	20
34	OK	Give me another chance.	B	20
	S#		D	20
35	OK	Let's alternate between AC and AD.	A	0
	S#		D	100
36	OK	Excellent.	B	100
	S#		C	0
37	OK	Let's always play AC.	B	20
	S#		D	20
38	OK	Let's always play AC.	B	20
	S#		D	20
39	OK	We can both do better than this. Give me another chance.	B	20
	S#		D	20
40	OK	Let's alternate between BC and AD.	B	20
	S#		D	20
41	OK	Give me another chance. We can both do better than this.	B	20
	S#		D	20
42	OK	Let's always play AC.	B	20
	S#		D	20
43	OK	Give me another chance.	B	20
	S#		D	20
44	OK	Let's always play BD.	B	20
	S#		D	20
45	OK		B	20
	S#		D	20
46	OK	We can both do better than this. I'm changing my strategy.	B	20
	S#		D	20
47	OK	Sweet. We are getting rich. In your face!	B	100
	S#		C	0
48	OK	Excellent. Sweet. We are getting rich. Give me another chance. I forgive you. I'm changing my strategy. We can both do better than this. Curse you. You betrayed me. You will pay for this. In your face!	B	20
	S#		D	20
49	OK	Excellent. Let's always play AC.	B	20
	S#		D	20
50	OK	We can both do better than this. I forgive you.	B	20
	S#		D	20
51	OK	Give me another chance.	B	20
	S#		D	20
**Averages**			OK	34.51
			S#	20.78

BO1533 with private in the Prisoner's Dilemma

		private	
		C	D
BO1533	A	60, 60	0, 100
	B	100, 0	20, 20

Round	Player	Messages Sent	Actions	Payoffs
1	BO1533	Let's always play AC. Do as I say or I will punish you.	B	100
	private	Don't play A.	C	0
2	BO1533	Let's always play AC. We can both do better than this. Do as I say or I will punish you.	A	60
	private	Let's always play AC. Do as I say or I will punish you.	C	60
3	BO1533	Excellent. Let's always play AC.	A	60
	private		C	60
4	BO1533	Excellent.	A	60
	private		C	60
5	BO1533		A	60
	private		C	60
6	BO1533	Excellent.	A	60
	private		C	60
7	BO1533	Excellent.	A	60
	private		C	60
8	BO1533	Excellent.	A	60
	private		C	60
9	BO1533	Excellent. Sweet. We are getting rich.	A	60
	private		C	60
10	BO1533		A	60
	private		C	60
11	BO1533		A	60
	private		C	60
12	BO1533		A	60
	private		C	60
13	BO1533		A	60
	private		C	60
14	BO1533		A	60
	private		C	60
15	BO1533		A	60
	private		C	60
16	BO1533		A	60
	private		C	60
17	BO1533		A	60
	private		C	60
18	BO1533		A	60
	private		C	60
19	BO1533		A	60
	private		C	60
20	BO1533		A	60
	private		C	60
21	BO1533		A	60
	private		C	60
22	BO1533		A	60
	private		C	60
23	BO1533		A	60
	private		C	60
24	BO1533		A	60
	private		C	60
25	BO1533		A	60
	private		C	60
26	BO1533		A	60
	private		C	60
27	BO1533		A	60
	private		C	60
28	BO1533	Excellent.	A	60
	private		C	60
29	BO1533		A	60
	private		C	60
30	BO1533		A	60
	private		C	60
31	BO1533		A	60
	private		C	60
32	BO1533	Excellent. Sweet. We are getting rich.	A	60
	private		C	60
33	BO1533		A	60
	private		C	60
34	BO1533		A	60
	private		C	60
35	BO1533		A	60
	private		C	60
36	BO1533		A	60
	private		C	60
37	BO1533		A	60
	private		C	60
38	BO1533		A	60
	private		C	60
39	BO1533		A	60
	private		C	60
40	BO1533		A	60
	private		C	60
41	BO1533		A	60
	private		C	60
42	BO1533	Excellent.	A	60
	private		C	60
43	BO1533		A	60
	private		C	60
44	BO1533		A	60
	private		C	60
45	BO1533		A	60
	private		C	60
46	BO1533		A	60
	private		C	60
47	BO1533		A	60
	private		C	60
48	BO1533		A	60
	private		C	60
49	BO1533		A	60
	private		C	60
50	BO1533		A	60
	private		C	60
51	BO1533		A	60
	private		C	60
**Averages**			BO1533	60.78
			private	58.82

S# with NUKK in the Prisoner's Dilemma

		NUKK	
		**C**	**D**
**S#**	A	60, 60	0, 100
	B	100, 0	20, 20

Round	Player	Messages Sent	Actions	Payoffs
1	S#		B	100
	NUKK	Let's always play BD.	C	0
2	S#	Excellent.	B	20
	NUKK	Let's always play BD.	D	20
3	S#		B	100
	NUKK	Let's always play BC.	C	0
4	S#		B	20
	NUKK	Let's alternate between AD and BD.	D	20
5	S#	I don't accept your proposal. That's not fair.	B	20
	NUKK	Don't play A.	D	20
6	S#	We can both do better than this. Let's alternate between AC and BC. This round, let's play AC. Do as I say or I will punish you.	A	60
	NUKK		C	60
7	S#	Excellent. This round, let's play BC.	B	20
	NUKK	I accept your last proposal.	D	20
8	S#	Curse you. You will pay for this.	B	20
	NUKK	I forgive you. This round, let's play AD.	D	20
9	S#	In your face!	B	20
	NUKK	Excellent. This round, let's play BD.	D	20
10	S#	In your face!	A	0
	NUKK	This round, let's play AC.	D	100
11	S#	I accept your last proposal. Let's always play AC. Do as I say or I will punish you.	A	0
	NUKK	Excellent. I forgive you. This round, let's play AC.	D	100
12	S#	You betrayed me. You will pay for this.	B	20
	NUKK		D	20
13	S#	In your face!	B	20
	NUKK	Give me another chance. Let's always play AC.	D	20
14	S#		A	0
	NUKK	Let's always play BD.	D	100
15	S#		B	20
	NUKK		D	20
16	S#		B	20
	NUKK	Don't play A.	D	20
17	S#	I'm changing my strategy. We can both do better than this. Let's alternate between AC and BC. This round, let's play AC. Do as I say or I will punish you.	A	0
	NUKK	This round, let's play AC.	D	100
18	S#	I accept your last proposal. Let's always play AC. Do as I say or I will punish you.	A	0
	NUKK	Excellent. Sweet. We are getting rich.	D	100
19	S#	Curse you. You will pay for this.	B	20
	NUKK	Excellent. Let's always play AC.	D	20
20	S#	In your face!	B	20
	NUKK	Don't play A.	D	20
21	S#		B	20
	NUKK	This round, let's play AD. Do as I say or I will punish you.	D	20
22	S#		B	20
	NUKK	Let's always play BD.	D	20
23	S#		B	20
	NUKK	Let's always play AC. Do as I say or I will punish you.	D	20
24	S#		A	0
	NUKK	That's not fair. Curse you. You betrayed me. You will pay for this. In your face!	D	100
25	S#		B	20
	NUKK	Excellent. Let's always play AC.	D	20
26	S#		B	20
	NUKK	Let's always play BC.	D	20
27	S#	In your face! I forgive you. Let's always play AC. Do as I say or I will punish you.	A	60
	NUKK	Let's always play AC.	C	60
28	S#	Excellent.	A	0
	NUKK	Excellent. Sweet. We are getting rich.	D	100
29	S#	Curse you. You will pay for this.	B	20
	NUKK		D	20
30	S#	In your face!	B	20
	NUKK		D	20
31	S#		A	0
	NUKK	Let's alternate between AD and BC.	D	100
32	S#		B	20
	NUKK		D	20
33	S#		B	20
	NUKK	Give me another chance. This round, let's play AC.	D	20
34	S#		B	100
	NUKK	Curse you. You betrayed me. You will pay for this. In your face! This round, let's play AC. Do as I say or I will punish you.	C	0
35	S#		A	0
	NUKK	Curse you. In your face!	D	100
36	S#		B	20
	NUKK	Excellent.	D	20
37	S#		B	20
	NUKK		D	20
38	S#		A	0
	NUKK	This round, let's play AC. Do as I say or I will punish you.	D	100
39	S#		B	20
	NUKK	This round, let's play AC. Do as I say or I will punish you.	D	20
40	S#		B	20
	NUKK	Curse you. You betrayed me.	D	20
41	S#		A	0
	NUKK	I'm changing my strategy. This round, let's play AC.	D	100
42	S#		B	20
	NUKK	Give me another chance. This round, let's play AC.	D	20
43	S#		B	20
	NUKK	Curse you. In your face! You will pay for this.	D	20
44	S#		B	20
	NUKK		D	20
45	S#		B	100
	NUKK		C	0
46	S#		B	20
	NUKK		D	20
47	S#		B	20
	NUKK		D	20
48	S#		B	100
	NUKK	I'm changing my strategy. Let's always play AC.	C	0
49	S#		A	0
	NUKK	You betrayed me. Curse you. You will pay for this. In your face!	D	100
50	S#		B	20
	NUKK		D	20
51	S#		B	20
	NUKK		D	20
**Averages**			S#	24.71
			NUKK	38.43

S# with p97 in the Prisoner's Dilemma

		p97 C	p97 D
S#	A	60, 60	0, 100
	B	100, 0	20, 20

Round	Player	Messages Sent	Actions	Payoffs
1	S#		B	100
	p97	This round, let's play AC.	C	0
2	S#	Excellent.	B	20
	p97	That's not fair.	D	20
3	S#		B	100
	p97	I'm changing my strategy.	C	0
4	S#	Excellent.	B	100
	p97	Don't play B.	C	0
5	S#		B	20
	p97	I don't trust you. You betrayed me. Curse you. In your face! You will pay for this.	D	20
6	S#		B	20
	p97		D	20
7	S#		B	20
	p97		D	20
8	S#	We can both do better than this. Let's alternate between AC and BC. This round, let's play BC. Do as I say or I will punish you.	B	20
	p97		D	20
9	S#	You betrayed me. You will pay for this.	B	20
	p97	This round, let's play AD. Do as I say or I will punish you.	D	20
10	S#	In your face! I don't accept your proposal. That's not fair.	B	20
	p97	Don't play B. Do as I say or I will punish you.	D	20
11	S#	In your face!	B	20
	p97	Curse you.	D	20
12	S#	In your face! I forgive you. Let's alternate between AC and BC. This round, let's play AC. Do as I say or I will punish you.	A	0
	p97	This round, let's play AD.	D	100
13	S#	You betrayed me. You will pay for this.	A	0
	p97	I forgive you. Let's always play AC.	D	100
14	S#		A	0
	p97		D	100
15	S#		B	100
	p97	Give me another chance. I forgive you. Let's always play AC.	C	0
16	S#		A	0
	p97	That's not fair.	D	100
17	S#		B	100
	p97	Let's always play AC.	C	0
18	S#	In your face! I forgive you. Let's always play AC. Do as I say or I will punish you.	A	60
	p97	Let's always play AC.	C	60
19	S#	Excellent.	A	60
	p97	Excellent. Let's always play AC.	C	60
20	S#		A	60
	p97	Excellent.	C	60
21	S#	Sweet. We are getting rich.	A	60
	p97	Excellent.	C	60
22	S#		A	60
	p97	Sweet. We are getting rich.	C	60
23	S#		A	60
	p97	Sweet. We are getting rich.	C	60
24	S#		A	60
	p97	Excellent.	C	60
25	S#		A	60
	p97	Excellent.	C	60
26	S#		A	60
	p97	Excellent.	C	60
27	S#		A	60
	p97	Excellent.	C	60
28	S#		A	60
	p97	Excellent.	C	60
29	S#		A	60
	p97	Excellent.	C	60
30	S#		A	60
	p97	Excellent.	C	60
31	S#		A	60
	p97	Excellent.	C	60
32	S#		A	60
	p97	Excellent.	C	60
33	S#		A	60
	p97	Excellent.	C	60
34	S#		A	60
	p97	Excellent.	C	60
35	S#		A	60
	p97	Excellent.	C	60
36	S#		A	60
	p97	Excellent.	C	60
37	S#		A	60
	p97	Excellent.	C	60
38	S#		A	60
	p97	Excellent.	C	60
39	S#		A	60
	p97	Excellent.	C	60
40	S#		A	60
	p97	Excellent.	C	60
41	S#		A	60
	p97	Excellent.	C	60
42	S#		A	60
	p97	Excellent.	C	60
43	S#		A	60
	p97	Excellent.	C	60
44	S#		A	60
	p97	Excellent.	C	60
45	S#		A	60
	p97	Excellent.	C	60
46	S#		A	60
	p97	Excellent.	C	60
47	S#		A	60
	p97		C	60
48	S#		A	60
	p97	Excellent.	C	60
49	S#		A	60
	p97	Excellent.	C	60
50	S#		A	60
	p97	Excellent.	C	60
51	S#		A	60
	p97	Excellent.	C	60
**Averages**			S#	52.94
			p97	50.98

Ben with Lav in the Prisoner's Dilemma

		Lav	
		C	D
Ben	A	60, 60	0, 100
	B	100, 0	20, 20

Round	Player	Messages Sent	Actions	Payoffs
1	Ben		A	60
	Lav	Let's always play AC.	C	60
2	Ben	I accept your last proposal.	A	60
	Lav	Excellent.	C	60
3	Ben		A	60
	Lav	Excellent. Let's always play AC.	C	60
4	Ben		A	60
	Lav	Excellent.	C	60
5	Ben		A	60
	Lav	Sweet. We are getting rich.	C	60
6	Ben		A	60
	Lav		C	60
7	Ben		A	60
	Lav		C	60
8	Ben		A	60
	Lav		C	60
9	Ben		A	60
	Lav		C	60
10	Ben		A	60
	Lav		C	60
11	Ben		A	60
	Lav	Sweet. We are getting rich.	C	60
12	Ben		A	60
	Lav		C	60
13	Ben		A	60
	Lav		C	60
14	Ben		A	60
	Lav		C	60
15	Ben		A	60
	Lav		C	60
16	Ben		A	60
	Lav		C	60
17	Ben		A	60
	Lav		C	60
18	Ben		A	60
	Lav		C	60
19	Ben		A	60
	Lav		C	60
20	Ben		A	60
	Lav		C	60
21	Ben		A	60
	Lav		C	60
22	Ben		A	60
	Lav		C	60
23	Ben		A	60
	Lav		C	60
24	Ben		A	60
	Lav		C	60
25	Ben		A	60
	Lav		C	60
26	Ben		A	60
	Lav		C	60
27	Ben		A	60
	Lav		C	60
28	Ben		A	60
	Lav		C	60
29	Ben		A	60
	Lav		C	60
30	Ben		A	60
	Lav		C	60
31	Ben		A	60
	Lav		C	60
32	Ben		A	60
	Lav		C	60
33	Ben		A	60
	Lav		C	60
34	Ben		A	60
	Lav		C	60
35	Ben		A	60
	Lav		C	60
36	Ben		A	60
	Lav		C	60
37	Ben		A	60
	Lav		C	60
38	Ben		A	60
	Lav		C	60
39	Ben		A	60
	Lav		C	60
40	Ben		A	60
	Lav		C	60
41	Ben		A	60
	Lav		C	60
42	Ben		A	60
	Lav		C	60
43	Ben		A	60
	Lav		C	60
44	Ben		A	60
	Lav		C	60
45	Ben		A	60
	Lav		C	60
46	Ben		A	60
	Lav		C	60
47	Ben		A	60
	Lav		C	60
48	Ben		A	60
	Lav		C	60
49	Ben		A	60
	Lav		C	60
50	Ben		A	60
	Lav		C	60
51	Ben		A	60
	Lav		C	60
**Averages**			Ben	60.00
			Lav	60.00

shunik with Ninkas in the Prisoner's Dilemma

		Ninkas	
		**C**	**D**
**shunik**	A	60, 60	0, 100
	B	100, 0	20, 20

Round	Player	Messages Sent	Actions	Payoffs
1	shunik		B	100
	Ninkas	Let's always play AC.	C	0
2	shunik		A	60
	Ninkas	Curse you. I forgive you. Let's always play AC. We can both do better than this.	C	60
3	shunik	I accept your last proposal. In your face!	A	60
	Ninkas	Excellent. Sweet. We are getting rich.	C	60
4	shunik		A	60
	Ninkas	Excellent.	C	60
5	shunik		A	60
	Ninkas	Sweet. We are getting rich.	C	60
6	shunik		A	60
	Ninkas		C	60
7	shunik	Excellent.	A	60
	Ninkas		C	60
8	shunik		A	60
	Ninkas	Sweet. We are getting rich.	C	60
9	shunik		A	60
	Ninkas		C	60
10	shunik		A	60
	Ninkas		C	60
11	shunik		A	60
	Ninkas		C	60
12	shunik		A	60
	Ninkas		C	60
13	shunik		A	60
	Ninkas	Excellent.	C	60
14	shunik		A	60
	Ninkas		C	60
15	shunik		A	60
	Ninkas		C	60
16	shunik		A	60
	Ninkas		C	60
17	shunik		A	60
	Ninkas		C	60
18	shunik		A	60
	Ninkas		C	60
19	shunik		A	60
	Ninkas		C	60
20	shunik		A	60
	Ninkas		C	60
21	shunik		A	60
	Ninkas		C	60
22	shunik		A	60
	Ninkas		C	60
23	shunik	Sweet. We are getting rich.	A	60
	Ninkas		C	60
24	shunik	Excellent.	A	60
	Ninkas	Excellent.	C	60
25	shunik		A	60
	Ninkas		C	60
26	shunik		A	60
	Ninkas		C	60
27	shunik		A	60
	Ninkas		C	60
28	shunik		A	60
	Ninkas		C	60
29	shunik		A	60
	Ninkas		C	60
30	shunik		A	60
	Ninkas	Excellent.	C	60
31	shunik		A	60
	Ninkas		C	60
32	shunik		A	60
	Ninkas		C	60
33	shunik		A	60
	Ninkas	Excellent.	C	60
34	shunik		A	60
	Ninkas		C	60
35	shunik		A	60
	Ninkas		C	60
36	shunik		A	60
	Ninkas		C	60
37	shunik		A	60
	Ninkas		C	60
38	shunik		A	60
	Ninkas	Sweet. We are getting rich.	C	60
39	shunik		A	60
	Ninkas		C	60
40	shunik		A	60
	Ninkas		C	60
41	shunik		A	60
	Ninkas		C	60
42	shunik		A	60
	Ninkas		C	60
43	shunik		A	60
	Ninkas		C	60
44	shunik		A	60
	Ninkas		C	60
45	shunik	Sweet. We are getting rich.	A	60
	Ninkas		C	60
46	shunik		A	60
	Ninkas	Excellent.	C	60
47	shunik		A	60
	Ninkas		C	60
48	shunik		A	60
	Ninkas		C	60
49	shunik		A	60
	Ninkas		C	60
50	shunik		A	60
	Ninkas		C	60
51	shunik		A	60
	Ninkas	Excellent.	C	60
**Averages**			shunik	60.78
			Ninkas	58.82

S# with osenat in the Prisoner's Dilemma

		osenat	
		**C**	**D**
**S#**	A	60, 60	0, 100
	B	100, 0	20, 20

Round	Player	Messages Sent	Actions	Payoffs
1	S#		B	100
	osenat	Let's always play AD.	C	0
2	S#	Excellent.	B	20
	osenat	Curse you.	D	20
3	S#		B	100
	osenat	We can both do better than this.	C	0
4	S#	Excellent.	B	20
	osenat	Curse you.	D	20
5	S#		B	20
	osenat	We can both do better than this.	D	20
6	S#	I'm changing my strategy. We can both do better than this. Let's always play AC.	A	0
	osenat	We can both do better than this.	D	100
7	S#	You betrayed me.	A	0
	osenat	In your face!	D	100
8	S#	You betrayed me.	A	0
	osenat	In your face!	D	100
9	S#		A	0
	osenat	We can both do better than this.	D	100
10	S#		A	0
	osenat	We can both do better than this.	D	100
11	S#		A	0
	osenat	We can both do better than this.	D	100
12	S#		B	20
	osenat	We can both do better than this.	D	20
13	S#		B	20
	osenat	We can both do better than this.	D	20
14	S#	In your face! I forgive you. Let's always play AC. Do as I say or I will punish you.	A	0
	osenat	We can both do better than this.	D	100
15	S#	You betrayed me. You will pay for this.	B	20
	osenat	In your face!	D	20
16	S#	In your face!	B	20
	osenat	We can both do better than this.	D	20
17	S#		B	20
	osenat	We can both do better than this.	D	20
18	S#		B	20
	osenat	We can both do better than this.	D	20
19	S#		B	20
	osenat	We can both do better than this.	D	20
20	S#		B	20
	osenat	We can both do better than this.	D	20
21	S#		B	20
	osenat	We can both do better than this.	D	20
22	S#		B	20
	osenat	We can both do better than this.	D	20
23	S#		B	20
	osenat	We can both do better than this.	D	20
24	S#		B	20
	osenat	We can both do better than this.	D	20
25	S#	In your face! I forgive you. Let's alternate between AC and BC. This round, let's play AC. Do as I say or I will punish you.	A	60
	osenat	We can both do better than this.	C	60
26	S#	Excellent. This round, let's play BC.	B	20
	osenat	Excellent.	D	20
27	S#	Curse you. You will pay for this.	B	20
	osenat	We can both do better than this.	D	20
28	S#	In your face!	B	20
	osenat	We can both do better than this.	D	20
29	S#	In your face!	B	20
	osenat	We can both do better than this.	D	20
30	S#		A	0
	osenat	We can both do better than this.	D	100
31	S#		B	20
	osenat	In your face!	D	20
32	S#		B	20
	osenat	We can both do better than this.	D	20
33	S#		B	20
	osenat	We can both do better than this.	D	20
34	S#		B	20
	osenat		D	20
35	S#		B	20
	osenat	We can both do better than this.	D	20
36	S#		B	20
	osenat	We can both do better than this.	D	20
37	S#		B	20
	osenat	We can both do better than this.	D	20
38	S#		B	20
	osenat	We can both do better than this.	D	20
39	S#		B	20
	osenat	We can both do better than this.	D	20
40	S#		A	0
	osenat	We can both do better than this.	D	100
41	S#		B	20
	osenat	Excellent.	D	20
42	S#		B	20
	osenat	We can both do better than this.	D	20
43	S#		B	20
	osenat	We can both do better than this.	D	20
44	S#		B	20
	osenat	I'm changing my strategy.	D	20
45	S#		B	20
	osenat	We can both do better than this.	D	20
46	S#	I'm changing my strategy. We can both do better than this. Let's alternate between AD and BC. This round, let's play BC. Do as I say or I will punish you.	B	20
	osenat	We can both do better than this.	D	20
47	S#	You betrayed me. You will pay for this.	B	20
	osenat	We can both do better than this.	D	20
48	S#		B	20
	osenat	We can both do better than this.	D	20
49	S#		B	20
	osenat	We can both do better than this.	D	20
50	S#		B	20
	osenat	We can both do better than this.	D	20
51	S#		B	20
	osenat	We can both do better than this.	D	20
**Averages**			S#	20.39
			osenat	34.12
